# Genetic Correlation and Causal Inference Between Female Fat Distribution and Preeclampsia: An Integrative Genomic Study

**DOI:** 10.1096/fj.202601888R

**Published:** 2026-06-23

**Authors:** Man Wang, Li Huang, Danfeng Zhang, Fengmei Yang, Lihua Wu, Bo Gao

**Affiliations:** ^1^ Department of Outpatient, Hubei Provincial Clinical Research Center for Umbilical Cord Blood Hematopoietic Stem Cells Taihe Hospital, Hubei University of Medicine Shiyan Hubei China; ^2^ Department of Obstetrics and Gynecology Taihe Hospital, Hubei University of Medicine Shiyan Hubei China; ^3^ Department of General Surgery Taihe Hospital, Hubei University of Medicine Shiyan Hubei China; ^4^ Department of Obstetrics and Gynecology The First Affiliated Hospital of Xi'an Jiaotong University Xi'an Shanxi China; ^5^ Department of Medical Laboratory Taihe Hospital, Hubei University of Medicine Shiyan Hubei China; ^6^ Brain Research Institute, Research Center of Neurological Diseases Taihe Hospital, Hubei University of Medicine Shiyan Hubei China; ^7^ Department of Assessment Office Taihe Hospital, Hubei University of Medicine Shiyan Hubei China

**Keywords:** fat distribution, genetic correlation, Mendelian randomization, preeclampsia, waist‐hip ratio

## Abstract

Preeclampsia (PE) is a major cause of maternal and perinatal morbidity. Because abnormal fat distribution is closely related to metabolic dysfunction, vascular injury, and hypertensive disorders during pregnancy, clarifying its genetic relationship with PE may improve our understanding of adverse pregnancy outcomes. Here, we investigated the shared genetic architecture between PE and waist–hip ratio adjusted for body mass index (WHRadjBMI) by integrating large‐scale genome‐wide association study (GWAS) summary statistics for female WHRadjBMI from the GIANT consortium (*n* ≈ 700 000) and PE from FinnGen R11 (7955 cases and 124 764 controls). Analyses included genome‐wide genetic correlation, polygenic overlap, local genetic correlation, cross‐trait GWAS meta‐analysis, tissue/cell type enrichment, functional annotation, and Mendelian randomization, using tools including linkage disequilibrium score regression (LDSC), MiXeR, LAVA, ρ‐HESS, MTAG, CPASSOC, and conjunctional false discovery rate (conjFDR). We identified approximately 0.5 k shared causal variants; MiXeR detected a modest but significant polygenic overlap (rg = 0.08, *p* = 0.006), whereas LDSC showed no significant genome‐wide correlation. A shared genetic locus near MTHFR‐CLCN6 (rs17367504) was detected, consistent with known PE biology. Enrichment analyses implicated VEGFA‐driven vascular and immune processes, with uterine pericytes displaying the strongest shared cell‐type enrichment. Mendelian randomization supported a causal effect of WHRadjBMI on PE (IVW: *p* = 2.7 × 10^−4^) but not reverse causation. These findings suggest that genetically predicted fat distribution may contribute to PE susceptibility and highlight shared vascular–immune pathways that may link WHR‐related genetic risk to pregnancy complications.

## Graphical abstract

We conducted integrative genomic analyses of large‐scale GWAS summary data to explore the shared polygenic architecture and causal link between female fat distribution (WHRadjBMI) and preeclampsia, identifying novel loci and critical vascular–immune pathways with implications for risk prediction.

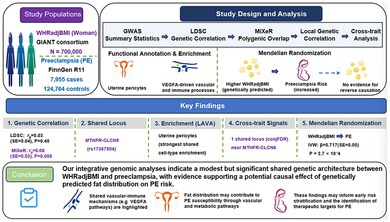

## 1 Introduction

Preeclampsia (PE) is a pregnancy‐specific hypertensive disorder and one of the leading causes of maternal mortality worldwide [1]. Obesity has been identified as an important risk factor for PE [2, 3]. Although the precise pathogenesis of PE remains incompletely understood, several biological processes have been implicated, including an imbalance between vascular endothelial growth factor A (VEGFA) and soluble Fms‐like tyrosine kinase‐1 (sFlt1), placental oxidative stress, and systemic immune activation [4].

Waist–hip ratio (WHR) is a key anthropometric indicator of body fat distribution and, compared with body mass index (BMI), provides a more accurate reflection of obesity‐related health risks [5]. In cardiovascular and metabolic research, WHR has been shown to outperform BMI by more precisely capturing visceral adiposity and by demonstrating superior predictive power for outcomes such as stroke, myocardial infarction, and cardiovascular mortality [6, 7]. Epidemiological studies further support its relevance to pregnancy complications: a prospective cohort reported that women with WHR ≥ 0.85 in early pregnancy had an approximately 2.3‐fold higher risk of PE compared with women with normal WHR [8], and a meta‐analysis of over 80 000 pregnancies confirmed that higher WHR significantly increases the risk of hypertensive disorders of pregnancy, including PE [9]. These findings suggest that fat distribution is closely linked to PE risk, positioning WHR as a potential early predictor of adverse pregnancy outcomes.

Central obesity (high WHR) may promote the development of PE through multiple biological pathways. Visceral adipose tissue secretes pro‐inflammatory and pro‐coagulant mediators that drive glucose dysregulation and insulin resistance [10, 11], while increased abdominal visceral fat thickness has been directly associated with elevated PE risk [12]. From a genetic perspective, body fat distribution traits such as WHR exhibit substantial heritability, and numerous genome‐wide association studies (GWAS) have identified loci strongly associated with WHR. This raises the possibility that WHR and PE may share a genetic basis. Collectively, these observations point to a potential shared genetic architecture between fat distribution and PE.

Building on these findings, this study aimed to investigate the shared genetic architecture between WHR adjusted for BMI in women (hereafter referred to as WHR) and PE by leveraging large‐scale GWAS summary statistics. In contrast to prior studies that focused primarily on epidemiological associations or isolated genetic factors, we integrated genetic correlation, causal inference, and functional annotation to comprehensively dissect the genetic overlap between WHR and PE. Through this approach, we sought to bridge the gap between epidemiological observations and molecular mechanisms, thereby providing novel insights into their comorbidity and underlying biological pathways.

## 2 Methods

### 2.1 Study Design

This study leveraged summary‐level GWAS data to investigate the potential causal relationship and shared genetic architecture between fat distribution and PE. Female WHR was used as a proxy measure of fat distribution. First, we applied MiXeR and linkage disequilibrium score regression (LDSC) to estimate genetic correlation, polygenic overlap, and sample overlap between WHR and PE. We then employed local genetic correlation analyses using LAVA and ρ‐HESS to dissect regional genetic correlations. Shared risk SNPs between WHR and PE were identified through cross‐trait GWAS meta‐analysis methods (MTAG and CPASSOC) and the conjunctional false discovery rate (conjFDR) approach. Next, we used LDSC‐seg and sclinker to integrate bulk transcriptomic data from the Genotype‐Tissue Expression (GTEx) project and single‐cell RNA sequencing (scRNA‐seq) datasets, aiming to characterize tissue‐ and cell type–specific genetic associations. We further applied gene set analysis (GSA)–MiXeR to assess SNP heritability enrichment at the pathway level and identify relevant biological processes. Two‐sample Mendelian randomization (MR) was subsequently conducted to evaluate causal effects between WHR and PE. Finally, we performed a transcriptome‐wide association study (TWAS) to prioritize putative shared risk genes.

### 2.2 GWAS Datasets

Summary statistics for female WHR were obtained from the Genetic Investigation of Anthropometric Traits (GIANT) consortium, with SNPs associated with BMI excluded to isolate the specific genetic component of WHR [13, 14]. PE GWAS data were derived from the FinnGen consortium (release R11; r11.risteys.finregistry.fi). Table S1 summarizes the disease endpoint codes, diagnostic definitions, and case–control numbers. All participants were of European ancestry. Female WHR data were based on women aged 40–69 years, whereas PE cases were from women aged 10–50 years.

### 2.3 Transcriptomic Data

We utilized bulk RNA‐seq data from GTEx v8, which covers 54 non‐diseased human tissues, in combination with cis‐eQTL data from GTEx v8 and summary statistics from eQTLGen, a large‐scale meta‐analysis comprising 14 115 individuals, to identify genetic variants associated with gene expression and evaluate their regulatory effects [15].

### 2.4 Single‐Cell Data

Publicly available scRNA‐seq datasets were used to obtain cellular resolution profiles from human whole blood (85 233 cells), adipose tissue (94 415 cells), uterus (21 198 cells), and ovary (22 029 cells) for cell type–specific enrichment analyses [16, 17].

### 2.5 Data Analysis

#### 2.5.1 Genome‐Wide Genetic Correlation Analysis

We first applied LDSC, a method robust to sample overlap, to estimate genome‐wide genetic correlations based on GWAS summary statistics [18, 19]. SNPs with a minor allele frequency (MAF) ≤ 0.01 were excluded, and analyses were performed on cleaned GWAS summary data. We estimated SNP‐based heritability (*h* ^*2*^) for WHR and PE, and then used precomputed LD scores from the 1000 Genomes Project to calculate the cross‐trait genetic correlation. A bivariate LDSC without intercept constraints was subsequently conducted to estimate the genetic correlation coefficient (𝑟_g_) between WHR and PE. Significant correlations were defined at *p* < 0.05. LDSC also provided estimates of potential sample overlap between the two GWAS datasets.

#### 2.5.2 Polygenic Overlap Analysis

To quantify polygenic overlap between WHR and PE, we used MiXeR, which estimates the total number of causal variants across traits and partitions SNPs into four categories: (i) null SNPs with no effect, (ii) trait‐specific SNPs for WHR, (iii) trait‐specific SNPs for PE, and (iv) shared SNPs influencing both traits. The degree of polygenic overlap was quantified on a scale from 0%–100%.

#### 2.5.3 Local Genetic Correlation Analysis

We next performed local genetic correlation analysis using LAVA, a statistical framework for estimating regional correlations across the genome, providing a more granular view of local architecture [20]. Bivariate LAVA analyses were conducted only in regions where both traits exhibited significant univariate heritability, with significance assessed using Benjamini–Hochberg (BH) false discovery rate (FDR) correction. Subsequently, to obtain an independent partitioning scheme and complementary validation, we applied ρ‐HESS, which identified approximately 1700 LD‐independent regions based on the European LD reference panel. Within these regions, we estimated SNP‐based heritability and local genetic correlations (PMID: 29100087).

#### 2.5.4 Shared Association Loci Analysis

We performed two complementary cross‐trait meta‐analysis approaches—multi‐trait analysis of GWAS (MTAG) and cross‐phenotype association test (CPASSOC)—to identify shared loci between WHR and PE. MTAG enhances statistical power by jointly estimating the genotype–phenotype variance–covariance matrix and produces trait‐specific SNP effect estimates [21, 22]. SNPs with MAF < 0.01 or sample size *N* < (2/3) × 90th percentile were excluded [23]. Given potential sample overlap, we applied bivariate LDSC adjustment. As a sensitivity analysis, CPASSOC was also used to integrate association evidence across traits. Independent SNPs were identified using PLINK with an LD threshold of r^2^ = 0.2. Loci reaching genome‐wide significance (*p* < 5 × 10^−8^) and located in regions of significant local correlation were prioritized.

To further refine locus discovery, we applied the conditional/conjunctional false discovery rate (condFDR/conjFDR) framework. ConjFDR identifies SNPs jointly associated with both traits at PFDR < 0.05. Conditional Q–Q plots were used to visualize cross‐trait enrichment, with increasing leftward deviation from the null line indicating stronger polygenic enrichment as secondary trait significance thresholds (*p* < 0.1, 0.01, 0.001) increased.

#### 2.5.5 Causal Inference

We conducted bidirectional two‐sample MR to test causal relationships while minimizing confounding and reverse causation [24, 25]. For WHR, genome‐wide significant (*p* ≤ 5 × 10^−8^), independent SNPs (LD r^2^ = 0.001, kb = 10 000) were selected as instrumental variables (IVs) using PLINK with the 1000 Genomes Project as reference. The Steiger test was applied to exclude IVs with incorrect causal direction. Causal effects were primarily estimated using inverse variance–weighted (IVW) MR, with MR‐Egger, Bayesian weighted MR (BWMR), weighted median (WM), and robust adjusted profile score (RAPS) as complementary methods. IV strength was evaluated by the F‐statistic (𝐹 = BETA^2^ / SE^2^), with F > 10 indicating sufficient instrument strength [26].

Reverse‐direction MR was performed using SNPs associated with PE at *p* < 1 × 10^−8^ (*r* ^2^ = 0.001, kb = 10 000). Given the relationships among gestational hypertension, blood pressure, WHR, and PE, multivariable MR was also conducted using systolic blood pressure and gestational diabetes GWAS as mediators. Sensitivity analyses assessed pleiotropy and heterogeneity: MR‐PRESSO was applied for pleiotropy detection (*p* < 0.05 indicating pleiotropy) [27], and Cochran's Q test was used for heterogeneity (*p* > 0.05 suggesting no heterogeneity) [28].

#### 2.5.6 Tissue‐Level SNP Heritability Enrichment

We used stratified LDSC (S‐LDSC) with GTEx v8 transcriptomic annotations to quantify SNP heritability enrichment across 54 human tissues. Enrichment significance was evaluated using Z‐score *p*‐values, with Bonferroni correction applied. Tissues with PFDR < 0.05 were considered significantly enriched for both WHR and PE.

#### 2.5.7 Cell‐Level SNP Heritability Enrichment

We applied MAGMA (v1.08) in combination with sc‐linker, a framework integrating scRNA‐seq, GWAS, and epigenomic annotations, to infer cell type–specific contributions [29, 30]. Single‐cell datasets from uterus and ovary were used to evaluate enrichment of WHR‐ and PE‐associated SNPs across cell types. Adjusted scRNA‐seq mean expression profiles were integrated with MAGMA results, and significant enrichments were defined at PFDR < 0.05 after BH correction.

#### 2.5.8 Functional Partitioning of Heritability

We applied GSA–MiXeR, a competitive gene set enrichment framework extending MiXeR, to partition SNP heritability and quantify multiplicative enrichment attributable to curated gene sets [31]. Candidate gene sets were first selected from MAGMA analyses for WHR and PE. GSA‐MiXeR enrichment estimates were then used to quantify the number of enriched sets, fold enrichment, and average gene counts. These were subsequently mapped to biological pathways to identify functional processes shared between WHR and PE.

#### 2.5.9 Transcriptome‐Wide Association Study (TWAS)

To prioritize putative shared risk genes, we performed TWAS using FUSION [32]. Single‐trait and cross‐trait TWAS were performed for WHR and PE separately and jointly [33]. Expression weights from uterus, ovary, whole blood, and hypothalamus were integrated with GTEx v8 transcriptomic data. Shared gene–tissue pairs were identified by intersecting single‐trait TWAS results. Bonferroni correction was applied, with significance defined as *p* < 0.05/𝑛.

### 2.6 Code Availability

This research used publicly available software and code, including:

Plink (https://www.cog‐genomics.org/plink/).

LDSC (https://github.com/bulik/ldsc).

LDSC (v1.0.1) (https://github.com/bulik/ldsc).

MiXeR (v1.0.0) (https://github.com/precimed/mixer).

ρ‐HESS (https://github.com/huwenboshi/hess).

LAVA (https://github.com/josefin‐werme/LAVA).

LDSC‐seg (https://gwaslab.org/2022/03/14/ldsc‐seg).

MTAG (https://github.com/JonJala/mtag).

CPASSOC (https://github.com/futurologist/UKB_phenotypes_and_scripts/).

ConjFDR (https://github.com/precimed/pleiofdr/blob/master/fuma/conj_fuma_combined_novelty.py).

LDSC‐SEG (https://github.com/bulik/ldsc/wiki/Cell‐type‐specific‐analyses).

Sclinker (https://github.com/karthikj89/scgenetics?tab=readme‐ov‐file).

GSA‐MiXeR (https://github.com/precimed/gsa‐mixer).

TwosampleMR (v0.6.6)(https://github.com/MRCIEU/TwoSampleMR).

TWAS (https://github.com/gusevlab/fusion_twas).

## 3 Results

### 3.1 Genome‐Wide Genetic Correlation and Polygenic Overlap

Using univariate LDSC without constraining the intercept, the observed‐scale SNP‐based h^2^ of WHR and PE was estimated at 0.202 and 0.035, respectively. Bivariate LDSC indicated no significant genome‐wide genetic correlation between the two traits (r_g_ = 0.045, SE = 0.0497, *p* = 0.368); when constraining the univariate intercepts to 1, the estimate remained non‐significant (r_g_ = 0.031, SE = 0.0401, *p* = 0.434). Cross‐trait intercepts were close to zero and not significant, suggesting minimal sample overlap. Overall, we did not observe significant genome‐wide genetic correlation, indicating limited polygenic sharing between WHR and PE.

**FIGURE 1:**
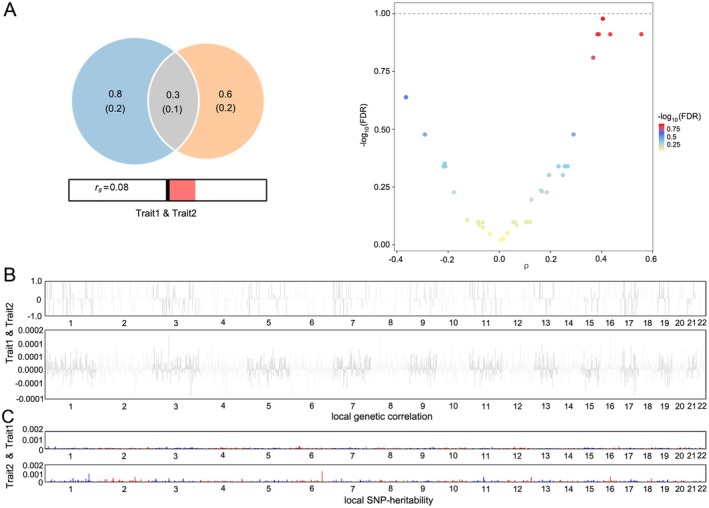
Integrative analysis of shared genetic architecture between WHR and PE. (A) Genome‐wide polygenic overlap estimated by MiXeR, showing SNP‐heritability of WHR (blue) and PE (orange), as well as their genetic correlation (*r*_g_ = 0.08). Numbers inside circles indicate the proportion of heritability explained (standard errors in parentheses). (B) Local genetic correlation (ρ) across genomic regions calculated by ρ‐HESS, with statistical significance threshold indicated by the dashed horizontal line (−log_10_(FDR)). (C) The Manhattan plot of the estimates of local genetic correlation and local genetic covariance between WHR and PE, and local SNP heritability of WHR and PE, respectively. Trait1: WHR, Trait2: PE.

Given the relatively low h^2^ for PE and thus limited statistical power, we further applied MiXeR. Univariate MiXeR estimated ~1.1 k (SD = 0.2 k) causal variants for WHR and ~0.9 k (SD = 0.2 k) for PE, with ~0.3 k (SD = 0.1 k) variants shared between the two traits (Figure 1A; Table S2). In the bivariate MiXeR model, we detected a modest but significant positive correlation (*r*_g_ = 0.08, *p* = 0.006), consistent in direction with LDSC. Taken together, LDSC did not detect a significant genome‐wide genetic correlation between WHR and PE, whereas MiXeR suggested a modest polygenic overlap. Therefore, the overall genome‐wide sharing between WHR and PE appears limited in magnitude, although a subset of shared causal variants may exist.

### 3.2 Local Genetic Correlation

Based on the default hg19 partitioning (1957 loci), LAVA estimated ~1% sample overlap, consistent with previous reports. Bivariate tests were performed only in regions with significant univariate heritability for both traits. After multiple testing correction, no loci reached significance (Figure 1B, Table S3).

To replicate these findings, we applied ρ‐HESS using the same partitioning scheme. Similarly, no loci survived multiple testing correction, although one region on chromosome 14 (22760701–23 985 936) showed nominal significance prior to adjustment (Figure 1C, Table S4).

Taken together, within the sample size and statistical power of this study, we did not detect robust local genetic correlation between WHR and PE. If such correlations exist, they are likely modest in magnitude or restricted to a few genomic regions, insufficient to withstand multiple testing correction.

### 3.3 Identification of Shared Risk SNPs

To identify shared loci, we performed cross‐trait GWAS meta‐analyses using MTAG and CPASSOC. We prioritized loci reaching genome‐wide significance (*p* < 5 × 10^−8^) in both methods. Two SNPs, rs17037390 and rs12567136, were identified; however, both had already achieved genome‐wide significance in the original single‐trait GWAS, and thus do not represent novel cross‐trait associations (Table S5).

**FIGURE 2:**
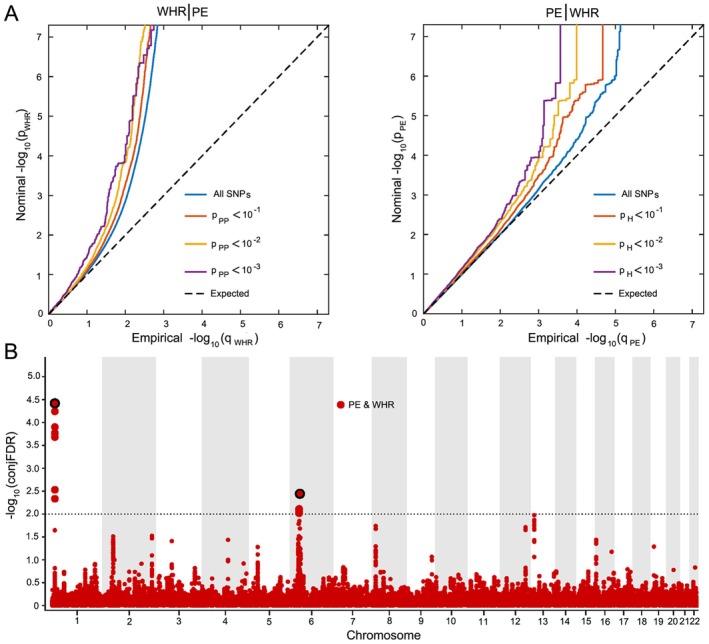
ConjFDR analysis of shared genetic architecture between PE and WHR. (A) Conjunctional false discovery rate (conjFDR) quantile–quantile (QQ) plots for WHR given PE (left) and PE given WHR (right). Enrichment of associations is shown for all SNPs (blue) and for SNPs below different *p*‐value thresholds in the conditional trait (orange, brown, purple). The dashed line represents the null expectation. (B) Manhattan plot of shared risk locus between PE and WHR. The *x*‐axis shows chromosomal positions and the *y*‐axis shows –log10 (conjFDR), reflecting the statistical significance of genomic regions. The dashed horizontal line indicates the significance threshold (−log10 (FDR) = 2).

We next applied the condFDR/conjFDR framework to capture additional variants. Cross‐trait Q–Q plots revealed clear enrichment: conditioning WHR on PE, or PE on WHR, showed increasing deviation from the null line as the conditioning trait threshold became more stringent (*p* < 10^−1^, 10^−2^, 10^−3^) (Figure 2A). This indicates substantial polygenic enrichment, suggesting that strongly associated loci in one trait are more likely to be associated with the other. However, when conditioning on PE at the strictest threshold (*P*_PE_ < 10^−3^), no further deviation was observed compared with more lenient thresholds, likely due to the small number of SNPs passing this cutoff and the limited statistical power of PE, although the existence of PE‐specific loci cannot be excluded. Overall, these results suggest limited cross‐trait enrichment between WHR and PE, although the evidence appears asymmetric and should be interpreted in light of the limited statistical power of the PE GWAS.

We identified a total of 12 genomic loci with *P*_*conjFDR*_ < 0.01, among which one locus showed opposite effect directions and 11 loci exhibited concordant effect directions based on Z‐scores. By further integrating the WHR and PE datasets, we ultimately identified two significant shared lead loci: rs17367504 on chromosome 1 and rs3129882 on chromosome 6 (Figure 2B). Notably, rs198358, rs5010528, rs9461684, and rs9468919 were not significant in either WHR or PE (*p* ≥ 5 × 10^−8^), located on chromosomes 1 and 6, respectively(Figure 2B, Table S6). These findings suggest that although the significance of most SNPs may be influenced by individual traits, three of the signals (rs5010528, rs9461684, and rs9468919) fall within the MHC region, indicating that immune‐related loci may play a role in the shared genetic contribution to both traits. However, given the highly complex LD structure of the MHC region and its potential to dominate enrichment results, we conservatively consider rs17367504 as a robust shared genetic locus, while the other signals on chromosome 6 warrant further investigation.

Importantly, the nearest genes to the chromosome 1 segment are MTHFR‐CLCN6, which has previously been reported as an independent locus associated with PE (PMID: 37248299). Here, we propose that this genomic region may also represent a shared risk locus for both PE and fat distribution.

### 3.4 Tissue‐Level SNP Heritability Enrichment

Using LDSC‐SEG, together with publicly available GWAS data and GTEx tissue annotations, we evaluated tissue‐specific enrichment of SNP heritability for WHR and PE. We identified four tissues with significant enrichment for PE, including cervix and adrenal gland (Table S7). WHR showed the strongest enrichment in adipose‐related tissues such as subcutaneous and visceral fat. Interestingly, WHR was also significantly enriched in breast, cervix, and uterus, suggesting a potential link to female‐specific diseases.

### 3.5 Cell‐Level SNP Heritability Enrichment

We next applied sclinker by integrating GWAS data for WHR, PE, and scRNA‐seq datasets from adipose, uterus, ovary, and whole blood. WHR showed strongest enrichment in classical monocytes (whole blood), mesenchymal stem cells (adipose), endothelial cells (ovary), and uterine endothelium. PE showed strongest enrichment in myofibroblasts (adipose), surface epithelial cells (uterus), uterine fibroblasts (ovary), and memory B cells (whole blood) (Table S8). The cell type with the highest joint enrichment for WHR and PE was uterine pericytes (Table S8).

### 3.6 Functional Enrichment of SNP Heritability

**FIGURE 3:**
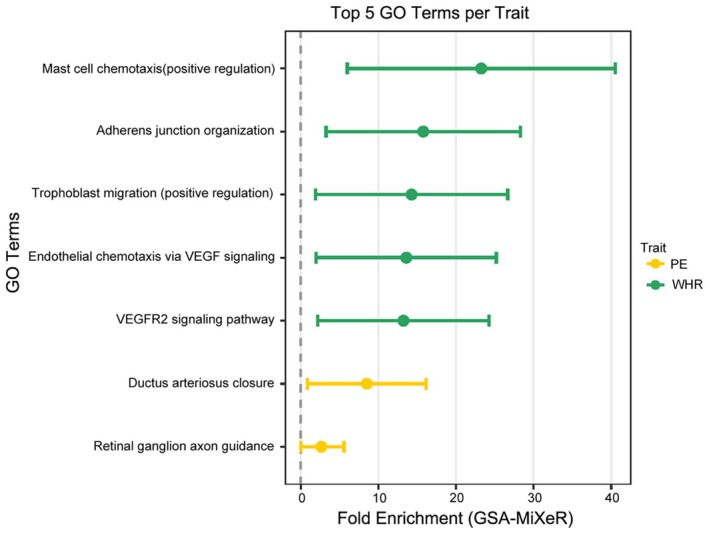
Top enriched GO biological processes for WHR and PE identified by GSA‐MiXeR. The top five Gene Ontology (GO) biological process terms for each trait are shown, with fold enrichment (x‐axis) and 95% confidence intervals. Green dots represent WHR, and yellow dots represent PE. GO terms were simplified for readability by removing prefixes and shortening long descriptions.

To further explore functional enrichment, we applied GSA‐MiXeR to gene sets identified by MAGMA (Bonferroni‐corrected *p* < 0.05). For WHR, we identified 387 enriched gene sets (average gene number = 26, mean fold enrichment = 5.75, RMSE = 1.45; Table S9). The top 20 sets were largely driven by VEGFA. The strongest enrichment signals involved positive regulation of mast cell chemotaxis and regulation of adherens junction organization (Figure 3, Table S10). Notably, exclusion of VEGFA dramatically reduced fold enrichment from an average of 16.05 (RMSE = 3.91) to 2.03 (RMSE = 0.75), underscoring the pivotal role of VEGFA.

For PE, only two gene sets passed Bonferroni correction (average gene number = 5, mean fold enrichment = 5.59, RMSE = 2.71; Table S11): ductus arteriosus closure (driven mainly by FOXF1) and regulation of retinal ganglion cell axon guidance (driven by VEGFA) (Table S9, Figure 3). Removal of the leading gene reduced fold enrichment for ductus arteriosus closure from 8.5 (RMSE = 3.91) to 1.5 (RMSE = 1.39), while enrichment for retinal ganglion cell axon guidance dropped from 8.1 to 0.6 (RMSE = 0.58). These results suggest that VEGFA may act as a central driver in the pathogenesis of PE.

Taken together, these findings implicate VEGFA as a potential core factor linking WHR and PE, likely through multiple biological processes.

### 3.7 Identification of Shared Genes

From the functional enrichment analyses, we noted that both WHR and PE shared gene sets involving *VEGFA*. To further identify potential shared genes not captured by GWAS alone, we performed TWAS by integrating GWAS summary data with eQTL meta‐analysis from GTEx. After Bonferroni correction, two genes were jointly associated with WHR and PE: *TCF19* (enriched in blood) and *CYP21A2* (enriched in ovary) (Table S12). The relatively small number of shared TWAS signals should be interpreted cautiously. This may reflect limited statistical power, stringent Bonferroni correction, tissue specificity of available expression reference panels, and the limited sample size of the PE GWAS rather than an absence of transcriptome‐mediated shared mechanisms.

### 3.8 Causal Inference of WHR and PE

We next conducted bidirectional MR to investigate causality. When WHR was considered as the exposure and PE as the outcome, 244 instrumental variables (IVs) were selected at the threshold *p* < 5 × 10^−8^. All IVs had F‐statistics >10 (mean = 87.17), indicating strong instrument strength, and passed Steiger filtering (Table S13). MR results supported a causal effect of WHR on PE (IVW: BETA = 0.717(SE = 0.05), *p* = 2.7 × 10^−4^). Sensitivity analyses detected heterogeneity but no evidence of horizontal pleiotropy, and therefore random‐effects IVW was taken as the primary estimate (Table S14).

In the reverse analysis, no evidence was found for a causal effect of PE on WHR (Wald ratio: BETA = −0.005 (SE = 0.028), *p* = 0.86). Collectively, these results suggest that the causal relationship between WHR and PE might be unidirectional, with WHR exerting a potential effect on PE but not vice versa.

## 4 Discussion

In this study, we comprehensively dissected the genetic relationship between BMI‐adjusted WHR, representing a more independent measure of fat distribution, and PE. We identified a modest number of shared genetic variants, whereas genome‐wide genetic correlation estimated by LDSC was not significant. MiXeR suggested limited but detectable polygenic overlap between WHR and PE, indicating that the shared genetic architecture is likely small in magnitude rather than broadly distributed across the genome. The most notable local signal was observed on chromosome 14 (22760701–23 985 936). Mendelian randomization further supported a causal relationship between WHR and PE. Through enrichment analyses across tissues, cell types, and biological pathways, we identified overlapping pathological contexts, highlighting VEGFA as a potential central mediator linking WHR to PE. These findings provide novel insights into the shared genetic and pathological mechanisms underlying female fat distribution and PE.

Previous cohort and GWAS studies have reported significant associations of both BMI and WHR with PE [8, 34], though the relative sensitivity of the two traits remains debated. Some studies suggest WHR is less predictive than BMI, while others report the opposite. Our analyses suggest that WHR and PE may share limited genetic components and that genetically predicted WHR may have a potential causal contribution to PE risk; however, BMI appears to show stronger sensitivity to PE [35]. These discrepancies may reflect the fact that BMI and WHR capture related but biologically distinct aspects of adiposity. BMI primarily reflects overall body mass and general adiposity, whereas WHR captures regional fat distribution, particularly central or visceral adiposity, independent of total body size. In pregnancy, BMI may better reflect global metabolic load, whereas WHR may be more closely related to abdominal fat accumulation, insulin resistance, adipokine imbalance, and vascular dysfunction. Therefore, BMI and WHR may predict PE through overlapping but non‐identical pathways. This distinction may also explain why BMI‐related genetic studies often show stronger associations with PE, whereas WHR‐related analyses may reveal more specific mechanisms related to fat distribution and vascular–metabolic regulation.

From a clinical perspective, WHR should be interpreted as a simple anthropometric proxy rather than a direct measure of adipose tissue biology. Adipose tissue is functionally heterogeneous. Metabolically healthy adipose tissue may preserve endocrine, regenerative, and stem cell–supporting properties, whereas dysfunctional or inflamed adipose tissue is characterized by altered adipokine secretion, immune‐cell infiltration, insulin resistance, and increased cardiometabolic risk [49, 50]. Therefore, the genetic association between WHR and PE may reflect not only the amount or distribution of adipose tissue, but also differences in adipose tissue quality, inflammatory status, and metabolic function. This distinction is clinically relevant because PE itself is a heterogeneous syndrome involving variable contributions from placental dysfunction, maternal vascular susceptibility, metabolic disturbance, immune activation, and endothelial injury. Thus, WHR‐related genetic risk should be viewed as one component of a broader clinical risk framework rather than as a standalone determinant of PE.

Notably, LDSC and MiXeR produced partially discordant results. This likely reflects differences in statistical power: given the low heritability of PE, LDSC had limited sensitivity for detecting rg, whereas MiXeR, which models the entire distribution of effect sizes, captured a subtle positive correlation. This suggests that WHR and PE share variants with heterogeneous effect sizes and directions.

Local genetic correlation analysis identified chromosome 14q11.2 as the most relevant locus, containing HNRNPC, DAD1, and MMP14. MMP14 is implicated in adipose and visceral fat remodeling during obesity [36, 37], while HNRNPC and DAD1 have been linked to endometriosis and apoptosis‐associated embryonic lethality [38, 39]. Among shared loci, rs17367504 in MTHFR emerged as particularly noteworthy. The minor allele (G) of rs17367504 has previously been associated with reduced PE risk [40], consistent with our findings. A potential mechanistic link between adipose distribution and endothelial dysfunction may involve visceral adipose tissue–driven inflammation and metabolic stress. Central adiposity is associated with increased secretion of pro‐inflammatory cytokines, altered adipokine profiles, insulin resistance, oxidative stress, and impaired nitric oxide bioavailability, all of which can compromise endothelial homeostasis. Because systemic endothelial dysfunction is a central feature of PE, genetically influenced fat distribution may increase PE susceptibility by amplifying maternal vascular inflammation and endothelial injury. This interpretation is also consistent with our enrichment results implicating vascular‐related tissues, uterine pericytes, and VEGFA‐related pathways [41, 42]. Future work should assess the functional impact of rs17367504 using functional genomics, metabolomics, and animal models to clarify its causal role.

Our enrichment results also suggest that WHR may be more strongly linked to female‐specific pathologies. Cervix emerged as a shared enriched tissue, while pericytes in the uterus represented the most enriched shared cell type. Uterine pericytes are multifunctional vascular mural cells with stem‐like features that regulate blood flow and vascular permeability [43]. At the pathway level, enrichment of ductus arteriosus closure and regulation of retinal ganglion cell axon guidance highlights the potential role of vascular–neuronal crosstalk, with VEGFA emerging as a central driver in both contexts [44]. These findings suggest that shared developmental signaling pathways across vascular and neuronal systems may underlie divergent disease susceptibilities.

At the gene level, we identified TCF19 in blood and CYP21A2 in ovary as novel shared candidates between WHR and PE. TCF19 encodes a transcription factor involved in cell cycle regulation and immune responses [45, 46] and may influence shared susceptibility through immune‐mediated pathways [47]. CYP21A2 encodes a key enzyme in adrenal steroid biosynthesis [48], with aberrant expression implicated in ovarian disorders such as polycystic ovary syndrome. Given the importance of steroid hormones in adipose distribution and blood pressure regulation, CYP21A2 may represent another mechanistic link between WHR and PE. Future experimental studies are needed to validate the roles of TCF19 and CYP21A2 in fat distribution and pregnancy‐related vascular function.

Compared with previous work, our study has several strengths. First, we systematically assessed both genome‐wide and local shared genetic architecture between fat distribution and PE, providing a novel perspective on their relationship. Second, we applied a comprehensive analytical framework integrating multiple complementary methods. Finally, our results highlight the potential involvement of vascular–neuronal networks and identify CYP21A2 and TCF19 as novel shared risk genes, offering new mechanistic hypotheses for future investigation.

Several limitations should be acknowledged. First, our study was restricted to individuals of European ancestry, which may limit generalizability, particularly to populations with higher PE incidence, such as African and Hispanic women. Second, the lack of individual‐level data precluded nonlinear or stratified analyses by age, WHR distribution, lifestyle factors, PE subtype, disease severity, family history, diabetes, cardiovascular disease, hypertension, gestational diabetes, medication use, or other metabolic and hereditary conditions. Because PE is a polyetiological disease with heterogeneous clinical trajectories, the observed genetic overlap with WHR may not apply equally to all PE subtypes. Third, there was an age mismatch between the WHR and PE GWAS datasets. The WHR GWAS was mainly based on women aged 40–69 years, whereas PE occurs in women of reproductive age and the PE dataset included women aged 10–50 years. Although germline variants are fixed at conception, WHRadjBMI measured in middle‐aged women may not fully reflect fat distribution during pregnancy, which could attenuate genetic correlation estimates and limit pregnancy‐specific interpretation. In addition, WHRadjBMI cannot distinguish metabolically healthy adipose tissue from dysfunctional, inflamed, or insulin‐resistant adipose tissue [49, 50]. Therefore, our findings should be interpreted as reflecting genetic liability to fat distribution rather than direct evidence that all adipose tissue contributes uniformly to PE risk. Finally, despite using the most updated PE GWAS, statistical power remains limited, which may have contributed to false‐negative findings in LDSC, local genetic correlation, TWAS, and enrichment analyses. Although sample overlap was estimated to be ~1%, we minimized its impact by triangulating results across multiple complementary approaches.

## 5 Conclusion

These findings provide genetic evidence for a limited but biologically informative relationship between fat distribution and PE. Although the overall shared genetic correlation appears small, the implicated vascular, immune, and adipose‐related pathways may offer hypotheses for future studies on pregnancy complications, risk prediction, and prevention strategies.

## Author Contributions

Man Wang: visualization, writing – original draft, software, writing – review and editing. Li Huang: software. Fengmei Yang: methodology, funding acquisition. Lihua Wu: methodology, writing – review and editing. Danfeng Zhang: writing – original draft, methodology. Bo Gao: conceptualization, writing – review and editing, project administration, supervision.

## Funding

Shiyan Municipal Bureau of Science and Technology (25Y062).

## Conflicts of Interest

The authors declare no conflicts of interest.

## Supporting information


**Table S1:** GWAS datasets description. PE: pre‐eclampsia; WHR adj BMI: WHR adjusted for BMI.

**Table S2:** Polygenicities for WHR and PE were estimated using univariate MiXeR. pi = polygenicity estimate; pi_se = standard error of the polygenicity estimate; sig2_beta = discoverability estimate; sig2_beta_se = standard error of the discoverability estimate; h2 = heritability estimate; h2_se = standard error of the heritability estimate; nc_p9 = the number of influential variants specific to each phenotype; nc_p9_se = standard error of nc_p9; AIC (Akaike Information Criterion) and BIC (Bayesian Information Criterion) values are estimates of the model fit.

**Table S3:** Results of Local Genetic Correlation Analysis between WHR and PE using LAVA. ρ: Local genetic correlation estimates between WHR and PE for each genomic region; ρ.lower and ρ.upper: The lower and upper bounds of the 95% confidence interval for the genetic correlation estimate; *r* ^2^: Proportion of shared variance explained by the genetic correlation between WHR and PE; *r* ^2^.lower and *r* ^2^.upper: The lower and upper bounds of the 95% confidence interval for the proportion of shared variance.P: P testing the significance of the genetic correlation; P FDR: *p*‐value adjusted for multiple testing using the FDR correction; CHR: Chromosome where the genetic region is located; START POSITION and STOP POSITION: Genomic coordinates (in base pairs) indicating the boundaries of each analyzed region on the specified chromosome. Other columns have been clarified in previous tables.

**Table S4:** Results of Local Genetic Correlation Analysis between WHR and PE using ρ‐HESS. NSNP: Number of SNPs (Single Nucleotide Polymorphisms) included in the analysis for the given region; LOCAL rg: Estimate of the local genetic correlation between WHR and PE for the specific genomic region; VAR: Variance of the local genetic correlation estimate; SE: Standard error of the local genetic correlation estimate. Other columns have been clarified in previous tables.

**Table S5:** Significant Loci identified through MTAG and CPASSOC analysis. MTAG_P_PE: P from MTAG analysis for PE; MTAG_P_WHR: P from MTAG analysis for WHR; CPASSOC_P: P from CPASSOC analysis, indicating the combined association significance of the SNP with PE and WHR in a joint analysis. Other columns have been clarified in previous tables.

**Table S6:** Significant Loci Identified through conjFDR Analysis for PE and WHR. BP: Base‐pair position of the variant on the chromosome (in reference to GRCh37/hg19 genome build); Pconj: conjunctional false discovery rate (conjFDR) P indicating the shared genetic association between PE and WHR; Leading SNP: It is the leading SNP (1 = yes, 0 = no); P_PE: P for PE derived after conjFDR analysis, reflecting the association significance of the SNP with PE in the shared genetic context of PE and WHR.; P_WHR: P for WHR derived after conjFDR analysis, reflecting the association significance of the SNP with WHR in the shared genetic context of PE and WHR; Nearest gene: The nearest gene to the identified variant, providing a potential functional annotation. Other columns have been clarified in previous tables.

**Table S7:** The SNP list of tissue‐level enrichment. Coefficient: regression coefficient; Other columns have been clarified in previous tables.

**Table S8:** The significant results of SNP heritability enrichment analysis of WHR and PE at cell level. Prop_SN: Proportion of SNP heritability attributable to the given cell type or tissue; Prop_h2: Proportion of total heritability explained by the given cell type or tissue; Prop_h2_SE: Standard error of the heritability proportion estimate; Enrichment: Enrichment score indicating the relative SNP heritability contribution of the cell type or tissue; Enrichment_SE: Standard error of the enrichment score; Enrichment_P: P for the enrichment score; cellType: The specific cell type within the analyzed tissue. Other columns have been clarified in previous tables.

**Table S9:** The results for WHR and PE: gene‐sets implicated by GSA‐MIXeR analysis (AIC > 0). GO: name of the gene‐set from GO or SynGO database; NGENES: number of genes in the gene‐set, after filtering on genes used in GSA‐MiXeR analysis; enrich: MiXeR fold‐enrichment of heritability; se_enrich: standard error of enrich estimate; h2_frac: gene‐set's fraction of heritability over heritability of all protein‐coding genes; se_h2_frac: standard error of h2_frac; h2_base_frac: same as h2_frac; se_h2_base_frac: standard error of h2_base_frac; excl_GENE: it reports which gene within gene set leads to lower enrichment of the remaining genes after excluding excl_GENE; excl_enrich: enrichment of all remaining genes in the gene‐set after exclusion of the excl_GENE; se_excl_enrich: standard error of excl_GENE; MIXER_AIC: the value of Akaike information criterion, computed as 2*loglike_diff: 2*loglike_df; positive MIXER_AIC indicates that full MiXeR model (with gene‐specific effect size variance) has a better fit than the baseline model in the genomic region defined by the GO column; loglike_diff: improvement in the log‐likelihood in the genomic region defined by the GO column; loglike_df: number of parameters fitted for the region defined by the GO column; GENE_LIST: this gives the list of genes included in the gene set.

**Table S10:** GSA‐MIXeR gene‐level heritability estimates for WHR. h2‐ an estimate of gene's heritability from the full GSA‐MiXeR model; se_h2: standard error of h2; Other columns have been clarified in previous tables.

**Table S11:** GSA‐MIXeR gene‐level heritability estimates for PE. The columns are as follows: GENE: gene name; h2: an estimate of gene's heritability from the full GSA‐MiXeR model; se_h2: standard error of h2; Other columns have been clarified in previous tables.

**Table S12:** Significant TWAS analysis results.

**Table S13:** Results of MR analyses between WHR and PE. No. SNP: The number of genetic variants used as instrumental variables in the MR analysis; WM: Weighted median; IVW: Inverse variance weighted; Mean F stasistic: The average F statistic of all SNPs involved in MR analysis. Other columns have been clarified in previous tables.

**Table S14:** Sensitivity analysis of MR analyses. Exposure: The exposure variable; Outcome: The outcome variable; Method: Mendelian Randomization method used for analysis; Q: Cochran's Q statistic, assessing heterogeneity among SNPs; Q_df: Degrees of freedom for the Q statistic; Q_P: P associated with the Q statistic, indicating the presence of heterogeneity.

## Data Availability

If derived from public domain information.
