# Supplementary material for: Genetic Correlation and Causal Inference Between Female Fat Distribution and Preeclampsia: An Integrative Genomic Study
Source: FASEB J. 2026 Jun 23;40(12):e72074. doi: 10.1096/fj.202601888R (PMC13288445; doi:10.1096/fj.202601888R)
Supplement: Supplementary file 1 — Table S1: GWAS datasets description. PE: pre‐eclampsia; WHR adj BMI: WHR adjusted for BMI. [file FSB2-40-e72074-s011.docx]

| **Supplementary Table S1** | |  |  |  |
| --- | --- | --- | --- | --- |
| ***GWAS datasets description.*** *PE: pre-eclampsia; WHR adj BMI: WHR adjusted for BMI* | | | | |
|  | **Case definition** | **Control definition** | **Cases number** | **Control number** |
| **PE** | ICD-10 O14; ICD-9 624[4-5]; ICD-8 6370[349] | ICD-10 — O10-O16; ICD-9 — 642; ICD-8 — 637 Women not classified under these definitions were considered as controls. | 8,185 | 234,147 |
| **WHR adj BMI** | WHR adjusted for BMI was derived as the residuals from a linear regression of WHR on BMI. | NA | 694,649 | NA |
