# Supplementary material for: Genetic Correlation and Causal Inference Between Female Fat Distribution and Preeclampsia: An Integrative Genomic Study
Source: FASEB J. 2026 Jun 23;40(12):e72074. doi: 10.1096/fj.202601888R (PMC13288445; doi:10.1096/fj.202601888R)
Supplement: Supplementary file 2 — Table S2: Polygenicities for WHR and PE were estimated using univariate MiXeR. pi = polygenicity estimate; pi_se = standard error of the polygenicity estimate; sig2_beta = discoverability estimate; sig2_beta_se = standard error of the discoverability estimate; h2 = heritability estimate; h2_se = standard error of the heritability estimate; nc_p9 = the number of influential variants specific to each phenotype; nc_p9_se = standard error of nc_p9; AIC (Akaike Information Criterion) and BIC (Bayesian Information Criterion) values are estimates of the model fit. [file FSB2-40-e72074-s004.docx]

| Supplementary Table S2 | | |  |  |  |  |  |  |  |  |  |  |
| --- | --- | --- | --- | --- | --- | --- | --- | --- | --- | --- | --- | --- |
| ***Polygenicities for WHR and PE were estimated using univariate MiXeR.*** *pi = polygenicity estimate; pi_se = standard error of the polygenicity estimate; sig2_beta = discoverability estimate; sig2_beta_se = standard error of the discoverability estimate; h2 = heritability estimate; h2_se = standard error of the heritability estimate; nc_p9 = the number of influential variants specific to each phenotype; nc_p9_se = standard error of nc_p9; AIC (Akaike Information Criterion) and BIC (Bayesian Information Criterion) values are estimates of the model fit.* | | | | | | | | | | | | |
| **Trait** | **Trait polygenicity** | | **Trait discoverability** | | | | **Trait heritability** | | **Number of trait-influencing variants (at 90% heritability)** | | **Model selection** | |
|  |  |  |  |  |  |  |  |  |  |  |  |  |
| **Trait** | **pi** | **pi(SD)** | **sig2_beta** | **sig2_beta(SD)** | **sig2_zero** | **sig2_zero(SD)** | **h2** | **h2(SD)** | **nc_p9** | **nc_p9(SD)** | **AIC** | **BIC** |
| WHR | 1.94E-04 | 5.77E-05 | 1.88E-03 | 8.54E-05 | 1.29E+00 | 7.60E-03 | 1.89E+00 | 2.63E-02 | 1.07E+03 | 1.31E+02 | 1.56E+01 | 6.55E+00 |
| PE | 3.34E-04 | 4.10E-05 | 1.61E-04 | 2.13E-05 | 1.07E+00 | 3.29E-03 | 1.13E-01 | 6.03E-03 | 6.19E+02 | 1.84E+02 | 1.16E+01 | 2.56E+00 |
