# Supplementary material for: Genetic Correlation and Causal Inference Between Female Fat Distribution and Preeclampsia: An Integrative Genomic Study
Source: FASEB J. 2026 Jun 23;40(12):e72074. doi: 10.1096/fj.202601888R (PMC13288445; doi:10.1096/fj.202601888R)
Supplement: Supplementary file 3 — Table S3: Results of Local Genetic Correlation Analysis between WHR and PE using LAVA. ρ: Local genetic correlation estimates between WHR and PE for each genomic region; ρ.lower and ρ.upper: The lower and upper bounds of the 95% confidence interval for the genetic correlation estimate; r 2: Proportion of shared variance explained by the genetic correlation between WHR and PE; r 2.lower and r 2.upper: The lower and upper bounds of the 95% confidence interval for the proportion of shared variance.P: P testing the significance of the genetic correlation; P FDR: p‐value adjusted for multiple testing using the FDR correction; CHR: Chromosome where the genetic region is located; START POSITION and STOP POSITION: Genomic coordinates (in base pairs) indicating the boundaries of each analyzed region on the specified chromosome. Other columns have been clarified in previous tables. [file FSB2-40-e72074-s007.docx]

| **Supplementary Table S3** | | |  |  |  |  |  |  |  |  |  |  |
| --- | --- | --- | --- | --- | --- | --- | --- | --- | --- | --- | --- | --- |
| ***Results of Local Genetic Correlation Analysis between WHR and PE using LAVA.*** *ρ: Local genetic correlation estimates between WHR and PE for each genomic region; ρ.lower and ρ.upper: The lower and upper bounds of the 95% confidence interval for the genetic correlation estimate; r²: Proportion of shared variance explained by the genetic correlation between WHR and PE; r².lower and r².upper: The lower and upper bounds of the 95% confidence interval for the proportion of shared variance.P: P testing the significance of the genetic correlation; P FDR: P-value adjusted for multiple testing using the FDR correction; CHR: Chromosome where the genetic region is located; START POSITION and STOP POSITION: Genomic coordinates (in base pairs) indicating the boundaries of each analyzed region on the specified chromosome. Other columns have been clarified in previous tables.* | | | | | | | | | | | | |
| **Trait1** | **Trait2** | **ρ** | **ρ.lower** | **ρ.upper** | **r2** | **r2.lower** | **r2.upper** | **P** | **P FDR** | **CHR** | **START POSITION** | **STOP POSITION** |
| PE | WHR | 0.267252 | -0.12987 | 0.68293 | 0.0714236 | 0 | 0.46831 | 0.181321 | 0.457544 | 1 | 169521552 | 170193207 |
| PE | WHR | -0.06408 | -0.5035 | 0.34947 | 0.0041058 | 0 | 0.27485 | 0.750053 | 0.840969 | 1 | 204092538 | 205009623 |
| PE | WHR | 0.166652 | -0.16052 | 0.50112 | 0.0277728 | 0 | 0.25113 | 0.301852 | 0.587817 | 1 | 205009624 | 205917548 |
| PE | WHR | 0.068223 | -0.30809 | 0.46174 | 0.0046544 | 0 | 0.22683 | 0.712092 | 0.823356 | 1 | 205917549 | 208162951 |
| PE | WHR | 0.016405 | -0.28461 | 0.32088 | 0.0002691 | 0 | 0.1246 | 0.913804 | 0.939187 | 3 | 24270190 | 25516193 |
| PE | WHR | -0.07864 | -0.49579 | 0.33404 | 0.0061842 | 0 | 0.2661 | 0.687067 | 0.820048 | 3 | 36840137 | 38729767 |
| PE | WHR | 0.186005 | -0.20133 | 0.61183 | 0.0345977 | 0 | 0.37637 | 0.33692 | 0.593621 | 3 | 42301658 | 44141693 |
| PE | WHR | 0.062976 | -0.20155 | 0.32834 | 0.003966 | 0 | 0.1104 | 0.628966 | 0.799989 | 3 | 64662374 | 65326751 |
| PE | WHR | 0.555517 | 0.15852 | 1 | 0.3086 | 0.02738 | 1 | 0.010175 | 0.122674 | 3 | 155928191 | 156795410 |
| PE | WHR | 0.194217 | -0.1198 | 0.51998 | 0.0377203 | 0 | 0.27038 | 0.222247 | 0.498997 | 5 | 130573818 | 132554690 |
| PE | WHR | -0.21057 | -0.49665 | 0.08351 | 0.0443398 | 0 | 0.24666 | 0.154504 | 0.457544 | 6 | 29833844 | 30070717 |
| PE | WHR | -0.17739 | -0.56077 | 0.19452 | 0.0314688 | 0 | 0.31482 | 0.333684 | 0.593621 | 6 | 30070718 | 30715006 |
| PE | WHR | 0.055048 | -0.17842 | 0.28435 | 0.0030303 | 0 | 0.08273 | 0.631346 | 0.799989 | 6 | 30715007 | 31106493 |
| PE | WHR | 0.125976 | -0.16435 | 0.40896 | 0.0158699 | 0 | 0.16732 | 0.379496 | 0.638243 | 6 | 31106494 | 31250556 |
| PE | WHR | 0.256486 | -0.11424 | 0.64426 | 0.0657853 | 0 | 0.41507 | 0.171437 | 0.457544 | 6 | 31250557 | 31320268 |
| PE | WHR | 0.105689 | -0.26853 | 0.47625 | 0.0111701 | 0 | 0.23081 | 0.557095 | 0.796603 | 6 | 31320269 | 31427209 |
| PE | WHR | 0.006303 | -0.20142 | 0.21492 | 3.97E-05 | 0 | 0.05686 | 0.951326 | 0.951326 | 6 | 31427210 | 32208901 |
| PE | WHR | 0.231853 | -0.08549 | 0.55663 | 0.0537559 | 0 | 0.30984 | 0.153413 | 0.457544 | 6 | 32208902 | 32454577 |
| PE | WHR | 0.367485 | 0.06139 | 0.6666 | 0.135045 | 0.00465 | 0.44436 | 0.025126 | 0.154944 | 6 | 32586785 | 32629239 |
| PE | WHR | 0.290381 | -0.03196 | 0.60233 | 0.0843211 | 0 | 0.36281 | 0.081033 | 0.333137 | 6 | 32629240 | 32682213 |
| PE | WHR | -0.06411 | -0.34916 | 0.21593 | 0.0041104 | 0 | 0.12624 | 0.64864 | 0.799989 | 6 | 32682214 | 32897998 |
| PE | WHR | 0.383718 | 0.10038 | 0.68945 | 0.14724 | 0.01008 | 0.47534 | 0.008419 | 0.122674 | 8 | 22895019 | 23788494 |
| PE | WHR | -0.03694 | -0.4551 | 0.3739 | 0.0013646 | 0 | 0.23715 | 0.852196 | 0.900893 | 9 | 93441051 | 94175374 |
| PE | WHR | 0.248386 | -0.16278 | 0.70929 | 0.0616958 | 0 | 0.50309 | 0.229269 | 0.498997 | 9 | 112865521 | 113646149 |
| PE | WHR | -0.21363 | -0.49145 | 0.06417 | 0.0456364 | 0 | 0.24152 | 0.120328 | 0.445214 | 10 | 104206838 | 106142283 |
| PE | WHR | 0.40498 | 0.15478 | 0.68488 | 0.164008 | 0.02407 | 0.46906 | 0.00284 | 0.105067 | 11 | 68887345 | 69713996 |
| PE | WHR | 0.106137 | -0.30035 | 0.53167 | 0.011265 | 0 | 0.29098 | 0.592401 | 0.799989 | 12 | 111592382 | 113947983 |
| PE | WHR | -0.08247 | -0.37431 | 0.20192 | 0.0068018 | 0 | 0.14205 | 0.559775 | 0.796603 | 15 | 39238841 | 40604780 |
| PE | WHR | -0.21781 | -0.56713 | 0.10353 | 0.0474427 | 0 | 0.32164 | 0.185491 | 0.457544 | 15 | 40604781 | 42332685 |
| PE | WHR | -0.36457 | -0.75443 | -0.00726 | 0.132913 | 0.00172 | 0.56916 | 0.043475 | 0.229794 | 15 | 74458114 | 76401952 |
| PE | WHR | 0.389253 | 0.08173 | 0.75569 | 0.151518 | 0.0076 | 0.57107 | 0.016492 | 0.122674 | 16 | 3379997 | 4816145 |
| PE | WHR | -0.12471 | -0.49503 | 0.23456 | 0.0155537 | 0 | 0.24696 | 0.486574 | 0.782749 | 16 | 66738844 | 68435728 |
| PE | WHR | 0.433865 | 0.09135 | 0.84186 | 0.188239 | 0.00922 | 0.70873 | 0.016578 | 0.122674 | 17 | 45883902 | 47516224 |
| PE | WHR | 0.031774 | -0.25041 | 0.30435 | 0.0010096 | 0 | 0.10373 | 0.816318 | 0.888346 | 19 | 10028841 | 11681978 |
| PE | WHR | -0.29082 | -0.63422 | 0.02563 | 0.084578 | 0 | 0.40224 | 0.073972 | 0.333137 | 19 | 45040933 | 45893307 |
| PE | WHR | 0.119686 | -0.31393 | 0.5384 | 0.0143248 | 0 | 0.30284 | 0.552113 | 0.796603 | 20 | 48212976 | 49236418 |
| PE | WHR | 0.162694 | -0.14297 | 0.48355 | 0.0264694 | 0 | 0.23382 | 0.28343 | 0.582606 | 20 | 51533751 | 52411532 |
