# Supplementary material for: Genetic Correlation and Causal Inference Between Female Fat Distribution and Preeclampsia: An Integrative Genomic Study
Source: FASEB J. 2026 Jun 23;40(12):e72074. doi: 10.1096/fj.202601888R (PMC13288445; doi:10.1096/fj.202601888R)
Supplement: Supplementary file 4 — Table S4: Results of Local Genetic Correlation Analysis between WHR and PE using ρ‐HESS. NSNP: Number of SNPs (Single Nucleotide Polymorphisms) included in the analysis for the given region; LOCAL rg: Estimate of the local genetic correlation between WHR and PE for the specific genomic region; VAR: Variance of the local genetic correlation estimate; SE: Standard error of the local genetic correlation estimate. Other columns have been clarified in previous tables. [file FSB2-40-e72074-s006.docx]

| **Supplementary Table S4** | | |  |  |  |  |  |  |  |
| --- | --- | --- | --- | --- | --- | --- | --- | --- | --- |
| ***Results of Local Genetic Correlation Analysis between WHR and PE using ρ-HESS.*** *NSNP: Number of SNPs (Single Nucleotide Polymorphisms) included in the analysis for the given region; LOCAL rg: Estimate of the local genetic correlation between WHR and PE for the specific genomic region; VAR: Variance of the local genetic correlation estimate; SE: Standard error of the local genetic correlation estimate. Other columns have been clarified in previous tables.* | | | | | | | | |  |
| **CHR** | **START POSITION** | **STOP POSITION** | **NSNP** | **LOCAL rg** | **VAR** | **SE** | **Z** | ***P*** | ***Padj*** |
| 1 | 10583 | 1892607 | 136 | 1.40E-05 | 1.03E-09 | 3.21E-05 | 0.43693 | 0.66216 | 0.99961 |
| 1 | 1892607 | 3582736 | 787 | -3.62E-05 | 3.89E-09 | 6.24E-05 | -0.58014 | 0.56182 | 0.99961 |
| 1 | 3582736 | 4380811 | 545 | 3.18E-05 | 2.87E-09 | 5.36E-05 | 0.59396 | 0.55254 | 0.99961 |
| 1 | 4380811 | 5913893 | 1848 | -1.91E-06 | 3.22E-09 | 5.68E-05 | -0.0336 | 0.9732 | 0.99961 |
| 1 | 5913893 | 7247335 | 730 | 2.51E-06 | 3.38E-09 | 5.81E-05 | 0.043152 | 0.96558 | 0.99961 |
| 1 | 7247335 | 9365199 | 1254 | 2.33E-05 | 4.17E-09 | 6.46E-05 | 0.36025 | 0.71866 | 0.99961 |
| 1 | 9365199 | 10806984 | 538 | 3.03E-06 | 2.85E-09 | 5.34E-05 | 0.056642 | 0.95483 | 0.99961 |
| 1 | 10806984 | 11777841 | 393 | -7.68E-06 | 2.41E-09 | 4.91E-05 | -0.15647 | 0.87566 | 0.99961 |
| 1 | 11777841 | 12779466 | 442 | 5.72E-05 | 3.21E-09 | 5.66E-05 | 1.0092 | 0.3129 | 0.99961 |
| 1 | 12779466 | 14891511 | 1186 | -1.08E-06 | 3.25E-09 | 5.70E-05 | -0.01892 | 0.98491 | 0.99961 |
| 1 | 14891511 | 16897730 | 1112 | 6.88E-06 | 3.42E-09 | 5.85E-05 | 0.11766 | 0.90633 | 0.99961 |
| 1 | 16897730 | 18662899 | 1111 | -1.29E-06 | 3.67E-09 | 6.06E-05 | -0.02122 | 0.98307 | 0.99961 |
| 1 | 18662899 | 20469149 | 1196 | 3.39E-06 | 3.21E-09 | 5.66E-05 | 0.059789 | 0.95232 | 0.99961 |
| 1 | 20469149 | 21736588 | 797 | 2.63E-05 | 3.82E-09 | 6.18E-05 | 0.42614 | 0.67 | 0.99961 |
| 1 | 21736588 | 23086883 | 925 | -4.00E-05 | 3.92E-09 | 6.26E-05 | -0.63863 | 0.52306 | 0.99961 |
| 1 | 23086883 | 23920590 | 346 | 6.47E-05 | 2.89E-09 | 5.38E-05 | 1.2037 | 0.22872 | 0.99961 |
| 1 | 23920590 | 25516845 | 892 | 9.31E-06 | 3.34E-09 | 5.78E-05 | 0.16107 | 0.87203 | 0.99961 |
| 1 | 25516845 | 27401867 | 650 | 1.66E-05 | 2.68E-09 | 5.18E-05 | 0.31968 | 0.74921 | 0.99961 |
| 1 | 27401867 | 30161881 | 944 | 2.99E-05 | 3.64E-09 | 6.03E-05 | 0.49541 | 0.62031 | 0.99961 |
| 1 | 30161881 | 32438685 | 1861 | 2.51E-05 | 3.24E-09 | 5.69E-05 | 0.44059 | 0.65951 | 0.99961 |
| 1 | 32438685 | 34799758 | 1151 | 3.65E-06 | 3.27E-09 | 5.72E-05 | 0.063845 | 0.94909 | 0.99961 |
| 1 | 34799758 | 37549183 | 970 | -3.72E-05 | 3.63E-09 | 6.03E-05 | -0.61668 | 0.53744 | 0.99961 |
| 1 | 37549183 | 38731847 | 611 | 1.45E-05 | 2.53E-09 | 5.03E-05 | 0.28908 | 0.77252 | 0.99961 |
| 1 | 38731847 | 40200567 | 737 | -1.54E-05 | 3.34E-09 | 5.78E-05 | -0.26619 | 0.7901 | 0.99961 |
| 1 | 40200567 | 41975327 | 1003 | 1.98E-05 | 3.23E-09 | 5.68E-05 | 0.34875 | 0.72728 | 0.99961 |
| 1 | 41975327 | 43758457 | 1069 | -2.09E-05 | 3.97E-09 | 6.30E-05 | -0.33199 | 0.7399 | 0.99961 |
| 1 | 43758457 | 44969183 | 616 | -2.04E-05 | 2.53E-09 | 5.03E-05 | -0.40429 | 0.686 | 0.99961 |
| 1 | 44969183 | 46899501 | 798 | -1.15E-05 | 2.92E-09 | 5.40E-05 | -0.21206 | 0.83206 | 0.99961 |
| 1 | 46899501 | 48002576 | 511 | 1.97E-05 | 1.81E-09 | 4.25E-05 | 0.46374 | 0.64284 | 0.99961 |
| 1 | 48002576 | 48978188 | 559 | -3.11E-05 | 2.62E-09 | 5.12E-05 | -0.60864 | 0.54277 | 0.99961 |
| 1 | 48978188 | 49894177 | 456 | 1.23E-05 | 1.65E-09 | 4.06E-05 | 0.30271 | 0.76211 | 0.99961 |
| 1 | 49894177 | 51713726 | 485 | -4.19E-06 | 1.89E-09 | 4.35E-05 | -0.09642 | 0.92319 | 0.99961 |
| 1 | 51713726 | 54226262 | 966 | -3.67E-06 | 3.38E-09 | 5.81E-05 | -0.06307 | 0.94971 | 0.99961 |
| 1 | 54226262 | 56413117 | 1440 | -9.14E-06 | 3.17E-09 | 5.63E-05 | -0.16218 | 0.87117 | 0.99961 |
| 1 | 56413117 | 57021728 | 518 | 1.38E-06 | 2.24E-09 | 4.74E-05 | 0.0291 | 0.97678 | 0.99961 |
| 1 | 57021728 | 58865399 | 1530 | -2.50E-05 | 3.22E-09 | 5.67E-05 | -0.4411 | 0.65914 | 0.99961 |
| 1 | 58865399 | 59890409 | 758 | 2.33E-05 | 2.92E-09 | 5.40E-05 | 0.43095 | 0.66651 | 0.99961 |
| 1 | 59890409 | 61922365 | 1199 | -1.69E-05 | 3.50E-09 | 5.91E-05 | -0.285 | 0.77565 | 0.99961 |
| 1 | 61922365 | 63455089 | 952 | -5.21E-06 | 3.07E-09 | 5.54E-05 | -0.09414 | 0.925 | 0.99961 |
| 1 | 63455089 | 65041704 | 1319 | -2.07E-05 | 3.33E-09 | 5.77E-05 | -0.35798 | 0.72036 | 0.99961 |
| 1 | 65041704 | 66939404 | 1326 | 4.30E-05 | 4.25E-09 | 6.52E-05 | 0.65988 | 0.50933 | 0.99961 |
| 1 | 66939404 | 68477895 | 1337 | 2.12E-05 | 3.22E-09 | 5.68E-05 | 0.37345 | 0.70881 | 0.99961 |
| 1 | 68477895 | 69687616 | 750 | 3.79E-05 | 2.64E-09 | 5.13E-05 | 0.73738 | 0.46089 | 0.99961 |
| 1 | 69687616 | 71372470 | 1096 | 2.69E-05 | 3.24E-09 | 5.69E-05 | 0.47266 | 0.63645 | 0.99961 |
| 1 | 71372470 | 71684405 | 175 | 1.38E-05 | 8.34E-10 | 2.89E-05 | 0.47839 | 0.63237 | 0.99961 |
| 1 | 71684405 | 74326907 | 1574 | 7.59E-06 | 3.27E-09 | 5.72E-05 | 0.13257 | 0.89453 | 0.99961 |
| 1 | 74326907 | 76728135 | 1661 | -5.42E-05 | 3.32E-09 | 5.77E-05 | -0.94015 | 0.34714 | 0.99961 |
| 1 | 76728135 | 79661198 | 1963 | -8.47E-07 | 3.84E-09 | 6.20E-05 | -0.01368 | 0.98909 | 0.99961 |
| 1 | 79661198 | 81354191 | 1189 | 1.45E-05 | 2.92E-09 | 5.41E-05 | 0.26868 | 0.78818 | 0.99961 |
| 1 | 81354191 | 83991748 | 2159 | 2.59E-05 | 3.26E-09 | 5.71E-05 | 0.45266 | 0.6508 | 0.99961 |
| 1 | 83991748 | 84844495 | 557 | 3.22E-06 | 1.76E-09 | 4.19E-05 | 0.076837 | 0.93875 | 0.99961 |
| 1 | 84844495 | 86454751 | 1267 | 1.02E-05 | 3.69E-09 | 6.07E-05 | 0.16854 | 0.86616 | 0.99961 |
| 1 | 86454751 | 88128631 | 1127 | -2.88E-05 | 3.42E-09 | 5.84E-05 | -0.49303 | 0.62199 | 0.99961 |
| 1 | 88128631 | 90066303 | 1005 | 9.91E-07 | 3.51E-09 | 5.93E-05 | 0.016722 | 0.98666 | 0.99961 |
| 1 | 90066303 | 91886317 | 1037 | -2.95E-05 | 3.36E-09 | 5.79E-05 | -0.50912 | 0.61067 | 0.99961 |
| 1 | 91886317 | 93536459 | 1045 | -1.10E-05 | 3.16E-09 | 5.62E-05 | -0.1962 | 0.84445 | 0.99961 |
| 1 | 93536459 | 94506191 | 521 | -3.22E-05 | 1.82E-09 | 4.26E-05 | -0.75546 | 0.44997 | 0.99961 |
| 1 | 94506191 | 96150893 | 1098 | -4.53E-05 | 3.42E-09 | 5.84E-05 | -0.77511 | 0.43827 | 0.99961 |
| 1 | 96150893 | 97885249 | 1124 | -2.05E-05 | 2.92E-09 | 5.41E-05 | -0.38014 | 0.70384 | 0.99961 |
| 1 | 97885249 | 99800604 | 1272 | 4.26E-05 | 3.51E-09 | 5.92E-05 | 0.71928 | 0.47197 | 0.99961 |
| 1 | 99800604 | 100826405 | 652 | 1.47E-05 | 1.95E-09 | 4.42E-05 | 0.3332 | 0.73899 | 0.99961 |
| 1 | 100826405 | 102041016 | 873 | -4.33E-05 | 3.00E-09 | 5.48E-05 | -0.78925 | 0.42997 | 0.99961 |
| 1 | 102041016 | 102898745 | 677 | 2.01E-05 | 1.70E-09 | 4.12E-05 | 0.48905 | 0.6248 | 0.99961 |
| 1 | 102898745 | 103914211 | 607 | 3.63E-05 | 1.81E-09 | 4.26E-05 | 0.85246 | 0.39396 | 0.99961 |
| 1 | 103914211 | 106087842 | 1247 | 2.57E-05 | 3.31E-09 | 5.76E-05 | 0.44604 | 0.65557 | 0.99961 |
| 1 | 106087842 | 108409665 | 1641 | -9.46E-05 | 3.45E-09 | 5.87E-05 | -1.6099 | 0.10741 | 0.99961 |
| 1 | 108409665 | 110303931 | 1039 | 8.22E-05 | 3.72E-09 | 6.10E-05 | 1.3477 | 0.17775 | 0.99961 |
| 1 | 110303931 | 111741737 | 961 | -1.89E-05 | 3.63E-09 | 6.03E-05 | -0.31369 | 0.75376 | 0.99961 |
| 1 | 111741737 | 113273306 | 1304 | 1.54E-05 | 3.13E-09 | 5.60E-05 | 0.27564 | 0.78283 | 0.99961 |
| 1 | 113273306 | 114873845 | 885 | -3.72E-06 | 3.36E-09 | 5.80E-05 | -0.06415 | 0.94885 | 0.99961 |
| 1 | 114873845 | 115880593 | 589 | -1.32E-05 | 2.25E-09 | 4.75E-05 | -0.27898 | 0.78026 | 0.99961 |
| 1 | 115880593 | 118839067 | 1602 | -1.41E-05 | 3.61E-09 | 6.01E-05 | -0.23428 | 0.81477 | 0.99961 |
| 1 | 118839067 | 144977494 | 1163 | -9.52E-06 | 6.38E-09 | 7.99E-05 | -0.11918 | 0.90514 | 0.99961 |
| 1 | 144977494 | 148361253 | 762 | 1.47E-07 | 2.81E-09 | 5.31E-05 | 0.002774 | 0.99779 | 0.99961 |
| 1 | 148361253 | 151538881 | 539 | -3.07E-06 | 2.60E-09 | 5.10E-05 | -0.06014 | 0.95205 | 0.99961 |
| 1 | 151538881 | 153180829 | 960 | 6.93E-05 | 2.79E-09 | 5.28E-05 | 1.3128 | 0.18926 | 0.99961 |
| 1 | 153180829 | 154770403 | 763 | -3.23E-05 | 3.38E-09 | 5.81E-05 | -0.55564 | 0.57846 | 0.99961 |
| 1 | 154770403 | 156336133 | 463 | 2.62E-05 | 4.40E-09 | 6.63E-05 | 0.39589 | 0.69219 | 0.99961 |
| 1 | 156336133 | 158027412 | 1140 | -3.30E-05 | 3.50E-09 | 5.92E-05 | -0.5569 | 0.57759 | 0.99961 |
| 1 | 158027412 | 159913048 | 1289 | 1.73E-05 | 3.52E-09 | 5.93E-05 | 0.29213 | 0.77019 | 0.99961 |
| 1 | 159913048 | 162346721 | 1582 | 2.82E-07 | 3.37E-09 | 5.81E-05 | 0.004851 | 0.99613 | 0.99961 |
| 1 | 162346721 | 165191702 | 2381 | 7.92E-07 | 3.95E-09 | 6.29E-05 | 0.01259 | 0.98995 | 0.99961 |
| 1 | 165191702 | 166460517 | 990 | 2.70E-05 | 3.67E-09 | 6.06E-05 | 0.44546 | 0.65599 | 0.99961 |
| 1 | 166460517 | 169086324 | 1973 | -1.49E-05 | 3.08E-09 | 5.55E-05 | -0.26821 | 0.78854 | 0.99961 |
| 1 | 169086324 | 170557776 | 1369 | -8.78E-06 | 5.81E-09 | 7.62E-05 | -0.11519 | 0.90829 | 0.99961 |
| 1 | 170557776 | 173097907 | 1653 | 5.35E-05 | 6.70E-09 | 8.19E-05 | 0.654 | 0.51311 | 0.99961 |
| 1 | 173097907 | 175089768 | 722 | 7.95E-06 | 1.81E-09 | 4.26E-05 | 0.1866 | 0.85198 | 0.99961 |
| 1 | 175089768 | 177433381 | 1514 | 5.02E-05 | 3.64E-09 | 6.04E-05 | 0.83169 | 0.40558 | 0.99961 |
| 1 | 177433381 | 178944309 | 907 | -8.29E-05 | 3.14E-09 | 5.60E-05 | -1.4791 | 0.1391 | 0.99961 |
| 1 | 178944309 | 178954470 | 3 | 4.99E-06 | 1.59E-10 | 1.26E-05 | 0.39575 | 0.69229 | 0.99961 |
| 1 | 178954470 | 181144121 | 1468 | -3.72E-05 | 3.01E-09 | 5.49E-05 | -0.67876 | 0.49729 | 0.99961 |
| 1 | 181144121 | 182755356 | 1077 | -3.78E-05 | 3.46E-09 | 5.89E-05 | -0.64243 | 0.52059 | 0.99961 |
| 1 | 182755356 | 184595513 | 1275 | 3.19E-05 | 3.50E-09 | 5.92E-05 | 0.53958 | 0.58949 | 0.99961 |
| 1 | 184595513 | 186810023 | 868 | 3.03E-05 | 2.72E-09 | 5.21E-05 | 0.58133 | 0.56102 | 0.99961 |
| 1 | 186810023 | 188759945 | 1480 | 2.01E-05 | 3.48E-09 | 5.90E-05 | 0.34091 | 0.73317 | 0.99961 |
| 1 | 188759945 | 189904130 | 606 | 2.01E-05 | 1.22E-09 | 3.49E-05 | 0.577 | 0.56394 | 0.99961 |
| 1 | 189904130 | 191868930 | 1491 | -2.25E-06 | 2.84E-09 | 5.33E-05 | -0.04218 | 0.96635 | 0.99961 |
| 1 | 191868930 | 194107442 | 1316 | -2.44E-05 | 2.94E-09 | 5.42E-05 | -0.44921 | 0.65328 | 0.99961 |
| 1 | 194107442 | 196176201 | 1570 | 1.39E-05 | 3.15E-09 | 5.61E-05 | 0.24826 | 0.80393 | 0.99961 |
| 1 | 196176201 | 197311514 | 482 | 2.32E-05 | 1.52E-09 | 3.90E-05 | 0.59441 | 0.55224 | 0.99961 |
| 1 | 197311514 | 199239884 | 1015 | 3.44E-05 | 3.28E-09 | 5.73E-05 | 0.60102 | 0.54783 | 0.99961 |
| 1 | 199239884 | 200137649 | 622 | 3.30E-05 | 2.60E-09 | 5.10E-05 | 0.64664 | 0.51786 | 0.99961 |
| 1 | 200137649 | 201589975 | 1233 | -2.37E-05 | 3.41E-09 | 5.84E-05 | -0.4066 | 0.6843 | 0.99961 |
| 1 | 201589975 | 203334734 | 1025 | -2.33E-05 | 3.66E-09 | 6.05E-05 | -0.38575 | 0.69968 | 0.99961 |
| 1 | 203334734 | 204681068 | 806 | 1.22E-05 | 3.68E-09 | 6.07E-05 | 0.20057 | 0.84103 | 0.99961 |
| 1 | 204681068 | 206073265 | 1037 | 5.84E-05 | 4.38E-09 | 6.62E-05 | 0.88293 | 0.37727 | 0.99961 |
| 1 | 206073265 | 208410364 | 1144 | 8.20E-06 | 4.07E-09 | 6.38E-05 | 0.12863 | 0.89765 | 0.99961 |
| 1 | 208410364 | 210787750 | 1705 | 4.18E-05 | 3.54E-09 | 5.95E-05 | 0.70273 | 0.48222 | 0.99961 |
| 1 | 210787750 | 212842330 | 1257 | 1.36E-05 | 4.08E-09 | 6.39E-05 | 0.21318 | 0.83119 | 0.99961 |
| 1 | 212842330 | 214578266 | 1090 | -6.46E-06 | 3.97E-09 | 6.30E-05 | -0.10258 | 0.9183 | 0.99961 |
| 1 | 214578266 | 216243634 | 1243 | -2.74E-05 | 3.64E-09 | 6.04E-05 | -0.45425 | 0.64965 | 0.99961 |
| 1 | 216243634 | 218705513 | 2275 | -2.07E-05 | 3.02E-09 | 5.50E-05 | -0.37759 | 0.70574 | 0.99961 |
| 1 | 218705513 | 219590571 | 640 | -1.13E-05 | 5.27E-09 | 7.26E-05 | -0.15512 | 0.87673 | 0.99961 |
| 1 | 219590571 | 221858231 | 1495 | -0.00011775 | 1.14E-08 | 0.0001067 | -1.1037 | 0.26974 | 0.99961 |
| 1 | 221858231 | 222230220 | 331 | -1.39E-05 | 1.77E-09 | 4.21E-05 | -0.3302 | 0.74125 | 0.99961 |
| 1 | 222230220 | 224938520 | 914 | 3.08E-05 | 3.55E-09 | 5.96E-05 | 0.51592 | 0.60591 | 0.99961 |
| 1 | 224938520 | 226810860 | 894 | -4.47E-07 | 3.29E-09 | 5.74E-05 | -0.00779 | 0.99379 | 0.99961 |
| 1 | 226810860 | 229156248 | 1389 | 2.63E-05 | 3.73E-09 | 6.11E-05 | 0.4309 | 0.66654 | 0.99961 |
| 1 | 229156248 | 230685255 | 864 | 5.97E-06 | 3.12E-09 | 5.59E-05 | 0.10683 | 0.91493 | 0.99961 |
| 1 | 230685255 | 232090252 | 1034 | 1.66E-05 | 2.98E-09 | 5.46E-05 | 0.30353 | 0.76149 | 0.99961 |
| 1 | 232090252 | 233429284 | 1108 | -4.79E-05 | 3.36E-09 | 5.79E-05 | -0.82612 | 0.40874 | 0.99961 |
| 1 | 233429284 | 234599434 | 1019 | -1.20E-06 | 3.06E-09 | 5.53E-05 | -0.02167 | 0.98271 | 0.99961 |
| 1 | 234599434 | 235819436 | 704 | 2.39E-05 | 3.43E-09 | 5.85E-05 | 0.4079 | 0.68334 | 0.99961 |
| 1 | 235819436 | 237555628 | 1323 | 9.32E-06 | 3.52E-09 | 5.94E-05 | 0.15707 | 0.87519 | 0.99961 |
| 1 | 237555628 | 238567807 | 865 | -2.03E-05 | 3.23E-09 | 5.69E-05 | -0.35713 | 0.72099 | 0.99961 |
| 1 | 238567807 | 239925314 | 1084 | 2.32E-06 | 3.10E-09 | 5.57E-05 | 0.041709 | 0.96673 | 0.99961 |
| 1 | 239925314 | 240564091 | 642 | -1.62E-06 | 2.66E-09 | 5.16E-05 | -0.03139 | 0.97496 | 0.99961 |
| 1 | 240564091 | 241582220 | 919 | -3.17E-06 | 3.20E-09 | 5.66E-05 | -0.05596 | 0.95537 | 0.99961 |
| 1 | 241582220 | 242071602 | 377 | -1.07E-05 | 1.60E-09 | 4.00E-05 | -0.26797 | 0.78872 | 0.99961 |
| 1 | 242071602 | 244109499 | 1093 | -2.12E-05 | 3.11E-09 | 5.58E-05 | -0.37965 | 0.7042 | 0.99961 |
| 1 | 244109499 | 245506746 | 900 | 6.67E-06 | 3.24E-09 | 5.69E-05 | 0.11718 | 0.90672 | 0.99961 |
| 1 | 245506746 | 247344518 | 1329 | 5.56E-05 | 3.18E-09 | 5.64E-05 | 0.98566 | 0.3243 | 0.99961 |
| 1 | 247344518 | 249239466 | 843 | 1.68E-05 | 3.09E-09 | 5.56E-05 | 0.30214 | 0.76254 | 0.99961 |
| 2 | 10133 | 1781022 | 1311 | -3.41E-05 | 3.17E-09 | 5.63E-05 | -0.60523 | 0.54502 | 0.99961 |
| 2 | 1781022 | 3973779 | 1793 | 1.18E-05 | 2.69E-09 | 5.18E-05 | 0.22777 | 0.81982 | 0.99961 |
| 2 | 3973779 | 5708337 | 1386 | -7.24E-06 | 3.00E-09 | 5.48E-05 | -0.13203 | 0.89496 | 0.99961 |
| 2 | 5708337 | 6836254 | 1251 | 1.51E-05 | 3.33E-09 | 5.77E-05 | 0.26156 | 0.79366 | 0.99961 |
| 2 | 6836254 | 8422147 | 1330 | 2.42E-05 | 3.17E-09 | 5.63E-05 | 0.42981 | 0.66733 | 0.99961 |
| 2 | 8422147 | 10298469 | 1310 | -2.77E-05 | 3.89E-09 | 6.24E-05 | -0.44434 | 0.65679 | 0.99961 |
| 2 | 10298469 | 12419150 | 1835 | 2.26E-05 | 3.47E-09 | 5.89E-05 | 0.3842 | 0.70083 | 0.99961 |
| 2 | 12419150 | 14335308 | 1564 | 3.27E-05 | 3.80E-09 | 6.17E-05 | 0.53012 | 0.59603 | 0.99961 |
| 2 | 14335308 | 16329735 | 1731 | -1.10E-05 | 3.59E-09 | 5.99E-05 | -0.18384 | 0.85414 | 0.99961 |
| 2 | 16329735 | 18647423 | 1839 | -2.68E-05 | 2.93E-09 | 5.41E-05 | -0.49573 | 0.62009 | 0.99961 |
| 2 | 18647423 | 19692404 | 789 | 3.55E-05 | 3.29E-09 | 5.74E-05 | 0.61866 | 0.53614 | 0.99961 |
| 2 | 19692404 | 21050490 | 1272 | -6.11E-06 | 2.83E-09 | 5.32E-05 | -0.11497 | 0.90847 | 0.99961 |
| 2 | 21050490 | 23341383 | 1369 | -3.84E-06 | 2.80E-09 | 5.29E-05 | -0.0726 | 0.94212 | 0.99961 |
| 2 | 23341383 | 24686918 | 854 | -2.80E-05 | 3.48E-09 | 5.90E-05 | -0.47462 | 0.63506 | 0.99961 |
| 2 | 24686918 | 26894985 | 1025 | 9.56E-06 | 3.71E-09 | 6.09E-05 | 0.15707 | 0.87519 | 0.99961 |
| 2 | 26894985 | 28598777 | 707 | 3.24E-05 | 2.53E-09 | 5.03E-05 | 0.64454 | 0.51922 | 0.99961 |
| 2 | 28598777 | 29217559 | 460 | 4.78E-05 | 2.00E-09 | 4.47E-05 | 1.0694 | 0.2849 | 0.99961 |
| 2 | 29217559 | 30463711 | 1180 | -2.22E-05 | 2.92E-09 | 5.40E-05 | -0.41004 | 0.68178 | 0.99961 |
| 2 | 30463711 | 31550450 | 1135 | 9.94E-06 | 3.02E-09 | 5.49E-05 | 0.18088 | 0.85646 | 0.99961 |
| 2 | 31550450 | 33363777 | 794 | -3.58E-05 | 3.04E-09 | 5.51E-05 | -0.64866 | 0.51656 | 0.99961 |
| 2 | 33363777 | 35345028 | 1733 | -5.47E-06 | 2.58E-09 | 5.07E-05 | -0.10771 | 0.91423 | 0.99961 |
| 2 | 35345028 | 36122006 | 841 | 2.12E-05 | 2.25E-09 | 4.75E-05 | 0.44711 | 0.6548 | 0.99961 |
| 2 | 36122006 | 38132712 | 1848 | 3.43E-05 | 3.06E-09 | 5.53E-05 | 0.62108 | 0.53455 | 0.99961 |
| 2 | 38132712 | 39031337 | 606 | -1.69E-05 | 3.02E-09 | 5.50E-05 | -0.30689 | 0.75893 | 0.99961 |
| 2 | 39031337 | 40281483 | 612 | -1.37E-05 | 2.71E-09 | 5.21E-05 | -0.2638 | 0.79194 | 0.99961 |
| 2 | 40281483 | 43309590 | 2748 | -1.13E-05 | 3.16E-09 | 5.62E-05 | -0.20137 | 0.84041 | 0.99961 |
| 2 | 43309590 | 44313803 | 825 | 4.49E-05 | 3.74E-09 | 6.12E-05 | 0.73408 | 0.4629 | 0.99961 |
| 2 | 44313803 | 46482634 | 1934 | -7.38E-06 | 2.87E-09 | 5.36E-05 | -0.1378 | 0.8904 | 0.99961 |
| 2 | 46482634 | 47318772 | 772 | -1.06E-05 | 3.08E-09 | 5.55E-05 | -0.19026 | 0.8491 | 0.99961 |
| 2 | 47318772 | 48213001 | 507 | 4.51E-05 | 3.05E-09 | 5.52E-05 | 0.81633 | 0.41431 | 0.99961 |
| 2 | 48213001 | 50022257 | 1487 | 4.19E-05 | 4.22E-09 | 6.50E-05 | 0.64426 | 0.51941 | 0.99961 |
| 2 | 50022257 | 50818928 | 805 | 3.31E-05 | 2.59E-09 | 5.09E-05 | 0.65112 | 0.51497 | 0.99961 |
| 2 | 50818928 | 52491133 | 1639 | -2.18E-05 | 2.91E-09 | 5.39E-05 | -0.4034 | 0.68666 | 0.99961 |
| 2 | 52491133 | 53508452 | 1078 | 4.36E-05 | 3.08E-09 | 5.55E-05 | 0.78567 | 0.43206 | 0.99961 |
| 2 | 53508452 | 54685226 | 872 | 2.98E-06 | 2.86E-09 | 5.35E-05 | 0.055741 | 0.95555 | 0.99961 |
| 2 | 54685226 | 56203345 | 1200 | 3.75E-07 | 2.95E-09 | 5.43E-05 | 0.006908 | 0.99449 | 0.99961 |
| 2 | 56203345 | 57429100 | 945 | -2.21E-05 | 3.15E-09 | 5.62E-05 | -0.39285 | 0.69443 | 0.99961 |
| 2 | 57429100 | 58297315 | 647 | -1.90E-05 | 2.10E-09 | 4.59E-05 | -0.41371 | 0.67909 | 0.99961 |
| 2 | 58297315 | 60292000 | 1311 | 3.19E-05 | 3.43E-09 | 5.86E-05 | 0.54433 | 0.58621 | 0.99961 |
| 2 | 60292000 | 62429044 | 1025 | -2.04E-05 | 3.46E-09 | 5.89E-05 | -0.34654 | 0.72893 | 0.99961 |
| 2 | 62429044 | 64625913 | 1111 | 3.32E-05 | 3.37E-09 | 5.81E-05 | 0.57219 | 0.56719 | 0.99961 |
| 2 | 64625913 | 65933444 | 870 | 1.74E-05 | 3.80E-09 | 6.17E-05 | 0.28185 | 0.77806 | 0.99961 |
| 2 | 65933444 | 67226166 | 1136 | 2.77E-06 | 4.48E-09 | 6.69E-05 | 0.041365 | 0.967 | 0.99961 |
| 2 | 67226166 | 68010176 | 916 | 0.00011134 | 5.70E-09 | 7.55E-05 | 1.4742 | 0.14042 | 0.99961 |
| 2 | 68010176 | 69139564 | 1012 | -1.77E-05 | 2.99E-09 | 5.46E-05 | -0.32457 | 0.74551 | 0.99961 |
| 2 | 69139564 | 70755198 | 957 | -1.53E-05 | 3.13E-09 | 5.60E-05 | -0.27406 | 0.78404 | 0.99961 |
| 2 | 70755198 | 73174848 | 1761 | 5.86E-06 | 2.76E-09 | 5.25E-05 | 0.11152 | 0.9112 | 0.99961 |
| 2 | 73174848 | 75630086 | 1432 | -1.06E-05 | 2.74E-09 | 5.23E-05 | -0.20283 | 0.83927 | 0.99961 |
| 2 | 75630086 | 76913661 | 1052 | 1.84E-07 | 2.77E-09 | 5.27E-05 | 0.003495 | 0.99721 | 0.99961 |
| 2 | 76913661 | 78079264 | 905 | 3.51E-06 | 2.98E-09 | 5.46E-05 | 0.064359 | 0.94868 | 0.99961 |
| 2 | 78079264 | 80050403 | 1986 | 2.18E-05 | 3.15E-09 | 5.61E-05 | 0.38818 | 0.69788 | 0.99961 |
| 2 | 80050403 | 81620841 | 1117 | 2.81E-05 | 2.85E-09 | 5.34E-05 | 0.52552 | 0.59922 | 0.99961 |
| 2 | 81620841 | 85140680 | 2386 | 9.38E-06 | 2.81E-09 | 5.30E-05 | 0.17692 | 0.85957 | 0.99961 |
| 2 | 85140680 | 88038507 | 1195 | 3.12E-05 | 2.86E-09 | 5.35E-05 | 0.58386 | 0.55931 | 0.99961 |
| 2 | 88038507 | 89154526 | 431 | -5.50E-05 | 1.51E-09 | 3.89E-05 | -1.413 | 0.15764 | 0.99961 |
| 2 | 89154526 | 95326452 | 12 | -8.67E-06 | 1.99E-10 | 1.41E-05 | -0.61403 | 0.5392 | 0.99961 |
| 2 | 95326452 | 98995201 | 837 | -3.05E-05 | 3.24E-09 | 5.69E-05 | -0.53582 | 0.59208 | 0.99961 |
| 2 | 98995201 | 101822329 | 1883 | -6.65E-05 | 4.10E-09 | 6.41E-05 | -1.038 | 0.29927 | 0.99961 |
| 2 | 101822329 | 102688765 | 587 | 4.32E-05 | 1.83E-09 | 4.28E-05 | 1.0113 | 0.31189 | 0.99961 |
| 2 | 102688765 | 103588215 | 948 | -5.85E-05 | 2.47E-09 | 4.97E-05 | -1.1764 | 0.23945 | 0.99961 |
| 2 | 103588215 | 105125034 | 772 | -1.13E-05 | 2.72E-09 | 5.22E-05 | -0.21628 | 0.82877 | 0.99961 |
| 2 | 105125034 | 106210058 | 839 | 4.51E-06 | 3.05E-09 | 5.52E-05 | 0.081734 | 0.93486 | 0.99961 |
| 2 | 106210058 | 107408267 | 916 | 1.11E-05 | 2.90E-09 | 5.38E-05 | 0.20607 | 0.83674 | 0.99961 |
| 2 | 107408267 | 108429177 | 917 | 2.30E-07 | 1.85E-09 | 4.30E-05 | 0.005349 | 0.99573 | 0.99961 |
| 2 | 108429177 | 110572432 | 1116 | 5.76E-06 | 2.88E-09 | 5.37E-05 | 0.10721 | 0.91462 | 0.99961 |
| 2 | 110572432 | 113921856 | 1381 | 8.25E-06 | 3.04E-09 | 5.51E-05 | 0.14974 | 0.88097 | 0.99961 |
| 2 | 113921856 | 116772470 | 1643 | 9.87E-05 | 3.32E-09 | 5.76E-05 | 1.7129 | 0.086722 | 0.99961 |
| 2 | 116772470 | 118367466 | 1007 | -1.32E-05 | 2.28E-09 | 4.78E-05 | -0.27675 | 0.78197 | 0.99961 |
| 2 | 118367466 | 121303783 | 2010 | 1.45E-05 | 3.07E-09 | 5.54E-05 | 0.26151 | 0.7937 | 0.99961 |
| 2 | 121303783 | 123302906 | 1205 | -1.73E-05 | 3.13E-09 | 5.60E-05 | -0.30953 | 0.75692 | 0.99961 |
| 2 | 123302906 | 124732250 | 1183 | -3.00E-06 | 2.14E-09 | 4.62E-05 | -0.0649 | 0.94826 | 0.99961 |
| 2 | 124732250 | 125840921 | 872 | -2.64E-05 | 2.25E-09 | 4.74E-05 | -0.55682 | 0.57765 | 0.99961 |
| 2 | 125840921 | 127373764 | 1055 | -1.04E-05 | 1.98E-09 | 4.45E-05 | -0.23269 | 0.816 | 0.99961 |
| 2 | 127373764 | 128034347 | 599 | -1.47E-05 | 1.95E-09 | 4.42E-05 | -0.33207 | 0.73984 | 0.99961 |
| 2 | 128034347 | 129474441 | 835 | 1.96E-05 | 3.07E-09 | 5.54E-05 | 0.35447 | 0.72299 | 0.99961 |
| 2 | 129474441 | 130067170 | 451 | -2.63E-05 | 1.37E-09 | 3.70E-05 | -0.71019 | 0.47758 | 0.99961 |
| 2 | 130067170 | 130658085 | 451 | 2.51E-05 | 1.55E-09 | 3.94E-05 | 0.63568 | 0.52498 | 0.99961 |
| 2 | 130658085 | 133042784 | 475 | -3.21E-05 | 2.06E-09 | 4.54E-05 | -0.70672 | 0.47974 | 0.99961 |
| 2 | 133042784 | 135158578 | 1714 | 1.69E-06 | 2.78E-09 | 5.27E-05 | 0.032115 | 0.97438 | 0.99961 |
| 2 | 135158578 | 137042794 | 840 | -1.10E-07 | 2.32E-09 | 4.81E-05 | -0.00229 | 0.99817 | 0.99961 |
| 2 | 137042794 | 138698117 | 1209 | 1.85E-06 | 3.06E-09 | 5.53E-05 | 0.033381 | 0.97337 | 0.99961 |
| 2 | 138698117 | 140100271 | 1006 | -3.71E-06 | 2.10E-09 | 4.58E-05 | -0.08104 | 0.93541 | 0.99961 |
| 2 | 140100271 | 141805351 | 1378 | -3.78E-05 | 3.13E-09 | 5.59E-05 | -0.67539 | 0.49943 | 0.99961 |
| 2 | 141805351 | 142518602 | 695 | -2.46E-06 | 1.89E-09 | 4.34E-05 | -0.05666 | 0.95482 | 0.99961 |
| 2 | 142518602 | 144519484 | 1468 | -1.30E-05 | 2.99E-09 | 5.47E-05 | -0.23744 | 0.81231 | 0.99961 |
| 2 | 144519484 | 146445570 | 849 | 2.89E-05 | 2.91E-09 | 5.40E-05 | 0.53601 | 0.59195 | 0.99961 |
| 2 | 146445570 | 147277162 | 371 | 4.63E-05 | 1.65E-09 | 4.06E-05 | 1.1386 | 0.25485 | 0.99961 |
| 2 | 147277162 | 150210292 | 1467 | 4.98E-07 | 3.22E-09 | 5.68E-05 | 0.008774 | 0.993 | 0.99961 |
| 2 | 150210292 | 151731462 | 1029 | -2.07E-05 | 3.32E-09 | 5.76E-05 | -0.35902 | 0.71958 | 0.99961 |
| 2 | 151731462 | 152486782 | 572 | 5.08E-06 | 2.10E-09 | 4.59E-05 | 0.11066 | 0.91189 | 0.99961 |
| 2 | 152486782 | 154005056 | 1004 | -6.76E-06 | 3.13E-09 | 5.59E-05 | -0.12086 | 0.90381 | 0.99961 |
| 2 | 154005056 | 155391135 | 1124 | 1.56E-05 | 2.67E-09 | 5.17E-05 | 0.30097 | 0.76344 | 0.99961 |
| 2 | 155391135 | 157560635 | 1205 | -1.39E-05 | 3.11E-09 | 5.58E-05 | -0.24934 | 0.8031 | 0.99961 |
| 2 | 157560635 | 158533218 | 346 | 5.70E-05 | 3.14E-09 | 5.61E-05 | 1.0169 | 0.30921 | 0.99961 |
| 2 | 158533218 | 159577082 | 695 | -3.30E-05 | 2.37E-09 | 4.86E-05 | -0.6777 | 0.49796 | 0.99961 |
| 2 | 159577082 | 161769733 | 1353 | 2.68E-05 | 3.24E-09 | 5.69E-05 | 0.47107 | 0.63759 | 0.99961 |
| 2 | 161769733 | 163503551 | 706 | 5.51E-06 | 2.07E-09 | 4.55E-05 | 0.12107 | 0.90364 | 0.99961 |
| 2 | 163503551 | 164466638 | 520 | 3.74E-05 | 1.89E-09 | 4.35E-05 | 0.86051 | 0.38951 | 0.99961 |
| 2 | 164466638 | 165178840 | 405 | -3.93E-05 | 2.11E-09 | 4.59E-05 | -0.85523 | 0.39242 | 0.99961 |
| 2 | 165178840 | 167160270 | 1341 | -2.74E-06 | 8.13E-09 | 9.02E-05 | -0.03035 | 0.97579 | 0.99961 |
| 2 | 167160270 | 167355970 | 167 | -2.91E-05 | 7.22E-10 | 2.69E-05 | -1.0815 | 0.27946 | 0.99961 |
| 2 | 167355970 | 169968236 | 1605 | 1.63E-05 | 3.30E-09 | 5.75E-05 | 0.28289 | 0.77726 | 0.99961 |
| 2 | 169968236 | 171226245 | 963 | -1.69E-05 | 3.16E-09 | 5.62E-05 | -0.30037 | 0.7639 | 0.99961 |
| 2 | 171226245 | 173138905 | 999 | 1.17E-05 | 3.30E-09 | 5.74E-05 | 0.20384 | 0.83848 | 0.99961 |
| 2 | 173138905 | 175588929 | 1598 | -6.82E-06 | 2.86E-09 | 5.35E-05 | -0.12748 | 0.89856 | 0.99961 |
| 2 | 175588929 | 177363636 | 978 | -4.03E-05 | 3.01E-09 | 5.48E-05 | -0.73525 | 0.46219 | 0.99961 |
| 2 | 177363636 | 178553183 | 1032 | -1.42E-05 | 2.87E-09 | 5.36E-05 | -0.26589 | 0.79032 | 0.99961 |
| 2 | 178553183 | 181312739 | 2162 | -1.88E-05 | 2.91E-09 | 5.40E-05 | -0.34778 | 0.728 | 0.99961 |
| 2 | 181312739 | 182266031 | 688 | 1.20E-05 | 1.84E-09 | 4.29E-05 | 0.27863 | 0.78053 | 0.99961 |
| 2 | 182266031 | 184357608 | 1296 | 2.31E-05 | 2.88E-09 | 5.37E-05 | 0.42977 | 0.66736 | 0.99961 |
| 2 | 184357608 | 185280173 | 599 | -6.06E-05 | 1.86E-09 | 4.31E-05 | -1.4066 | 0.15956 | 0.99961 |
| 2 | 185280173 | 189882065 | 2470 | 0.00011804 | 4.41E-09 | 6.64E-05 | 1.778 | 0.075406 | 0.99961 |
| 2 | 189882065 | 191973357 | 1042 | 4.11E-05 | 3.27E-09 | 5.72E-05 | 0.71957 | 0.47179 | 0.99961 |
| 2 | 191973357 | 195861164 | 1685 | -1.19E-05 | 2.77E-09 | 5.26E-05 | -0.22543 | 0.82164 | 0.99961 |
| 2 | 195861164 | 196447456 | 326 | 1.74E-05 | 1.10E-09 | 3.32E-05 | 0.523 | 0.60097 | 0.99961 |
| 2 | 196447456 | 198078110 | 873 | 2.73E-05 | 2.90E-09 | 5.38E-05 | 0.50633 | 0.61262 | 0.99961 |
| 2 | 198078110 | 199311125 | 593 | 1.20E-05 | 1.64E-09 | 4.04E-05 | 0.2979 | 0.76578 | 0.99961 |
| 2 | 199311125 | 201576284 | 1227 | -9.24E-06 | 3.06E-09 | 5.53E-05 | -0.16703 | 0.86735 | 0.99961 |
| 2 | 201576284 | 202818637 | 508 | 2.47E-05 | 2.28E-09 | 4.77E-05 | 0.51754 | 0.60478 | 0.99961 |
| 2 | 202818637 | 205799241 | 1346 | 2.86E-06 | 2.74E-09 | 5.24E-05 | 0.054614 | 0.95645 | 0.99961 |
| 2 | 205799241 | 206735704 | 663 | 8.83E-06 | 3.31E-09 | 5.75E-05 | 0.1534 | 0.87808 | 0.99961 |
| 2 | 206735704 | 208645399 | 1158 | 1.92E-05 | 3.35E-09 | 5.79E-05 | 0.33095 | 0.74068 | 0.99961 |
| 2 | 208645399 | 209941529 | 712 | -3.61E-05 | 2.44E-09 | 4.94E-05 | -0.73022 | 0.46526 | 0.99961 |
| 2 | 209941529 | 212379518 | 1662 | 2.70E-05 | 2.93E-09 | 5.41E-05 | 0.49907 | 0.61773 | 0.99961 |
| 2 | 212379518 | 214014282 | 1402 | -2.64E-05 | 2.83E-09 | 5.32E-05 | -0.49689 | 0.61926 | 0.99961 |
| 2 | 214014282 | 215573795 | 1029 | 4.93E-05 | 3.09E-09 | 5.56E-05 | 0.88638 | 0.37541 | 0.99961 |
| 2 | 215573795 | 217715661 | 1468 | -5.31E-06 | 2.95E-09 | 5.43E-05 | -0.09778 | 0.92211 | 0.99961 |
| 2 | 217715661 | 218395480 | 512 | 4.02E-05 | 3.60E-09 | 6.00E-05 | 0.66999 | 0.50286 | 0.99961 |
| 2 | 218395480 | 220454551 | 906 | 1.96E-05 | 3.81E-09 | 6.17E-05 | 0.31684 | 0.75137 | 0.99961 |
| 2 | 220454551 | 222521176 | 1800 | 2.58E-05 | 2.81E-09 | 5.30E-05 | 0.48743 | 0.62596 | 0.99961 |
| 2 | 222521176 | 224859419 | 1664 | 2.54E-06 | 2.84E-09 | 5.33E-05 | 0.047742 | 0.96192 | 0.99961 |
| 2 | 224859419 | 225840004 | 721 | -1.87E-05 | 2.64E-09 | 5.13E-05 | -0.36325 | 0.71641 | 0.99961 |
| 2 | 225840004 | 228119842 | 1575 | -2.12E-06 | 3.64E-09 | 6.03E-05 | -0.03518 | 0.97193 | 0.99961 |
| 2 | 228119842 | 229370787 | 906 | 7.29E-06 | 2.64E-09 | 5.14E-05 | 0.14191 | 0.88715 | 0.99961 |
| 2 | 229370787 | 231843389 | 1822 | -4.17E-07 | 2.65E-09 | 5.15E-05 | -0.0081 | 0.99354 | 0.99961 |
| 2 | 231843389 | 233550003 | 774 | -1.76E-05 | 3.27E-09 | 5.72E-05 | -0.30846 | 0.75774 | 0.99961 |
| 2 | 233550003 | 235150987 | 1425 | 7.46E-06 | 2.94E-09 | 5.42E-05 | 0.13762 | 0.89054 | 0.99961 |
| 2 | 235150987 | 236043550 | 735 | -3.47E-05 | 3.46E-09 | 5.88E-05 | -0.58994 | 0.55523 | 0.99961 |
| 2 | 236043550 | 237449032 | 877 | 2.39E-06 | 3.11E-09 | 5.57E-05 | 0.042789 | 0.96587 | 0.99961 |
| 2 | 237449032 | 238760981 | 1052 | -2.57E-07 | 2.86E-09 | 5.34E-05 | -0.00481 | 0.99617 | 0.99961 |
| 2 | 238760981 | 239952643 | 805 | -3.38E-05 | 3.04E-09 | 5.51E-05 | -0.61358 | 0.53949 | 0.99961 |
| 2 | 239952643 | 241560084 | 1178 | -7.99E-06 | 2.96E-09 | 5.44E-05 | -0.14682 | 0.88327 | 0.99961 |
| 2 | 241560084 | 242149921 | 369 | -9.52E-06 | 1.97E-09 | 4.44E-05 | -0.21441 | 0.83023 | 0.99961 |
| 2 | 242149921 | 243188920 | 203 | -7.18E-06 | 9.88E-10 | 3.14E-05 | -0.2285 | 0.81925 | 0.99961 |
| 3 | 60157 | 1441779 | 1199 | -3.66E-05 | 3.47E-09 | 5.89E-05 | -0.62183 | 0.53405 | 0.99961 |
| 3 | 1441779 | 2992120 | 1642 | 5.14E-06 | 3.19E-09 | 5.65E-05 | 0.09098 | 0.92751 | 0.99961 |
| 3 | 2992120 | 4431647 | 1477 | 2.58E-05 | 3.48E-09 | 5.90E-05 | 0.43677 | 0.66228 | 0.99961 |
| 3 | 4431647 | 6083183 | 1421 | 5.17E-06 | 3.20E-09 | 5.66E-05 | 0.091299 | 0.92726 | 0.99961 |
| 3 | 6083183 | 7083387 | 886 | 3.23E-05 | 3.16E-09 | 5.62E-05 | 0.57505 | 0.56526 | 0.99961 |
| 3 | 7083387 | 8648561 | 1708 | 7.10E-06 | 3.08E-09 | 5.55E-05 | 0.12792 | 0.89822 | 0.99961 |
| 3 | 8648561 | 9543183 | 783 | -1.24E-05 | 3.29E-09 | 5.74E-05 | -0.2157 | 0.82922 | 0.99961 |
| 3 | 9543183 | 11019665 | 929 | 4.49E-06 | 3.33E-09 | 5.77E-05 | 0.077851 | 0.93795 | 0.99961 |
| 3 | 11019665 | 13070799 | 1288 | 8.68E-05 | 7.29E-09 | 8.54E-05 | 1.0172 | 0.30908 | 0.99961 |
| 3 | 13070799 | 14816900 | 1400 | 1.21E-05 | 3.22E-09 | 5.67E-05 | 0.2127 | 0.83156 | 0.99961 |
| 3 | 14816900 | 16282442 | 975 | 3.98E-05 | 3.68E-09 | 6.07E-05 | 0.65591 | 0.51188 | 0.99961 |
| 3 | 16282442 | 17891118 | 1008 | -4.08E-05 | 3.65E-09 | 6.04E-05 | -0.67575 | 0.4992 | 0.99961 |
| 3 | 17891118 | 19125144 | 746 | 2.70E-05 | 2.23E-09 | 4.72E-05 | 0.57119 | 0.56787 | 0.99961 |
| 3 | 19125144 | 20091348 | 331 | -1.75E-05 | 1.64E-09 | 4.04E-05 | -0.43365 | 0.66454 | 0.99961 |
| 3 | 20091348 | 21643707 | 1287 | 2.61E-05 | 3.17E-09 | 5.63E-05 | 0.46319 | 0.64323 | 0.99961 |
| 3 | 21643707 | 22204244 | 869 | -4.94E-05 | 3.57E-09 | 5.98E-05 | -0.8259 | 0.40886 | 0.99961 |
| 3 | 22204244 | 23804865 | 1131 | 6.34E-06 | 3.37E-09 | 5.80E-05 | 0.10925 | 0.91301 | 0.99961 |
| 3 | 23804865 | 25461558 | 1396 | 2.61E-05 | 4.11E-09 | 6.41E-05 | 0.40722 | 0.68385 | 0.99961 |
| 3 | 25461558 | 26877769 | 947 | 8.36E-06 | 3.65E-09 | 6.04E-05 | 0.13829 | 0.89001 | 0.99961 |
| 3 | 26877769 | 27840910 | 640 | 4.35E-06 | 2.38E-09 | 4.88E-05 | 0.08922 | 0.92891 | 0.99961 |
| 3 | 27840910 | 29142260 | 917 | -2.32E-05 | 3.15E-09 | 5.62E-05 | -0.41376 | 0.67905 | 0.99961 |
| 3 | 29142260 | 30717955 | 1329 | -1.20E-05 | 2.97E-09 | 5.45E-05 | -0.22028 | 0.82565 | 0.99961 |
| 3 | 30717955 | 32351715 | 1228 | -2.52E-06 | 3.12E-09 | 5.59E-05 | -0.04516 | 0.96398 | 0.99961 |
| 3 | 32351715 | 33255592 | 459 | 8.12E-06 | 2.46E-09 | 4.96E-05 | 0.16361 | 0.87004 | 0.99961 |
| 3 | 33255592 | 35283458 | 1213 | 7.39E-06 | 3.75E-09 | 6.12E-05 | 0.12065 | 0.90397 | 0.99961 |
| 3 | 35283458 | 36486842 | 863 | 1.81E-05 | 3.09E-09 | 5.56E-05 | 0.32649 | 0.74406 | 0.99961 |
| 3 | 36486842 | 38356116 | 1030 | -7.55E-05 | 3.57E-09 | 5.97E-05 | -1.2638 | 0.20631 | 0.99961 |
| 3 | 38356116 | 40221298 | 1268 | -4.18E-05 | 3.22E-09 | 5.67E-05 | -0.73635 | 0.46152 | 0.99961 |
| 3 | 40221298 | 41064528 | 300 | 3.77E-06 | 1.06E-09 | 3.25E-05 | 0.11584 | 0.90778 | 0.99961 |
| 3 | 41064528 | 42540002 | 968 | 8.43E-06 | 2.89E-09 | 5.37E-05 | 0.15693 | 0.8753 | 0.99961 |
| 3 | 42540002 | 45165153 | 1261 | -2.14E-05 | 3.84E-09 | 6.20E-05 | -0.346 | 0.72934 | 0.99961 |
| 3 | 45165153 | 46657500 | 988 | -2.20E-06 | 2.99E-09 | 5.46E-05 | -0.04035 | 0.96782 | 0.99961 |
| 3 | 46657500 | 47727212 | 297 | -4.82E-05 | 2.02E-09 | 4.49E-05 | -1.073 | 0.28327 | 0.99961 |
| 3 | 47727212 | 49316972 | 350 | -1.59E-05 | 1.98E-09 | 4.45E-05 | -0.35653 | 0.72145 | 0.99961 |
| 3 | 49316972 | 51832015 | 650 | 1.89E-05 | 2.47E-09 | 4.97E-05 | 0.38028 | 0.70374 | 0.99961 |
| 3 | 51832015 | 54081390 | 994 | -0.00012665 | 5.95E-09 | 7.71E-05 | -1.642 | 0.10058 | 0.99961 |
| 3 | 54081390 | 55348860 | 1112 | -5.16E-05 | 3.11E-09 | 5.57E-05 | -0.92581 | 0.35454 | 0.99961 |
| 3 | 55348860 | 56433907 | 708 | 3.70E-06 | 3.15E-09 | 5.61E-05 | 0.065922 | 0.94744 | 0.99961 |
| 3 | 56433907 | 58157519 | 947 | 1.51E-05 | 4.19E-09 | 6.48E-05 | 0.23304 | 0.81573 | 0.99961 |
| 3 | 58157519 | 60272906 | 1758 | 2.15E-05 | 3.12E-09 | 5.59E-05 | 0.38436 | 0.70071 | 0.99961 |
| 3 | 60272906 | 62355666 | 1753 | 4.02E-05 | 3.43E-09 | 5.86E-05 | 0.686 | 0.49272 | 0.99961 |
| 3 | 62355666 | 63669286 | 1247 | -6.06E-05 | 3.19E-09 | 5.65E-05 | -1.073 | 0.28328 | 0.99961 |
| 3 | 63669286 | 65273538 | 1309 | 0.00020303 | 8.66E-09 | 9.30E-05 | 2.182 | 0.029109 | 0.99961 |
| 3 | 65273538 | 66270447 | 587 | 1.28E-05 | 3.20E-09 | 5.66E-05 | 0.22598 | 0.82122 | 0.99961 |
| 3 | 66270447 | 68286914 | 1337 | -1.07E-06 | 3.77E-09 | 6.14E-05 | -0.01739 | 0.98613 | 0.99961 |
| 3 | 68286914 | 69276762 | 688 | -1.76E-05 | 2.90E-09 | 5.39E-05 | -0.3269 | 0.74375 | 0.99961 |
| 3 | 69276762 | 70449028 | 830 | -3.05E-05 | 3.96E-09 | 6.29E-05 | -0.4849 | 0.62775 | 0.99961 |
| 3 | 70449028 | 72529329 | 1389 | -1.58E-05 | 3.19E-09 | 5.65E-05 | -0.28045 | 0.77913 | 0.99961 |
| 3 | 72529329 | 74321817 | 1433 | -1.70E-05 | 3.07E-09 | 5.54E-05 | -0.30736 | 0.75857 | 0.99961 |
| 3 | 74321817 | 75704949 | 567 | -3.86E-07 | 2.05E-09 | 4.52E-05 | -0.00854 | 0.99319 | 0.99961 |
| 3 | 75704949 | 77508835 | 1097 | 7.76E-06 | 3.50E-09 | 5.92E-05 | 0.13104 | 0.89574 | 0.99961 |
| 3 | 77508835 | 79024541 | 890 | 1.86E-05 | 3.55E-09 | 5.95E-05 | 0.31249 | 0.75467 | 0.99961 |
| 3 | 79024541 | 81427310 | 1271 | -3.38E-05 | 3.09E-09 | 5.56E-05 | -0.60859 | 0.5428 | 0.99961 |
| 3 | 81427310 | 82643886 | 697 | -1.62E-05 | 2.06E-09 | 4.54E-05 | -0.35739 | 0.7208 | 0.99961 |
| 3 | 82643886 | 84367479 | 841 | 1.51E-06 | 2.51E-09 | 5.01E-05 | 0.030164 | 0.97594 | 0.99961 |
| 3 | 84367479 | 85582231 | 628 | -1.28E-05 | 1.80E-09 | 4.24E-05 | -0.30113 | 0.76332 | 0.99961 |
| 3 | 85582231 | 87409732 | 1245 | 6.72E-06 | 3.17E-09 | 5.63E-05 | 0.11934 | 0.90501 | 0.99961 |
| 3 | 87409732 | 88298373 | 503 | -2.50E-05 | 1.28E-09 | 3.58E-05 | -0.69715 | 0.48571 | 0.99961 |
| 3 | 88298373 | 94255316 | 1208 | -1.76E-06 | 3.30E-09 | 5.74E-05 | -0.03065 | 0.97555 | 0.99961 |
| 3 | 94255316 | 95311971 | 738 | -6.06E-06 | 1.67E-09 | 4.09E-05 | -0.14804 | 0.88231 | 0.99961 |
| 3 | 95311971 | 98215950 | 1513 | -4.63E-05 | 3.40E-09 | 5.83E-05 | -0.79414 | 0.42711 | 0.99961 |
| 3 | 98215950 | 99373762 | 785 | -7.46E-06 | 2.91E-09 | 5.39E-05 | -0.13826 | 0.89003 | 0.99961 |
| 3 | 99373762 | 100592217 | 597 | -1.89E-05 | 2.49E-09 | 4.99E-05 | -0.37971 | 0.70416 | 0.99961 |
| 3 | 100592217 | 103084960 | 1430 | -8.97E-06 | 3.74E-09 | 6.12E-05 | -0.14653 | 0.8835 | 0.99961 |
| 3 | 103084960 | 104581613 | 1148 | 1.91E-05 | 2.72E-09 | 5.22E-05 | 0.36588 | 0.71445 | 0.99961 |
| 3 | 104581613 | 106982811 | 1582 | 1.68E-05 | 3.50E-09 | 5.91E-05 | 0.28428 | 0.77619 | 0.99961 |
| 3 | 106982811 | 109522395 | 1702 | 4.87E-05 | 3.75E-09 | 6.12E-05 | 0.7954 | 0.42638 | 0.99961 |
| 3 | 109522395 | 110513770 | 685 | 7.70E-06 | 1.38E-09 | 3.71E-05 | 0.20751 | 0.83561 | 0.99961 |
| 3 | 110513770 | 112815699 | 1464 | -0.0001287 | 3.94E-09 | 6.27E-05 | -2.0514 | 0.040224 | 0.99961 |
| 3 | 112815699 | 113947863 | 674 | -4.18E-05 | 2.68E-09 | 5.17E-05 | -0.80808 | 0.41904 | 0.99961 |
| 3 | 113947863 | 115447080 | 762 | -1.48E-05 | 3.20E-09 | 5.66E-05 | -0.26225 | 0.79313 | 0.99961 |
| 3 | 115447080 | 116800153 | 1024 | 3.51E-05 | 3.49E-09 | 5.91E-05 | 0.59479 | 0.55198 | 0.99961 |
| 3 | 116800153 | 118530251 | 1326 | 1.87E-05 | 3.25E-09 | 5.70E-05 | 0.32847 | 0.74256 | 0.99961 |
| 3 | 118530251 | 120523055 | 1153 | -2.03E-05 | 3.21E-09 | 5.67E-05 | -0.35746 | 0.72075 | 0.99961 |
| 3 | 120523055 | 121974097 | 768 | -8.01E-06 | 1.66E-09 | 4.07E-05 | -0.19676 | 0.84402 | 0.99961 |
| 3 | 121974097 | 123517768 | 958 | 4.85E-06 | 3.78E-09 | 6.15E-05 | 0.078842 | 0.93716 | 0.99961 |
| 3 | 123517768 | 124936642 | 1165 | 1.16E-05 | 3.42E-09 | 5.85E-05 | 0.19898 | 0.84228 | 0.99961 |
| 3 | 124936642 | 126214943 | 884 | -1.22E-05 | 3.22E-09 | 5.67E-05 | -0.21491 | 0.82984 | 0.99961 |
| 3 | 126214943 | 128194861 | 1230 | -4.74E-05 | 3.54E-09 | 5.95E-05 | -0.79683 | 0.42555 | 0.99961 |
| 3 | 128194861 | 130244735 | 772 | 4.58E-05 | 7.43E-09 | 8.62E-05 | 0.53095 | 0.59545 | 0.99961 |
| 3 | 130244735 | 131836516 | 1120 | -5.26E-05 | 2.99E-09 | 5.46E-05 | -0.96304 | 0.33553 | 0.99961 |
| 3 | 131836516 | 133252173 | 1051 | 2.32E-05 | 2.95E-09 | 5.43E-05 | 0.42716 | 0.66926 | 0.99961 |
| 3 | 133252173 | 135456906 | 1608 | 4.36E-05 | 3.70E-09 | 6.09E-05 | 0.71679 | 0.47351 | 0.99961 |
| 3 | 135456906 | 137371083 | 853 | 7.11E-06 | 2.57E-09 | 5.07E-05 | 0.14036 | 0.88838 | 0.99961 |
| 3 | 137371083 | 139954597 | 1600 | 4.99E-05 | 3.78E-09 | 6.15E-05 | 0.81037 | 0.41773 | 0.99961 |
| 3 | 139954597 | 141339097 | 837 | 4.97E-05 | 3.19E-09 | 5.65E-05 | 0.88066 | 0.3785 | 0.99961 |
| 3 | 141339097 | 143164628 | 1080 | 2.00E-05 | 3.47E-09 | 5.89E-05 | 0.34038 | 0.73357 | 0.99961 |
| 3 | 143164628 | 144410816 | 817 | 2.93E-05 | 3.01E-09 | 5.49E-05 | 0.53303 | 0.59401 | 0.99961 |
| 3 | 144410816 | 145664619 | 1030 | 2.30E-05 | 2.57E-09 | 5.06E-05 | 0.45488 | 0.6492 | 0.99961 |
| 3 | 145664619 | 146726629 | 850 | 1.15E-05 | 2.55E-09 | 5.05E-05 | 0.22825 | 0.81945 | 0.99961 |
| 3 | 146726629 | 149043237 | 1394 | 3.67E-05 | 3.39E-09 | 5.83E-05 | 0.62989 | 0.52876 | 0.99961 |
| 3 | 149043237 | 150252004 | 808 | 4.18E-05 | 4.69E-09 | 6.85E-05 | 0.61023 | 0.54171 | 0.99961 |
| 3 | 150252004 | 151348730 | 645 | 3.20E-05 | 3.02E-09 | 5.49E-05 | 0.5833 | 0.55969 | 0.99961 |
| 3 | 151348730 | 153256571 | 1250 | 2.04E-05 | 3.44E-09 | 5.87E-05 | 0.34823 | 0.72767 | 0.99961 |
| 3 | 153256571 | 154714218 | 961 | 6.50E-05 | 2.84E-09 | 5.33E-05 | 1.2207 | 0.22218 | 0.99961 |
| 3 | 154714218 | 156008700 | 682 | 3.85E-06 | 3.09E-09 | 5.56E-05 | 0.069239 | 0.9448 | 0.99961 |
| 3 | 156008700 | 157312028 | 917 | 8.01E-05 | 4.40E-09 | 6.63E-05 | 1.2077 | 0.22715 | 0.99961 |
| 3 | 157312028 | 159477890 | 1406 | 5.44E-06 | 3.37E-09 | 5.81E-05 | 0.093599 | 0.92543 | 0.99961 |
| 3 | 159477890 | 161524504 | 1236 | 2.86E-05 | 3.08E-09 | 5.55E-05 | 0.51644 | 0.60555 | 0.99961 |
| 3 | 161524504 | 163253205 | 1552 | -5.77E-05 | 3.02E-09 | 5.50E-05 | -1.0493 | 0.29405 | 0.99961 |
| 3 | 163253205 | 165055469 | 1167 | -7.42E-06 | 2.29E-09 | 4.78E-05 | -0.15508 | 0.87676 | 0.99961 |
| 3 | 165055469 | 167117429 | 1160 | 5.36E-06 | 2.71E-09 | 5.21E-05 | 0.10298 | 0.91798 | 0.99961 |
| 3 | 167117429 | 168580960 | 754 | 3.27E-05 | 2.55E-09 | 5.05E-05 | 0.64653 | 0.51794 | 0.99961 |
| 3 | 168580960 | 170964909 | 1385 | -5.08E-05 | 4.10E-09 | 6.40E-05 | -0.79409 | 0.42714 | 0.99961 |
| 3 | 170964909 | 172295731 | 895 | 3.38E-06 | 3.54E-09 | 5.95E-05 | 0.056801 | 0.9547 | 0.99961 |
| 3 | 172295731 | 173477285 | 739 | -4.11E-06 | 3.59E-09 | 5.99E-05 | -0.06869 | 0.94523 | 0.99961 |
| 3 | 173477285 | 175116539 | 1298 | 5.22E-06 | 3.14E-09 | 5.60E-05 | 0.093165 | 0.92577 | 0.99961 |
| 3 | 175116539 | 176320287 | 812 | -9.89E-06 | 2.35E-09 | 4.85E-05 | -0.20386 | 0.83846 | 0.99961 |
| 3 | 176320287 | 178107165 | 1084 | -3.43E-05 | 3.29E-09 | 5.74E-05 | -0.59756 | 0.55013 | 0.99961 |
| 3 | 178107165 | 179374586 | 780 | -1.69E-05 | 2.74E-09 | 5.23E-05 | -0.32222 | 0.74729 | 0.99961 |
| 3 | 179374586 | 181511166 | 915 | -1.11E-05 | 3.08E-09 | 5.55E-05 | -0.19903 | 0.84224 | 0.99961 |
| 3 | 181511166 | 183769683 | 1195 | -3.55E-05 | 3.22E-09 | 5.67E-05 | -0.6261 | 0.53125 | 0.99961 |
| 3 | 183769683 | 185068255 | 729 | 4.09E-05 | 3.60E-09 | 6.00E-05 | 0.6819 | 0.4953 | 0.99961 |
| 3 | 185068255 | 186890344 | 1020 | 5.65E-05 | 3.87E-09 | 6.22E-05 | 0.9087 | 0.36351 | 0.99961 |
| 3 | 186890344 | 188856214 | 1476 | 2.32E-05 | 3.85E-09 | 6.20E-05 | 0.37373 | 0.7086 | 0.99961 |
| 3 | 188856214 | 190226607 | 1350 | 3.80E-05 | 3.39E-09 | 5.83E-05 | 0.65164 | 0.51464 | 0.99961 |
| 3 | 190226607 | 192343814 | 1553 | 4.51E-07 | 3.20E-09 | 5.66E-05 | 0.007978 | 0.99363 | 0.99961 |
| 3 | 192343814 | 194059671 | 709 | -1.16E-05 | 3.22E-09 | 5.67E-05 | -0.20462 | 0.83787 | 0.99961 |
| 3 | 194059671 | 194786674 | 386 | 3.19E-05 | 2.15E-09 | 4.64E-05 | 0.68804 | 0.49143 | 0.99961 |
| 3 | 194786674 | 195971408 | 317 | 1.76E-05 | 1.80E-09 | 4.25E-05 | 0.41405 | 0.67884 | 0.99961 |
| 3 | 195971408 | 197075987 | 380 | 1.22E-05 | 2.41E-09 | 4.91E-05 | 0.24922 | 0.80319 | 0.99961 |
| 3 | 197075987 | 197946622 | 328 | 1.11E-05 | 1.87E-09 | 4.32E-05 | 0.25703 | 0.79716 | 0.99961 |
| 4 | 10240 | 694715 | 189 | 4.74E-06 | 1.03E-09 | 3.21E-05 | 0.14753 | 0.88272 | 0.99961 |
| 4 | 694715 | 1478711 | 407 | -1.68E-05 | 3.41E-09 | 5.84E-05 | -0.28844 | 0.77301 | 0.99961 |
| 4 | 1478711 | 2842979 | 464 | -1.71E-05 | 2.67E-09 | 5.17E-05 | -0.33047 | 0.74105 | 0.99961 |
| 4 | 2842979 | 3846040 | 478 | -5.16E-05 | 2.89E-09 | 5.38E-05 | -0.95999 | 0.33706 | 0.99961 |
| 4 | 3846040 | 4684116 | 425 | 7.37E-06 | 2.20E-09 | 4.69E-05 | 0.15734 | 0.87498 | 0.99961 |
| 4 | 4684116 | 5502388 | 678 | 9.75E-06 | 4.41E-09 | 6.64E-05 | 0.14682 | 0.88327 | 0.99961 |
| 4 | 5502388 | 6773043 | 1081 | -4.73E-06 | 2.80E-09 | 5.29E-05 | -0.08935 | 0.92881 | 0.99961 |
| 4 | 6773043 | 7539692 | 644 | -6.06E-05 | 3.05E-09 | 5.53E-05 | -1.0966 | 0.2728 | 0.99961 |
| 4 | 7539692 | 8152235 | 548 | -2.37E-05 | 2.97E-09 | 5.45E-05 | -0.43549 | 0.6632 | 0.99961 |
| 4 | 8152235 | 9326479 | 447 | 3.66E-06 | 2.38E-09 | 4.87E-05 | 0.074985 | 0.94023 | 0.99961 |
| 4 | 9326479 | 10699152 | 1121 | 1.32E-07 | 2.88E-09 | 5.36E-05 | 0.002458 | 0.99804 | 0.99961 |
| 4 | 10699152 | 12323034 | 1356 | -7.34E-06 | 3.16E-09 | 5.62E-05 | -0.13062 | 0.89608 | 0.99961 |
| 4 | 12323034 | 14272001 | 1618 | -1.86E-05 | 3.44E-09 | 5.87E-05 | -0.31723 | 0.75107 | 0.99961 |
| 4 | 14272001 | 15147446 | 714 | 3.36E-06 | 2.99E-09 | 5.47E-05 | 0.061448 | 0.951 | 0.99961 |
| 4 | 15147446 | 15927009 | 482 | 1.51E-05 | 2.31E-09 | 4.80E-05 | 0.31495 | 0.7528 | 0.99961 |
| 4 | 15927009 | 17383322 | 1235 | -1.91E-05 | 2.90E-09 | 5.39E-05 | -0.35463 | 0.72287 | 0.99961 |
| 4 | 17383322 | 18841874 | 925 | 1.05E-05 | 3.22E-09 | 5.68E-05 | 0.18504 | 0.85319 | 0.99961 |
| 4 | 18841874 | 20544557 | 1114 | 5.38E-05 | 2.96E-09 | 5.44E-05 | 0.98851 | 0.3229 | 0.99961 |
| 4 | 20544557 | 22319347 | 1403 | -1.40E-05 | 3.11E-09 | 5.57E-05 | -0.25028 | 0.80237 | 0.99961 |
| 4 | 22319347 | 24135529 | 1257 | 4.26E-06 | 3.15E-09 | 5.61E-05 | 0.076011 | 0.93941 | 0.99961 |
| 4 | 24135529 | 25609322 | 1052 | 3.16E-06 | 2.93E-09 | 5.42E-05 | 0.058281 | 0.95352 | 0.99961 |
| 4 | 25609322 | 27343722 | 997 | 7.12E-05 | 5.32E-09 | 7.30E-05 | 0.97503 | 0.32954 | 0.99961 |
| 4 | 27343722 | 27965868 | 465 | -1.17E-05 | 1.48E-09 | 3.85E-05 | -0.30508 | 0.7603 | 0.99961 |
| 4 | 27965868 | 29762208 | 1375 | 2.80E-05 | 3.33E-09 | 5.77E-05 | 0.48462 | 0.62794 | 0.99961 |
| 4 | 29762208 | 30454535 | 376 | -4.23E-05 | 1.39E-09 | 3.73E-05 | -1.1363 | 0.25583 | 0.99961 |
| 4 | 30454535 | 31782981 | 907 | -1.05E-07 | 3.16E-09 | 5.62E-05 | -0.00187 | 0.99851 | 0.99961 |
| 4 | 31782981 | 33172766 | 805 | 9.45E-06 | 2.71E-09 | 5.21E-05 | 0.1814 | 0.85606 | 0.99961 |
| 4 | 33172766 | 35151415 | 910 | 2.98E-05 | 1.85E-09 | 4.30E-05 | 0.69275 | 0.48847 | 0.99961 |
| 4 | 35151415 | 36020436 | 749 | 1.73E-05 | 1.83E-09 | 4.28E-05 | 0.40419 | 0.68607 | 0.99961 |
| 4 | 36020436 | 37255047 | 1046 | 7.66E-05 | 2.98E-09 | 5.46E-05 | 1.404 | 0.16031 | 0.99961 |
| 4 | 37255047 | 38907022 | 1368 | 1.28E-07 | 3.40E-09 | 5.83E-05 | 0.00219 | 0.99825 | 0.99961 |
| 4 | 38907022 | 40202609 | 675 | -5.48E-05 | 2.82E-09 | 5.31E-05 | -1.0331 | 0.30158 | 0.99961 |
| 4 | 40202609 | 42213058 | 1267 | 3.65E-05 | 3.37E-09 | 5.80E-05 | 0.62969 | 0.5289 | 0.99961 |
| 4 | 42213058 | 43965045 | 1150 | 8.33E-06 | 3.16E-09 | 5.62E-05 | 0.14817 | 0.88221 | 0.99961 |
| 4 | 43965045 | 45189157 | 888 | 2.52E-06 | 2.26E-09 | 4.75E-05 | 0.052982 | 0.95775 | 0.99961 |
| 4 | 45189157 | 47411896 | 1110 | 2.96E-06 | 2.92E-09 | 5.41E-05 | 0.054818 | 0.95628 | 0.99961 |
| 4 | 47411896 | 48123600 | 466 | -1.10E-05 | 1.53E-09 | 3.91E-05 | -0.28213 | 0.77784 | 0.99961 |
| 4 | 48123600 | 53877433 | 889 | -4.24E-05 | 2.73E-09 | 5.23E-05 | -0.8111 | 0.41731 | 0.99961 |
| 4 | 53877433 | 55429556 | 991 | -4.41E-06 | 3.43E-09 | 5.86E-05 | -0.07528 | 0.93999 | 0.99961 |
| 4 | 55429556 | 56547644 | 726 | 1.03E-05 | 4.23E-09 | 6.51E-05 | 0.15857 | 0.874 | 0.99961 |
| 4 | 56547644 | 58935008 | 1580 | -3.90E-05 | 3.41E-09 | 5.84E-05 | -0.66778 | 0.50428 | 0.99961 |
| 4 | 58935008 | 60741087 | 1193 | 1.66E-05 | 3.26E-09 | 5.71E-05 | 0.29072 | 0.77127 | 0.99961 |
| 4 | 60741087 | 62371763 | 1051 | -6.85E-05 | 3.23E-09 | 5.68E-05 | -1.206 | 0.2278 | 0.99961 |
| 4 | 62371763 | 64341001 | 1033 | 2.15E-05 | 3.09E-09 | 5.56E-05 | 0.3872 | 0.69861 | 0.99961 |
| 4 | 64341001 | 66600492 | 1255 | -1.53E-05 | 3.05E-09 | 5.52E-05 | -0.27651 | 0.78216 | 0.99961 |
| 4 | 66600492 | 68059497 | 951 | 1.60E-05 | 2.51E-09 | 5.01E-05 | 0.31882 | 0.74986 | 0.99961 |
| 4 | 68059497 | 68854765 | 517 | -8.97E-06 | 1.62E-09 | 4.03E-05 | -0.22251 | 0.82392 | 0.99961 |
| 4 | 68854765 | 71049152 | 1136 | -5.59E-05 | 2.56E-09 | 5.06E-05 | -1.1056 | 0.2689 | 0.99961 |
| 4 | 71049152 | 72140558 | 495 | -8.58E-07 | 1.67E-09 | 4.08E-05 | -0.02103 | 0.98322 | 0.99961 |
| 4 | 72140558 | 74592390 | 1017 | -1.14E-05 | 2.90E-09 | 5.38E-05 | -0.21209 | 0.83203 | 0.99961 |
| 4 | 74592390 | 77130707 | 1462 | -6.11E-05 | 3.20E-09 | 5.66E-05 | -1.0805 | 0.27992 | 0.99961 |
| 4 | 77130707 | 79093979 | 1331 | 1.76E-05 | 3.45E-09 | 5.87E-05 | 0.29978 | 0.76434 | 0.99961 |
| 4 | 79093979 | 80990297 | 1178 | 1.28E-05 | 3.12E-09 | 5.58E-05 | 0.22926 | 0.81867 | 0.99961 |
| 4 | 80990297 | 82125315 | 493 | -1.11E-05 | 2.07E-09 | 4.55E-05 | -0.24493 | 0.80651 | 0.99961 |
| 4 | 82125315 | 83372593 | 732 | -8.89E-06 | 2.57E-09 | 5.07E-05 | -0.17549 | 0.8607 | 0.99961 |
| 4 | 83372593 | 84799656 | 891 | 6.33E-05 | 3.38E-09 | 5.82E-05 | 1.089 | 0.27617 | 0.99961 |
| 4 | 84799656 | 86930609 | 1239 | 8.56E-06 | 3.44E-09 | 5.86E-05 | 0.14604 | 0.88389 | 0.99961 |
| 4 | 86930609 | 87534648 | 279 | -3.79E-05 | 9.04E-10 | 3.01E-05 | -1.2621 | 0.2069 | 0.99961 |
| 4 | 87534648 | 89238028 | 1069 | -5.56E-05 | 3.66E-09 | 6.05E-05 | -0.91976 | 0.3577 | 0.99961 |
| 4 | 89238028 | 90231564 | 609 | 0.00014997 | 6.73E-09 | 8.20E-05 | 1.8279 | 0.067566 | 0.99961 |
| 4 | 90231564 | 91560677 | 779 | 4.72E-05 | 2.60E-09 | 5.10E-05 | 0.9255 | 0.35471 | 0.99961 |
| 4 | 91560677 | 94233299 | 1206 | -2.14E-05 | 3.27E-09 | 5.72E-05 | -0.3745 | 0.70803 | 0.99961 |
| 4 | 94233299 | 96182188 | 1279 | 3.07E-06 | 3.11E-09 | 5.58E-05 | 0.05495 | 0.95618 | 0.99961 |
| 4 | 96182188 | 97540703 | 1148 | -4.23E-06 | 3.43E-09 | 5.85E-05 | -0.07224 | 0.94241 | 0.99961 |
| 4 | 97540703 | 99424067 | 1009 | 2.30E-05 | 2.75E-09 | 5.24E-05 | 0.43921 | 0.66051 | 0.99961 |
| 4 | 99424067 | 100678360 | 800 | 1.30E-05 | 3.09E-09 | 5.56E-05 | 0.23303 | 0.81574 | 0.99961 |
| 4 | 100678360 | 103221356 | 1627 | -4.52E-05 | 3.80E-09 | 6.16E-05 | -0.73309 | 0.4635 | 0.99961 |
| 4 | 103221356 | 105305294 | 1070 | 4.44E-07 | 3.08E-09 | 5.55E-05 | 0.007991 | 0.99362 | 0.99961 |
| 4 | 105305294 | 107501305 | 1163 | -5.59E-05 | 4.27E-09 | 6.54E-05 | -0.85524 | 0.39242 | 0.99961 |
| 4 | 107501305 | 108815348 | 748 | 3.92E-05 | 2.25E-09 | 4.75E-05 | 0.82702 | 0.40822 | 0.99961 |
| 4 | 108815348 | 111256567 | 1460 | 3.93E-05 | 3.05E-09 | 5.52E-05 | 0.71125 | 0.47693 | 0.99961 |
| 4 | 111256567 | 113870102 | 1417 | 3.13E-05 | 3.16E-09 | 5.62E-05 | 0.55614 | 0.57811 | 0.99961 |
| 4 | 113870102 | 115666246 | 1134 | 8.61E-06 | 3.39E-09 | 5.82E-05 | 0.14785 | 0.88246 | 0.99961 |
| 4 | 115666246 | 117276929 | 875 | 6.73E-06 | 2.12E-09 | 4.61E-05 | 0.14602 | 0.8839 | 0.99961 |
| 4 | 117276929 | 118479918 | 1015 | 2.80E-06 | 2.29E-09 | 4.79E-05 | 0.058475 | 0.95337 | 0.99961 |
| 4 | 118479918 | 119933512 | 1087 | -4.31E-06 | 2.77E-09 | 5.26E-05 | -0.08199 | 0.93465 | 0.99961 |
| 4 | 119933512 | 120392684 | 297 | -1.45E-05 | 1.58E-09 | 3.98E-05 | -0.36363 | 0.71614 | 0.99961 |
| 4 | 120392684 | 122657987 | 1559 | -9.05E-07 | 3.91E-09 | 6.25E-05 | -0.01447 | 0.98845 | 0.99961 |
| 4 | 122657987 | 124286481 | 862 | 3.83E-06 | 3.93E-09 | 6.27E-05 | 0.061053 | 0.95132 | 0.99961 |
| 4 | 124286481 | 125210862 | 506 | -1.84E-05 | 3.00E-09 | 5.48E-05 | -0.33677 | 0.73629 | 0.99961 |
| 4 | 125210862 | 126480886 | 844 | -2.77E-06 | 3.29E-09 | 5.73E-05 | -0.04836 | 0.96143 | 0.99961 |
| 4 | 126480886 | 127275477 | 565 | 3.83E-05 | 1.94E-09 | 4.40E-05 | 0.86998 | 0.38431 | 0.99961 |
| 4 | 127275477 | 127830293 | 420 | -1.70E-05 | 1.29E-09 | 3.59E-05 | -0.47363 | 0.63577 | 0.99961 |
| 4 | 127830293 | 128996665 | 443 | -1.04E-05 | 1.39E-09 | 3.73E-05 | -0.27899 | 0.78026 | 0.99961 |
| 4 | 128996665 | 130591885 | 917 | 1.23E-05 | 3.25E-09 | 5.70E-05 | 0.21519 | 0.82962 | 0.99961 |
| 4 | 130591885 | 131283821 | 450 | -2.33E-05 | 1.43E-09 | 3.79E-05 | -0.61454 | 0.53886 | 0.99961 |
| 4 | 131283821 | 132954033 | 923 | -1.51E-05 | 2.69E-09 | 5.18E-05 | -0.29103 | 0.77103 | 0.99961 |
| 4 | 132954033 | 134628434 | 1129 | 1.30E-05 | 2.62E-09 | 5.12E-05 | 0.25319 | 0.80012 | 0.99961 |
| 4 | 134628434 | 136566407 | 1151 | 2.68E-05 | 2.62E-09 | 5.12E-05 | 0.52254 | 0.60129 | 0.99961 |
| 4 | 136566407 | 138541971 | 1251 | -2.32E-05 | 3.05E-09 | 5.52E-05 | -0.42001 | 0.67448 | 0.99961 |
| 4 | 138541971 | 139556511 | 717 | -3.30E-05 | 2.86E-09 | 5.34E-05 | -0.6173 | 0.53704 | 0.99961 |
| 4 | 139556511 | 141084631 | 817 | 1.63E-05 | 3.20E-09 | 5.66E-05 | 0.28826 | 0.77315 | 0.99961 |
| 4 | 141084631 | 143443265 | 1479 | -4.95E-06 | 3.58E-09 | 5.99E-05 | -0.08262 | 0.93415 | 0.99961 |
| 4 | 143443265 | 146178187 | 1151 | 2.01E-05 | 4.02E-09 | 6.34E-05 | 0.31738 | 0.75096 | 0.99961 |
| 4 | 146178187 | 148521873 | 1189 | -1.52E-05 | 3.57E-09 | 5.98E-05 | -0.25508 | 0.79866 | 0.99961 |
| 4 | 148521873 | 150683580 | 1121 | -3.15E-05 | 3.49E-09 | 5.91E-05 | -0.53356 | 0.59365 | 0.99961 |
| 4 | 150683580 | 152782184 | 907 | 7.74E-06 | 2.49E-09 | 4.99E-05 | 0.15532 | 0.87657 | 0.99961 |
| 4 | 152782184 | 154477641 | 843 | 2.62E-05 | 3.39E-09 | 5.82E-05 | 0.44956 | 0.65303 | 0.99961 |
| 4 | 154477641 | 155056126 | 391 | -1.06E-05 | 1.65E-09 | 4.06E-05 | -0.26047 | 0.7945 | 0.99961 |
| 4 | 155056126 | 157485097 | 1628 | -1.40E-05 | 3.27E-09 | 5.72E-05 | -0.24421 | 0.80707 | 0.99961 |
| 4 | 157485097 | 158743718 | 665 | 1.64E-05 | 2.41E-09 | 4.91E-05 | 0.33425 | 0.73819 | 0.99961 |
| 4 | 158743718 | 161058864 | 1103 | 1.67E-05 | 3.34E-09 | 5.78E-05 | 0.28943 | 0.77225 | 0.99961 |
| 4 | 161058864 | 161826435 | 593 | 2.56E-06 | 2.04E-09 | 4.52E-05 | 0.056789 | 0.95471 | 0.99961 |
| 4 | 161826435 | 163243679 | 1039 | 8.54E-06 | 2.85E-09 | 5.34E-05 | 0.15989 | 0.87297 | 0.99961 |
| 4 | 163243679 | 164478234 | 733 | 1.72E-05 | 2.29E-09 | 4.79E-05 | 0.36015 | 0.71873 | 0.99961 |
| 4 | 164478234 | 165823448 | 944 | 1.24E-05 | 3.22E-09 | 5.67E-05 | 0.21771 | 0.82766 | 0.99961 |
| 4 | 165823448 | 167018858 | 712 | -2.70E-05 | 3.11E-09 | 5.58E-05 | -0.48394 | 0.62843 | 0.99961 |
| 4 | 167018858 | 167852917 | 486 | -5.25E-06 | 1.25E-09 | 3.54E-05 | -0.14848 | 0.88196 | 0.99961 |
| 4 | 167852917 | 169676825 | 1202 | -7.47E-06 | 3.35E-09 | 5.79E-05 | -0.12898 | 0.89737 | 0.99961 |
| 4 | 169676825 | 170776510 | 524 | -2.11E-05 | 2.25E-09 | 4.75E-05 | -0.44413 | 0.65695 | 0.99961 |
| 4 | 170776510 | 172558635 | 1027 | -5.36E-05 | 3.03E-09 | 5.51E-05 | -0.97366 | 0.33023 | 0.99961 |
| 4 | 172558635 | 174264132 | 911 | -3.78E-05 | 2.70E-09 | 5.20E-05 | -0.7273 | 0.46704 | 0.99961 |
| 4 | 174264132 | 176570716 | 1335 | 1.78E-05 | 3.34E-09 | 5.78E-05 | 0.30831 | 0.75785 | 0.99961 |
| 4 | 176570716 | 178991910 | 1715 | 3.48E-05 | 3.44E-09 | 5.87E-05 | 0.59309 | 0.55312 | 0.99961 |
| 4 | 178991910 | 180122043 | 758 | -5.24E-06 | 3.27E-09 | 5.71E-05 | -0.09165 | 0.92698 | 0.99961 |
| 4 | 180122043 | 182066807 | 1635 | -2.14E-06 | 3.08E-09 | 5.55E-05 | -0.0386 | 0.96921 | 0.99961 |
| 4 | 182066807 | 183371275 | 1290 | 3.70E-05 | 3.14E-09 | 5.60E-05 | 0.66131 | 0.50842 | 0.99961 |
| 4 | 183371275 | 184929455 | 996 | -3.41E-05 | 3.17E-09 | 5.63E-05 | -0.60661 | 0.54411 | 0.99961 |
| 4 | 184929455 | 186909090 | 1425 | -2.44E-05 | 3.14E-09 | 5.61E-05 | -0.43476 | 0.66374 | 0.99961 |
| 4 | 186909090 | 188472981 | 1089 | 2.84E-05 | 3.37E-09 | 5.80E-05 | 0.48929 | 0.62463 | 0.99961 |
| 4 | 188472981 | 189693511 | 787 | -8.43E-06 | 3.03E-09 | 5.51E-05 | -0.15313 | 0.87829 | 0.99961 |
| 4 | 189693511 | 191043594 | 592 | -9.42E-06 | 2.62E-09 | 5.12E-05 | -0.18407 | 0.85396 | 0.99961 |
| 5 | 11940 | 982252 | 397 | 1.72E-05 | 1.69E-09 | 4.11E-05 | 0.41771 | 0.67616 | 0.99961 |
| 5 | 982252 | 2132442 | 848 | 2.16E-05 | 3.37E-09 | 5.80E-05 | 0.37234 | 0.70964 | 0.99961 |
| 5 | 2132442 | 3361941 | 1593 | -1.40E-06 | 3.12E-09 | 5.59E-05 | -0.02498 | 0.98007 | 0.99961 |
| 5 | 3361941 | 4632110 | 1104 | 7.79E-05 | 4.83E-09 | 6.95E-05 | 1.1217 | 0.26201 | 0.99961 |
| 5 | 4632110 | 5973233 | 1434 | 6.79E-06 | 3.12E-09 | 5.58E-05 | 0.12165 | 0.90317 | 0.99961 |
| 5 | 5973233 | 7143218 | 1135 | -5.21E-06 | 3.25E-09 | 5.70E-05 | -0.09135 | 0.92722 | 0.99961 |
| 5 | 7143218 | 8446336 | 1201 | -8.32E-06 | 3.23E-09 | 5.69E-05 | -0.14636 | 0.88364 | 0.99961 |
| 5 | 8446336 | 9718832 | 1265 | 4.13E-05 | 3.61E-09 | 6.01E-05 | 0.68794 | 0.49149 | 0.99961 |
| 5 | 9718832 | 11173271 | 1358 | -7.29E-06 | 2.90E-09 | 5.39E-05 | -0.13535 | 0.89233 | 0.99961 |
| 5 | 11173271 | 13464832 | 1165 | -4.54E-07 | 3.32E-09 | 5.76E-05 | -0.00788 | 0.99372 | 0.99961 |
| 5 | 13464832 | 14757804 | 1101 | -3.04E-05 | 3.18E-09 | 5.64E-05 | -0.53877 | 0.59005 | 0.99961 |
| 5 | 14757804 | 16963613 | 1374 | 4.14E-05 | 3.39E-09 | 5.82E-05 | 0.71048 | 0.4774 | 0.99961 |
| 5 | 16963613 | 18051592 | 553 | 5.38E-05 | 2.42E-09 | 4.92E-05 | 1.0948 | 0.27362 | 0.99961 |
| 5 | 18051592 | 19917716 | 1295 | 4.30E-06 | 3.34E-09 | 5.78E-05 | 0.074531 | 0.94059 | 0.99961 |
| 5 | 19917716 | 22152073 | 1044 | 1.07E-06 | 3.04E-09 | 5.52E-05 | 0.019452 | 0.98448 | 0.99961 |
| 5 | 22152073 | 23494324 | 924 | 1.40E-06 | 2.88E-09 | 5.37E-05 | 0.026107 | 0.97917 | 0.99961 |
| 5 | 23494324 | 26184851 | 1809 | -5.12E-05 | 3.16E-09 | 5.62E-05 | -0.91153 | 0.36201 | 0.99961 |
| 5 | 26184851 | 26857604 | 415 | -6.64E-06 | 1.42E-09 | 3.77E-05 | -0.17633 | 0.86004 | 0.99961 |
| 5 | 26857604 | 27984513 | 579 | -1.25E-05 | 2.03E-09 | 4.51E-05 | -0.2775 | 0.7814 | 0.99961 |
| 5 | 27984513 | 29714820 | 1162 | -3.16E-05 | 3.40E-09 | 5.83E-05 | -0.54267 | 0.58736 | 0.99961 |
| 5 | 29714820 | 31390249 | 1389 | -1.30E-05 | 3.52E-09 | 5.93E-05 | -0.21866 | 0.82692 | 0.99961 |
| 5 | 31390249 | 33500180 | 1363 | 3.19E-05 | 3.92E-09 | 6.26E-05 | 0.51042 | 0.60976 | 0.99961 |
| 5 | 33500180 | 35048970 | 1006 | -3.89E-05 | 3.89E-09 | 6.24E-05 | -0.62387 | 0.53271 | 0.99961 |
| 5 | 35048970 | 36433954 | 1007 | 2.77E-05 | 3.46E-09 | 5.88E-05 | 0.47124 | 0.63747 | 0.99961 |
| 5 | 36433954 | 38802410 | 1505 | -1.87E-05 | 3.61E-09 | 6.01E-05 | -0.31101 | 0.75579 | 0.99961 |
| 5 | 38802410 | 40287729 | 1136 | -3.93E-06 | 3.73E-09 | 6.11E-05 | -0.06431 | 0.94872 | 0.99961 |
| 5 | 40287729 | 41888710 | 1239 | 6.10E-06 | 2.65E-09 | 5.15E-05 | 0.11842 | 0.90574 | 0.99961 |
| 5 | 41888710 | 43983499 | 901 | -2.51E-05 | 2.53E-09 | 5.03E-05 | -0.49919 | 0.61765 | 0.99961 |
| 5 | 43983499 | 50163398 | 964 | -7.22E-06 | 2.32E-09 | 4.81E-05 | -0.14992 | 0.88083 | 0.99961 |
| 5 | 50163398 | 52134622 | 1338 | -3.80E-05 | 3.31E-09 | 5.76E-05 | -0.66047 | 0.50895 | 0.99961 |
| 5 | 52134622 | 53703044 | 1298 | -1.85E-05 | 3.53E-09 | 5.94E-05 | -0.31195 | 0.75508 | 0.99961 |
| 5 | 53703044 | 55417349 | 1124 | 8.54E-05 | 4.37E-09 | 6.61E-05 | 1.2919 | 0.19639 | 0.99961 |
| 5 | 55417349 | 56621102 | 970 | 9.86E-05 | 7.24E-09 | 8.51E-05 | 1.1586 | 0.2466 | 0.99961 |
| 5 | 56621102 | 57340073 | 585 | 1.53E-06 | 1.93E-09 | 4.40E-05 | 0.034844 | 0.9722 | 0.99961 |
| 5 | 57340073 | 58524622 | 1149 | 2.38E-05 | 3.27E-09 | 5.72E-05 | 0.41628 | 0.6772 | 0.99961 |
| 5 | 58524622 | 60935907 | 1425 | 3.15E-05 | 3.72E-09 | 6.10E-05 | 0.5161 | 0.60578 | 0.99961 |
| 5 | 60935907 | 61672995 | 528 | 2.26E-05 | 1.91E-09 | 4.38E-05 | 0.51696 | 0.60518 | 0.99961 |
| 5 | 61672995 | 63218987 | 1047 | -3.24E-05 | 2.84E-09 | 5.33E-05 | -0.60818 | 0.54307 | 0.99961 |
| 5 | 63218987 | 63968304 | 353 | -3.37E-05 | 1.31E-09 | 3.62E-05 | -0.93221 | 0.35123 | 0.99961 |
| 5 | 63968304 | 65911286 | 1226 | 6.54E-05 | 3.87E-09 | 6.22E-05 | 1.0507 | 0.29338 | 0.99961 |
| 5 | 65911286 | 67850860 | 1470 | -2.93E-05 | 4.04E-09 | 6.36E-05 | -0.46134 | 0.64455 | 0.99961 |
| 5 | 67850860 | 71240456 | 871 | 4.74E-06 | 3.12E-09 | 5.59E-05 | 0.084793 | 0.93243 | 0.99961 |
| 5 | 71240456 | 73759326 | 1806 | 1.13E-05 | 3.49E-09 | 5.90E-05 | 0.19064 | 0.84881 | 0.99961 |
| 5 | 73759326 | 75798866 | 1282 | 3.82E-05 | 3.29E-09 | 5.74E-05 | 0.66511 | 0.50598 | 0.99961 |
| 5 | 75798866 | 77623332 | 1314 | -3.11E-05 | 3.38E-09 | 5.82E-05 | -0.53389 | 0.59342 | 0.99961 |
| 5 | 77623332 | 79393144 | 1149 | 1.96E-05 | 3.08E-09 | 5.55E-05 | 0.35319 | 0.72395 | 0.99961 |
| 5 | 79393144 | 80481471 | 851 | -2.62E-05 | 3.10E-09 | 5.57E-05 | -0.47058 | 0.63794 | 0.99961 |
| 5 | 80481471 | 82025648 | 888 | 3.99E-06 | 3.49E-09 | 5.91E-05 | 0.067498 | 0.94619 | 0.99961 |
| 5 | 82025648 | 83972689 | 1030 | 6.93E-06 | 3.31E-09 | 5.75E-05 | 0.12036 | 0.90419 | 0.99961 |
| 5 | 83972689 | 85767290 | 1384 | 1.75E-05 | 3.17E-09 | 5.63E-05 | 0.31101 | 0.75579 | 0.99961 |
| 5 | 85767290 | 87389991 | 640 | -6.25E-05 | 2.47E-09 | 4.97E-05 | -1.2578 | 0.20846 | 0.99961 |
| 5 | 87389991 | 88891530 | 587 | 1.23E-05 | 2.41E-09 | 4.91E-05 | 0.25099 | 0.80182 | 0.99961 |
| 5 | 88891530 | 90422654 | 850 | -5.29E-07 | 2.89E-09 | 5.37E-05 | -0.00984 | 0.99215 | 0.99961 |
| 5 | 90422654 | 91840542 | 659 | -2.05E-05 | 2.52E-09 | 5.02E-05 | -0.40922 | 0.68237 | 0.99961 |
| 5 | 91840542 | 93809984 | 786 | -3.93E-05 | 2.76E-09 | 5.26E-05 | -0.7469 | 0.45513 | 0.99961 |
| 5 | 93809984 | 95963519 | 1298 | -4.61E-06 | 3.40E-09 | 5.83E-05 | -0.07902 | 0.93702 | 0.99961 |
| 5 | 95963519 | 97315601 | 922 | 1.10E-05 | 2.59E-09 | 5.09E-05 | 0.21709 | 0.82814 | 0.99961 |
| 5 | 97315601 | 99260992 | 1135 | 3.68E-05 | 3.25E-09 | 5.70E-05 | 0.64639 | 0.51803 | 0.99961 |
| 5 | 99260992 | 101578769 | 1302 | 4.22E-06 | 3.40E-09 | 5.83E-05 | 0.072435 | 0.94226 | 0.99961 |
| 5 | 101578769 | 103320005 | 1062 | -2.29E-05 | 4.20E-09 | 6.48E-05 | -0.35336 | 0.72382 | 0.99961 |
| 5 | 103320005 | 104851483 | 1026 | -1.55E-05 | 2.78E-09 | 5.27E-05 | -0.29417 | 0.76863 | 0.99961 |
| 5 | 104851483 | 105893055 | 400 | 4.47E-05 | 1.77E-09 | 4.21E-05 | 1.0617 | 0.28839 | 0.99961 |
| 5 | 105893055 | 107264196 | 1154 | -7.92E-05 | 3.49E-09 | 5.90E-05 | -1.3422 | 0.17954 | 0.99961 |
| 5 | 107264196 | 108633934 | 890 | 6.98E-06 | 2.96E-09 | 5.44E-05 | 0.12817 | 0.89801 | 0.99961 |
| 5 | 108633934 | 110821144 | 1470 | 1.15E-05 | 3.22E-09 | 5.67E-05 | 0.20224 | 0.83973 | 0.99961 |
| 5 | 110821144 | 111981562 | 860 | -7.73E-06 | 3.41E-09 | 5.84E-05 | -0.13245 | 0.89463 | 0.99961 |
| 5 | 111981562 | 113396285 | 1170 | 7.09E-05 | 3.79E-09 | 6.15E-05 | 1.1518 | 0.2494 | 0.99961 |
| 5 | 113396285 | 114830370 | 1094 | -2.78E-05 | 3.39E-09 | 5.82E-05 | -0.47798 | 0.63266 | 0.99961 |
| 5 | 114830370 | 115831047 | 888 | -3.47E-06 | 3.30E-09 | 5.75E-05 | -0.0604 | 0.95183 | 0.99961 |
| 5 | 115831047 | 117346213 | 1323 | 1.25E-05 | 3.09E-09 | 5.56E-05 | 0.22504 | 0.82195 | 0.99961 |
| 5 | 117346213 | 118608820 | 793 | 4.01E-05 | 3.61E-09 | 6.01E-05 | 0.66789 | 0.5042 | 0.99961 |
| 5 | 118608820 | 119669042 | 713 | -3.40E-05 | 4.91E-09 | 7.00E-05 | -0.48573 | 0.62716 | 0.99961 |
| 5 | 119669042 | 120452166 | 565 | -3.18E-05 | 2.39E-09 | 4.89E-05 | -0.65045 | 0.5154 | 0.99961 |
| 5 | 120452166 | 122556905 | 1412 | -5.75E-05 | 3.50E-09 | 5.92E-05 | -0.97133 | 0.33138 | 0.99961 |
| 5 | 122556905 | 123798100 | 988 | 1.53E-05 | 3.05E-09 | 5.52E-05 | 0.27622 | 0.78238 | 0.99961 |
| 5 | 123798100 | 125785649 | 1827 | 3.04E-05 | 3.53E-09 | 5.94E-05 | 0.51079 | 0.6095 | 0.99961 |
| 5 | 125785649 | 127344604 | 815 | 2.16E-06 | 3.17E-09 | 5.63E-05 | 0.038266 | 0.96948 | 0.99961 |
| 5 | 127344604 | 129519025 | 1538 | -2.39E-05 | 3.72E-09 | 6.10E-05 | -0.39261 | 0.69461 | 0.99961 |
| 5 | 129519025 | 132139649 | 1135 | 3.82E-05 | 3.49E-09 | 5.91E-05 | 0.64676 | 0.51779 | 0.99961 |
| 5 | 132139649 | 134777401 | 1515 | 5.42E-05 | 4.28E-09 | 6.54E-05 | 0.82937 | 0.4069 | 0.99961 |
| 5 | 134777401 | 136376050 | 1204 | 4.78E-06 | 3.30E-09 | 5.75E-05 | 0.083098 | 0.93377 | 0.99961 |
| 5 | 136376050 | 139265072 | 1208 | -0.00010564 | 3.68E-09 | 6.07E-05 | -1.7409 | 0.08171 | 0.99961 |
| 5 | 139265072 | 140645971 | 578 | -5.62E-05 | 2.66E-09 | 5.16E-05 | -1.089 | 0.27616 | 0.99961 |
| 5 | 140645971 | 142981248 | 1522 | 2.40E-05 | 4.99E-09 | 7.06E-05 | 0.33959 | 0.73417 | 0.99961 |
| 5 | 142981248 | 144943354 | 1418 | 2.27E-05 | 3.43E-09 | 5.86E-05 | 0.38792 | 0.69808 | 0.99961 |
| 5 | 144943354 | 147181998 | 1498 | 7.96E-05 | 3.65E-09 | 6.04E-05 | 1.3172 | 0.18779 | 0.99961 |
| 5 | 147181998 | 148662624 | 1157 | -3.70E-05 | 3.66E-09 | 6.05E-05 | -0.6118 | 0.54067 | 0.99961 |
| 5 | 148662624 | 150561298 | 1438 | -6.54E-05 | 3.90E-09 | 6.24E-05 | -1.0486 | 0.29435 | 0.99961 |
| 5 | 150561298 | 152867774 | 1796 | 4.42E-05 | 3.30E-09 | 5.74E-05 | 0.76966 | 0.4415 | 0.99961 |
| 5 | 152867774 | 153773088 | 945 | -5.26E-05 | 2.49E-09 | 4.99E-05 | -1.055 | 0.29144 | 0.99961 |
| 5 | 153773088 | 155373505 | 1179 | -4.38E-05 | 3.12E-09 | 5.59E-05 | -0.78311 | 0.43356 | 0.99961 |
| 5 | 155373505 | 156628700 | 784 | 2.91E-05 | 2.53E-09 | 5.03E-05 | 0.57827 | 0.56308 | 0.99961 |
| 5 | 156628700 | 158825698 | 1755 | 8.09E-06 | 3.54E-09 | 5.95E-05 | 0.13597 | 0.89185 | 0.99961 |
| 5 | 158825698 | 159973944 | 721 | -2.40E-06 | 3.24E-09 | 5.69E-05 | -0.04222 | 0.96632 | 0.99961 |
| 5 | 159973944 | 161482133 | 1089 | -1.42E-06 | 2.93E-09 | 5.41E-05 | -0.0263 | 0.97902 | 0.99961 |
| 5 | 161482133 | 162743908 | 869 | 3.40E-06 | 2.85E-09 | 5.34E-05 | 0.063804 | 0.94913 | 0.99961 |
| 5 | 162743908 | 163771582 | 839 | 4.14E-05 | 2.76E-09 | 5.26E-05 | 0.78753 | 0.43097 | 0.99961 |
| 5 | 163771582 | 165642395 | 1197 | 1.64E-05 | 3.04E-09 | 5.51E-05 | 0.29843 | 0.76538 | 0.99961 |
| 5 | 165642395 | 166847740 | 874 | -1.78E-05 | 3.26E-09 | 5.71E-05 | -0.31267 | 0.75453 | 0.99961 |
| 5 | 166847740 | 168525318 | 1298 | 1.33E-05 | 3.15E-09 | 5.62E-05 | 0.23662 | 0.81295 | 0.99961 |
| 5 | 168525318 | 169505664 | 869 | -8.60E-06 | 3.23E-09 | 5.68E-05 | -0.15138 | 0.87968 | 0.99961 |
| 5 | 169505664 | 171074292 | 1214 | 1.43E-05 | 3.28E-09 | 5.73E-05 | 0.25018 | 0.80245 | 0.99961 |
| 5 | 171074292 | 172678327 | 1132 | -2.10E-05 | 4.63E-09 | 6.80E-05 | -0.30933 | 0.75707 | 0.99961 |
| 5 | 172678327 | 173642871 | 920 | -0.00010678 | 5.72E-09 | 7.57E-05 | -1.4114 | 0.15813 | 0.99961 |
| 5 | 173642871 | 175450595 | 1480 | -3.52E-05 | 3.33E-09 | 5.77E-05 | -0.61009 | 0.54181 | 0.99961 |
| 5 | 175450595 | 177251507 | 530 | -6.24E-05 | 2.76E-09 | 5.25E-05 | -1.1887 | 0.23457 | 0.99961 |
| 5 | 177251507 | 178413464 | 781 | 2.13E-06 | 3.25E-09 | 5.70E-05 | 0.037288 | 0.97026 | 0.99961 |
| 5 | 178413464 | 179401244 | 622 | -1.15E-06 | 3.65E-09 | 6.04E-05 | -0.01896 | 0.98487 | 0.99961 |
| 5 | 179401244 | 180559933 | 584 | -1.14E-05 | 2.78E-09 | 5.27E-05 | -0.21577 | 0.82917 | 0.99961 |
| 5 | 180559933 | 180885156 | 80 | -4.25E-06 | 7.56E-10 | 2.75E-05 | -0.15438 | 0.87731 | 0.99961 |
| 6 | 73924 | 1452362 | 1247 | 1.25E-05 | 2.92E-09 | 5.41E-05 | 0.23047 | 0.81773 | 0.99961 |
| 6 | 1452362 | 2458936 | 815 | -1.59E-05 | 3.77E-09 | 6.14E-05 | -0.25841 | 0.79609 | 0.99961 |
| 6 | 2458936 | 3573593 | 1010 | 1.22E-07 | 3.43E-09 | 5.86E-05 | 0.002086 | 0.99834 | 0.99961 |
| 6 | 3573593 | 4617817 | 1073 | -4.47E-05 | 3.64E-09 | 6.03E-05 | -0.74163 | 0.45831 | 0.99961 |
| 6 | 4617817 | 5791472 | 1106 | 2.41E-05 | 3.29E-09 | 5.73E-05 | 0.42106 | 0.67371 | 0.99961 |
| 6 | 5791472 | 6785207 | 1072 | -8.94E-05 | 6.11E-09 | 7.81E-05 | -1.1447 | 0.25234 | 0.99961 |
| 6 | 6785207 | 7808936 | 932 | -6.35E-05 | 4.00E-09 | 6.33E-05 | -1.0039 | 0.31541 | 0.99961 |
| 6 | 7808936 | 9020026 | 1193 | -8.23E-06 | 3.30E-09 | 5.75E-05 | -0.14326 | 0.88609 | 0.99961 |
| 6 | 9020026 | 10540317 | 1304 | 3.58E-05 | 3.36E-09 | 5.80E-05 | 0.61657 | 0.53752 | 0.99961 |
| 6 | 10540317 | 11790816 | 1027 | -5.14E-05 | 3.79E-09 | 6.16E-05 | -0.83546 | 0.40346 | 0.99961 |
| 6 | 11790816 | 13209388 | 1208 | 2.69E-05 | 3.20E-09 | 5.66E-05 | 0.47497 | 0.63481 | 0.99961 |
| 6 | 13209388 | 14802924 | 1301 | 6.70E-06 | 3.48E-09 | 5.90E-05 | 0.11357 | 0.90958 | 0.99961 |
| 6 | 14802924 | 17386405 | 1892 | 2.55E-05 | 3.59E-09 | 5.99E-05 | 0.42565 | 0.67036 | 0.99961 |
| 6 | 17386405 | 19207487 | 1422 | -3.83E-05 | 3.19E-09 | 5.65E-05 | -0.67854 | 0.49743 | 0.99961 |
| 6 | 19207487 | 21684065 | 2122 | 0.00010428 | 3.93E-09 | 6.27E-05 | 1.6645 | 0.096022 | 0.99961 |
| 6 | 21684065 | 22748307 | 1069 | -3.06E-08 | 3.87E-09 | 6.22E-05 | -0.00049 | 0.99961 | 0.99961 |
| 6 | 22748307 | 23936619 | 1144 | -5.42E-06 | 3.31E-09 | 5.75E-05 | -0.09415 | 0.92499 | 0.99961 |
| 6 | 23936619 | 24852275 | 734 | -1.44E-06 | 3.33E-09 | 5.77E-05 | -0.02499 | 0.98007 | 0.99961 |
| 6 | 24852275 | 25684587 | 925 | -4.78E-05 | 3.51E-09 | 5.92E-05 | -0.80649 | 0.41996 | 0.99961 |
| 6 | 25684587 | 26791233 | 874 | -1.35E-05 | 2.41E-09 | 4.91E-05 | -0.27427 | 0.78388 | 0.99961 |
| 6 | 26791233 | 28017819 | 545 | -1.44E-05 | 2.17E-09 | 4.66E-05 | -0.30916 | 0.7572 | 0.99961 |
| 6 | 28017819 | 28917608 | 572 | -5.57E-05 | 1.83E-09 | 4.28E-05 | -1.3008 | 0.19332 | 0.99961 |
| 6 | 28917608 | 29737971 | 882 | -5.72E-05 | 2.71E-09 | 5.20E-05 | -1.0994 | 0.27158 | 0.99961 |
| 6 | 29737971 | 30798168 | 1600 | -2.33E-05 | 4.10E-09 | 6.40E-05 | -0.36442 | 0.71555 | 0.99961 |
| 6 | 30798168 | 31571218 | 1530 | -1.24E-05 | 5.84E-09 | 7.64E-05 | -0.1626 | 0.87083 | 0.99961 |
| 6 | 31571218 | 32682664 | 1104 | 5.93E-05 | 5.43E-09 | 7.37E-05 | 0.80475 | 0.42096 | 0.99961 |
| 6 | 32682664 | 33236497 | 1156 | -3.29E-06 | 4.74E-09 | 6.89E-05 | -0.04784 | 0.96184 | 0.99961 |
| 6 | 33236497 | 35455756 | 1295 | 1.56E-05 | 6.23E-09 | 7.89E-05 | 0.19802 | 0.84303 | 0.99961 |
| 6 | 35455756 | 37572596 | 1415 | -1.98E-05 | 4.25E-09 | 6.52E-05 | -0.3038 | 0.76128 | 0.99961 |
| 6 | 37572596 | 39295677 | 1522 | 4.92E-06 | 3.34E-09 | 5.78E-05 | 0.085133 | 0.93216 | 0.99961 |
| 6 | 39295677 | 40345115 | 808 | 2.10E-05 | 2.74E-09 | 5.24E-05 | 0.4008 | 0.68857 | 0.99961 |
| 6 | 40345115 | 42038721 | 1316 | 5.46E-05 | 3.57E-09 | 5.98E-05 | 0.91264 | 0.36143 | 0.99961 |
| 6 | 42038721 | 43756169 | 735 | 9.93E-05 | 4.68E-09 | 6.84E-05 | 1.4516 | 0.1466 | 0.99961 |
| 6 | 43756169 | 45406563 | 1178 | 0.00010701 | 9.01E-09 | 9.49E-05 | 1.1275 | 0.25952 | 0.99961 |
| 6 | 45406563 | 47311898 | 1498 | 1.99E-05 | 3.68E-09 | 6.07E-05 | 0.32743 | 0.74334 | 0.99961 |
| 6 | 47311898 | 48391125 | 891 | 3.14E-05 | 2.20E-09 | 4.69E-05 | 0.66927 | 0.50332 | 0.99961 |
| 6 | 48391125 | 50386145 | 1429 | -9.87E-06 | 2.42E-09 | 4.92E-05 | -0.20055 | 0.84105 | 0.99961 |
| 6 | 50386145 | 52210476 | 1373 | 7.27E-06 | 3.23E-09 | 5.68E-05 | 0.12804 | 0.89812 | 0.99961 |
| 6 | 52210476 | 53279102 | 809 | 1.91E-05 | 2.91E-09 | 5.39E-05 | 0.35387 | 0.72344 | 0.99961 |
| 6 | 53279102 | 55468270 | 2037 | -1.29E-05 | 3.59E-09 | 5.99E-05 | -0.21591 | 0.82906 | 0.99961 |
| 6 | 55468270 | 56105313 | 641 | 9.04E-06 | 1.62E-09 | 4.02E-05 | 0.22479 | 0.82214 | 0.99961 |
| 6 | 56105313 | 57599465 | 687 | -3.76E-05 | 2.50E-09 | 5.00E-05 | -0.75088 | 0.45273 | 0.99961 |
| 6 | 57599465 | 61880512 | 362 | 4.72E-06 | 7.60E-10 | 2.76E-05 | 0.1713 | 0.86399 | 0.99961 |
| 6 | 61880512 | 63552888 | 1051 | 2.21E-05 | 1.75E-09 | 4.18E-05 | 0.52777 | 0.59766 | 0.99961 |
| 6 | 63552888 | 65765742 | 1215 | -2.31E-05 | 2.75E-09 | 5.24E-05 | -0.44053 | 0.65955 | 0.99961 |
| 6 | 65765742 | 67329215 | 1553 | -3.30E-05 | 3.68E-09 | 6.07E-05 | -0.5441 | 0.58637 | 0.99961 |
| 6 | 67329215 | 68849483 | 1346 | 1.21E-05 | 3.39E-09 | 5.83E-05 | 0.20687 | 0.83611 | 0.99961 |
| 6 | 68849483 | 69523448 | 584 | -2.03E-06 | 1.66E-09 | 4.07E-05 | -0.04984 | 0.96025 | 0.99961 |
| 6 | 69523448 | 71609510 | 1695 | 5.15E-05 | 3.45E-09 | 5.87E-05 | 0.87734 | 0.3803 | 0.99961 |
| 6 | 71609510 | 73450097 | 1506 | -6.58E-05 | 3.41E-09 | 5.84E-05 | -1.1275 | 0.25955 | 0.99961 |
| 6 | 73450097 | 75462003 | 1227 | -4.73E-05 | 3.22E-09 | 5.67E-05 | -0.83343 | 0.4046 | 0.99961 |
| 6 | 75462003 | 77414881 | 1032 | -1.99E-05 | 2.70E-09 | 5.19E-05 | -0.38289 | 0.7018 | 0.99961 |
| 6 | 77414881 | 78957728 | 1346 | 2.60E-05 | 2.26E-09 | 4.75E-05 | 0.54794 | 0.58373 | 0.99961 |
| 6 | 78957728 | 80691170 | 1318 | 3.72E-05 | 3.38E-09 | 5.81E-05 | 0.64039 | 0.52192 | 0.99961 |
| 6 | 80691170 | 81929222 | 1060 | 6.45E-05 | 4.39E-09 | 6.63E-05 | 0.97289 | 0.33061 | 0.99961 |
| 6 | 81929222 | 83127149 | 842 | 4.99E-06 | 3.28E-09 | 5.73E-05 | 0.087111 | 0.93058 | 0.99961 |
| 6 | 83127149 | 85209989 | 1192 | -2.27E-05 | 3.90E-09 | 6.24E-05 | -0.36308 | 0.71654 | 0.99961 |
| 6 | 85209989 | 87069500 | 1383 | 2.85E-06 | 3.54E-09 | 5.95E-05 | 0.047881 | 0.96181 | 0.99961 |
| 6 | 87069500 | 88822140 | 1202 | -1.69E-05 | 3.35E-09 | 5.79E-05 | -0.292 | 0.77029 | 0.99961 |
| 6 | 88822140 | 89973052 | 887 | -1.44E-05 | 3.31E-09 | 5.75E-05 | -0.25099 | 0.80182 | 0.99961 |
| 6 | 89973052 | 91843196 | 1478 | 7.27E-05 | 3.33E-09 | 5.77E-05 | 1.2585 | 0.20823 | 0.99961 |
| 6 | 91843196 | 93428644 | 1275 | 1.50E-05 | 3.31E-09 | 5.75E-05 | 0.26065 | 0.79436 | 0.99961 |
| 6 | 93428644 | 94118142 | 704 | -1.46E-05 | 2.05E-09 | 4.53E-05 | -0.3229 | 0.74677 | 0.99961 |
| 6 | 94118142 | 94441175 | 208 | 1.28E-05 | 5.32E-10 | 2.31E-05 | 0.55402 | 0.57956 | 0.99961 |
| 6 | 94441175 | 97093511 | 1910 | -1.83E-05 | 3.24E-09 | 5.69E-05 | -0.32121 | 0.74805 | 0.99961 |
| 6 | 97093511 | 97842284 | 547 | 4.13E-05 | 1.56E-09 | 3.95E-05 | 1.045 | 0.29602 | 0.99961 |
| 6 | 97842284 | 100630146 | 1814 | 5.37E-07 | 4.65E-09 | 6.82E-05 | 0.007884 | 0.99371 | 0.99961 |
| 6 | 100630146 | 102636772 | 1317 | 6.85E-06 | 3.70E-09 | 6.08E-05 | 0.11258 | 0.91036 | 0.99961 |
| 6 | 102636772 | 103983395 | 917 | 3.57E-05 | 2.04E-09 | 4.52E-05 | 0.79041 | 0.42929 | 0.99961 |
| 6 | 103983395 | 106056733 | 1382 | -6.90E-06 | 3.32E-09 | 5.77E-05 | -0.11975 | 0.90468 | 0.99961 |
| 6 | 106056733 | 107450306 | 1187 | 3.33E-05 | 3.54E-09 | 5.95E-05 | 0.55993 | 0.57553 | 0.99961 |
| 6 | 107450306 | 108464380 | 609 | 1.29E-05 | 2.33E-09 | 4.83E-05 | 0.26729 | 0.78925 | 0.99961 |
| 6 | 108464380 | 110304247 | 1064 | -1.32E-05 | 4.05E-09 | 6.37E-05 | -0.20721 | 0.83585 | 0.99961 |
| 6 | 110304247 | 112345014 | 1184 | 4.11E-05 | 3.18E-09 | 5.64E-05 | 0.73023 | 0.46525 | 0.99961 |
| 6 | 112345014 | 114220833 | 1399 | 0.00012144 | 4.00E-09 | 6.33E-05 | 1.9191 | 0.054971 | 0.99961 |
| 6 | 114220833 | 116131396 | 1142 | -3.64E-05 | 2.72E-09 | 5.21E-05 | -0.69924 | 0.4844 | 0.99961 |
| 6 | 116131396 | 117672972 | 1034 | 2.44E-05 | 3.14E-09 | 5.60E-05 | 0.4348 | 0.66371 | 0.99961 |
| 6 | 117672972 | 118963115 | 854 | -4.41E-06 | 2.34E-09 | 4.84E-05 | -0.09112 | 0.9274 | 0.99961 |
| 6 | 118963115 | 119963278 | 747 | 2.20E-05 | 2.53E-09 | 5.03E-05 | 0.43641 | 0.66254 | 0.99961 |
| 6 | 119963278 | 120512128 | 504 | -7.84E-06 | 1.21E-09 | 3.49E-05 | -0.22504 | 0.82194 | 0.99961 |
| 6 | 120512128 | 121905676 | 1059 | 2.18E-05 | 2.31E-09 | 4.81E-05 | 0.45293 | 0.6506 | 0.99961 |
| 6 | 121905676 | 123856181 | 1449 | 5.71E-05 | 3.83E-09 | 6.19E-05 | 0.92352 | 0.35574 | 0.99961 |
| 6 | 123856181 | 125424383 | 1244 | -6.34E-05 | 3.59E-09 | 5.99E-05 | -1.0586 | 0.28976 | 0.99961 |
| 6 | 125424383 | 127540461 | 1020 | -3.44E-05 | 1.22E-08 | 0.0001103 | -0.31166 | 0.7553 | 0.99961 |
| 6 | 127540461 | 129861031 | 1511 | 3.86E-05 | 3.71E-09 | 6.09E-05 | 0.63434 | 0.52586 | 0.99961 |
| 6 | 129861031 | 130935454 | 1044 | -1.07E-05 | 4.11E-09 | 6.41E-05 | -0.16626 | 0.86795 | 0.99961 |
| 6 | 130935454 | 132765669 | 1362 | 1.44E-05 | 3.41E-09 | 5.84E-05 | 0.24626 | 0.80548 | 0.99961 |
| 6 | 132765669 | 134244243 | 1248 | -1.79E-05 | 4.38E-09 | 6.62E-05 | -0.26979 | 0.78732 | 0.99961 |
| 6 | 134244243 | 136224177 | 1221 | 1.87E-06 | 3.06E-09 | 5.53E-05 | 0.033711 | 0.97311 | 0.99961 |
| 6 | 136224177 | 137614218 | 648 | -1.40E-06 | 2.48E-09 | 4.98E-05 | -0.02812 | 0.97756 | 0.99961 |
| 6 | 137614218 | 138822629 | 936 | -5.67E-05 | 3.07E-09 | 5.54E-05 | -1.0228 | 0.3064 | 0.99961 |
| 6 | 138822629 | 139845436 | 694 | 2.00E-05 | 5.39E-09 | 7.34E-05 | 0.27185 | 0.78574 | 0.99961 |
| 6 | 139845436 | 142288479 | 1360 | 2.86E-05 | 4.00E-09 | 6.32E-05 | 0.45165 | 0.65152 | 0.99961 |
| 6 | 142288479 | 145319810 | 2136 | 7.35E-05 | 3.49E-09 | 5.91E-05 | 1.243 | 0.21387 | 0.99961 |
| 6 | 145319810 | 146665424 | 899 | 1.51E-05 | 1.72E-09 | 4.15E-05 | 0.36492 | 0.71517 | 0.99961 |
| 6 | 146665424 | 148920352 | 1833 | 6.93E-06 | 3.24E-09 | 5.69E-05 | 0.12176 | 0.90309 | 0.99961 |
| 6 | 148920352 | 150253404 | 1063 | -4.03E-05 | 3.53E-09 | 5.94E-05 | -0.67755 | 0.49805 | 0.99961 |
| 6 | 150253404 | 151912703 | 1633 | -4.09E-06 | 3.16E-09 | 5.62E-05 | -0.07283 | 0.94194 | 0.99961 |
| 6 | 151912703 | 153094496 | 1045 | -1.47E-05 | 4.03E-09 | 6.35E-05 | -0.23118 | 0.81717 | 0.99961 |
| 6 | 153094496 | 154974120 | 1763 | 4.31E-05 | 3.92E-09 | 6.26E-05 | 0.68768 | 0.49166 | 0.99961 |
| 6 | 154974120 | 156763326 | 1363 | 2.16E-06 | 3.24E-09 | 5.69E-05 | 0.037892 | 0.96977 | 0.99961 |
| 6 | 156763326 | 158218719 | 629 | 9.08E-06 | 2.59E-09 | 5.09E-05 | 0.17853 | 0.85831 | 0.99961 |
| 6 | 158218719 | 160580497 | 2045 | -1.48E-05 | 3.29E-09 | 5.73E-05 | -0.25898 | 0.79565 | 0.99961 |
| 6 | 160580497 | 162169564 | 1390 | -7.14E-05 | 4.01E-09 | 6.33E-05 | -1.1265 | 0.25995 | 0.99961 |
| 6 | 162169564 | 162931203 | 697 | 6.63E-06 | 2.89E-09 | 5.38E-05 | 0.12334 | 0.90183 | 0.99961 |
| 6 | 162931203 | 164383521 | 1402 | 1.72E-05 | 3.48E-09 | 5.90E-05 | 0.29084 | 0.77117 | 0.99961 |
| 6 | 164383521 | 165586864 | 1286 | 1.23E-05 | 3.35E-09 | 5.79E-05 | 0.21328 | 0.8311 | 0.99961 |
| 6 | 165586864 | 167024733 | 1531 | 1.06E-05 | 3.20E-09 | 5.65E-05 | 0.18817 | 0.85074 | 0.99961 |
| 6 | 167024733 | 168042835 | 852 | 1.54E-05 | 3.81E-09 | 6.17E-05 | 0.24901 | 0.80335 | 0.99961 |
| 6 | 168042835 | 169382050 | 1230 | -1.39E-05 | 3.46E-09 | 5.88E-05 | -0.23589 | 0.81352 | 0.99961 |
| 6 | 169382050 | 170330173 | 834 | -6.14E-05 | 3.16E-09 | 5.62E-05 | -1.0926 | 0.27456 | 0.99961 |
| 6 | 170330173 | 171051270 | 481 | 5.90E-05 | 2.12E-09 | 4.61E-05 | 1.2808 | 0.20028 | 0.99961 |
| 7 | 16161 | 972752 | 144 | -1.23E-05 | 1.00E-09 | 3.17E-05 | -0.38844 | 0.69769 | 0.99961 |
| 7 | 972752 | 1353067 | 140 | -7.70E-05 | 1.50E-09 | 3.87E-05 | -1.9897 | 0.046629 | 0.99961 |
| 7 | 1353067 | 2062398 | 247 | 2.67E-05 | 1.59E-09 | 3.98E-05 | 0.67025 | 0.5027 | 0.99961 |
| 7 | 2062398 | 2772227 | 351 | 8.10E-05 | 2.60E-09 | 5.10E-05 | 1.5883 | 0.11223 | 0.99961 |
| 7 | 2772227 | 4573428 | 1697 | 2.90E-05 | 3.61E-09 | 6.01E-05 | 0.48237 | 0.62954 | 0.99961 |
| 7 | 4573428 | 5416232 | 317 | 1.18E-05 | 2.28E-09 | 4.78E-05 | 0.24721 | 0.80475 | 0.99961 |
| 7 | 5416232 | 5854526 | 152 | 6.25E-06 | 1.04E-09 | 3.23E-05 | 0.19328 | 0.84674 | 0.99961 |
| 7 | 5854526 | 6573857 | 231 | -2.13E-05 | 1.64E-09 | 4.05E-05 | -0.52647 | 0.59856 | 0.99961 |
| 7 | 6573857 | 7808752 | 844 | -2.23E-05 | 3.13E-09 | 5.59E-05 | -0.39926 | 0.6897 | 0.99961 |
| 7 | 7808752 | 9123423 | 1519 | 2.46E-05 | 3.39E-09 | 5.83E-05 | 0.42303 | 0.67227 | 0.99961 |
| 7 | 9123423 | 9987499 | 851 | 2.00E-05 | 2.87E-09 | 5.36E-05 | 0.37389 | 0.70849 | 0.99961 |
| 7 | 9987499 | 11299198 | 1162 | -9.51E-06 | 3.02E-09 | 5.49E-05 | -0.17325 | 0.86246 | 0.99961 |
| 7 | 11299198 | 12635461 | 1616 | 1.68E-05 | 2.98E-09 | 5.46E-05 | 0.3075 | 0.75846 | 0.99961 |
| 7 | 12635461 | 13884202 | 1323 | 1.65E-05 | 3.44E-09 | 5.87E-05 | 0.28102 | 0.7787 | 0.99961 |
| 7 | 13884202 | 15143657 | 1256 | 2.61E-05 | 3.29E-09 | 5.74E-05 | 0.45494 | 0.64915 | 0.99961 |
| 7 | 15143657 | 16902069 | 1588 | 6.34E-05 | 3.51E-09 | 5.93E-05 | 1.0695 | 0.28484 | 0.99961 |
| 7 | 16902069 | 19481547 | 2007 | 1.26E-05 | 3.66E-09 | 6.05E-05 | 0.2079 | 0.83531 | 0.99961 |
| 7 | 19481547 | 20124908 | 574 | -3.28E-05 | 2.35E-09 | 4.85E-05 | -0.67674 | 0.49857 | 0.99961 |
| 7 | 20124908 | 22507629 | 2309 | -5.45E-05 | 3.89E-09 | 6.23E-05 | -0.87381 | 0.38222 | 0.99961 |
| 7 | 22507629 | 23471442 | 574 | -2.77E-05 | 2.63E-09 | 5.12E-05 | -0.54133 | 0.58828 | 0.99961 |
| 7 | 23471442 | 25077259 | 1313 | 1.95E-05 | 3.32E-09 | 5.76E-05 | 0.33857 | 0.73493 | 0.99961 |
| 7 | 25077259 | 25909555 | 927 | 0.00014042 | 5.59E-09 | 7.48E-05 | 1.878 | 0.060381 | 0.99961 |
| 7 | 25909555 | 28360309 | 1926 | 1.99E-05 | 7.06E-09 | 8.40E-05 | 0.2372 | 0.8125 | 0.99961 |
| 7 | 28360309 | 31137289 | 2084 | 1.78E-05 | 3.39E-09 | 5.82E-05 | 0.30633 | 0.75935 | 0.99961 |
| 7 | 31137289 | 33555768 | 2049 | 4.24E-05 | 3.62E-09 | 6.01E-05 | 0.70524 | 0.48066 | 0.99961 |
| 7 | 33555768 | 36213538 | 2020 | -5.69E-05 | 3.61E-09 | 6.01E-05 | -0.94668 | 0.3438 | 0.99961 |
| 7 | 36213538 | 37555184 | 1132 | -5.50E-05 | 3.61E-09 | 6.01E-05 | -0.91488 | 0.36026 | 0.99961 |
| 7 | 37555184 | 38966703 | 1291 | -2.54E-05 | 3.23E-09 | 5.68E-05 | -0.44711 | 0.6548 | 0.99961 |
| 7 | 38966703 | 39862670 | 617 | -3.11E-05 | 1.82E-09 | 4.27E-05 | -0.7298 | 0.46551 | 0.99961 |
| 7 | 39862670 | 42001811 | 1285 | 1.84E-05 | 3.33E-09 | 5.77E-05 | 0.31865 | 0.74999 | 0.99961 |
| 7 | 42001811 | 43159074 | 865 | -9.82E-06 | 3.92E-09 | 6.26E-05 | -0.15685 | 0.87537 | 0.99961 |
| 7 | 43159074 | 44763828 | 955 | -7.20E-07 | 3.18E-09 | 5.64E-05 | -0.01276 | 0.98982 | 0.99961 |
| 7 | 44763828 | 45952922 | 685 | -1.70E-05 | 2.72E-09 | 5.21E-05 | -0.32688 | 0.74376 | 0.99961 |
| 7 | 45952922 | 46986720 | 1016 | 1.82E-05 | 3.31E-09 | 5.76E-05 | 0.3166 | 0.75155 | 0.99961 |
| 7 | 46986720 | 49212278 | 1810 | -1.20E-05 | 3.25E-09 | 5.70E-05 | -0.20999 | 0.83368 | 0.99961 |
| 7 | 49212278 | 51675322 | 1908 | -5.29E-05 | 3.83E-09 | 6.18E-05 | -0.85512 | 0.39249 | 0.99961 |
| 7 | 51675322 | 53176312 | 1568 | -4.24E-06 | 2.94E-09 | 5.42E-05 | -0.07829 | 0.9376 | 0.99961 |
| 7 | 53176312 | 54222976 | 897 | 2.17E-05 | 2.22E-09 | 4.71E-05 | 0.46016 | 0.6454 | 0.99961 |
| 7 | 54222976 | 55691584 | 1122 | 8.56E-06 | 3.55E-09 | 5.96E-05 | 0.14358 | 0.88583 | 0.99961 |
| 7 | 55691584 | 57382638 | 597 | 1.70E-05 | 2.03E-09 | 4.51E-05 | 0.37635 | 0.70666 | 0.99961 |
| 7 | 57382638 | 63307359 | 378 | 3.39E-05 | 1.57E-09 | 3.96E-05 | 0.85428 | 0.39295 | 0.99961 |
| 7 | 63307359 | 63641125 | 149 | -9.08E-06 | 5.64E-10 | 2.37E-05 | -0.38245 | 0.70213 | 0.99961 |
| 7 | 63641125 | 65689809 | 712 | 1.24E-05 | 2.26E-09 | 4.76E-05 | 0.2605 | 0.79448 | 0.99961 |
| 7 | 65689809 | 68234074 | 1522 | 6.16E-05 | 3.82E-09 | 6.18E-05 | 0.99696 | 0.31879 | 0.99961 |
| 7 | 68234074 | 69085364 | 505 | -1.78E-06 | 2.24E-09 | 4.73E-05 | -0.03759 | 0.97001 | 0.99961 |
| 7 | 69085364 | 71874885 | 1574 | 1.67E-05 | 3.51E-09 | 5.93E-05 | 0.28165 | 0.77821 | 0.99961 |
| 7 | 71874885 | 73334602 | 237 | 3.20E-06 | 2.69E-09 | 5.18E-05 | 0.06179 | 0.95073 | 0.99961 |
| 7 | 73334602 | 76458564 | 570 | -4.87E-05 | 3.48E-09 | 5.90E-05 | -0.82494 | 0.40941 | 0.99961 |
| 7 | 76458564 | 78093859 | 1160 | -1.89E-06 | 4.86E-09 | 6.97E-05 | -0.02714 | 0.97835 | 0.99961 |
| 7 | 78093859 | 79888069 | 1434 | -1.45E-05 | 3.45E-09 | 5.87E-05 | -0.24739 | 0.80461 | 0.99961 |
| 7 | 79888069 | 82022686 | 1526 | -4.86E-05 | 4.39E-09 | 6.63E-05 | -0.73236 | 0.46395 | 0.99961 |
| 7 | 82022686 | 83801203 | 1426 | 1.61E-05 | 3.11E-09 | 5.58E-05 | 0.28835 | 0.77308 | 0.99961 |
| 7 | 83801203 | 85572182 | 1046 | 5.46E-05 | 3.29E-09 | 5.73E-05 | 0.95144 | 0.34138 | 0.99961 |
| 7 | 85572182 | 87825004 | 1202 | -4.08E-06 | 2.94E-09 | 5.43E-05 | -0.0752 | 0.94006 | 0.99961 |
| 7 | 87825004 | 90661784 | 2547 | 2.85E-05 | 3.21E-09 | 5.67E-05 | 0.50195 | 0.6157 | 0.99961 |
| 7 | 90661784 | 92493752 | 731 | 1.48E-06 | 2.59E-09 | 5.09E-05 | 0.029052 | 0.97682 | 0.99961 |
| 7 | 92493752 | 93966601 | 782 | 2.66E-05 | 3.41E-09 | 5.84E-05 | 0.45612 | 0.6483 | 0.99961 |
| 7 | 93966601 | 96073508 | 1316 | 3.92E-05 | 4.10E-09 | 6.40E-05 | 0.61315 | 0.53978 | 0.99961 |
| 7 | 96073508 | 97099570 | 612 | 5.48E-05 | 2.72E-09 | 5.22E-05 | 1.0498 | 0.29379 | 0.99961 |
| 7 | 97099570 | 98715474 | 806 | -2.34E-06 | 3.41E-09 | 5.84E-05 | -0.04001 | 0.96808 | 0.99961 |
| 7 | 98715474 | 100196651 | 383 | 7.36E-06 | 1.73E-09 | 4.16E-05 | 0.17693 | 0.85956 | 0.99961 |
| 7 | 100196651 | 101199253 | 389 | -7.62E-07 | 2.06E-09 | 4.54E-05 | -0.01678 | 0.98661 | 0.99961 |
| 7 | 101199253 | 103197510 | 698 | 3.95E-05 | 3.23E-09 | 5.68E-05 | 0.69602 | 0.48641 | 0.99961 |
| 7 | 103197510 | 104159525 | 957 | -3.61E-05 | 3.31E-09 | 5.75E-05 | -0.62722 | 0.53052 | 0.99961 |
| 7 | 104159525 | 105682905 | 838 | 2.94E-05 | 3.36E-09 | 5.80E-05 | 0.5068 | 0.6123 | 0.99961 |
| 7 | 105682905 | 107780178 | 1399 | 2.23E-05 | 5.05E-09 | 7.11E-05 | 0.31366 | 0.75378 | 0.99961 |
| 7 | 107780178 | 109647594 | 1246 | 7.36E-05 | 3.58E-09 | 5.98E-05 | 1.2293 | 0.21897 | 0.99961 |
| 7 | 109647594 | 112618684 | 1623 | 2.06E-05 | 3.06E-09 | 5.54E-05 | 0.37303 | 0.70913 | 0.99961 |
| 7 | 112618684 | 113712387 | 544 | -3.35E-05 | 1.76E-09 | 4.19E-05 | -0.79858 | 0.42454 | 0.99961 |
| 7 | 113712387 | 116780178 | 1538 | -4.04E-05 | 3.45E-09 | 5.87E-05 | -0.68767 | 0.49166 | 0.99961 |
| 7 | 116780178 | 118351581 | 887 | 7.92E-05 | 2.80E-09 | 5.29E-05 | 1.4975 | 0.13426 | 0.99961 |
| 7 | 118351581 | 121045273 | 1112 | 7.84E-06 | 2.98E-09 | 5.46E-05 | 0.14376 | 0.88569 | 0.99961 |
| 7 | 121045273 | 121933630 | 445 | -2.30E-05 | 1.57E-09 | 3.96E-05 | -0.58142 | 0.56096 | 0.99961 |
| 7 | 121933630 | 124156805 | 1602 | -1.22E-05 | 3.11E-09 | 5.57E-05 | -0.21942 | 0.82632 | 0.99961 |
| 7 | 124156805 | 124167552 | 9 | -1.67E-06 | 1.73E-10 | 1.32E-05 | -0.12683 | 0.89908 | 0.99961 |
| 7 | 124167552 | 126519170 | 1821 | 1.14E-05 | 3.30E-09 | 5.74E-05 | 0.19889 | 0.84235 | 0.99961 |
| 7 | 126519170 | 126869221 | 373 | -2.49E-05 | 1.38E-09 | 3.71E-05 | -0.67229 | 0.5014 | 0.99961 |
| 7 | 126869221 | 128778386 | 1076 | -1.68E-05 | 3.57E-09 | 5.97E-05 | -0.28111 | 0.77862 | 0.99961 |
| 7 | 128778386 | 130422414 | 731 | 7.33E-06 | 3.32E-09 | 5.76E-05 | 0.12719 | 0.89879 | 0.99961 |
| 7 | 130422414 | 132805848 | 1685 | 7.57E-05 | 4.51E-09 | 6.72E-05 | 1.1279 | 0.25937 | 0.99961 |
| 7 | 132805848 | 134307596 | 1025 | 1.77E-05 | 2.73E-09 | 5.22E-05 | 0.33941 | 0.7343 | 0.99961 |
| 7 | 134307596 | 135591083 | 966 | 9.98E-06 | 3.55E-09 | 5.96E-05 | 0.16758 | 0.86692 | 0.99961 |
| 7 | 135591083 | 136876562 | 944 | -2.03E-05 | 2.80E-09 | 5.29E-05 | -0.38256 | 0.70205 | 0.99961 |
| 7 | 136876562 | 138744679 | 1104 | 2.04E-05 | 3.52E-09 | 5.93E-05 | 0.3444 | 0.73055 | 0.99961 |
| 7 | 138744679 | 139933177 | 510 | 9.39E-05 | 2.64E-09 | 5.14E-05 | 1.8268 | 0.067737 | 0.99961 |
| 7 | 139933177 | 140235210 | 169 | -2.96E-05 | 1.01E-09 | 3.18E-05 | -0.93247 | 0.35109 | 0.99961 |
| 7 | 140235210 | 141226557 | 519 | 1.64E-06 | 1.84E-09 | 4.29E-05 | 0.038227 | 0.96951 | 0.99961 |
| 7 | 141226557 | 142656310 | 693 | 4.46E-05 | 1.81E-09 | 4.26E-05 | 1.046 | 0.29558 | 0.99961 |
| 7 | 142656310 | 144968289 | 1135 | -2.50E-05 | 3.23E-09 | 5.68E-05 | -0.44023 | 0.65977 | 0.99961 |
| 7 | 144968289 | 146427960 | 1046 | -4.14E-06 | 2.71E-09 | 5.21E-05 | -0.07946 | 0.93667 | 0.99961 |
| 7 | 146427960 | 147815318 | 1416 | -2.69E-05 | 3.16E-09 | 5.63E-05 | -0.47767 | 0.63288 | 0.99961 |
| 7 | 147815318 | 149840658 | 1050 | 3.23E-05 | 3.14E-09 | 5.61E-05 | 0.57688 | 0.56402 | 0.99961 |
| 7 | 149840658 | 150711505 | 551 | 2.46E-05 | 2.49E-09 | 4.99E-05 | 0.4935 | 0.62166 | 0.99961 |
| 7 | 150711505 | 152249806 | 724 | 2.44E-05 | 3.77E-09 | 6.14E-05 | 0.39694 | 0.69141 | 0.99961 |
| 7 | 152249806 | 153674019 | 946 | 2.82E-05 | 3.38E-09 | 5.82E-05 | 0.48521 | 0.62753 | 0.99961 |
| 7 | 153674019 | 154964730 | 943 | 2.74E-06 | 3.22E-09 | 5.68E-05 | 0.048354 | 0.96143 | 0.99961 |
| 7 | 154964730 | 156065250 | 875 | -3.03E-06 | 3.38E-09 | 5.81E-05 | -0.05209 | 0.95846 | 0.99961 |
| 7 | 156065250 | 157634597 | 933 | -1.38E-06 | 3.48E-09 | 5.90E-05 | -0.02345 | 0.98129 | 0.99961 |
| 7 | 157634597 | 159128575 | 823 | -1.76E-05 | 3.05E-09 | 5.52E-05 | -0.31933 | 0.74948 | 0.99961 |
| 8 | 10422 | 1044217 | 689 | 2.65E-06 | 3.38E-09 | 5.82E-05 | 0.04556 | 0.96366 | 0.99961 |
| 8 | 1044217 | 1163245 | 125 | -2.23E-05 | 8.23E-10 | 2.87E-05 | -0.77594 | 0.43778 | 0.99961 |
| 8 | 1163245 | 2042942 | 729 | -3.38E-05 | 3.42E-09 | 5.84E-05 | -0.57868 | 0.5628 | 0.99961 |
| 8 | 2042942 | 2573279 | 534 | -1.36E-05 | 2.67E-09 | 5.16E-05 | -0.26393 | 0.79183 | 0.99961 |
| 8 | 2573279 | 3392926 | 1099 | -2.37E-06 | 3.17E-09 | 5.63E-05 | -0.04209 | 0.96643 | 0.99961 |
| 8 | 3392926 | 3783017 | 859 | 4.07E-05 | 3.31E-09 | 5.75E-05 | 0.7075 | 0.47926 | 0.99961 |
| 8 | 3783017 | 4480476 | 1256 | 1.21E-05 | 3.18E-09 | 5.64E-05 | 0.21478 | 0.82994 | 0.99961 |
| 8 | 4480476 | 5146927 | 961 | 5.91E-06 | 3.34E-09 | 5.78E-05 | 0.10225 | 0.91856 | 0.99961 |
| 8 | 5146927 | 6142797 | 1726 | -2.29E-05 | 3.27E-09 | 5.71E-05 | -0.40098 | 0.68843 | 0.99961 |
| 8 | 6142797 | 7153079 | 919 | -1.13E-05 | 3.28E-09 | 5.72E-05 | -0.19729 | 0.8436 | 0.99961 |
| 8 | 7153079 | 9154694 | 1133 | -2.52E-05 | 3.52E-09 | 5.93E-05 | -0.4253 | 0.67062 | 0.99961 |
| 8 | 9154694 | 9640787 | 459 | -4.01E-05 | 2.36E-09 | 4.85E-05 | -0.82712 | 0.40817 | 0.99961 |
| 8 | 9640787 | 10463197 | 920 | -2.47E-05 | 3.37E-09 | 5.81E-05 | -0.42613 | 0.67001 | 0.99961 |
| 8 | 10463197 | 11278998 | 732 | -3.24E-05 | 3.59E-09 | 5.99E-05 | -0.54038 | 0.58893 | 0.99961 |
| 8 | 11278998 | 13491775 | 1481 | 1.63E-06 | 4.03E-09 | 6.35E-05 | 0.025721 | 0.97948 | 0.99961 |
| 8 | 13491775 | 13944992 | 674 | 1.19E-05 | 3.00E-09 | 5.48E-05 | 0.21695 | 0.82825 | 0.99961 |
| 8 | 13944992 | 15991660 | 2222 | 2.15E-05 | 3.14E-09 | 5.60E-05 | 0.38317 | 0.70159 | 0.99961 |
| 8 | 15991660 | 17387876 | 1144 | 5.81E-05 | 3.76E-09 | 6.13E-05 | 0.94776 | 0.34325 | 0.99961 |
| 8 | 17387876 | 17836399 | 530 | 5.98E-05 | 2.99E-09 | 5.47E-05 | 1.0928 | 0.27447 | 0.99961 |
| 8 | 17836399 | 19492840 | 1763 | -2.64E-05 | 3.44E-09 | 5.87E-05 | -0.45054 | 0.65232 | 0.99961 |
| 8 | 19492840 | 20060856 | 606 | 2.02E-05 | 2.97E-09 | 5.45E-05 | 0.37038 | 0.7111 | 0.99961 |
| 8 | 20060856 | 21661737 | 1542 | -4.53E-05 | 3.53E-09 | 5.94E-05 | -0.76159 | 0.4463 | 0.99961 |
| 8 | 21661737 | 22897057 | 714 | 1.97E-05 | 3.70E-09 | 6.08E-05 | 0.32449 | 0.74557 | 0.99961 |
| 8 | 22897057 | 24674718 | 1268 | 0.00014714 | 5.50E-09 | 7.42E-05 | 1.9842 | 0.04723 | 0.99961 |
| 8 | 24674718 | 25483454 | 531 | 1.51E-05 | 2.34E-09 | 4.84E-05 | 0.31135 | 0.75553 | 0.99961 |
| 8 | 25483454 | 26682525 | 1146 | -4.81E-05 | 4.41E-09 | 6.64E-05 | -0.72413 | 0.46899 | 0.99961 |
| 8 | 26682525 | 28162392 | 1517 | -7.49E-05 | 3.50E-09 | 5.92E-05 | -1.2657 | 0.20562 | 0.99961 |
| 8 | 28162392 | 29327896 | 656 | -1.94E-07 | 2.50E-09 | 5.00E-05 | -0.00388 | 0.9969 | 0.99961 |
| 8 | 29327896 | 31133729 | 1046 | -3.79E-05 | 4.07E-09 | 6.38E-05 | -0.5948 | 0.55198 | 0.99961 |
| 8 | 31133729 | 31548360 | 271 | -6.81E-06 | 1.26E-09 | 3.55E-05 | -0.19168 | 0.84799 | 0.99961 |
| 8 | 31548360 | 32977218 | 1029 | 1.26E-06 | 3.05E-09 | 5.52E-05 | 0.022892 | 0.98174 | 0.99961 |
| 8 | 32977218 | 35317232 | 1425 | 1.25E-05 | 3.21E-09 | 5.66E-05 | 0.21997 | 0.82589 | 0.99961 |
| 8 | 35317232 | 37379389 | 805 | -6.20E-06 | 2.91E-09 | 5.40E-05 | -0.11479 | 0.90861 | 0.99961 |
| 8 | 37379389 | 38938806 | 545 | -9.13E-07 | 2.67E-09 | 5.17E-05 | -0.01766 | 0.98591 | 0.99961 |
| 8 | 38938806 | 40703337 | 1143 | -2.44E-06 | 3.08E-09 | 5.55E-05 | -0.044 | 0.9649 | 0.99961 |
| 8 | 40703337 | 41721454 | 735 | -2.71E-05 | 3.06E-09 | 5.53E-05 | -0.49005 | 0.6241 | 0.99961 |
| 8 | 41721454 | 42773823 | 305 | -5.35E-06 | 1.35E-09 | 3.67E-05 | -0.14571 | 0.88415 | 0.99961 |
| 8 | 42773823 | 46841315 | 111 | 9.17E-06 | 5.32E-10 | 2.31E-05 | 0.39764 | 0.6909 | 0.99961 |
| 8 | 46841315 | 50082470 | 825 | 1.11E-05 | 2.69E-09 | 5.19E-05 | 0.2149 | 0.82985 | 0.99961 |
| 8 | 50082470 | 53302930 | 2240 | -9.69E-06 | 3.38E-09 | 5.82E-05 | -0.16654 | 0.86774 | 0.99961 |
| 8 | 53302930 | 54418044 | 811 | -5.39E-07 | 2.19E-09 | 4.68E-05 | -0.01151 | 0.99082 | 0.99961 |
| 8 | 54418044 | 55945581 | 1018 | 3.25E-05 | 2.94E-09 | 5.42E-05 | 0.60022 | 0.54836 | 0.99961 |
| 8 | 55945581 | 56956383 | 730 | 1.16E-05 | 2.32E-09 | 4.82E-05 | 0.24108 | 0.80949 | 0.99961 |
| 8 | 56956383 | 59068651 | 1602 | -3.65E-06 | 3.36E-09 | 5.80E-05 | -0.063 | 0.94977 | 0.99961 |
| 8 | 59068651 | 59728100 | 613 | 3.27E-05 | 2.11E-09 | 4.59E-05 | 0.71264 | 0.47607 | 0.99961 |
| 8 | 59728100 | 62329099 | 1765 | 2.20E-05 | 3.64E-09 | 6.04E-05 | 0.36505 | 0.71508 | 0.99961 |
| 8 | 62329099 | 63349306 | 752 | -6.06E-06 | 2.11E-09 | 4.59E-05 | -0.13195 | 0.89503 | 0.99961 |
| 8 | 63349306 | 65232872 | 1280 | 1.84E-05 | 3.00E-09 | 5.47E-05 | 0.33668 | 0.73636 | 0.99961 |
| 8 | 65232872 | 66489090 | 734 | 2.95E-05 | 2.49E-09 | 4.99E-05 | 0.59097 | 0.55454 | 0.99961 |
| 8 | 66489090 | 69863057 | 1757 | 3.79E-05 | 3.64E-09 | 6.03E-05 | 0.62904 | 0.52932 | 0.99961 |
| 8 | 69863057 | 70867979 | 896 | -1.98E-05 | 3.38E-09 | 5.81E-05 | -0.33995 | 0.7339 | 0.99961 |
| 8 | 70867979 | 72289454 | 983 | -2.52E-05 | 3.80E-09 | 6.16E-05 | -0.40876 | 0.68271 | 0.99961 |
| 8 | 72289454 | 73817199 | 1498 | 4.48E-05 | 5.27E-09 | 7.26E-05 | 0.61666 | 0.53746 | 0.99961 |
| 8 | 73817199 | 75445064 | 1158 | 3.64E-05 | 3.31E-09 | 5.76E-05 | 0.63222 | 0.52724 | 0.99961 |
| 8 | 75445064 | 76456542 | 698 | 3.48E-05 | 2.54E-09 | 5.04E-05 | 0.68983 | 0.4903 | 0.99961 |
| 8 | 76456542 | 79132861 | 1835 | 3.77E-05 | 3.96E-09 | 6.29E-05 | 0.59868 | 0.54938 | 0.99961 |
| 8 | 79132861 | 81956395 | 1561 | -1.75E-05 | 3.47E-09 | 5.89E-05 | -0.29689 | 0.76655 | 0.99961 |
| 8 | 81956395 | 82693739 | 511 | -4.04E-05 | 1.95E-09 | 4.41E-05 | -0.91604 | 0.35965 | 0.99961 |
| 8 | 82693739 | 84225589 | 1176 | 3.64E-05 | 2.50E-09 | 5.00E-05 | 0.72697 | 0.46725 | 0.99961 |
| 8 | 84225589 | 85955619 | 957 | -4.14E-06 | 2.23E-09 | 4.72E-05 | -0.08762 | 0.93018 | 0.99961 |
| 8 | 85955619 | 87411709 | 510 | -4.67E-05 | 1.67E-09 | 4.09E-05 | -1.1419 | 0.25351 | 0.99961 |
| 8 | 87411709 | 89085481 | 1099 | -1.72E-05 | 3.04E-09 | 5.51E-05 | -0.3122 | 0.75489 | 0.99961 |
| 8 | 89085481 | 90638162 | 954 | -6.45E-05 | 3.32E-09 | 5.76E-05 | -1.1194 | 0.26295 | 0.99961 |
| 8 | 90638162 | 93554386 | 1514 | 3.90E-05 | 3.74E-09 | 6.11E-05 | 0.63792 | 0.52352 | 0.99961 |
| 8 | 93554386 | 95005527 | 774 | 2.42E-05 | 2.93E-09 | 5.41E-05 | 0.4472 | 0.65473 | 0.99961 |
| 8 | 95005527 | 96534806 | 1146 | 8.56E-06 | 3.47E-09 | 5.89E-05 | 0.14545 | 0.88436 | 0.99961 |
| 8 | 96534806 | 99401581 | 2079 | 1.16E-05 | 3.51E-09 | 5.93E-05 | 0.19495 | 0.84543 | 0.99961 |
| 8 | 99401581 | 101620958 | 817 | 1.04E-05 | 2.60E-09 | 5.10E-05 | 0.20359 | 0.83867 | 0.99961 |
| 8 | 101620958 | 104123306 | 1899 | 6.15E-07 | 3.37E-09 | 5.80E-05 | 0.010592 | 0.99155 | 0.99961 |
| 8 | 104123306 | 104988752 | 697 | 2.12E-05 | 1.53E-09 | 3.91E-05 | 0.5427 | 0.58734 | 0.99961 |
| 8 | 104988752 | 107410754 | 1823 | -1.12E-05 | 3.35E-09 | 5.79E-05 | -0.19302 | 0.84694 | 0.99961 |
| 8 | 107410754 | 108648177 | 1095 | -3.55E-05 | 2.64E-09 | 5.14E-05 | -0.69122 | 0.48943 | 0.99961 |
| 8 | 108648177 | 109248173 | 504 | 1.99E-07 | 1.42E-09 | 3.77E-05 | 0.00528 | 0.99579 | 0.99961 |
| 8 | 109248173 | 110484314 | 953 | -2.02E-05 | 2.01E-09 | 4.49E-05 | -0.44962 | 0.65299 | 0.99961 |
| 8 | 110484314 | 111850847 | 726 | 5.50E-05 | 1.72E-09 | 4.14E-05 | 1.3287 | 0.18396 | 0.99961 |
| 8 | 111850847 | 113064320 | 744 | -2.48E-06 | 1.27E-09 | 3.57E-05 | -0.06954 | 0.94456 | 0.99961 |
| 8 | 113064320 | 116096495 | 2134 | -5.00E-06 | 3.09E-09 | 5.56E-05 | -0.08994 | 0.92834 | 0.99961 |
| 8 | 116096495 | 119685457 | 2548 | -2.62E-05 | 3.59E-09 | 5.99E-05 | -0.43678 | 0.66227 | 0.99961 |
| 8 | 119685457 | 121201700 | 1222 | -2.40E-05 | 3.64E-09 | 6.03E-05 | -0.39748 | 0.69102 | 0.99961 |
| 8 | 121201700 | 124861459 | 2672 | 3.33E-05 | 3.75E-09 | 6.13E-05 | 0.54291 | 0.58719 | 0.99961 |
| 8 | 124861459 | 125683719 | 584 | -2.85E-05 | 1.96E-09 | 4.43E-05 | -0.6441 | 0.51951 | 0.99961 |
| 8 | 125683719 | 126410917 | 465 | -6.26E-05 | 2.41E-09 | 4.91E-05 | -1.2745 | 0.20247 | 0.99961 |
| 8 | 126410917 | 128659111 | 1829 | 7.61E-05 | 5.39E-09 | 7.34E-05 | 1.0364 | 0.30001 | 0.99961 |
| 8 | 128659111 | 130381139 | 1467 | 6.13E-05 | 3.84E-09 | 6.19E-05 | 0.99032 | 0.32202 | 0.99961 |
| 8 | 130381139 | 131639625 | 928 | 1.36E-05 | 3.31E-09 | 5.75E-05 | 0.23683 | 0.81279 | 0.99961 |
| 8 | 131639625 | 133351144 | 1606 | 4.48E-05 | 2.98E-09 | 5.46E-05 | 0.82125 | 0.4115 | 0.99961 |
| 8 | 133351144 | 134570001 | 1386 | 1.63E-05 | 2.97E-09 | 5.45E-05 | 0.29947 | 0.76459 | 0.99961 |
| 8 | 134570001 | 136035605 | 1567 | 6.75E-05 | 4.36E-09 | 6.61E-05 | 1.022 | 0.30677 | 0.99961 |
| 8 | 136035605 | 137524151 | 1269 | -2.12E-06 | 3.32E-09 | 5.76E-05 | -0.03674 | 0.97069 | 0.99961 |
| 8 | 137524151 | 139254830 | 1752 | 4.47E-05 | 3.10E-09 | 5.57E-05 | 0.80344 | 0.42172 | 0.99961 |
| 8 | 139254830 | 140725643 | 1405 | -8.13E-06 | 3.35E-09 | 5.79E-05 | -0.14038 | 0.88836 | 0.99961 |
| 8 | 140725643 | 143044914 | 1504 | 1.19E-05 | 3.23E-09 | 5.68E-05 | 0.20975 | 0.83386 | 0.99961 |
| 8 | 143044914 | 144236881 | 717 | -1.80E-05 | 2.83E-09 | 5.32E-05 | -0.33896 | 0.73464 | 0.99961 |
| 8 | 144236881 | 146303867 | 589 | 4.03E-05 | 3.21E-09 | 5.67E-05 | 0.71086 | 0.47717 | 0.99961 |
| 9 | 10023 | 1079707 | 1025 | -2.03E-05 | 3.11E-09 | 5.58E-05 | -0.36429 | 0.71564 | 0.99961 |
| 9 | 1079707 | 1916877 | 1195 | 9.46E-06 | 3.20E-09 | 5.66E-05 | 0.16723 | 0.86719 | 0.99961 |
| 9 | 1916877 | 3190064 | 1438 | -4.57E-05 | 3.29E-09 | 5.73E-05 | -0.7963 | 0.42586 | 0.99961 |
| 9 | 3190064 | 4495328 | 1362 | -2.43E-05 | 3.30E-09 | 5.75E-05 | -0.42335 | 0.67204 | 0.99961 |
| 9 | 4495328 | 4884926 | 434 | -1.75E-05 | 2.71E-09 | 5.20E-05 | -0.33672 | 0.73633 | 0.99961 |
| 9 | 4884926 | 6557589 | 1053 | -2.93E-05 | 3.61E-09 | 6.01E-05 | -0.48657 | 0.62657 | 0.99961 |
| 9 | 6557589 | 7154923 | 576 | 4.20E-05 | 2.86E-09 | 5.35E-05 | 0.78501 | 0.43245 | 0.99961 |
| 9 | 7154923 | 8456299 | 1818 | 1.28E-05 | 3.34E-09 | 5.78E-05 | 0.22143 | 0.82476 | 0.99961 |
| 9 | 8456299 | 9166403 | 767 | -1.51E-05 | 3.14E-09 | 5.60E-05 | -0.26867 | 0.78818 | 0.99961 |
| 9 | 9166403 | 10879253 | 2024 | 3.43E-05 | 3.34E-09 | 5.78E-05 | 0.5936 | 0.55278 | 0.99961 |
| 9 | 10879253 | 12276489 | 1409 | 1.04E-05 | 3.12E-09 | 5.58E-05 | 0.18548 | 0.85285 | 0.99961 |
| 9 | 12276489 | 14836363 | 2522 | 4.30E-06 | 3.51E-09 | 5.93E-05 | 0.072474 | 0.94222 | 0.99961 |
| 9 | 14836363 | 16659655 | 1623 | 8.00E-06 | 3.31E-09 | 5.76E-05 | 0.13898 | 0.88947 | 0.99961 |
| 9 | 16659655 | 18661051 | 2031 | 3.93E-05 | 3.64E-09 | 6.03E-05 | 0.65136 | 0.51482 | 0.99961 |
| 9 | 18661051 | 20463534 | 1583 | -1.07E-05 | 3.15E-09 | 5.61E-05 | -0.19114 | 0.84842 | 0.99961 |
| 9 | 20463534 | 22206559 | 1431 | 3.64E-05 | 3.50E-09 | 5.92E-05 | 0.61462 | 0.53881 | 0.99961 |
| 9 | 22206559 | 24158518 | 1343 | -2.74E-05 | 3.12E-09 | 5.59E-05 | -0.49039 | 0.62386 | 0.99961 |
| 9 | 24158518 | 25330810 | 698 | 1.10E-05 | 3.06E-09 | 5.53E-05 | 0.19828 | 0.84283 | 0.99961 |
| 9 | 25330810 | 26111757 | 594 | 1.30E-05 | 3.08E-09 | 5.55E-05 | 0.2341 | 0.81491 | 0.99961 |
| 9 | 26111757 | 28224283 | 1922 | -1.79E-05 | 2.96E-09 | 5.44E-05 | -0.32977 | 0.74158 | 0.99961 |
| 9 | 28224283 | 28811584 | 562 | -1.06E-05 | 1.89E-09 | 4.35E-05 | -0.24437 | 0.80695 | 0.99961 |
| 9 | 28811584 | 30387392 | 1506 | 4.94E-05 | 3.44E-09 | 5.86E-05 | 0.84225 | 0.39965 | 0.99961 |
| 9 | 30387392 | 31310383 | 799 | 3.14E-05 | 2.12E-09 | 4.60E-05 | 0.68215 | 0.49514 | 0.99961 |
| 9 | 31310383 | 32019368 | 642 | 1.81E-05 | 1.96E-09 | 4.43E-05 | 0.40916 | 0.68243 | 0.99961 |
| 9 | 32019368 | 33578334 | 1264 | -4.40E-05 | 3.57E-09 | 5.98E-05 | -0.73666 | 0.46133 | 0.99961 |
| 9 | 33578334 | 34642243 | 557 | -1.93E-06 | 1.40E-09 | 3.74E-05 | -0.05164 | 0.95881 | 0.99961 |
| 9 | 34642243 | 36743283 | 1028 | -2.43E-05 | 3.81E-09 | 6.17E-05 | -0.39333 | 0.69407 | 0.99961 |
| 9 | 36743283 | 38641599 | 1358 | -9.17E-06 | 3.08E-09 | 5.55E-05 | -0.16517 | 0.86881 | 0.99961 |
| 9 | 38641599 | 65472190 | 175 | 4.60E-06 | 9.70E-10 | 3.11E-05 | 0.14768 | 0.88259 | 0.99961 |
| 9 | 65472190 | 72512711 | 1092 | -4.70E-05 | 3.31E-09 | 5.76E-05 | -0.81705 | 0.4139 | 0.99961 |
| 9 | 72512711 | 72753755 | 213 | -2.15E-05 | 8.28E-10 | 2.88E-05 | -0.74639 | 0.45543 | 0.99961 |
| 9 | 72753755 | 74253040 | 1211 | -1.97E-05 | 3.17E-09 | 5.63E-05 | -0.34944 | 0.72676 | 0.99961 |
| 9 | 74253040 | 76203946 | 1217 | 5.79E-08 | 3.36E-09 | 5.80E-05 | 0.001 | 0.9992 | 0.99961 |
| 9 | 76203946 | 76973081 | 513 | -1.72E-05 | 1.36E-09 | 3.69E-05 | -0.46729 | 0.64029 | 0.99961 |
| 9 | 76973081 | 78900183 | 1669 | -1.40E-05 | 3.35E-09 | 5.79E-05 | -0.24095 | 0.80959 | 0.99961 |
| 9 | 78900183 | 79471208 | 495 | 2.72E-05 | 2.22E-09 | 4.71E-05 | 0.57729 | 0.56375 | 0.99961 |
| 9 | 79471208 | 81079055 | 1134 | 3.25E-05 | 3.33E-09 | 5.77E-05 | 0.56321 | 0.57329 | 0.99961 |
| 9 | 81079055 | 82590928 | 1291 | 9.16E-05 | 3.48E-09 | 5.90E-05 | 1.5537 | 0.12025 | 0.99961 |
| 9 | 82590928 | 84211233 | 1644 | 8.76E-06 | 3.04E-09 | 5.52E-05 | 0.15875 | 0.87387 | 0.99961 |
| 9 | 84211233 | 85440801 | 647 | -1.24E-05 | 2.91E-09 | 5.40E-05 | -0.23058 | 0.81764 | 0.99961 |
| 9 | 85440801 | 86938196 | 1270 | -4.21E-05 | 3.23E-09 | 5.68E-05 | -0.74142 | 0.45844 | 0.99961 |
| 9 | 86938196 | 88109333 | 963 | -1.13E-05 | 3.31E-09 | 5.76E-05 | -0.19613 | 0.84451 | 0.99961 |
| 9 | 88109333 | 88590417 | 200 | -8.27E-06 | 7.42E-10 | 2.72E-05 | -0.30345 | 0.76155 | 0.99961 |
| 9 | 88590417 | 90136246 | 1192 | -2.37E-06 | 3.47E-09 | 5.89E-05 | -0.04022 | 0.96792 | 0.99961 |
| 9 | 90136246 | 92528698 | 1673 | 2.65E-05 | 3.36E-09 | 5.79E-05 | 0.45751 | 0.64731 | 0.99961 |
| 9 | 92528698 | 94167203 | 1223 | -4.54E-05 | 3.94E-09 | 6.28E-05 | -0.72338 | 0.46945 | 0.99961 |
| 9 | 94167203 | 96671698 | 1592 | 1.36E-05 | 4.20E-09 | 6.48E-05 | 0.21034 | 0.8334 | 0.99961 |
| 9 | 96671698 | 98922040 | 1243 | -2.23E-05 | 4.21E-09 | 6.49E-05 | -0.34449 | 0.73048 | 0.99961 |
| 9 | 98922040 | 101592859 | 1560 | 2.39E-05 | 3.31E-09 | 5.75E-05 | 0.41599 | 0.67742 | 0.99961 |
| 9 | 101592859 | 104624437 | 2135 | -3.90E-06 | 3.56E-09 | 5.97E-05 | -0.06536 | 0.94789 | 0.99961 |
| 9 | 104624437 | 105967976 | 1512 | -9.20E-06 | 3.19E-09 | 5.65E-05 | -0.16273 | 0.87073 | 0.99961 |
| 9 | 105967976 | 107581749 | 1654 | -1.62E-06 | 3.64E-09 | 6.03E-05 | -0.02685 | 0.97858 | 0.99961 |
| 9 | 107581749 | 109298754 | 1238 | 8.70E-05 | 7.26E-09 | 8.52E-05 | 1.0211 | 0.30722 | 0.99961 |
| 9 | 109298754 | 110695062 | 1081 | 5.29E-05 | 3.60E-09 | 6.00E-05 | 0.88186 | 0.37785 | 0.99961 |
| 9 | 110695062 | 112778024 | 2285 | -4.47E-05 | 4.65E-09 | 6.82E-05 | -0.6546 | 0.51273 | 0.99961 |
| 9 | 112778024 | 114831082 | 1772 | 5.58E-06 | 4.23E-09 | 6.50E-05 | 0.085719 | 0.93169 | 0.99961 |
| 9 | 114831082 | 115693145 | 682 | 1.40E-06 | 2.24E-09 | 4.73E-05 | 0.029519 | 0.97645 | 0.99961 |
| 9 | 115693145 | 117019801 | 1191 | -8.34E-05 | 3.32E-09 | 5.76E-05 | -1.4473 | 0.14782 | 0.99961 |
| 9 | 117019801 | 117921960 | 891 | -6.91E-06 | 3.70E-09 | 6.08E-05 | -0.1136 | 0.90956 | 0.99961 |
| 9 | 117921960 | 121321537 | 3031 | -6.31E-06 | 3.66E-09 | 6.05E-05 | -0.10425 | 0.91697 | 0.99961 |
| 9 | 121321537 | 122260297 | 790 | -3.97E-07 | 2.52E-09 | 5.02E-05 | -0.0079 | 0.99369 | 0.99961 |
| 9 | 122260297 | 124871322 | 1583 | 1.01E-05 | 3.73E-09 | 6.11E-05 | 0.16552 | 0.86854 | 0.99961 |
| 9 | 124871322 | 126971887 | 1204 | -6.98E-06 | 3.26E-09 | 5.71E-05 | -0.12212 | 0.90281 | 0.99961 |
| 9 | 126971887 | 129059665 | 956 | 7.16E-05 | 4.38E-09 | 6.62E-05 | 1.0814 | 0.27951 | 0.99961 |
| 9 | 129059665 | 130055510 | 619 | -3.67E-06 | 2.65E-09 | 5.15E-05 | -0.07139 | 0.94309 | 0.99961 |
| 9 | 130055510 | 132165470 | 934 | 1.21E-05 | 4.01E-09 | 6.33E-05 | 0.19061 | 0.84883 | 0.99961 |
| 9 | 132165470 | 133223217 | 592 | 4.49E-05 | 3.10E-09 | 5.57E-05 | 0.80589 | 0.42031 | 0.99961 |
| 9 | 133223217 | 134127888 | 551 | -1.27E-05 | 2.98E-09 | 5.46E-05 | -0.23278 | 0.81593 | 0.99961 |
| 9 | 134127888 | 135298842 | 743 | -2.73E-05 | 3.21E-09 | 5.67E-05 | -0.48098 | 0.63053 | 0.99961 |
| 9 | 135298842 | 137041122 | 1177 | 1.28E-06 | 3.39E-09 | 5.82E-05 | 0.021928 | 0.98251 | 0.99961 |
| 9 | 137041122 | 137589364 | 371 | 1.47E-07 | 2.73E-09 | 5.22E-05 | 0.00281 | 0.99776 | 0.99961 |
| 9 | 137589364 | 138938978 | 1137 | 4.25E-05 | 3.00E-09 | 5.48E-05 | 0.77688 | 0.43723 | 0.99961 |
| 9 | 138938978 | 139500342 | 214 | 3.22E-05 | 1.59E-09 | 3.99E-05 | 0.80621 | 0.42012 | 0.99961 |
| 9 | 139500342 | 141144796 | 392 | 1.97E-05 | 2.30E-09 | 4.79E-05 | 0.41178 | 0.6805 | 0.99961 |
| 10 | 60523 | 751339 | 223 | 8.88E-06 | 1.12E-09 | 3.35E-05 | 0.2649 | 0.79109 | 0.99961 |
| 10 | 751339 | 1523544 | 618 | 1.43E-05 | 2.78E-09 | 5.27E-05 | 0.27043 | 0.78683 | 0.99961 |
| 10 | 1523544 | 2254500 | 936 | -1.75E-05 | 3.04E-09 | 5.52E-05 | -0.31698 | 0.75126 | 0.99961 |
| 10 | 2254500 | 3352699 | 1266 | 6.96E-06 | 3.28E-09 | 5.73E-05 | 0.12146 | 0.90333 | 0.99961 |
| 10 | 3352699 | 4572274 | 1235 | -1.89E-05 | 4.56E-09 | 6.75E-05 | -0.28032 | 0.77923 | 0.99961 |
| 10 | 4572274 | 5983762 | 1392 | 7.10E-06 | 3.90E-09 | 6.24E-05 | 0.11381 | 0.90939 | 0.99961 |
| 10 | 5983762 | 7171484 | 1234 | 4.45E-05 | 3.31E-09 | 5.76E-05 | 0.7737 | 0.43911 | 0.99961 |
| 10 | 7171484 | 8774665 | 1585 | 1.56E-05 | 3.47E-09 | 5.89E-05 | 0.26474 | 0.79121 | 0.99961 |
| 10 | 8774665 | 10249396 | 1225 | -1.59E-06 | 3.21E-09 | 5.67E-05 | -0.0281 | 0.97758 | 0.99961 |
| 10 | 10249396 | 12586797 | 1964 | -2.05E-05 | 3.13E-09 | 5.59E-05 | -0.36574 | 0.71456 | 0.99961 |
| 10 | 12586797 | 13321600 | 752 | 2.12E-05 | 3.51E-09 | 5.92E-05 | 0.35794 | 0.72039 | 0.99961 |
| 10 | 13321600 | 15026068 | 1654 | -3.37E-05 | 3.45E-09 | 5.87E-05 | -0.57447 | 0.56565 | 0.99961 |
| 10 | 15026068 | 16551767 | 1102 | -5.99E-06 | 3.24E-09 | 5.69E-05 | -0.10526 | 0.91617 | 0.99961 |
| 10 | 16551767 | 18537267 | 1519 | -1.96E-05 | 3.42E-09 | 5.85E-05 | -0.33449 | 0.73801 | 0.99961 |
| 10 | 18537267 | 19716878 | 1264 | 2.60E-05 | 2.96E-09 | 5.44E-05 | 0.47824 | 0.63248 | 0.99961 |
| 10 | 19716878 | 22772283 | 2039 | -3.05E-05 | 3.32E-09 | 5.76E-05 | -0.5283 | 0.59729 | 0.99961 |
| 10 | 22772283 | 23175132 | 275 | 2.06E-05 | 1.13E-09 | 3.36E-05 | 0.61271 | 0.54007 | 0.99961 |
| 10 | 23175132 | 25578596 | 1546 | -3.98E-05 | 3.51E-09 | 5.93E-05 | -0.67219 | 0.50146 | 0.99961 |
| 10 | 25578596 | 26888684 | 1267 | -4.49E-05 | 3.35E-09 | 5.79E-05 | -0.77498 | 0.43835 | 0.99961 |
| 10 | 26888684 | 29323236 | 1913 | 3.71E-06 | 4.05E-09 | 6.37E-05 | 0.058254 | 0.95355 | 0.99961 |
| 10 | 29323236 | 29783692 | 527 | 2.72E-05 | 2.10E-09 | 4.58E-05 | 0.59277 | 0.55333 | 0.99961 |
| 10 | 29783692 | 30458838 | 704 | 2.66E-05 | 3.26E-09 | 5.71E-05 | 0.46605 | 0.64118 | 0.99961 |
| 10 | 30458838 | 31907519 | 1024 | 4.43E-05 | 3.79E-09 | 6.16E-05 | 0.71946 | 0.47185 | 0.99961 |
| 10 | 31907519 | 33707968 | 1317 | 5.99E-05 | 4.48E-09 | 6.69E-05 | 0.89482 | 0.37089 | 0.99961 |
| 10 | 33707968 | 35109355 | 1044 | -6.07E-05 | 4.25E-09 | 6.52E-05 | -0.93171 | 0.35149 | 0.99961 |
| 10 | 35109355 | 36572508 | 906 | 3.88E-05 | 3.52E-09 | 5.94E-05 | 0.65391 | 0.51317 | 0.99961 |
| 10 | 36572508 | 37790029 | 745 | -3.84E-05 | 2.35E-09 | 4.84E-05 | -0.79252 | 0.42806 | 0.99961 |
| 10 | 37790029 | 42372579 | 551 | -4.92E-06 | 9.69E-10 | 3.11E-05 | -0.15812 | 0.87436 | 0.99961 |
| 10 | 42372579 | 43894771 | 672 | 7.90E-06 | 2.35E-09 | 4.85E-05 | 0.16288 | 0.87061 | 0.99961 |
| 10 | 43894771 | 44730075 | 688 | 2.21E-05 | 2.06E-09 | 4.53E-05 | 0.48847 | 0.62522 | 0.99961 |
| 10 | 44730075 | 48760450 | 1398 | 9.51E-06 | 3.15E-09 | 5.62E-05 | 0.16933 | 0.86553 | 0.99961 |
| 10 | 48760450 | 50840915 | 1311 | 2.97E-05 | 3.17E-09 | 5.63E-05 | 0.52752 | 0.59783 | 0.99961 |
| 10 | 50840915 | 53616629 | 1477 | -1.82E-05 | 3.39E-09 | 5.82E-05 | -0.31262 | 0.75457 | 0.99961 |
| 10 | 53616629 | 55103572 | 1476 | -1.73E-05 | 3.08E-09 | 5.55E-05 | -0.31207 | 0.75498 | 0.99961 |
| 10 | 55103572 | 57031140 | 1943 | -3.12E-05 | 3.07E-09 | 5.54E-05 | -0.56389 | 0.57283 | 0.99961 |
| 10 | 57031140 | 57662146 | 511 | 1.83E-05 | 1.17E-09 | 3.42E-05 | 0.53362 | 0.5936 | 0.99961 |
| 10 | 57662146 | 58959528 | 1216 | -7.85E-06 | 1.72E-09 | 4.14E-05 | -0.18948 | 0.84972 | 0.99961 |
| 10 | 58959528 | 60289987 | 1113 | 2.98E-05 | 2.49E-09 | 4.99E-05 | 0.59802 | 0.54983 | 0.99961 |
| 10 | 60289987 | 61891409 | 1148 | 3.35E-05 | 3.90E-09 | 6.25E-05 | 0.53596 | 0.59199 | 0.99961 |
| 10 | 61891409 | 62659679 | 664 | -2.51E-05 | 2.34E-09 | 4.84E-05 | -0.51859 | 0.60404 | 0.99961 |
| 10 | 62659679 | 63341695 | 329 | 6.10E-07 | 1.25E-09 | 3.54E-05 | 0.017221 | 0.98626 | 0.99961 |
| 10 | 63341695 | 65794114 | 1771 | -5.69E-06 | 4.69E-09 | 6.85E-05 | -0.08306 | 0.9338 | 0.99961 |
| 10 | 65794114 | 67549615 | 1252 | 1.16E-05 | 3.08E-09 | 5.55E-05 | 0.20841 | 0.83491 | 0.99961 |
| 10 | 67549615 | 69900148 | 2025 | -6.52E-05 | 3.24E-09 | 5.69E-05 | -1.146 | 0.2518 | 0.99961 |
| 10 | 69900148 | 70195991 | 197 | -3.45E-06 | 7.95E-10 | 2.82E-05 | -0.12246 | 0.90253 | 0.99961 |
| 10 | 70195991 | 72220850 | 1529 | 5.00E-05 | 3.19E-09 | 5.65E-05 | 0.88519 | 0.37606 | 0.99961 |
| 10 | 72220850 | 73508512 | 1356 | 3.09E-05 | 3.15E-09 | 5.61E-05 | 0.55082 | 0.58175 | 0.99961 |
| 10 | 73508512 | 75422550 | 711 | 2.86E-05 | 2.23E-09 | 4.73E-05 | 0.60399 | 0.54585 | 0.99961 |
| 10 | 75422550 | 78706814 | 1600 | -5.41E-05 | 4.23E-09 | 6.51E-05 | -0.8308 | 0.40608 | 0.99961 |
| 10 | 78706814 | 80876749 | 1907 | -3.32E-05 | 3.41E-09 | 5.84E-05 | -0.56946 | 0.56904 | 0.99961 |
| 10 | 80876749 | 82414679 | 973 | -5.42E-05 | 3.90E-09 | 6.25E-05 | -0.86778 | 0.38551 | 0.99961 |
| 10 | 82414679 | 84383745 | 1581 | 1.39E-05 | 3.13E-09 | 5.59E-05 | 0.24933 | 0.80311 | 0.99961 |
| 10 | 84383745 | 85458703 | 1137 | -1.77E-05 | 2.33E-09 | 4.83E-05 | -0.36655 | 0.71395 | 0.99961 |
| 10 | 85458703 | 87937577 | 2188 | -9.30E-06 | 3.09E-09 | 5.56E-05 | -0.16733 | 0.86711 | 0.99961 |
| 10 | 87937577 | 89127064 | 486 | 1.64E-05 | 2.13E-09 | 4.62E-05 | 0.35534 | 0.72233 | 0.99961 |
| 10 | 89127064 | 91013381 | 1402 | 2.89E-05 | 3.90E-09 | 6.24E-05 | 0.4622 | 0.64394 | 0.99961 |
| 10 | 91013381 | 92006581 | 831 | -4.32E-06 | 2.47E-09 | 4.97E-05 | -0.08706 | 0.93062 | 0.99961 |
| 10 | 92006581 | 93335047 | 1001 | -3.76E-06 | 2.75E-09 | 5.25E-05 | -0.07173 | 0.94281 | 0.99961 |
| 10 | 93335047 | 95396368 | 1170 | 1.14E-05 | 4.47E-09 | 6.69E-05 | 0.17117 | 0.86409 | 0.99961 |
| 10 | 95396368 | 96221243 | 532 | 7.13E-05 | 2.51E-09 | 5.01E-05 | 1.4241 | 0.15442 | 0.99961 |
| 10 | 96221243 | 97822357 | 1045 | 2.48E-06 | 2.97E-09 | 5.45E-05 | 0.045552 | 0.96367 | 0.99961 |
| 10 | 97822357 | 100241302 | 1525 | 8.00E-06 | 3.27E-09 | 5.71E-05 | 0.14 | 0.88866 | 0.99961 |
| 10 | 100241302 | 100668400 | 335 | 1.68E-05 | 1.13E-09 | 3.36E-05 | 0.50042 | 0.61678 | 0.99961 |
| 10 | 100668400 | 102949239 | 1301 | -1.81E-06 | 3.65E-09 | 6.04E-05 | -0.03003 | 0.97605 | 0.99961 |
| 10 | 102949239 | 104380410 | 504 | -9.74E-06 | 2.49E-09 | 4.99E-05 | -0.19522 | 0.84522 | 0.99961 |
| 10 | 104380410 | 106695048 | 1396 | -3.59E-05 | 4.22E-09 | 6.49E-05 | -0.5527 | 0.58047 | 0.99961 |
| 10 | 106695048 | 108726686 | 1610 | -3.60E-05 | 3.34E-09 | 5.78E-05 | -0.62348 | 0.53297 | 0.99961 |
| 10 | 108726686 | 110317705 | 1461 | -1.76E-05 | 2.90E-09 | 5.39E-05 | -0.32751 | 0.74328 | 0.99961 |
| 10 | 110317705 | 112561493 | 1619 | 1.20E-05 | 3.57E-09 | 5.97E-05 | 0.20077 | 0.84088 | 0.99961 |
| 10 | 112561493 | 115328432 | 1981 | 7.41E-05 | 3.74E-09 | 6.11E-05 | 1.2112 | 0.22581 | 0.99961 |
| 10 | 115328432 | 116421406 | 916 | -3.22E-05 | 3.66E-09 | 6.05E-05 | -0.53277 | 0.59419 | 0.99961 |
| 10 | 116421406 | 119523934 | 2023 | 2.20E-05 | 3.82E-09 | 6.18E-05 | 0.3562 | 0.72169 | 0.99961 |
| 10 | 119523934 | 120591353 | 753 | -9.43E-06 | 3.27E-09 | 5.72E-05 | -0.16488 | 0.86904 | 0.99961 |
| 10 | 120591353 | 122407323 | 1253 | -2.26E-05 | 3.41E-09 | 5.84E-05 | -0.38663 | 0.69903 | 0.99961 |
| 10 | 122407323 | 123231465 | 738 | 0.00011119 | 5.26E-09 | 7.25E-05 | 1.5335 | 0.12515 | 0.99961 |
| 10 | 123231465 | 123900545 | 494 | 2.66E-05 | 2.65E-09 | 5.15E-05 | 0.51661 | 0.60543 | 0.99961 |
| 10 | 123900545 | 125869346 | 1481 | 2.67E-05 | 3.23E-09 | 5.68E-05 | 0.46958 | 0.63865 | 0.99961 |
| 10 | 125869346 | 128001098 | 1829 | -1.13E-05 | 3.44E-09 | 5.86E-05 | -0.19265 | 0.84723 | 0.99961 |
| 10 | 128001098 | 129154362 | 946 | -2.49E-05 | 3.16E-09 | 5.62E-05 | -0.44313 | 0.65767 | 0.99961 |
| 10 | 129154362 | 130844284 | 1706 | -7.99E-06 | 3.09E-09 | 5.56E-05 | -0.14366 | 0.88577 | 0.99961 |
| 10 | 130844284 | 131741327 | 823 | 4.79E-05 | 3.60E-09 | 6.00E-05 | 0.79793 | 0.42491 | 0.99961 |
| 10 | 131741327 | 132580245 | 892 | 1.71E-05 | 3.28E-09 | 5.73E-05 | 0.2986 | 0.76524 | 0.99961 |
| 10 | 132580245 | 134336329 | 1736 | -2.15E-05 | 2.89E-09 | 5.38E-05 | -0.39923 | 0.68972 | 0.99961 |
| 10 | 134336329 | 134494673 | 59 | -9.62E-06 | 4.72E-10 | 2.17E-05 | -0.44292 | 0.65782 | 0.99961 |
| 10 | 134494673 | 135523865 | 464 | -3.90E-05 | 2.36E-09 | 4.86E-05 | -0.8027 | 0.42215 | 0.99961 |
| 11 | 70855 | 1213590 | 401 | 6.99E-06 | 2.78E-09 | 5.27E-05 | 0.1326 | 0.89451 | 0.99961 |
| 11 | 1213590 | 3665481 | 1428 | 6.31E-06 | 3.48E-09 | 5.90E-05 | 0.10695 | 0.91483 | 0.99961 |
| 11 | 3665481 | 4746709 | 843 | -9.83E-06 | 3.06E-09 | 5.53E-05 | -0.17766 | 0.85899 | 0.99961 |
| 11 | 4746709 | 6322869 | 2118 | 1.52E-05 | 3.21E-09 | 5.67E-05 | 0.26755 | 0.78904 | 0.99961 |
| 11 | 6322869 | 7436701 | 1006 | -6.15E-06 | 3.11E-09 | 5.57E-05 | -0.11026 | 0.9122 | 0.99961 |
| 11 | 7436701 | 8333274 | 832 | -2.27E-05 | 3.16E-09 | 5.62E-05 | -0.40399 | 0.68622 | 0.99961 |
| 11 | 8333274 | 9087317 | 493 | 4.25E-05 | 2.19E-09 | 4.68E-05 | 0.90798 | 0.36389 | 0.99961 |
| 11 | 9087317 | 10952027 | 1188 | 1.47E-05 | 4.03E-09 | 6.35E-05 | 0.23197 | 0.81656 | 0.99961 |
| 11 | 10952027 | 12564229 | 1961 | 5.07E-05 | 3.53E-09 | 5.94E-05 | 0.85437 | 0.3929 | 0.99961 |
| 11 | 12564229 | 13373124 | 787 | 9.70E-06 | 3.28E-09 | 5.73E-05 | 0.16939 | 0.86549 | 0.99961 |
| 11 | 13373124 | 15742552 | 1651 | -1.52E-05 | 4.57E-09 | 6.76E-05 | -0.22479 | 0.82214 | 0.99961 |
| 11 | 15742552 | 17578402 | 1124 | -1.34E-05 | 3.30E-09 | 5.74E-05 | -0.2326 | 0.81607 | 0.99961 |
| 11 | 17578402 | 19569535 | 1468 | 6.67E-05 | 3.56E-09 | 5.96E-05 | 1.1191 | 0.26311 | 0.99961 |
| 11 | 19569535 | 21017138 | 1384 | -2.80E-05 | 3.15E-09 | 5.61E-05 | -0.49977 | 0.61724 | 0.99961 |
| 11 | 21017138 | 22374142 | 1182 | -1.35E-05 | 3.32E-09 | 5.76E-05 | -0.23378 | 0.81515 | 0.99961 |
| 11 | 22374142 | 24088517 | 1486 | -2.23E-05 | 3.17E-09 | 5.63E-05 | -0.39644 | 0.69178 | 0.99961 |
| 11 | 24088517 | 26045753 | 1713 | 4.60E-06 | 3.45E-09 | 5.88E-05 | 0.078243 | 0.93763 | 0.99961 |
| 11 | 26045753 | 27020461 | 811 | -1.14E-05 | 3.10E-09 | 5.57E-05 | -0.20429 | 0.83813 | 0.99961 |
| 11 | 27020461 | 28481593 | 770 | -3.98E-05 | 2.30E-09 | 4.79E-05 | -0.83122 | 0.40585 | 0.99961 |
| 11 | 28481593 | 30141357 | 973 | -2.10E-05 | 2.79E-09 | 5.29E-05 | -0.39768 | 0.69087 | 0.99961 |
| 11 | 30141357 | 32276901 | 1301 | 6.45E-05 | 3.63E-09 | 6.03E-05 | 1.0697 | 0.28477 | 0.99961 |
| 11 | 32276901 | 33958739 | 1008 | -1.07E-05 | 3.47E-09 | 5.89E-05 | -0.18137 | 0.85608 | 0.99961 |
| 11 | 33958739 | 35502605 | 1568 | -2.80E-05 | 3.16E-09 | 5.62E-05 | -0.49859 | 0.61807 | 0.99961 |
| 11 | 35502605 | 37116815 | 1358 | 9.58E-05 | 3.90E-09 | 6.25E-05 | 1.5337 | 0.12511 | 0.99961 |
| 11 | 37116815 | 39382003 | 1495 | 1.13E-05 | 2.96E-09 | 5.44E-05 | 0.20758 | 0.83556 | 0.99961 |
| 11 | 39382003 | 42310003 | 2229 | 2.17E-06 | 3.08E-09 | 5.55E-05 | 0.039167 | 0.96876 | 0.99961 |
| 11 | 42310003 | 44693799 | 1925 | -2.37E-05 | 2.93E-09 | 5.41E-05 | -0.43896 | 0.66069 | 0.99961 |
| 11 | 44693799 | 47006137 | 1312 | -5.82E-05 | 3.46E-09 | 5.89E-05 | -0.9892 | 0.32257 | 0.99961 |
| 11 | 47006137 | 49866050 | 1050 | -3.91E-06 | 2.77E-09 | 5.26E-05 | -0.07435 | 0.94074 | 0.99961 |
| 11 | 49866050 | 54695473 | 430 | 1.98E-05 | 8.46E-10 | 2.91E-05 | 0.68036 | 0.49628 | 0.99961 |
| 11 | 54695473 | 55082657 | 29 | 1.95E-06 | 2.08E-10 | 1.44E-05 | 0.13479 | 0.89278 | 0.99961 |
| 11 | 55082657 | 58457495 | 2685 | -3.69E-05 | 3.54E-09 | 5.95E-05 | -0.6192 | 0.53579 | 0.99961 |
| 11 | 58457495 | 58780549 | 179 | -1.67E-05 | 5.24E-10 | 2.29E-05 | -0.7295 | 0.46569 | 0.99961 |
| 11 | 58780549 | 62223771 | 1720 | 4.03E-05 | 4.02E-09 | 6.34E-05 | 0.63527 | 0.52525 | 0.99961 |
| 11 | 62223771 | 63804569 | 467 | 6.83E-06 | 3.89E-09 | 6.23E-05 | 0.10957 | 0.91275 | 0.99961 |
| 11 | 63804569 | 65898631 | 765 | 4.21E-05 | 6.41E-09 | 8.01E-05 | 0.52549 | 0.59924 | 0.99961 |
| 11 | 65898631 | 68005825 | 490 | 1.91E-05 | 2.97E-09 | 5.45E-05 | 0.35045 | 0.726 | 0.99961 |
| 11 | 68005825 | 69516130 | 868 | 0.00010743 | 6.40E-09 | 8.00E-05 | 1.3427 | 0.17938 | 0.99961 |
| 11 | 69516130 | 70926292 | 861 | -4.28E-05 | 3.53E-09 | 5.94E-05 | -0.72054 | 0.47119 | 0.99961 |
| 11 | 70926292 | 72286017 | 602 | 1.70E-05 | 1.93E-09 | 4.40E-05 | 0.38586 | 0.6996 | 0.99961 |
| 11 | 72286017 | 74412351 | 1009 | -8.40E-06 | 3.38E-09 | 5.81E-05 | -0.14443 | 0.88516 | 0.99961 |
| 11 | 74412351 | 76797209 | 1305 | -2.38E-06 | 3.24E-09 | 5.69E-05 | -0.04187 | 0.96661 | 0.99961 |
| 11 | 76797209 | 78355058 | 787 | -1.96E-05 | 2.39E-09 | 4.89E-05 | -0.40047 | 0.68881 | 0.99961 |
| 11 | 78355058 | 80722373 | 2087 | -2.06E-05 | 3.41E-09 | 5.84E-05 | -0.35341 | 0.72378 | 0.99961 |
| 11 | 80722373 | 81266712 | 636 | 2.77E-06 | 2.33E-09 | 4.83E-05 | 0.057358 | 0.95426 | 0.99961 |
| 11 | 81266712 | 84381272 | 2556 | 5.40E-05 | 3.60E-09 | 6.00E-05 | 0.90091 | 0.36763 | 0.99961 |
| 11 | 84381272 | 86619301 | 1524 | -2.40E-05 | 3.50E-09 | 5.92E-05 | -0.40583 | 0.68487 | 0.99961 |
| 11 | 86619301 | 87430235 | 726 | -3.49E-05 | 2.42E-09 | 4.92E-05 | -0.70969 | 0.47789 | 0.99961 |
| 11 | 87430235 | 89208936 | 1175 | -1.58E-05 | 3.21E-09 | 5.67E-05 | -0.27957 | 0.77981 | 0.99961 |
| 11 | 89208936 | 90966490 | 984 | 1.65E-05 | 2.38E-09 | 4.87E-05 | 0.3387 | 0.73483 | 0.99961 |
| 11 | 90966490 | 92077144 | 833 | 7.39E-06 | 2.25E-09 | 4.74E-05 | 0.1558 | 0.87619 | 0.99961 |
| 11 | 92077144 | 93276157 | 803 | 1.30E-05 | 2.29E-09 | 4.79E-05 | 0.27207 | 0.78557 | 0.99961 |
| 11 | 93276157 | 94242260 | 549 | -1.51E-05 | 1.58E-09 | 3.97E-05 | -0.38023 | 0.70377 | 0.99961 |
| 11 | 94242260 | 95726041 | 1147 | 2.01E-05 | 3.17E-09 | 5.63E-05 | 0.35663 | 0.72137 | 0.99961 |
| 11 | 95726041 | 97057058 | 945 | -4.14E-05 | 3.32E-09 | 5.76E-05 | -0.71851 | 0.47244 | 0.99961 |
| 11 | 97057058 | 98705065 | 1361 | 2.88E-05 | 2.82E-09 | 5.31E-05 | 0.54363 | 0.5867 | 0.99961 |
| 11 | 98705065 | 100417169 | 1754 | 3.88E-05 | 3.27E-09 | 5.72E-05 | 0.67907 | 0.49709 | 0.99961 |
| 11 | 100417169 | 101331121 | 952 | 3.30E-05 | 3.19E-09 | 5.65E-05 | 0.58354 | 0.55953 | 0.99961 |
| 11 | 101331121 | 103959636 | 2447 | 5.00E-05 | 3.25E-09 | 5.70E-05 | 0.87692 | 0.38053 | 0.99961 |
| 11 | 103959636 | 104873284 | 656 | -1.10E-05 | 1.39E-09 | 3.72E-05 | -0.29468 | 0.76824 | 0.99961 |
| 11 | 104873284 | 106520894 | 1136 | 1.56E-05 | 2.48E-09 | 4.98E-05 | 0.31368 | 0.75377 | 0.99961 |
| 11 | 106520894 | 107844200 | 1002 | -3.84E-06 | 3.43E-09 | 5.86E-05 | -0.06553 | 0.94775 | 0.99961 |
| 11 | 107844200 | 108437037 | 219 | 2.78E-06 | 7.94E-10 | 2.82E-05 | 0.098819 | 0.92128 | 0.99961 |
| 11 | 108437037 | 109866116 | 720 | -3.55E-05 | 2.63E-09 | 5.12E-05 | -0.69382 | 0.4878 | 0.99961 |
| 11 | 109866116 | 111114397 | 997 | -3.09E-06 | 3.44E-09 | 5.86E-05 | -0.05262 | 0.95803 | 0.99961 |
| 11 | 111114397 | 112459488 | 700 | 7.25E-05 | 4.10E-09 | 6.40E-05 | 1.1331 | 0.25716 | 0.99961 |
| 11 | 112459488 | 114257728 | 1507 | -2.35E-05 | 3.44E-09 | 5.87E-05 | -0.40065 | 0.68868 | 0.99961 |
| 11 | 114257728 | 114830666 | 500 | 3.26E-05 | 1.40E-09 | 3.74E-05 | 0.87024 | 0.38417 | 0.99961 |
| 11 | 114830666 | 116383348 | 1165 | -3.92E-05 | 3.35E-09 | 5.79E-05 | -0.67618 | 0.49892 | 0.99961 |
| 11 | 116383348 | 117747110 | 1031 | -3.77E-05 | 3.27E-09 | 5.72E-05 | -0.65987 | 0.50934 | 0.99961 |
| 11 | 117747110 | 119215476 | 839 | 6.32E-05 | 3.73E-09 | 6.11E-05 | 1.0346 | 0.30087 | 0.99961 |
| 11 | 119215476 | 120766806 | 1076 | -5.73E-06 | 3.36E-09 | 5.79E-05 | -0.09893 | 0.9212 | 0.99961 |
| 11 | 120766806 | 121175943 | 399 | 3.59E-05 | 1.42E-09 | 3.77E-05 | 0.95125 | 0.34148 | 0.99961 |
| 11 | 121175943 | 122591910 | 922 | 2.50E-05 | 3.76E-09 | 6.13E-05 | 0.4082 | 0.68313 | 0.99961 |
| 11 | 122591910 | 123500117 | 654 | -4.25E-06 | 3.62E-09 | 6.01E-05 | -0.07073 | 0.94361 | 0.99961 |
| 11 | 123500117 | 124495528 | 1006 | 5.46E-05 | 2.85E-09 | 5.34E-05 | 1.0217 | 0.30694 | 0.99961 |
| 11 | 124495528 | 126311320 | 1395 | -3.55E-05 | 3.32E-09 | 5.76E-05 | -0.61562 | 0.53815 | 0.99961 |
| 11 | 126311320 | 127316679 | 1039 | 9.47E-06 | 3.36E-09 | 5.80E-05 | 0.16331 | 0.87028 | 0.99961 |
| 11 | 127316679 | 128188537 | 635 | -9.63E-06 | 2.41E-09 | 4.91E-05 | -0.19617 | 0.84447 | 0.99961 |
| 11 | 128188537 | 130342575 | 1446 | 2.76E-05 | 3.39E-09 | 5.83E-05 | 0.4735 | 0.63586 | 0.99961 |
| 11 | 130342575 | 131074612 | 719 | -3.24E-05 | 3.02E-09 | 5.50E-05 | -0.58989 | 0.55527 | 0.99961 |
| 11 | 131074612 | 133000046 | 1982 | 3.03E-05 | 3.11E-09 | 5.58E-05 | 0.54327 | 0.58695 | 0.99961 |
| 11 | 133000046 | 134205993 | 899 | -1.07E-05 | 3.08E-09 | 5.55E-05 | -0.19275 | 0.84716 | 0.99961 |
| 11 | 134205993 | 134946452 | 830 | -1.45E-05 | 3.02E-09 | 5.49E-05 | -0.26312 | 0.79245 | 0.99961 |
| 12 | 61107 | 1080331 | 521 | -1.39E-05 | 2.94E-09 | 5.42E-05 | -0.25712 | 0.79709 | 0.99961 |
| 12 | 1080331 | 2544786 | 822 | 3.79E-05 | 3.53E-09 | 5.94E-05 | 0.63805 | 0.52344 | 0.99961 |
| 12 | 2544786 | 3677037 | 879 | -1.39E-05 | 3.15E-09 | 5.61E-05 | -0.24762 | 0.80443 | 0.99961 |
| 12 | 3677037 | 4417679 | 630 | 4.31E-05 | 2.99E-09 | 5.47E-05 | 0.78786 | 0.43078 | 0.99961 |
| 12 | 4417679 | 5321472 | 866 | -1.55E-05 | 3.36E-09 | 5.79E-05 | -0.26812 | 0.78861 | 0.99961 |
| 12 | 5321472 | 6419753 | 993 | -4.22E-05 | 3.43E-09 | 5.85E-05 | -0.72039 | 0.47129 | 0.99961 |
| 12 | 6419753 | 8377536 | 626 | -8.98E-05 | 3.35E-09 | 5.79E-05 | -1.551 | 0.1209 | 0.99961 |
| 12 | 8377536 | 9031395 | 137 | 2.49E-05 | 9.98E-10 | 3.16E-05 | 0.7896 | 0.42976 | 0.99961 |
| 12 | 9031395 | 10481534 | 862 | 4.73E-05 | 3.88E-09 | 6.23E-05 | 0.75999 | 0.44726 | 0.99961 |
| 12 | 10481534 | 11754846 | 735 | 4.11E-06 | 2.27E-09 | 4.77E-05 | 0.086219 | 0.93129 | 0.99961 |
| 12 | 11754846 | 12733528 | 730 | 7.42E-06 | 3.18E-09 | 5.64E-05 | 0.13166 | 0.89525 | 0.99961 |
| 12 | 12733528 | 15241484 | 1901 | -3.45E-06 | 3.54E-09 | 5.95E-05 | -0.05808 | 0.95368 | 0.99961 |
| 12 | 15241484 | 16310460 | 468 | -7.33E-06 | 1.67E-09 | 4.09E-05 | -0.17905 | 0.8579 | 0.99961 |
| 12 | 16310460 | 18087873 | 1279 | 3.28E-05 | 3.58E-09 | 5.98E-05 | 0.54786 | 0.58379 | 0.99961 |
| 12 | 18087873 | 20863943 | 2205 | -3.02E-05 | 3.65E-09 | 6.04E-05 | -0.50024 | 0.6169 | 0.99961 |
| 12 | 20863943 | 23820634 | 2133 | 2.56E-05 | 3.23E-09 | 5.69E-05 | 0.45036 | 0.65245 | 0.99961 |
| 12 | 23820634 | 25371083 | 1420 | 8.04E-06 | 3.08E-09 | 5.55E-05 | 0.14491 | 0.88478 | 0.99961 |
| 12 | 25371083 | 27799773 | 2097 | -5.70E-05 | 7.33E-09 | 8.56E-05 | -0.66585 | 0.50551 | 0.99961 |
| 12 | 27799773 | 29651255 | 1522 | -2.56E-05 | 3.55E-09 | 5.95E-05 | -0.43007 | 0.66714 | 0.99961 |
| 12 | 29651255 | 30512406 | 1156 | -2.20E-05 | 3.18E-09 | 5.64E-05 | -0.38997 | 0.69656 | 0.99961 |
| 12 | 30512406 | 32165592 | 1306 | -7.67E-05 | 3.54E-09 | 5.95E-05 | -1.2897 | 0.19715 | 0.99961 |
| 12 | 32165592 | 33076989 | 828 | -6.91E-05 | 3.12E-09 | 5.59E-05 | -1.2372 | 0.21601 | 0.99961 |
| 12 | 33076989 | 37856717 | 1023 | -2.16E-05 | 2.40E-09 | 4.89E-05 | -0.44186 | 0.65859 | 0.99961 |
| 12 | 37856717 | 39227169 | 456 | -2.37E-05 | 1.53E-09 | 3.91E-05 | -0.60484 | 0.54529 | 0.99961 |
| 12 | 39227169 | 40816185 | 1288 | -3.75E-05 | 3.00E-09 | 5.48E-05 | -0.6841 | 0.49391 | 0.99961 |
| 12 | 40816185 | 41658586 | 805 | 2.53E-05 | 2.17E-09 | 4.66E-05 | 0.54257 | 0.58742 | 0.99961 |
| 12 | 41658586 | 43984474 | 2105 | 7.66E-06 | 3.43E-09 | 5.85E-05 | 0.13089 | 0.89586 | 0.99961 |
| 12 | 43984474 | 46024229 | 1100 | 2.55E-05 | 3.62E-09 | 6.01E-05 | 0.4249 | 0.67091 | 0.99961 |
| 12 | 46024229 | 47714793 | 910 | 4.60E-05 | 3.47E-09 | 5.89E-05 | 0.78015 | 0.4353 | 0.99961 |
| 12 | 47714793 | 49001866 | 1022 | 4.13E-05 | 3.47E-09 | 5.89E-05 | 0.70185 | 0.48277 | 0.99961 |
| 12 | 49001866 | 51776494 | 1022 | -5.81E-05 | 3.73E-09 | 6.11E-05 | -0.95139 | 0.34141 | 0.99961 |
| 12 | 51776494 | 53039004 | 1079 | 4.92E-05 | 3.91E-09 | 6.25E-05 | 0.78685 | 0.43137 | 0.99961 |
| 12 | 53039004 | 54778823 | 985 | 4.50E-05 | 6.59E-09 | 8.12E-05 | 0.55389 | 0.57965 | 0.99961 |
| 12 | 54778823 | 55665837 | 588 | 5.23E-05 | 2.09E-09 | 4.57E-05 | 1.1442 | 0.25255 | 0.99961 |
| 12 | 55665837 | 57548860 | 661 | 7.32E-05 | 3.19E-09 | 5.65E-05 | 1.2956 | 0.19512 | 0.99961 |
| 12 | 57548860 | 59308666 | 855 | -3.01E-05 | 3.12E-09 | 5.58E-05 | -0.53856 | 0.59019 | 0.99961 |
| 12 | 59308666 | 61123729 | 1075 | 2.59E-05 | 3.11E-09 | 5.58E-05 | 0.46384 | 0.64277 | 0.99961 |
| 12 | 61123729 | 64032461 | 2325 | 4.38E-05 | 3.64E-09 | 6.04E-05 | 0.7258 | 0.46796 | 0.99961 |
| 12 | 64032461 | 65559695 | 716 | -4.31E-05 | 3.11E-09 | 5.57E-05 | -0.77261 | 0.43975 | 0.99961 |
| 12 | 65559695 | 67181144 | 995 | 1.48E-05 | 4.23E-09 | 6.50E-05 | 0.22782 | 0.81979 | 0.99961 |
| 12 | 67181144 | 67909729 | 705 | -2.27E-06 | 2.03E-09 | 4.51E-05 | -0.05039 | 0.95981 | 0.99961 |
| 12 | 67909729 | 69826542 | 1470 | -1.79E-05 | 3.25E-09 | 5.70E-05 | -0.31382 | 0.75366 | 0.99961 |
| 12 | 69826542 | 70957987 | 1097 | 6.50E-06 | 2.90E-09 | 5.39E-05 | 0.12058 | 0.90402 | 0.99961 |
| 12 | 70957987 | 72645075 | 1177 | 1.15E-05 | 3.49E-09 | 5.91E-05 | 0.19391 | 0.84625 | 0.99961 |
| 12 | 72645075 | 73818454 | 732 | -2.51E-05 | 2.35E-09 | 4.85E-05 | -0.51703 | 0.60514 | 0.99961 |
| 12 | 73818454 | 76511314 | 1745 | 1.12E-06 | 3.29E-09 | 5.74E-05 | 0.019519 | 0.98443 | 0.99961 |
| 12 | 76511314 | 78570570 | 1615 | 3.50E-06 | 3.77E-09 | 6.14E-05 | 0.057082 | 0.95448 | 0.99961 |
| 12 | 78570570 | 80448150 | 917 | 1.38E-05 | 2.99E-09 | 5.47E-05 | 0.25325 | 0.80008 | 0.99961 |
| 12 | 80448150 | 81467767 | 740 | -6.92E-06 | 2.10E-09 | 4.59E-05 | -0.15084 | 0.8801 | 0.99961 |
| 12 | 81467767 | 83502666 | 1296 | -1.07E-05 | 3.71E-09 | 6.09E-05 | -0.17532 | 0.86083 | 0.99961 |
| 12 | 83502666 | 84303844 | 617 | -5.13E-06 | 1.60E-09 | 3.99E-05 | -0.1284 | 0.89783 | 0.99961 |
| 12 | 84303844 | 85990426 | 1033 | 1.84E-05 | 2.69E-09 | 5.19E-05 | 0.35443 | 0.72302 | 0.99961 |
| 12 | 85990426 | 89682122 | 1941 | -3.15E-05 | 3.22E-09 | 5.67E-05 | -0.5554 | 0.57862 | 0.99961 |
| 12 | 89682122 | 92066155 | 1507 | -8.48E-05 | 3.70E-09 | 6.08E-05 | -1.3935 | 0.16347 | 0.99961 |
| 12 | 92066155 | 94514567 | 1782 | -3.60E-05 | 3.97E-09 | 6.30E-05 | -0.57177 | 0.56748 | 0.99961 |
| 12 | 94514567 | 96020785 | 1146 | 7.46E-07 | 3.39E-09 | 5.82E-05 | 0.012819 | 0.98977 | 0.99961 |
| 12 | 96020785 | 97108839 | 846 | 3.00E-06 | 3.45E-09 | 5.87E-05 | 0.051142 | 0.95921 | 0.99961 |
| 12 | 97108839 | 99305987 | 1749 | 2.00E-05 | 4.19E-09 | 6.47E-05 | 0.30933 | 0.75707 | 0.99961 |
| 12 | 99305987 | 101447641 | 1366 | 1.52E-05 | 3.18E-09 | 5.64E-05 | 0.26932 | 0.78768 | 0.99961 |
| 12 | 101447641 | 101862690 | 268 | 2.99E-05 | 1.17E-09 | 3.42E-05 | 0.87561 | 0.38124 | 0.99961 |
| 12 | 101862690 | 102964986 | 618 | 8.90E-06 | 2.55E-09 | 5.05E-05 | 0.17626 | 0.86009 | 0.99961 |
| 12 | 102964986 | 104848696 | 1444 | 1.49E-05 | 3.34E-09 | 5.78E-05 | 0.25804 | 0.79638 | 0.99961 |
| 12 | 104848696 | 106436213 | 1455 | -1.02E-05 | 3.26E-09 | 5.71E-05 | -0.17832 | 0.85847 | 0.99961 |
| 12 | 106436213 | 106958748 | 412 | -4.05E-05 | 2.08E-09 | 4.56E-05 | -0.88795 | 0.37457 | 0.99961 |
| 12 | 106958748 | 109025901 | 1360 | 3.51E-05 | 4.47E-09 | 6.68E-05 | 0.5258 | 0.59903 | 0.99961 |
| 12 | 109025901 | 110336719 | 708 | -3.54E-05 | 2.66E-09 | 5.16E-05 | -0.68729 | 0.4919 | 0.99961 |
| 12 | 110336719 | 113263518 | 689 | 1.92E-05 | 2.75E-09 | 5.24E-05 | 0.36549 | 0.71474 | 0.99961 |
| 12 | 113263518 | 113986709 | 376 | -7.60E-06 | 2.23E-09 | 4.72E-05 | -0.16116 | 0.87197 | 0.99961 |
| 12 | 113986709 | 115036602 | 1042 | -8.02E-06 | 3.55E-09 | 5.96E-05 | -0.13449 | 0.89301 | 0.99961 |
| 12 | 115036602 | 115503216 | 369 | 9.71E-05 | 2.04E-09 | 4.51E-05 | 2.1526 | 0.031354 | 0.99961 |
| 12 | 115503216 | 117087471 | 1262 | -1.06E-05 | 3.59E-09 | 5.99E-05 | -0.17655 | 0.85987 | 0.99961 |
| 12 | 117087471 | 118135375 | 799 | -1.55E-05 | 3.38E-09 | 5.82E-05 | -0.26706 | 0.78943 | 0.99961 |
| 12 | 118135375 | 119754110 | 1219 | -2.39E-06 | 3.09E-09 | 5.56E-05 | -0.04307 | 0.96565 | 0.99961 |
| 12 | 119754110 | 122007651 | 1132 | -1.05E-05 | 3.64E-09 | 6.03E-05 | -0.17426 | 0.86166 | 0.99961 |
| 12 | 122007651 | 124977980 | 1114 | -0.00016723 | 1.54E-08 | 0.0001242 | -1.3468 | 0.17804 | 0.99961 |
| 12 | 124977980 | 126445505 | 1112 | 1.58E-05 | 3.54E-09 | 5.95E-05 | 0.26501 | 0.791 | 0.99961 |
| 12 | 126445505 | 127875098 | 1323 | 1.34E-05 | 3.06E-09 | 5.53E-05 | 0.24275 | 0.8082 | 0.99961 |
| 12 | 127875098 | 129237297 | 1303 | 2.73E-05 | 3.34E-09 | 5.78E-05 | 0.47257 | 0.63652 | 0.99961 |
| 12 | 129237297 | 130377921 | 1215 | -4.80E-08 | 3.21E-09 | 5.67E-05 | -0.00085 | 0.99932 | 0.99961 |
| 12 | 130377921 | 131786751 | 1347 | 4.50E-05 | 3.82E-09 | 6.18E-05 | 0.72785 | 0.4667 | 0.99961 |
| 12 | 131786751 | 132807034 | 603 | 2.29E-05 | 2.56E-09 | 5.06E-05 | 0.45241 | 0.65097 | 0.99961 |
| 12 | 132807034 | 133841511 | 319 | 4.67E-06 | 2.03E-09 | 4.50E-05 | 0.10374 | 0.91737 | 0.99961 |
| 13 | 19020013 | 20686720 | 705 | 2.36E-05 | 2.28E-09 | 4.77E-05 | 0.49512 | 0.62051 | 0.99961 |
| 13 | 20686720 | 22242174 | 1091 | -2.03E-05 | 3.12E-09 | 5.59E-05 | -0.36278 | 0.71677 | 0.99961 |
| 13 | 22242174 | 23594702 | 1236 | -3.70E-06 | 3.29E-09 | 5.74E-05 | -0.06443 | 0.94863 | 0.99961 |
| 13 | 23594702 | 24531937 | 980 | 7.76E-06 | 3.16E-09 | 5.62E-05 | 0.1381 | 0.89016 | 0.99961 |
| 13 | 24531937 | 25784362 | 1187 | 4.07E-05 | 3.10E-09 | 5.57E-05 | 0.73113 | 0.4647 | 0.99961 |
| 13 | 25784362 | 27284362 | 1130 | -2.45E-05 | 3.09E-09 | 5.56E-05 | -0.44033 | 0.6597 | 0.99961 |
| 13 | 27284362 | 29257551 | 1574 | -4.87E-05 | 3.56E-09 | 5.97E-05 | -0.81533 | 0.41488 | 0.99961 |
| 13 | 29257551 | 30483267 | 1031 | 2.34E-05 | 3.03E-09 | 5.50E-05 | 0.42425 | 0.67138 | 0.99961 |
| 13 | 30483267 | 32301815 | 1472 | 1.80E-05 | 3.10E-09 | 5.57E-05 | 0.32359 | 0.74625 | 0.99961 |
| 13 | 32301815 | 33776903 | 1156 | -3.18E-05 | 3.76E-09 | 6.13E-05 | -0.51863 | 0.60402 | 0.99961 |
| 13 | 33776903 | 35026464 | 968 | -3.83E-06 | 3.14E-09 | 5.60E-05 | -0.06838 | 0.94548 | 0.99961 |
| 13 | 35026464 | 36046367 | 467 | -1.27E-05 | 2.19E-09 | 4.68E-05 | -0.27139 | 0.78609 | 0.99961 |
| 13 | 36046367 | 37498185 | 1254 | 2.26E-05 | 3.45E-09 | 5.88E-05 | 0.38409 | 0.70091 | 0.99961 |
| 13 | 37498185 | 38878163 | 1376 | -9.19E-06 | 3.15E-09 | 5.61E-05 | -0.16393 | 0.86978 | 0.99961 |
| 13 | 38878163 | 41069263 | 1764 | -2.74E-05 | 3.36E-09 | 5.79E-05 | -0.47355 | 0.63582 | 0.99961 |
| 13 | 41069263 | 43101027 | 1263 | 5.01E-05 | 3.70E-09 | 6.09E-05 | 0.8238 | 0.41005 | 0.99961 |
| 13 | 43101027 | 44901409 | 1595 | -3.10E-05 | 3.40E-09 | 5.83E-05 | -0.53154 | 0.59504 | 0.99961 |
| 13 | 44901409 | 46495214 | 963 | -6.48E-06 | 3.02E-09 | 5.49E-05 | -0.11798 | 0.90608 | 0.99961 |
| 13 | 46495214 | 47431079 | 689 | 1.30E-06 | 2.29E-09 | 4.78E-05 | 0.027156 | 0.97833 | 0.99961 |
| 13 | 47431079 | 49383962 | 1380 | -3.22E-05 | 3.15E-09 | 5.61E-05 | -0.57427 | 0.56578 | 0.99961 |
| 13 | 49383962 | 51591091 | 1401 | 0.00014381 | 6.55E-09 | 8.09E-05 | 1.7769 | 0.075577 | 0.99961 |
| 13 | 51591091 | 53339622 | 689 | -1.67E-05 | 3.46E-09 | 5.88E-05 | -0.28439 | 0.77611 | 0.99961 |
| 13 | 53339622 | 54682864 | 1144 | -1.39E-05 | 3.15E-09 | 5.62E-05 | -0.24743 | 0.80458 | 0.99961 |
| 13 | 54682864 | 55817131 | 848 | 4.39E-07 | 2.21E-09 | 4.70E-05 | 0.009358 | 0.99253 | 0.99961 |
| 13 | 55817131 | 57554217 | 925 | -3.62E-05 | 1.78E-09 | 4.21E-05 | -0.8594 | 0.39012 | 0.99961 |
| 13 | 57554217 | 58410626 | 540 | 1.13E-05 | 1.22E-09 | 3.50E-05 | 0.32377 | 0.74611 | 0.99961 |
| 13 | 58410626 | 59302271 | 661 | 8.87E-06 | 1.44E-09 | 3.80E-05 | 0.23369 | 0.81522 | 0.99961 |
| 13 | 59302271 | 60854778 | 1279 | -5.88E-07 | 3.10E-09 | 5.57E-05 | -0.01054 | 0.99159 | 0.99961 |
| 13 | 60854778 | 61591949 | 675 | 6.66E-06 | 1.67E-09 | 4.09E-05 | 0.16296 | 0.87055 | 0.99961 |
| 13 | 61591949 | 63971559 | 1845 | 1.45E-05 | 3.28E-09 | 5.73E-05 | 0.25361 | 0.79979 | 0.99961 |
| 13 | 63971559 | 65200602 | 1057 | 1.25E-05 | 2.02E-09 | 4.50E-05 | 0.27872 | 0.78046 | 0.99961 |
| 13 | 65200602 | 67844453 | 1663 | -8.78E-06 | 3.17E-09 | 5.63E-05 | -0.15593 | 0.87608 | 0.99961 |
| 13 | 67844453 | 70724516 | 2536 | -2.20E-05 | 3.42E-09 | 5.85E-05 | -0.3768 | 0.70632 | 0.99961 |
| 13 | 70724516 | 71526791 | 703 | 1.00E-05 | 3.04E-09 | 5.52E-05 | 0.18151 | 0.85597 | 0.99961 |
| 13 | 71526791 | 73934089 | 1737 | 8.89E-05 | 3.65E-09 | 6.04E-05 | 1.4704 | 0.14146 | 0.99961 |
| 13 | 73934089 | 75670143 | 1461 | 2.45E-05 | 3.49E-09 | 5.91E-05 | 0.41461 | 0.67843 | 0.99961 |
| 13 | 75670143 | 77410555 | 1622 | -9.62E-06 | 3.52E-09 | 5.93E-05 | -0.16218 | 0.87117 | 0.99961 |
| 13 | 77410555 | 78808813 | 973 | -2.19E-05 | 2.94E-09 | 5.43E-05 | -0.40445 | 0.68588 | 0.99961 |
| 13 | 78808813 | 80585676 | 1214 | -1.24E-05 | 3.38E-09 | 5.81E-05 | -0.2126 | 0.83164 | 0.99961 |
| 13 | 80585676 | 82581814 | 1528 | 2.10E-05 | 3.68E-09 | 6.07E-05 | 0.34617 | 0.72921 | 0.99961 |
| 13 | 82581814 | 84133144 | 1073 | 3.60E-05 | 2.21E-09 | 4.70E-05 | 0.76615 | 0.44359 | 0.99961 |
| 13 | 84133144 | 85610916 | 1020 | 2.76E-05 | 2.74E-09 | 5.24E-05 | 0.52767 | 0.59773 | 0.99961 |
| 13 | 85610916 | 88559882 | 2126 | 1.58E-05 | 3.35E-09 | 5.79E-05 | 0.27217 | 0.78549 | 0.99961 |
| 13 | 88559882 | 90521311 | 1310 | -4.03E-05 | 2.91E-09 | 5.40E-05 | -0.74719 | 0.45495 | 0.99961 |
| 13 | 90521311 | 93129691 | 2051 | 7.12E-05 | 3.40E-09 | 5.83E-05 | 1.2209 | 0.22211 | 0.99961 |
| 13 | 93129691 | 93586455 | 285 | -2.37E-05 | 1.11E-09 | 3.34E-05 | -0.70846 | 0.47866 | 0.99961 |
| 13 | 93586455 | 96087558 | 2138 | -8.88E-06 | 3.20E-09 | 5.65E-05 | -0.15708 | 0.87518 | 0.99961 |
| 13 | 96087558 | 97519210 | 757 | -8.10E-06 | 2.26E-09 | 4.75E-05 | -0.17048 | 0.86464 | 0.99961 |
| 13 | 97519210 | 98938919 | 819 | -1.01E-05 | 3.41E-09 | 5.84E-05 | -0.17228 | 0.86322 | 0.99961 |
| 13 | 98938919 | 100574095 | 1496 | 3.15E-05 | 3.61E-09 | 6.01E-05 | 0.52429 | 0.60008 | 0.99961 |
| 13 | 100574095 | 102300523 | 1503 | -1.54E-06 | 3.47E-09 | 5.89E-05 | -0.02619 | 0.97911 | 0.99961 |
| 13 | 102300523 | 104066710 | 1818 | 1.32E-05 | 3.07E-09 | 5.54E-05 | 0.23857 | 0.81144 | 0.99961 |
| 13 | 104066710 | 104844114 | 729 | 1.58E-05 | 2.31E-09 | 4.81E-05 | 0.32891 | 0.74222 | 0.99961 |
| 13 | 104844114 | 106247554 | 1429 | -4.60E-06 | 3.14E-09 | 5.60E-05 | -0.0821 | 0.93457 | 0.99961 |
| 13 | 106247554 | 106750000 | 593 | -1.39E-05 | 1.94E-09 | 4.41E-05 | -0.31595 | 0.75204 | 0.99961 |
| 13 | 106750000 | 108189877 | 1234 | 4.17E-05 | 3.11E-09 | 5.57E-05 | 0.74894 | 0.45389 | 0.99961 |
| 13 | 108189877 | 109121210 | 926 | -2.42E-05 | 3.17E-09 | 5.63E-05 | -0.42883 | 0.66805 | 0.99961 |
| 13 | 109121210 | 109815112 | 736 | -1.44E-05 | 2.50E-09 | 5.00E-05 | -0.28792 | 0.77341 | 0.99961 |
| 13 | 109815112 | 111232073 | 1324 | -6.82E-05 | 3.72E-09 | 6.10E-05 | -1.1188 | 0.26321 | 0.99961 |
| 13 | 111232073 | 112247592 | 823 | -2.36E-05 | 3.14E-09 | 5.60E-05 | -0.42183 | 0.67315 | 0.99961 |
| 13 | 112247592 | 113572488 | 895 | 2.66E-05 | 2.93E-09 | 5.41E-05 | 0.49122 | 0.62327 | 0.99961 |
| 13 | 113572488 | 115109853 | 594 | 4.99E-05 | 2.88E-09 | 5.36E-05 | 0.93081 | 0.35195 | 0.99961 |
| 14 | 19002084 | 21589402 | 968 | -8.90E-06 | 3.13E-09 | 5.60E-05 | -0.15898 | 0.87369 | 0.99961 |
| 14 | 21589402 | 23018665 | 1408 | 7.08E-05 | 3.17E-09 | 5.63E-05 | 1.2572 | 0.20868 | 0.99961 |
| 14 | 23018665 | 24905123 | 1163 | 6.46E-05 | 3.69E-09 | 6.08E-05 | 1.0639 | 0.28736 | 0.99961 |
| 14 | 24905123 | 26135686 | 1223 | -8.01E-06 | 3.40E-09 | 5.83E-05 | -0.13734 | 0.89077 | 0.99961 |
| 14 | 26135686 | 28466533 | 1597 | 2.73E-05 | 3.09E-09 | 5.56E-05 | 0.49079 | 0.62357 | 0.99961 |
| 14 | 28466533 | 29972145 | 879 | -1.95E-05 | 2.84E-09 | 5.33E-05 | -0.3667 | 0.71384 | 0.99961 |
| 14 | 29972145 | 32383265 | 1182 | -9.26E-06 | 3.49E-09 | 5.90E-05 | -0.15679 | 0.87541 | 0.99961 |
| 14 | 32383265 | 34846251 | 2140 | -7.08E-05 | 3.31E-09 | 5.75E-05 | -1.2303 | 0.21858 | 0.99961 |
| 14 | 34846251 | 35859593 | 455 | -2.74E-05 | 2.21E-09 | 4.70E-05 | -0.58394 | 0.55926 | 0.99961 |
| 14 | 35859593 | 38667725 | 1595 | -6.15E-06 | 3.30E-09 | 5.74E-05 | -0.10719 | 0.91464 | 0.99961 |
| 14 | 38667725 | 40341990 | 1124 | -2.82E-05 | 3.13E-09 | 5.60E-05 | -0.50296 | 0.61499 | 0.99961 |
| 14 | 40341990 | 41615502 | 1064 | 1.81E-05 | 2.07E-09 | 4.55E-05 | 0.39859 | 0.69019 | 0.99961 |
| 14 | 41615502 | 43137389 | 942 | 4.41E-06 | 2.51E-09 | 5.01E-05 | 0.088025 | 0.92986 | 0.99961 |
| 14 | 43137389 | 44543227 | 929 | 1.78E-05 | 2.78E-09 | 5.28E-05 | 0.33797 | 0.73539 | 0.99961 |
| 14 | 44543227 | 46081896 | 780 | 2.27E-05 | 2.33E-09 | 4.82E-05 | 0.47062 | 0.63791 | 0.99961 |
| 14 | 46081896 | 46749130 | 508 | 1.80E-05 | 1.15E-09 | 3.39E-05 | 0.53065 | 0.59566 | 0.99961 |
| 14 | 46749130 | 48573586 | 1507 | -4.31E-05 | 3.16E-09 | 5.62E-05 | -0.76675 | 0.44323 | 0.99961 |
| 14 | 48573586 | 49005471 | 285 | -6.74E-06 | 8.53E-10 | 2.92E-05 | -0.23064 | 0.81759 | 0.99961 |
| 14 | 49005471 | 50507760 | 957 | -1.29E-05 | 3.00E-09 | 5.48E-05 | -0.2351 | 0.81413 | 0.99961 |
| 14 | 50507760 | 51493572 | 681 | 6.19E-05 | 2.74E-09 | 5.23E-05 | 1.182 | 0.23722 | 0.99961 |
| 14 | 51493572 | 53474383 | 1583 | 3.75E-05 | 4.03E-09 | 6.35E-05 | 0.59119 | 0.5544 | 0.99961 |
| 14 | 53474383 | 53997831 | 390 | -2.07E-05 | 1.36E-09 | 3.69E-05 | -0.5622 | 0.57398 | 0.99961 |
| 14 | 53997831 | 55233681 | 773 | -1.34E-05 | 3.67E-09 | 6.06E-05 | -0.22163 | 0.8246 | 0.99961 |
| 14 | 55233681 | 56216880 | 558 | -1.44E-06 | 2.05E-09 | 4.53E-05 | -0.0319 | 0.97455 | 0.99961 |
| 14 | 56216880 | 57481262 | 1280 | 1.04E-05 | 3.08E-09 | 5.55E-05 | 0.18706 | 0.85162 | 0.99961 |
| 14 | 57481262 | 59448336 | 1409 | -1.63E-05 | 4.10E-09 | 6.40E-05 | -0.25377 | 0.79967 | 0.99961 |
| 14 | 59448336 | 61680424 | 1381 | -6.42E-05 | 3.95E-09 | 6.29E-05 | -1.0204 | 0.30755 | 0.99961 |
| 14 | 61680424 | 63790015 | 1616 | 4.20E-05 | 3.44E-09 | 5.86E-05 | 0.71588 | 0.47406 | 0.99961 |
| 14 | 63790015 | 65220298 | 647 | 1.32E-05 | 2.82E-09 | 5.31E-05 | 0.24925 | 0.80317 | 0.99961 |
| 14 | 65220298 | 66399555 | 750 | 6.60E-06 | 2.93E-09 | 5.41E-05 | 0.12189 | 0.90299 | 0.99961 |
| 14 | 66399555 | 67992317 | 678 | 2.34E-05 | 2.07E-09 | 4.55E-05 | 0.51365 | 0.6075 | 0.99961 |
| 14 | 67992317 | 71131957 | 2177 | 3.26E-05 | 3.54E-09 | 5.95E-05 | 0.54787 | 0.58378 | 0.99961 |
| 14 | 71131957 | 72889615 | 1404 | 5.39E-05 | 3.88E-09 | 6.23E-05 | 0.86469 | 0.38721 | 0.99961 |
| 14 | 72889615 | 76444767 | 2024 | 3.17E-05 | 3.94E-09 | 6.28E-05 | 0.50513 | 0.61346 | 0.99961 |
| 14 | 76444767 | 77229472 | 609 | -4.25E-05 | 2.70E-09 | 5.19E-05 | -0.81906 | 0.41275 | 0.99961 |
| 14 | 77229472 | 78561945 | 1080 | 1.61E-06 | 3.40E-09 | 5.83E-05 | 0.027563 | 0.97801 | 0.99961 |
| 14 | 78561945 | 80186579 | 1205 | -1.01E-05 | 3.19E-09 | 5.65E-05 | -0.17922 | 0.85776 | 0.99961 |
| 14 | 80186579 | 81579714 | 1145 | -1.29E-05 | 3.22E-09 | 5.67E-05 | -0.22779 | 0.81981 | 0.99961 |
| 14 | 81579714 | 83138097 | 1027 | 1.57E-05 | 3.17E-09 | 5.63E-05 | 0.27954 | 0.77983 | 0.99961 |
| 14 | 83138097 | 84241633 | 928 | -3.19E-05 | 2.80E-09 | 5.29E-05 | -0.60402 | 0.54583 | 0.99961 |
| 14 | 84241633 | 85746319 | 1126 | 4.56E-05 | 3.64E-09 | 6.03E-05 | 0.7565 | 0.44935 | 0.99961 |
| 14 | 85746319 | 86647002 | 627 | -4.37E-06 | 2.22E-09 | 4.72E-05 | -0.09273 | 0.92612 | 0.99961 |
| 14 | 86647002 | 87635341 | 705 | 1.91E-05 | 2.31E-09 | 4.80E-05 | 0.39707 | 0.69132 | 0.99961 |
| 14 | 87635341 | 89497643 | 1312 | -2.43E-05 | 3.24E-09 | 5.69E-05 | -0.42668 | 0.66962 | 0.99961 |
| 14 | 89497643 | 91296860 | 1279 | -3.20E-05 | 3.25E-09 | 5.70E-05 | -0.5613 | 0.57459 | 0.99961 |
| 14 | 91296860 | 93132299 | 1250 | 2.23E-05 | 4.46E-09 | 6.68E-05 | 0.33399 | 0.73839 | 0.99961 |
| 14 | 93132299 | 94325285 | 741 | -3.99E-06 | 3.02E-09 | 5.50E-05 | -0.07267 | 0.94207 | 0.99961 |
| 14 | 94325285 | 95750867 | 1521 | -2.28E-05 | 3.15E-09 | 5.62E-05 | -0.40543 | 0.68516 | 0.99961 |
| 14 | 95750867 | 96262415 | 666 | -6.17E-06 | 2.80E-09 | 5.30E-05 | -0.11653 | 0.90723 | 0.99961 |
| 14 | 96262415 | 98341162 | 1534 | 9.30E-06 | 3.31E-09 | 5.75E-05 | 0.16165 | 0.87158 | 0.99961 |
| 14 | 98341162 | 99138532 | 844 | 2.84E-05 | 3.57E-09 | 5.97E-05 | 0.47629 | 0.63386 | 0.99961 |
| 14 | 99138532 | 101534307 | 1560 | 2.97E-06 | 3.36E-09 | 5.79E-05 | 0.051246 | 0.95913 | 0.99961 |
| 14 | 101534307 | 103012102 | 730 | 2.36E-05 | 3.21E-09 | 5.67E-05 | 0.41667 | 0.67692 | 0.99961 |
| 14 | 103012102 | 105001723 | 889 | -4.28E-05 | 3.72E-09 | 6.10E-05 | -0.7014 | 0.48306 | 0.99961 |
| 14 | 105001723 | 106340358 | 270 | 3.80E-05 | 1.60E-09 | 4.00E-05 | 0.94963 | 0.3423 | 0.99961 |
| 14 | 106340358 | 107289454 | 210 | 1.64E-05 | 1.11E-09 | 3.33E-05 | 0.49152 | 0.62306 | 0.99961 |
| 15 | 20001200 | 21131604 | 7 | 1.83E-07 | 1.60E-10 | 1.27E-05 | 0.014409 | 0.9885 | 0.99961 |
| 15 | 21131604 | 24195127 | 544 | -1.26E-05 | 2.36E-09 | 4.85E-05 | -0.25967 | 0.79512 | 0.99961 |
| 15 | 24195127 | 25108590 | 613 | -2.39E-05 | 2.06E-09 | 4.54E-05 | -0.52643 | 0.59859 | 0.99961 |
| 15 | 25108590 | 27298112 | 1629 | 1.06E-05 | 3.08E-09 | 5.55E-05 | 0.19109 | 0.84845 | 0.99961 |
| 15 | 27298112 | 29338502 | 1006 | -8.11E-06 | 3.18E-09 | 5.64E-05 | -0.14376 | 0.88569 | 0.99961 |
| 15 | 29338502 | 30165568 | 572 | 2.47E-05 | 2.25E-09 | 4.74E-05 | 0.52167 | 0.6019 | 0.99961 |
| 15 | 30165568 | 31591821 | 511 | -5.27E-06 | 2.54E-09 | 5.04E-05 | -0.10445 | 0.91681 | 0.99961 |
| 15 | 31591821 | 32441811 | 480 | 4.04E-05 | 2.44E-09 | 4.94E-05 | 0.8182 | 0.41324 | 0.99961 |
| 15 | 32441811 | 34015425 | 1291 | -2.23E-06 | 3.20E-09 | 5.66E-05 | -0.03937 | 0.96859 | 0.99961 |
| 15 | 34015425 | 35083251 | 689 | 7.99E-06 | 2.66E-09 | 5.16E-05 | 0.1548 | 0.87698 | 0.99961 |
| 15 | 35083251 | 37456502 | 1832 | 4.81E-05 | 3.56E-09 | 5.97E-05 | 0.80497 | 0.42084 | 0.99961 |
| 15 | 37456502 | 38530777 | 720 | -5.56E-05 | 3.25E-09 | 5.70E-05 | -0.97382 | 0.33014 | 0.99961 |
| 15 | 38530777 | 40384132 | 1573 | -1.48E-05 | 4.30E-09 | 6.56E-05 | -0.22551 | 0.82158 | 0.99961 |
| 15 | 40384132 | 41177514 | 397 | -2.37E-05 | 3.03E-09 | 5.51E-05 | -0.43019 | 0.66706 | 0.99961 |
| 15 | 41177514 | 42776399 | 760 | -4.12E-05 | 4.20E-09 | 6.48E-05 | -0.63554 | 0.52508 | 0.99961 |
| 15 | 42776399 | 44198049 | 409 | 1.31E-05 | 1.47E-09 | 3.83E-05 | 0.34205 | 0.73231 | 0.99961 |
| 15 | 44198049 | 46618787 | 938 | 5.08E-05 | 3.34E-09 | 5.78E-05 | 0.87878 | 0.37952 | 0.99961 |
| 15 | 46618787 | 48136048 | 1306 | 4.06E-06 | 3.58E-09 | 5.98E-05 | 0.067802 | 0.94594 | 0.99961 |
| 15 | 48136048 | 50008043 | 926 | -5.77E-06 | 2.88E-09 | 5.37E-05 | -0.10752 | 0.91438 | 0.99961 |
| 15 | 50008043 | 51677560 | 1095 | -1.29E-05 | 3.35E-09 | 5.79E-05 | -0.22207 | 0.82426 | 0.99961 |
| 15 | 51677560 | 53069096 | 759 | -6.19E-06 | 3.10E-09 | 5.57E-05 | -0.11111 | 0.91153 | 0.99961 |
| 15 | 53069096 | 54508528 | 1334 | -2.63E-05 | 3.25E-09 | 5.70E-05 | -0.46186 | 0.64418 | 0.99961 |
| 15 | 54508528 | 55136269 | 568 | -3.64E-05 | 2.32E-09 | 4.82E-05 | -0.75605 | 0.44962 | 0.99961 |
| 15 | 55136269 | 56901773 | 1064 | 5.07E-05 | 4.46E-09 | 6.68E-05 | 0.7585 | 0.44815 | 0.99961 |
| 15 | 56901773 | 58441366 | 1213 | -3.82E-05 | 3.64E-09 | 6.03E-05 | -0.63338 | 0.52648 | 0.99961 |
| 15 | 58441366 | 59694116 | 891 | -4.60E-05 | 3.65E-09 | 6.04E-05 | -0.76125 | 0.44651 | 0.99961 |
| 15 | 59694116 | 61265836 | 1177 | 3.32E-05 | 3.30E-09 | 5.74E-05 | 0.57862 | 0.56285 | 0.99961 |
| 15 | 61265836 | 63215222 | 1632 | -2.97E-06 | 3.79E-09 | 6.16E-05 | -0.04821 | 0.96155 | 0.99961 |
| 15 | 63215222 | 65104668 | 889 | 9.21E-06 | 3.97E-09 | 6.30E-05 | 0.14615 | 0.88381 | 0.99961 |
| 15 | 65104668 | 67094767 | 994 | 4.52E-05 | 3.69E-09 | 6.08E-05 | 0.74382 | 0.45699 | 0.99961 |
| 15 | 67094767 | 69017999 | 1250 | 3.99E-05 | 3.81E-09 | 6.17E-05 | 0.64757 | 0.51726 | 0.99961 |
| 15 | 69017999 | 70133341 | 543 | -3.69E-06 | 2.38E-09 | 4.87E-05 | -0.07577 | 0.9396 | 0.99961 |
| 15 | 70133341 | 71922649 | 1201 | -1.63E-05 | 3.61E-09 | 6.01E-05 | -0.27206 | 0.78557 | 0.99961 |
| 15 | 71922649 | 73628714 | 716 | -4.34E-05 | 2.43E-09 | 4.93E-05 | -0.88073 | 0.37846 | 0.99961 |
| 15 | 73628714 | 76398624 | 967 | -4.32E-05 | 3.94E-09 | 6.28E-05 | -0.68755 | 0.49174 | 0.99961 |
| 15 | 76398624 | 78516053 | 1042 | -2.99E-05 | 3.56E-09 | 5.97E-05 | -0.50093 | 0.61642 | 0.99961 |
| 15 | 78516053 | 80860978 | 1844 | 5.47E-06 | 3.46E-09 | 5.88E-05 | 0.093034 | 0.92588 | 0.99961 |
| 15 | 80860978 | 84260468 | 1468 | 2.39E-05 | 3.56E-09 | 5.97E-05 | 0.40094 | 0.68847 | 0.99961 |
| 15 | 84260468 | 86652905 | 1571 | 1.87E-05 | 3.57E-09 | 5.98E-05 | 0.31233 | 0.75479 | 0.99961 |
| 15 | 86652905 | 88369943 | 1465 | -2.23E-05 | 3.17E-09 | 5.63E-05 | -0.39574 | 0.6923 | 0.99961 |
| 15 | 88369943 | 90475551 | 1228 | -2.46E-05 | 3.37E-09 | 5.80E-05 | -0.42369 | 0.67179 | 0.99961 |
| 15 | 90475551 | 92164392 | 1128 | 2.92E-05 | 3.41E-09 | 5.84E-05 | 0.50013 | 0.61698 | 0.99961 |
| 15 | 92164392 | 93780902 | 1341 | -1.92E-05 | 3.31E-09 | 5.75E-05 | -0.33306 | 0.73909 | 0.99961 |
| 15 | 93780902 | 94924330 | 1116 | 4.43E-05 | 3.44E-09 | 5.86E-05 | 0.75583 | 0.44975 | 0.99961 |
| 15 | 94924330 | 96141596 | 1060 | -2.58E-05 | 3.59E-09 | 5.99E-05 | -0.4311 | 0.66639 | 0.99961 |
| 15 | 96141596 | 98030910 | 1334 | -2.11E-05 | 3.51E-09 | 5.93E-05 | -0.35596 | 0.72187 | 0.99961 |
| 15 | 98030910 | 99244059 | 1053 | -1.30E-05 | 3.31E-09 | 5.75E-05 | -0.22562 | 0.8215 | 0.99961 |
| 15 | 99244059 | 100636847 | 1011 | -5.82E-05 | 3.84E-09 | 6.20E-05 | -0.93911 | 0.34768 | 0.99961 |
| 15 | 100636847 | 101552694 | 673 | -2.50E-05 | 3.45E-09 | 5.87E-05 | -0.42624 | 0.66993 | 0.99961 |
| 15 | 101552694 | 102520966 | 694 | 2.17E-05 | 3.39E-09 | 5.82E-05 | 0.37325 | 0.70896 | 0.99961 |
| 16 | 60054 | 1207206 | 451 | -2.58E-06 | 3.52E-09 | 5.93E-05 | -0.04341 | 0.96538 | 0.99961 |
| 16 | 1207206 | 2764829 | 584 | 1.42E-06 | 3.62E-09 | 6.02E-05 | 0.023571 | 0.98119 | 0.99961 |
| 16 | 2764829 | 4001196 | 448 | 1.44E-05 | 3.06E-09 | 5.53E-05 | 0.26016 | 0.79474 | 0.99961 |
| 16 | 4001196 | 5118345 | 571 | 0.00011168 | 4.63E-09 | 6.80E-05 | 1.6415 | 0.1007 | 0.99961 |
| 16 | 5118345 | 5891580 | 665 | 4.54E-05 | 3.18E-09 | 5.64E-05 | 0.80518 | 0.42071 | 0.99961 |
| 16 | 5891580 | 6893551 | 1176 | 2.77E-05 | 2.92E-09 | 5.40E-05 | 0.51349 | 0.60761 | 0.99961 |
| 16 | 6893551 | 7529121 | 894 | 4.29E-05 | 3.67E-09 | 6.06E-05 | 0.70731 | 0.47938 | 0.99961 |
| 16 | 7529121 | 8338519 | 999 | 3.62E-06 | 3.33E-09 | 5.77E-05 | 0.062757 | 0.94996 | 0.99961 |
| 16 | 8338519 | 9799419 | 1305 | 4.02E-05 | 3.65E-09 | 6.04E-05 | 0.66534 | 0.50583 | 0.99961 |
| 16 | 9799419 | 10426032 | 729 | -7.71E-06 | 2.42E-09 | 4.92E-05 | -0.15675 | 0.87545 | 0.99961 |
| 16 | 10426032 | 11520161 | 750 | 4.98E-06 | 4.12E-09 | 6.42E-05 | 0.077548 | 0.93819 | 0.99961 |
| 16 | 11520161 | 12613098 | 683 | -2.29E-05 | 3.90E-09 | 6.24E-05 | -0.36641 | 0.71406 | 0.99961 |
| 16 | 12613098 | 13154437 | 612 | 8.43E-06 | 2.53E-09 | 5.03E-05 | 0.16749 | 0.86699 | 0.99961 |
| 16 | 13154437 | 14464002 | 1057 | 2.95E-05 | 3.12E-09 | 5.59E-05 | 0.52874 | 0.59698 | 0.99961 |
| 16 | 14464002 | 16154060 | 417 | 5.28E-06 | 2.70E-09 | 5.20E-05 | 0.10158 | 0.91909 | 0.99961 |
| 16 | 16154060 | 17644749 | 662 | 4.05E-05 | 3.19E-09 | 5.65E-05 | 0.71791 | 0.47282 | 0.99961 |
| 16 | 17644749 | 18643607 | 342 | 1.72E-05 | 1.69E-09 | 4.11E-05 | 0.41972 | 0.67469 | 0.99961 |
| 16 | 18643607 | 20150571 | 752 | -2.90E-06 | 3.48E-09 | 5.90E-05 | -0.04913 | 0.96082 | 0.99961 |
| 16 | 20150571 | 22448904 | 725 | 1.66E-05 | 3.06E-09 | 5.53E-05 | 0.29995 | 0.76421 | 0.99961 |
| 16 | 22448904 | 24136875 | 903 | 9.64E-06 | 3.07E-09 | 5.54E-05 | 0.17396 | 0.8619 | 0.99961 |
| 16 | 24136875 | 25986862 | 1298 | -2.83E-05 | 2.97E-09 | 5.45E-05 | -0.51894 | 0.6038 | 0.99961 |
| 16 | 25986862 | 27445755 | 1162 | 3.73E-06 | 3.18E-09 | 5.64E-05 | 0.066166 | 0.94725 | 0.99961 |
| 16 | 27445755 | 29036613 | 496 | 2.06E-05 | 1.99E-09 | 4.46E-05 | 0.46199 | 0.64409 | 0.99961 |
| 16 | 29036613 | 31382943 | 425 | 6.40E-05 | 2.78E-09 | 5.27E-05 | 1.2142 | 0.22467 | 0.99961 |
| 16 | 31382943 | 46399473 | 217 | 1.24E-06 | 1.22E-09 | 3.49E-05 | 0.035672 | 0.97154 | 0.99961 |
| 16 | 46399473 | 49007926 | 590 | 6.04E-06 | 2.08E-09 | 4.56E-05 | 0.13245 | 0.89463 | 0.99961 |
| 16 | 49007926 | 52035823 | 1937 | 6.40E-05 | 3.33E-09 | 5.77E-05 | 1.1092 | 0.26735 | 0.99961 |
| 16 | 52035823 | 53382572 | 866 | 2.32E-05 | 2.93E-09 | 5.41E-05 | 0.42783 | 0.66878 | 0.99961 |
| 16 | 53382572 | 55903774 | 2115 | 2.73E-05 | 3.82E-09 | 6.18E-05 | 0.44219 | 0.65835 | 0.99961 |
| 16 | 55903774 | 57664330 | 1124 | 1.65E-05 | 3.45E-09 | 5.87E-05 | 0.28184 | 0.77806 | 0.99961 |
| 16 | 57664330 | 59045405 | 863 | 7.76E-06 | 3.15E-09 | 5.61E-05 | 0.13818 | 0.8901 | 0.99961 |
| 16 | 59045405 | 60745694 | 1079 | -3.12E-05 | 3.39E-09 | 5.82E-05 | -0.53574 | 0.59214 | 0.99961 |
| 16 | 60745694 | 62229849 | 787 | 2.45E-05 | 2.09E-09 | 4.57E-05 | 0.53596 | 0.59199 | 0.99961 |
| 16 | 62229849 | 63691589 | 973 | 4.92E-06 | 3.13E-09 | 5.59E-05 | 0.087976 | 0.9299 | 0.99961 |
| 16 | 63691589 | 65938566 | 1683 | 1.98E-05 | 3.42E-09 | 5.85E-05 | 0.33902 | 0.73459 | 0.99961 |
| 16 | 65938566 | 68841363 | 1163 | -1.44E-05 | 4.42E-09 | 6.65E-05 | -0.21722 | 0.82804 | 0.99961 |
| 16 | 68841363 | 71054028 | 599 | 4.83E-05 | 2.96E-09 | 5.44E-05 | 0.88751 | 0.37481 | 0.99961 |
| 16 | 71054028 | 72935150 | 739 | -1.30E-05 | 2.94E-09 | 5.42E-05 | -0.24022 | 0.81016 | 0.99961 |
| 16 | 72935150 | 74971503 | 1237 | 1.62E-05 | 3.42E-09 | 5.85E-05 | 0.27748 | 0.78141 | 0.99961 |
| 16 | 74971503 | 75977954 | 436 | 1.86E-05 | 2.27E-09 | 4.76E-05 | 0.39028 | 0.69633 | 0.99961 |
| 16 | 75977954 | 77523678 | 1382 | -5.75E-05 | 3.54E-09 | 5.95E-05 | -0.96628 | 0.33391 | 0.99961 |
| 16 | 77523678 | 78456292 | 1102 | -6.80E-05 | 3.36E-09 | 5.80E-05 | -1.1722 | 0.24113 | 0.99961 |
| 16 | 78456292 | 79134815 | 925 | 1.64E-05 | 3.25E-09 | 5.70E-05 | 0.2883 | 0.77312 | 0.99961 |
| 16 | 79134815 | 80297374 | 1312 | 1.43E-05 | 3.37E-09 | 5.81E-05 | 0.24548 | 0.80608 | 0.99961 |
| 16 | 80297374 | 81772536 | 1373 | 7.33E-05 | 5.69E-09 | 7.55E-05 | 0.97147 | 0.33131 | 0.99961 |
| 16 | 81772536 | 82747728 | 983 | 2.76E-05 | 3.18E-09 | 5.64E-05 | 0.49017 | 0.62401 | 0.99961 |
| 16 | 82747728 | 83477208 | 1042 | -1.23E-05 | 3.49E-09 | 5.90E-05 | -0.20815 | 0.83511 | 0.99961 |
| 16 | 83477208 | 84445883 | 1264 | -1.53E-05 | 3.19E-09 | 5.64E-05 | -0.27044 | 0.78682 | 0.99961 |
| 16 | 84445883 | 85148685 | 788 | -2.83E-05 | 3.74E-09 | 6.12E-05 | -0.46347 | 0.64303 | 0.99961 |
| 16 | 85148685 | 86156599 | 814 | 1.75E-05 | 4.06E-09 | 6.37E-05 | 0.2746 | 0.78363 | 0.99961 |
| 16 | 86156599 | 86815411 | 860 | -8.68E-05 | 4.00E-09 | 6.32E-05 | -1.3728 | 0.1698 | 0.99961 |
| 16 | 86815411 | 87647349 | 615 | -1.86E-05 | 3.79E-09 | 6.16E-05 | -0.30284 | 0.76201 | 0.99961 |
| 16 | 87647349 | 89041165 | 597 | 1.69E-05 | 3.83E-09 | 6.19E-05 | 0.27312 | 0.78476 | 0.99961 |
| 16 | 89041165 | 90292812 | 516 | 3.35E-05 | 3.04E-09 | 5.51E-05 | 0.60838 | 0.54294 | 0.99961 |
| 17 | 56 | 1172399 | 463 | 4.55E-06 | 2.55E-09 | 5.05E-05 | 0.090003 | 0.92828 | 0.99961 |
| 17 | 1172399 | 1928731 | 391 | 1.40E-06 | 2.72E-09 | 5.22E-05 | 0.026765 | 0.97865 | 0.99961 |
| 17 | 1928731 | 3702312 | 1061 | 7.37E-06 | 3.37E-09 | 5.80E-05 | 0.12689 | 0.89902 | 0.99961 |
| 17 | 3702312 | 4696261 | 561 | 3.63E-05 | 2.89E-09 | 5.37E-05 | 0.67622 | 0.4989 | 0.99961 |
| 17 | 4696261 | 5741334 | 734 | 2.04E-05 | 3.02E-09 | 5.49E-05 | 0.37082 | 0.71077 | 0.99961 |
| 17 | 5741334 | 7317398 | 1046 | 2.32E-07 | 3.28E-09 | 5.73E-05 | 0.004044 | 0.99677 | 0.99961 |
| 17 | 7317398 | 8306425 | 430 | 4.43E-06 | 3.87E-09 | 6.22E-05 | 0.071228 | 0.94322 | 0.99961 |
| 17 | 8306425 | 9965921 | 1170 | 1.11E-05 | 3.19E-09 | 5.65E-05 | 0.19698 | 0.84385 | 0.99961 |
| 17 | 9965921 | 11102815 | 855 | 4.94E-07 | 3.62E-09 | 6.02E-05 | 0.008205 | 0.99345 | 0.99961 |
| 17 | 11102815 | 12534769 | 974 | 3.29E-05 | 3.54E-09 | 5.95E-05 | 0.55311 | 0.58019 | 0.99961 |
| 17 | 12534769 | 13180372 | 578 | -9.19E-06 | 2.74E-09 | 5.23E-05 | -0.17568 | 0.86055 | 0.99961 |
| 17 | 13180372 | 13894637 | 690 | 1.04E-05 | 3.05E-09 | 5.53E-05 | 0.18735 | 0.85139 | 0.99961 |
| 17 | 13894637 | 15019097 | 1232 | -9.09E-06 | 3.28E-09 | 5.72E-05 | -0.15871 | 0.87389 | 0.99961 |
| 17 | 15019097 | 16412342 | 579 | 5.51E-05 | 2.79E-09 | 5.28E-05 | 1.0431 | 0.29689 | 0.99961 |
| 17 | 16412342 | 18856320 | 950 | 3.79E-05 | 5.54E-09 | 7.44E-05 | 0.50896 | 0.61078 | 0.99961 |
| 17 | 18856320 | 21290357 | 675 | 1.88E-05 | 3.06E-09 | 5.53E-05 | 0.3405 | 0.73348 | 0.99961 |
| 17 | 21290357 | 27334244 | 978 | 5.34E-05 | 3.28E-09 | 5.73E-05 | 0.93115 | 0.35178 | 0.99961 |
| 17 | 27334244 | 29786491 | 737 | 7.60E-06 | 3.55E-09 | 5.96E-05 | 0.12767 | 0.89841 | 0.99961 |
| 17 | 29786491 | 31538425 | 944 | -1.53E-06 | 3.57E-09 | 5.98E-05 | -0.02556 | 0.97961 | 0.99961 |
| 17 | 31538425 | 32912798 | 1554 | 3.05E-06 | 3.20E-09 | 5.65E-05 | 0.053917 | 0.957 | 0.99961 |
| 17 | 32912798 | 34469036 | 879 | 1.26E-05 | 3.41E-09 | 5.84E-05 | 0.21616 | 0.82887 | 0.99961 |
| 17 | 34469036 | 36809344 | 779 | -7.66E-06 | 3.34E-09 | 5.78E-05 | -0.13244 | 0.89463 | 0.99961 |
| 17 | 36809344 | 38877404 | 688 | -1.81E-05 | 3.38E-09 | 5.82E-05 | -0.31102 | 0.75578 | 0.99961 |
| 17 | 38877404 | 39899810 | 605 | -1.59E-05 | 2.12E-09 | 4.60E-05 | -0.34655 | 0.72893 | 0.99961 |
| 17 | 39899810 | 41772087 | 526 | 4.24E-05 | 3.84E-09 | 6.20E-05 | 0.68362 | 0.49422 | 0.99961 |
| 17 | 41772087 | 43056905 | 534 | 1.66E-05 | 2.44E-09 | 4.94E-05 | 0.33574 | 0.73707 | 0.99961 |
| 17 | 43056905 | 45876022 | 1169 | 8.23E-05 | 4.48E-09 | 6.69E-05 | 1.2299 | 0.21872 | 0.99961 |
| 17 | 45876022 | 47517400 | 871 | 7.43E-05 | 3.82E-09 | 6.18E-05 | 1.2031 | 0.22895 | 0.99961 |
| 17 | 47517400 | 49898957 | 1275 | -1.20E-05 | 3.55E-09 | 5.96E-05 | -0.20128 | 0.84048 | 0.99961 |
| 17 | 49898957 | 50719055 | 585 | 6.26E-06 | 1.73E-09 | 4.16E-05 | 0.1506 | 0.88029 | 0.99961 |
| 17 | 50719055 | 51826118 | 814 | -2.89E-05 | 2.12E-09 | 4.60E-05 | -0.62698 | 0.53067 | 0.99961 |
| 17 | 51826118 | 53599432 | 1213 | -2.32E-05 | 3.25E-09 | 5.70E-05 | -0.40657 | 0.68432 | 0.99961 |
| 17 | 53599432 | 55357541 | 1123 | 1.23E-06 | 3.63E-09 | 6.02E-05 | 0.02036 | 0.98376 | 0.99961 |
| 17 | 55357541 | 57487512 | 1100 | -3.98E-06 | 3.49E-09 | 5.91E-05 | -0.06743 | 0.94624 | 0.99961 |
| 17 | 57487512 | 59312755 | 406 | 4.03E-05 | 1.66E-09 | 4.08E-05 | 0.98948 | 0.32243 | 0.99961 |
| 17 | 59312755 | 61545589 | 788 | -5.69E-05 | 3.93E-09 | 6.27E-05 | -0.90659 | 0.36462 | 0.99961 |
| 17 | 61545589 | 63148128 | 501 | 0.00010534 | 2.73E-09 | 5.23E-05 | 2.0144 | 0.043965 | 0.99961 |
| 17 | 63148128 | 64800430 | 1299 | -6.28E-05 | 3.59E-09 | 5.99E-05 | -1.0491 | 0.29415 | 0.99961 |
| 17 | 64800430 | 67858770 | 1682 | 0.00011923 | 4.27E-09 | 6.54E-05 | 1.8241 | 0.068135 | 0.99961 |
| 17 | 67858770 | 69387817 | 1340 | -4.91E-05 | 6.52E-09 | 8.07E-05 | -0.60848 | 0.54287 | 0.99961 |
| 17 | 69387817 | 72672203 | 2464 | -4.73E-05 | 3.52E-09 | 5.93E-05 | -0.79709 | 0.4254 | 0.99961 |
| 17 | 72672203 | 74375560 | 652 | 7.71E-05 | 4.51E-09 | 6.71E-05 | 1.1491 | 0.25051 | 0.99961 |
| 17 | 74375560 | 76263413 | 1202 | 4.56E-05 | 3.40E-09 | 5.83E-05 | 0.78176 | 0.43435 | 0.99961 |
| 17 | 76263413 | 77298636 | 724 | 2.54E-05 | 4.04E-09 | 6.36E-05 | 0.3995 | 0.68953 | 0.99961 |
| 17 | 77298636 | 78837588 | 955 | 7.72E-06 | 3.28E-09 | 5.73E-05 | 0.13467 | 0.89287 | 0.99961 |
| 17 | 78837588 | 80034408 | 432 | -2.63E-05 | 2.74E-09 | 5.23E-05 | -0.50261 | 0.61524 | 0.99961 |
| 17 | 80034408 | 81194908 | 518 | -4.73E-05 | 2.18E-09 | 4.67E-05 | -1.0118 | 0.31164 | 0.99961 |
| 18 | 10644 | 912486 | 662 | -3.98E-06 | 3.29E-09 | 5.73E-05 | -0.06933 | 0.94473 | 0.99961 |
| 18 | 912486 | 1942911 | 796 | -9.45E-06 | 2.77E-09 | 5.26E-05 | -0.17961 | 0.85746 | 0.99961 |
| 18 | 1942911 | 3891658 | 1447 | 7.62E-06 | 3.25E-09 | 5.70E-05 | 0.13352 | 0.89378 | 0.99961 |
| 18 | 3891658 | 5834180 | 1508 | -2.23E-05 | 2.91E-09 | 5.39E-05 | -0.41382 | 0.67901 | 0.99961 |
| 18 | 5834180 | 7090485 | 1030 | 3.98E-06 | 3.36E-09 | 5.79E-05 | 0.068602 | 0.94531 | 0.99961 |
| 18 | 7090485 | 8498931 | 1047 | 2.70E-05 | 3.22E-09 | 5.67E-05 | 0.47656 | 0.63367 | 0.99961 |
| 18 | 8498931 | 11075913 | 2238 | -7.85E-06 | 3.17E-09 | 5.63E-05 | -0.1394 | 0.88913 | 0.99961 |
| 18 | 11075913 | 11905440 | 516 | 1.72E-05 | 2.37E-09 | 4.87E-05 | 0.35367 | 0.72359 | 0.99961 |
| 18 | 11905440 | 14440799 | 1309 | -8.16E-06 | 3.53E-09 | 5.94E-05 | -0.13748 | 0.89065 | 0.99961 |
| 18 | 14440799 | 19485844 | 319 | 2.56E-05 | 1.40E-09 | 3.75E-05 | 0.68199 | 0.49524 | 0.99961 |
| 18 | 19485844 | 20649472 | 640 | -7.89E-06 | 2.27E-09 | 4.77E-05 | -0.16562 | 0.86845 | 0.99961 |
| 18 | 20649472 | 22996651 | 1469 | -1.23E-05 | 3.84E-09 | 6.20E-05 | -0.19904 | 0.84223 | 0.99961 |
| 18 | 22996651 | 24026191 | 627 | -4.56E-05 | 2.59E-09 | 5.09E-05 | -0.8962 | 0.37015 | 0.99961 |
| 18 | 24026191 | 25927682 | 1475 | 1.81E-05 | 3.41E-09 | 5.84E-05 | 0.3092 | 0.75717 | 0.99961 |
| 18 | 25927682 | 26875587 | 627 | 1.42E-05 | 1.96E-09 | 4.42E-05 | 0.32077 | 0.74838 | 0.99961 |
| 18 | 26875587 | 27866478 | 690 | -2.21E-06 | 1.65E-09 | 4.06E-05 | -0.05441 | 0.95661 | 0.99961 |
| 18 | 27866478 | 30264066 | 1794 | -1.36E-05 | 3.24E-09 | 5.69E-05 | -0.2389 | 0.81118 | 0.99961 |
| 18 | 30264066 | 31780067 | 845 | 6.18E-06 | 2.37E-09 | 4.87E-05 | 0.12708 | 0.89887 | 0.99961 |
| 18 | 31780067 | 33861964 | 1310 | 3.44E-05 | 3.19E-09 | 5.65E-05 | 0.60873 | 0.54271 | 0.99961 |
| 18 | 33861964 | 35075250 | 890 | -7.98E-07 | 3.09E-09 | 5.56E-05 | -0.01435 | 0.98855 | 0.99961 |
| 18 | 35075250 | 36365490 | 1059 | 3.07E-05 | 2.90E-09 | 5.39E-05 | 0.56905 | 0.56932 | 0.99961 |
| 18 | 36365490 | 37684367 | 812 | 3.76E-05 | 2.02E-09 | 4.50E-05 | 0.83657 | 0.40284 | 0.99961 |
| 18 | 37684367 | 39892648 | 1772 | -1.92E-05 | 3.31E-09 | 5.75E-05 | -0.33427 | 0.73818 | 0.99961 |
| 18 | 39892648 | 42922106 | 2093 | 3.33E-05 | 4.11E-09 | 6.41E-05 | 0.51925 | 0.60359 | 0.99961 |
| 18 | 42922106 | 44299246 | 1184 | 2.11E-05 | 3.23E-09 | 5.69E-05 | 0.371 | 0.71064 | 0.99961 |
| 18 | 44299246 | 45939732 | 1077 | -1.44E-05 | 3.84E-09 | 6.19E-05 | -0.23256 | 0.8161 | 0.99961 |
| 18 | 45939732 | 47730584 | 1312 | -1.71E-05 | 4.28E-09 | 6.54E-05 | -0.26171 | 0.79354 | 0.99961 |
| 18 | 47730584 | 51062185 | 2450 | 3.19E-05 | 3.67E-09 | 6.06E-05 | 0.52568 | 0.59911 | 0.99961 |
| 18 | 51062185 | 51554175 | 459 | 5.66E-06 | 1.20E-09 | 3.46E-05 | 0.16365 | 0.87 | 0.99961 |
| 18 | 51554175 | 55213838 | 2155 | -2.41E-05 | 3.23E-09 | 5.69E-05 | -0.4244 | 0.67128 | 0.99961 |
| 18 | 55213838 | 56530220 | 1066 | 2.92E-05 | 3.03E-09 | 5.51E-05 | 0.5301 | 0.59604 | 0.99961 |
| 18 | 56530220 | 57630483 | 1027 | 1.90E-05 | 3.07E-09 | 5.54E-05 | 0.3423 | 0.73213 | 0.99961 |
| 18 | 57630483 | 59020751 | 993 | -5.52E-05 | 3.29E-09 | 5.73E-05 | -0.96379 | 0.33515 | 0.99961 |
| 18 | 59020751 | 60277715 | 1170 | 4.12E-05 | 3.61E-09 | 6.01E-05 | 0.68537 | 0.49311 | 0.99961 |
| 18 | 60277715 | 61820252 | 1178 | 0.00013808 | 4.28E-09 | 6.54E-05 | 2.1113 | 0.03475 | 0.99961 |
| 18 | 61820252 | 63463402 | 1262 | 4.60E-05 | 3.22E-09 | 5.67E-05 | 0.81145 | 0.41711 | 0.99961 |
| 18 | 63463402 | 64252801 | 708 | -5.31E-06 | 1.87E-09 | 4.33E-05 | -0.12267 | 0.90237 | 0.99961 |
| 18 | 64252801 | 66125817 | 1674 | -5.11E-05 | 3.20E-09 | 5.66E-05 | -0.9019 | 0.36711 | 0.99961 |
| 18 | 66125817 | 67175820 | 725 | -3.23E-05 | 2.56E-09 | 5.06E-05 | -0.639 | 0.52282 | 0.99961 |
| 18 | 67175820 | 68621179 | 920 | 3.61E-06 | 2.95E-09 | 5.43E-05 | 0.066382 | 0.94707 | 0.99961 |
| 18 | 68621179 | 69621618 | 926 | 2.10E-05 | 3.07E-09 | 5.54E-05 | 0.37852 | 0.70504 | 0.99961 |
| 18 | 69621618 | 71038474 | 1244 | -3.77E-05 | 3.13E-09 | 5.59E-05 | -0.6734 | 0.50069 | 0.99961 |
| 18 | 71038474 | 71971462 | 987 | 1.94E-05 | 3.03E-09 | 5.51E-05 | 0.35196 | 0.72487 | 0.99961 |
| 18 | 71971462 | 73526987 | 1310 | -4.12E-05 | 3.59E-09 | 5.99E-05 | -0.68732 | 0.49188 | 0.99961 |
| 18 | 73526987 | 74813815 | 1105 | -3.58E-05 | 4.09E-09 | 6.40E-05 | -0.55929 | 0.57596 | 0.99961 |
| 18 | 74813815 | 75976317 | 1173 | -4.45E-05 | 3.50E-09 | 5.92E-05 | -0.75182 | 0.45216 | 0.99961 |
| 18 | 75976317 | 77149991 | 926 | -3.97E-07 | 3.18E-09 | 5.64E-05 | -0.00705 | 0.99438 | 0.99961 |
| 18 | 77149991 | 78017158 | 500 | 2.22E-05 | 2.61E-09 | 5.11E-05 | 0.43442 | 0.66398 | 0.99961 |
| 19 | 80840 | 610729 | 123 | -5.12E-05 | 1.04E-09 | 3.23E-05 | -1.5861 | 0.11272 | 0.99961 |
| 19 | 610729 | 2098396 | 472 | 6.37E-06 | 3.04E-09 | 5.51E-05 | 0.11566 | 0.90792 | 0.99961 |
| 19 | 2098396 | 3019660 | 387 | -5.27E-05 | 2.95E-09 | 5.43E-05 | -0.97134 | 0.33138 | 0.99961 |
| 19 | 3019660 | 4348967 | 500 | 3.12E-05 | 3.46E-09 | 5.88E-05 | 0.53027 | 0.59592 | 0.99961 |
| 19 | 4348967 | 5811852 | 621 | 3.34E-05 | 3.94E-09 | 6.27E-05 | 0.53309 | 0.59397 | 0.99961 |
| 19 | 5811852 | 6684885 | 330 | 3.22E-05 | 2.10E-09 | 4.58E-05 | 0.70185 | 0.48278 | 0.99961 |
| 19 | 6684885 | 8347513 | 859 | 2.13E-05 | 4.47E-09 | 6.69E-05 | 0.31853 | 0.75008 | 0.99961 |
| 19 | 8347513 | 9238393 | 406 | 3.86E-05 | 2.43E-09 | 4.93E-05 | 0.78303 | 0.43361 | 0.99961 |
| 19 | 9238393 | 11284028 | 759 | -1.98E-05 | 4.27E-09 | 6.53E-05 | -0.30318 | 0.76175 | 0.99961 |
| 19 | 11284028 | 13471127 | 698 | 1.16E-05 | 3.63E-09 | 6.03E-05 | 0.19193 | 0.8478 | 0.99961 |
| 19 | 13471127 | 14486347 | 350 | -2.34E-05 | 2.32E-09 | 4.82E-05 | -0.48473 | 0.62786 | 0.99961 |
| 19 | 14486347 | 15648610 | 554 | -1.30E-06 | 2.76E-09 | 5.25E-05 | -0.02477 | 0.98024 | 0.99961 |
| 19 | 15648610 | 16374416 | 634 | -1.94E-05 | 2.42E-09 | 4.92E-05 | -0.39383 | 0.69371 | 0.99961 |
| 19 | 16374416 | 18409862 | 845 | 0.00010311 | 5.06E-09 | 7.11E-05 | 1.4492 | 0.14727 | 0.99961 |
| 19 | 18409862 | 19877471 | 505 | 9.97E-05 | 3.59E-09 | 5.99E-05 | 1.6635 | 0.096203 | 0.99961 |
| 19 | 19877471 | 20905757 | 310 | 9.06E-07 | 1.27E-09 | 3.57E-05 | 0.025413 | 0.97973 | 0.99961 |
| 19 | 20905757 | 22732896 | 830 | 9.55E-06 | 2.33E-09 | 4.82E-05 | 0.19787 | 0.84315 | 0.99961 |
| 19 | 22732896 | 23467746 | 310 | -3.05E-05 | 1.14E-09 | 3.38E-05 | -0.9025 | 0.36679 | 0.99961 |
| 19 | 23467746 | 28557893 | 734 | -4.78E-06 | 2.00E-09 | 4.47E-05 | -0.10708 | 0.91473 | 0.99961 |
| 19 | 28557893 | 29790947 | 1159 | 2.74E-05 | 3.22E-09 | 5.67E-05 | 0.4836 | 0.62867 | 0.99961 |
| 19 | 29790947 | 30727954 | 679 | -8.22E-05 | 2.93E-09 | 5.42E-05 | -1.5183 | 0.12894 | 0.99961 |
| 19 | 30727954 | 32746520 | 1197 | 3.58E-06 | 3.57E-09 | 5.98E-05 | 0.059876 | 0.95225 | 0.99961 |
| 19 | 32746520 | 34262952 | 915 | -9.24E-06 | 5.32E-09 | 7.29E-05 | -0.12668 | 0.89919 | 0.99961 |
| 19 | 34262952 | 36469295 | 1079 | -9.17E-06 | 3.12E-09 | 5.59E-05 | -0.1641 | 0.86966 | 0.99961 |
| 19 | 36469295 | 37527033 | 445 | -9.20E-06 | 1.81E-09 | 4.25E-05 | -0.21641 | 0.82867 | 0.99961 |
| 19 | 37527033 | 40170619 | 1049 | 1.57E-05 | 4.00E-09 | 6.32E-05 | 0.24887 | 0.80346 | 0.99961 |
| 19 | 40170619 | 40984601 | 309 | -3.09E-05 | 1.74E-09 | 4.17E-05 | -0.74155 | 0.45836 | 0.99961 |
| 19 | 40984601 | 42131573 | 464 | 4.48E-05 | 3.14E-09 | 5.61E-05 | 0.79964 | 0.42392 | 0.99961 |
| 19 | 42131573 | 43862455 | 431 | -4.00E-06 | 1.82E-09 | 4.27E-05 | -0.09372 | 0.92533 | 0.99961 |
| 19 | 43862455 | 44744108 | 508 | 2.44E-05 | 2.39E-09 | 4.88E-05 | 0.5006 | 0.61665 | 0.99961 |
| 19 | 44744108 | 46102697 | 616 | -4.34E-05 | 4.01E-09 | 6.33E-05 | -0.68509 | 0.49329 | 0.99961 |
| 19 | 46102697 | 47150082 | 460 | 6.71E-05 | 2.65E-09 | 5.15E-05 | 1.3042 | 0.19216 | 0.99961 |
| 19 | 47150082 | 49282227 | 919 | -1.45E-05 | 3.43E-09 | 5.86E-05 | -0.24794 | 0.80418 | 0.99961 |
| 19 | 49282227 | 51532567 | 973 | 3.45E-05 | 3.45E-09 | 5.87E-05 | 0.5879 | 0.5566 | 0.99961 |
| 19 | 51532567 | 52985290 | 1137 | 9.23E-06 | 3.22E-09 | 5.67E-05 | 0.16267 | 0.87078 | 0.99961 |
| 19 | 52985290 | 54602362 | 878 | 3.08E-05 | 3.40E-09 | 5.83E-05 | 0.52759 | 0.59779 | 0.99961 |
| 19 | 54602362 | 55728080 | 544 | 1.89E-05 | 2.86E-09 | 5.35E-05 | 0.35384 | 0.72346 | 0.99961 |
| 19 | 55728080 | 57521335 | 1078 | 2.06E-05 | 3.47E-09 | 5.89E-05 | 0.34952 | 0.7267 | 0.99961 |
| 19 | 57521335 | 59118839 | 828 | 3.47E-05 | 3.45E-09 | 5.88E-05 | 0.59109 | 0.55446 | 0.99961 |
| 20 | 60479 | 697600 | 409 | -7.10E-06 | 2.38E-09 | 4.88E-05 | -0.14558 | 0.88426 | 0.99961 |
| 20 | 697600 | 2469501 | 1750 | 1.00E-05 | 3.41E-09 | 5.84E-05 | 0.17171 | 0.86366 | 0.99961 |
| 20 | 2469501 | 3828413 | 877 | 4.47E-05 | 3.23E-09 | 5.68E-05 | 0.78657 | 0.43153 | 0.99961 |
| 20 | 3828413 | 5477850 | 1395 | -1.93E-05 | 3.08E-09 | 5.55E-05 | -0.34845 | 0.7275 | 0.99961 |
| 20 | 5477850 | 7084073 | 1485 | -4.09E-05 | 4.26E-09 | 6.52E-05 | -0.62742 | 0.53039 | 0.99961 |
| 20 | 7084073 | 8117011 | 1015 | 6.66E-06 | 3.29E-09 | 5.73E-05 | 0.11611 | 0.90756 | 0.99961 |
| 20 | 8117011 | 9730921 | 1521 | 3.10E-05 | 3.22E-09 | 5.68E-05 | 0.54633 | 0.58484 | 0.99961 |
| 20 | 9730921 | 11249536 | 1456 | -4.03E-06 | 4.03E-09 | 6.35E-05 | -0.06343 | 0.94942 | 0.99961 |
| 20 | 11249536 | 12447499 | 1058 | 1.01E-05 | 3.32E-09 | 5.76E-05 | 0.17556 | 0.86064 | 0.99961 |
| 20 | 12447499 | 13689864 | 1117 | -1.26E-05 | 3.14E-09 | 5.61E-05 | -0.22423 | 0.82258 | 0.99961 |
| 20 | 13689864 | 15958359 | 1999 | 6.24E-05 | 3.24E-09 | 5.69E-05 | 1.0954 | 0.27336 | 0.99961 |
| 20 | 15958359 | 17487232 | 1797 | 1.06E-05 | 3.14E-09 | 5.60E-05 | 0.18836 | 0.85059 | 0.99961 |
| 20 | 17487232 | 18972203 | 1161 | 4.63E-06 | 3.85E-09 | 6.20E-05 | 0.074702 | 0.94045 | 0.99961 |
| 20 | 18972203 | 20959710 | 1744 | -4.70E-05 | 3.63E-09 | 6.02E-05 | -0.78064 | 0.43502 | 0.99961 |
| 20 | 20959710 | 22491456 | 865 | -2.76E-05 | 2.77E-09 | 5.26E-05 | -0.52566 | 0.59912 | 0.99961 |
| 20 | 22491456 | 23293790 | 815 | -3.16E-05 | 2.43E-09 | 4.93E-05 | -0.6411 | 0.52145 | 0.99961 |
| 20 | 23293790 | 24717724 | 1110 | -2.23E-05 | 3.10E-09 | 5.57E-05 | -0.39972 | 0.68936 | 0.99961 |
| 20 | 24717724 | 25344231 | 574 | 1.32E-05 | 1.47E-09 | 3.83E-05 | 0.34498 | 0.73011 | 0.99961 |
| 20 | 25344231 | 31614823 | 1091 | -3.17E-05 | 3.46E-09 | 5.89E-05 | -0.53827 | 0.59039 | 0.99961 |
| 20 | 31614823 | 32813441 | 455 | 1.39E-05 | 1.99E-09 | 4.46E-05 | 0.31242 | 0.75472 | 0.99961 |
| 20 | 32813441 | 34960446 | 762 | 4.75E-05 | 2.93E-09 | 5.42E-05 | 0.87628 | 0.38088 | 0.99961 |
| 20 | 34960446 | 36909530 | 887 | 6.45E-06 | 3.55E-09 | 5.96E-05 | 0.1083 | 0.91376 | 0.99961 |
| 20 | 36909530 | 38436761 | 1069 | 1.48E-05 | 3.30E-09 | 5.75E-05 | 0.25765 | 0.79667 | 0.99961 |
| 20 | 38436761 | 39610856 | 937 | -2.13E-06 | 4.60E-09 | 6.78E-05 | -0.03137 | 0.97498 | 0.99961 |
| 20 | 39610856 | 40585689 | 557 | 2.07E-05 | 2.55E-09 | 5.05E-05 | 0.41052 | 0.68142 | 0.99961 |
| 20 | 40585689 | 41615460 | 1034 | 3.60E-05 | 2.96E-09 | 5.44E-05 | 0.66265 | 0.50755 | 0.99961 |
| 20 | 41615460 | 42680176 | 688 | 3.22E-06 | 3.17E-09 | 5.63E-05 | 0.057168 | 0.95441 | 0.99961 |
| 20 | 42680176 | 44839056 | 1377 | -4.73E-05 | 3.14E-09 | 5.61E-05 | -0.84349 | 0.39895 | 0.99961 |
| 20 | 44839056 | 47199980 | 1886 | -1.54E-05 | 5.91E-09 | 7.69E-05 | -0.2 | 0.84148 | 0.99961 |
| 20 | 47199980 | 49239658 | 1408 | 4.71E-05 | 3.56E-09 | 5.97E-05 | 0.78884 | 0.4302 | 0.99961 |
| 20 | 49239658 | 52472549 | 2517 | 6.31E-05 | 4.59E-09 | 6.78E-05 | 0.93154 | 0.35157 | 0.99961 |
| 20 | 52472549 | 54055266 | 1278 | 2.91E-05 | 3.27E-09 | 5.71E-05 | 0.50905 | 0.61072 | 0.99961 |
| 20 | 54055266 | 56447959 | 2138 | 1.38E-05 | 3.47E-09 | 5.89E-05 | 0.23505 | 0.81417 | 0.99961 |
| 20 | 56447959 | 58406572 | 1501 | 2.12E-05 | 3.74E-09 | 6.11E-05 | 0.34633 | 0.7291 | 0.99961 |
| 20 | 58406572 | 59561724 | 1034 | -3.37E-06 | 3.00E-09 | 5.48E-05 | -0.06157 | 0.9509 | 0.99961 |
| 20 | 59561724 | 61301855 | 1357 | -1.21E-05 | 3.28E-09 | 5.73E-05 | -0.21121 | 0.83272 | 0.99961 |
| 20 | 61301855 | 62190180 | 523 | 2.40E-05 | 3.06E-09 | 5.53E-05 | 0.43346 | 0.66468 | 0.99961 |
| 20 | 62190180 | 62965163 | 379 | -5.04E-06 | 3.23E-09 | 5.68E-05 | -0.08867 | 0.92935 | 0.99961 |
| 21 | 9411243 | 15950982 | 557 | -4.77E-05 | 2.62E-09 | 5.12E-05 | -0.93117 | 0.35177 | 0.99961 |
| 21 | 15950982 | 18053165 | 1316 | -2.82E-05 | 3.45E-09 | 5.88E-05 | -0.48057 | 0.63083 | 0.99961 |
| 21 | 18053165 | 19480375 | 887 | 1.75E-05 | 3.98E-09 | 6.31E-05 | 0.2781 | 0.78094 | 0.99961 |
| 21 | 19480375 | 20962339 | 1273 | -6.76E-06 | 3.27E-09 | 5.71E-05 | -0.11832 | 0.90582 | 0.99961 |
| 21 | 20962339 | 22072764 | 856 | 1.18E-06 | 3.25E-09 | 5.70E-05 | 0.020654 | 0.98352 | 0.99961 |
| 21 | 22072764 | 23362875 | 1153 | -3.89E-05 | 3.38E-09 | 5.81E-05 | -0.67021 | 0.50272 | 0.99961 |
| 21 | 23362875 | 24696231 | 829 | 2.83E-05 | 2.97E-09 | 5.45E-05 | 0.52035 | 0.60282 | 0.99961 |
| 21 | 24696231 | 26469122 | 1455 | -2.77E-05 | 3.11E-09 | 5.58E-05 | -0.49591 | 0.61996 | 0.99961 |
| 21 | 26469122 | 27271019 | 454 | 8.91E-06 | 1.62E-09 | 4.03E-05 | 0.22108 | 0.82503 | 0.99961 |
| 21 | 27271019 | 29125226 | 1679 | 2.34E-05 | 3.37E-09 | 5.81E-05 | 0.40284 | 0.68707 | 0.99961 |
| 21 | 29125226 | 31197025 | 1466 | 1.36E-05 | 3.67E-09 | 6.06E-05 | 0.22463 | 0.82227 | 0.99961 |
| 21 | 31197025 | 32668642 | 1060 | 1.74E-07 | 2.38E-09 | 4.88E-05 | 0.003562 | 0.99716 | 0.99961 |
| 21 | 32668642 | 34376999 | 1074 | -4.21E-05 | 3.69E-09 | 6.07E-05 | -0.69292 | 0.48836 | 0.99961 |
| 21 | 34376999 | 35225465 | 475 | -5.55E-06 | 2.42E-09 | 4.92E-05 | -0.11279 | 0.9102 | 0.99961 |
| 21 | 35225465 | 36524373 | 948 | -5.39E-05 | 4.31E-09 | 6.57E-05 | -0.82104 | 0.41163 | 0.99961 |
| 21 | 36524373 | 37870779 | 940 | 6.25E-06 | 3.71E-09 | 6.09E-05 | 0.10255 | 0.91832 | 0.99961 |
| 21 | 37870779 | 38711704 | 592 | 4.18E-05 | 2.73E-09 | 5.22E-05 | 0.80016 | 0.42362 | 0.99961 |
| 21 | 38711704 | 40482902 | 1529 | 9.28E-05 | 4.45E-09 | 6.67E-05 | 1.3907 | 0.16432 | 0.99961 |
| 21 | 40482902 | 41389527 | 887 | 1.37E-05 | 3.09E-09 | 5.55E-05 | 0.24713 | 0.80481 | 0.99961 |
| 21 | 41389527 | 43321528 | 1693 | 4.20E-08 | 3.13E-09 | 5.59E-05 | 0.000751 | 0.9994 | 0.99961 |
| 21 | 43321528 | 44506268 | 1113 | -2.93E-05 | 3.11E-09 | 5.58E-05 | -0.52497 | 0.59961 | 0.99961 |
| 21 | 44506268 | 46177105 | 698 | 5.01E-05 | 3.24E-09 | 5.70E-05 | 0.87983 | 0.37895 | 0.99961 |
| 21 | 46177105 | 47492226 | 884 | -7.62E-05 | 4.20E-09 | 6.48E-05 | -1.176 | 0.23958 | 0.99961 |
| 21 | 47492226 | 48119752 | 405 | 9.09E-05 | 2.62E-09 | 5.12E-05 | 1.7754 | 0.075832 | 0.99961 |
| 22 | 16050408 | 17674295 | 369 | 1.86E-05 | 2.00E-09 | 4.47E-05 | 0.41677 | 0.67685 | 0.99961 |
| 22 | 17674295 | 18296088 | 407 | 4.86E-05 | 1.88E-09 | 4.34E-05 | 1.1193 | 0.26303 | 0.99961 |
| 22 | 18296088 | 19912358 | 896 | 1.90E-05 | 3.31E-09 | 5.75E-05 | 0.33074 | 0.74084 | 0.99961 |
| 22 | 19912358 | 22357325 | 972 | 5.82E-05 | 3.40E-09 | 5.83E-05 | 0.99804 | 0.31826 | 0.99961 |
| 22 | 22357325 | 23712647 | 715 | -2.45E-05 | 2.83E-09 | 5.32E-05 | -0.46087 | 0.6449 | 0.99961 |
| 22 | 23712647 | 24984204 | 618 | 5.51E-05 | 2.41E-09 | 4.91E-05 | 1.1227 | 0.26157 | 0.99961 |
| 22 | 24984204 | 26791628 | 1298 | -1.54E-05 | 3.23E-09 | 5.68E-05 | -0.27068 | 0.78663 | 0.99961 |
| 22 | 26791628 | 27834752 | 1302 | -3.93E-05 | 3.43E-09 | 5.86E-05 | -0.6717 | 0.50177 | 0.99961 |
| 22 | 27834752 | 29651799 | 988 | 2.77E-05 | 5.19E-09 | 7.20E-05 | 0.38417 | 0.70085 | 0.99961 |
| 22 | 29651799 | 31439918 | 949 | -3.42E-06 | 3.82E-09 | 6.18E-05 | -0.05531 | 0.95589 | 0.99961 |
| 22 | 31439918 | 32664986 | 576 | 5.75E-06 | 2.92E-09 | 5.41E-05 | 0.10643 | 0.91524 | 0.99961 |
| 22 | 32664986 | 34494914 | 1714 | 3.60E-05 | 3.12E-09 | 5.59E-05 | 0.64479 | 0.51907 | 0.99961 |
| 22 | 34494914 | 35530985 | 1112 | -1.58E-05 | 3.18E-09 | 5.64E-05 | -0.28046 | 0.77912 | 0.99961 |
| 22 | 35530985 | 37570269 | 1555 | 5.89E-05 | 3.83E-09 | 6.19E-05 | 0.95125 | 0.34148 | 0.99961 |
| 22 | 37570269 | 39307894 | 881 | 0.00010009 | 4.13E-09 | 6.42E-05 | 1.5582 | 0.1192 | 0.99961 |
| 22 | 39307894 | 40545797 | 585 | 3.66E-05 | 3.14E-09 | 5.61E-05 | 0.65281 | 0.51388 | 0.99961 |
| 22 | 40545797 | 42690818 | 823 | 1.59E-05 | 3.45E-09 | 5.87E-05 | 0.2706 | 0.7867 | 0.99961 |
| 22 | 42690818 | 43714200 | 731 | -4.35E-05 | 3.85E-09 | 6.20E-05 | -0.70069 | 0.4835 | 0.99961 |
| 22 | 43714200 | 44995308 | 1237 | -2.83E-07 | 3.21E-09 | 5.67E-05 | -0.005 | 0.99601 | 0.99961 |
| 22 | 44995308 | 46470495 | 1120 | 3.28E-05 | 3.34E-09 | 5.78E-05 | 0.56792 | 0.57009 | 0.99961 |
| 22 | 46470495 | 47596318 | 866 | -4.50E-05 | 3.47E-09 | 5.89E-05 | -0.76401 | 0.44486 | 0.99961 |
| 22 | 47596318 | 48903703 | 1511 | 1.11E-05 | 2.96E-09 | 5.44E-05 | 0.20434 | 0.83808 | 0.99961 |
| 22 | 48903703 | 49824534 | 1282 | 5.20E-05 | 2.99E-09 | 5.47E-05 | 0.95013 | 0.34205 | 0.99961 |
| 22 | 49824534 | 51243298 | 709 | -1.69E-05 | 3.16E-09 | 5.62E-05 | -0.30143 | 0.76308 | 0.99961 |
