# Supplementary material for: Genetic Correlation and Causal Inference Between Female Fat Distribution and Preeclampsia: An Integrative Genomic Study
Source: FASEB J. 2026 Jun 23;40(12):e72074. doi: 10.1096/fj.202601888R (PMC13288445; doi:10.1096/fj.202601888R)
Supplement: Supplementary file 5 — Table S5: Significant Loci identified through MTAG and CPASSOC analysis. MTAG_P_PE: P from MTAG analysis for PE; MTAG_P_WHR: P from MTAG analysis for WHR; CPASSOC_P: P from CPASSOC analysis, indicating the combined association significance of the SNP with PE and WHR in a joint analysis. Other columns have been clarified in previous tables. [file FSB2-40-e72074-s002.docx]

| **Supplementary Table S5** | | |  |  |  |  |  |
| --- | --- | --- | --- | --- | --- | --- | --- |
| ***Significant Loci identified through MTAG and CPASSOC analysis.*** *MTAG_P_PE: P from MTAG analysis for PE; MTAG_P_WHR: P from MTAG analysis for WHR; CPASSOC_P: P from CPASSOC analysis, indicating the combined association significance of the SNP with PE and WHR in a joint analysis. Other columns have been clarified in previous tables.* | | | | | | | |
| **SNP** | **CHR** | **BP** | **A1** | **A2** | **MTAG_*P*_PE** | **MTAG_*P*_WHR** | **CPASSOC_*P*** |
| rs17037390 | 1 | 11860843 | A | G | 7.11E-09 | 2.75E-08 | 2.56E-14 |
| rs12567136 | 1 | 11883731 | T | C | 3.41E-09 | 8.95E-09 | 4.07E-15 |
