# Supplementary material for: Genetic Correlation and Causal Inference Between Female Fat Distribution and Preeclampsia: An Integrative Genomic Study
Source: FASEB J. 2026 Jun 23;40(12):e72074. doi: 10.1096/fj.202601888R (PMC13288445; doi:10.1096/fj.202601888R)
Supplement: Supplementary file 6 — Table S6: Significant Loci Identified through conjFDR Analysis for PE and WHR. BP: Base‐pair position of the variant on the chromosome (in reference to GRCh37/hg19 genome build); Pconj: conjunctional false discovery rate (conjFDR) P indicating the shared genetic association between PE and WHR; Leading SNP: It is the leading SNP (1 = yes, 0 = no); P_PE: P for PE derived after conjFDR analysis, reflecting the association significance of the SNP with PE in the shared genetic context of PE and WHR.; P_WHR: P for WHR derived after conjFDR analysis, reflecting the association significance of the SNP with WHR in the shared genetic context of PE and WHR; Nearest gene: The nearest gene to the identified variant, providing a potential functional annotation. Other columns have been clarified in previous tables. [file FSB2-40-e72074-s012.docx]

| **Supplementary Table S6** | | |  |  |  |  |  |  |  |
| --- | --- | --- | --- | --- | --- | --- | --- | --- | --- |
| ***Significant Loci Identified through conjFDR Analysis for PE and WHR.*** *BP: Base-pair position of the variant on the chromosome (in reference to GRCh37/hg19 genome build); Pconj: conjunctional false discovery rate (conjFDR) P indicating the shared genetic association between PE and WHR; Leading SNP: It is the leading SNP (1 = yes, 0= no); P_PE: P for PE derived after conjFDR analysis, reflecting the association significance of the SNP with PE in the shared genetic context of PE and WHR.; P_WHR: P for WHR derived after conjFDR analysis, reflecting the association significance of the SNP with WHR in the shared genetic context of PE and WHR; Nearest gene: The nearest gene to the identified variant, providing a potential functional annotation. Other columns have been clarified in previous tables.* | | | | | | | | | |
| **CHR** | **BP** | **Leading SNP** | **Z_PE** | **Z_WHR** | ***Pconj*** | ***P PE*** | ***P WHR*** | ***Nearest gene*** |  |
| 1 | 11860843 | 0 | 5.579503 | 4.882543 | 1.25E-04 | 2.41E-08 | 1.05E-06 | MTHFR |  |
| 1 | 11862778 | 1 | 5.705608 | -5.13756 | 3.80E-05 | 1.16E-08 | 2.78E-07 | MTHFR |  |
| 1 | 11865804 | 0 | 5.815012 | -4.81759 | 1.69E-04 | 6.06E-09 | 1.45E-06 | CLCN6 |  |
| 1 | 11879699 | 0 | 5.75884 | -4.77199 | 2.08E-04 | 8.47E-09 | 1.82E-06 | CLCN6 |  |
| 1 | 11883731 | 0 | 5.697301 | 5.052273 | 5.69E-05 | 1.22E-08 | 4.37E-07 | CLCN6 |  |
| 1 | 11887303 | 0 | 5.771412 | -4.81763 | 1.69E-04 | 7.86E-09 | 1.45E-06 | CLCN6 |  |
| 1 | 11895675 | 0 | 5.761047 | -3.97304 | 4.63E-03 | 8.36E-09 | 7.10E-05 | CLCN6 |  |
| 1 | 11904076 | 0 | 4.595765 | -4.32193 | 2.95E-03 | 4.31E-06 | 1.55E-05 | NPPA-AS1 |  |
| 6 | 31241032 | 0 | 4.357504 | -4.1018 | 8.50E-03 | 1.32E-05 | 4.10E-05 | HLA-C |  |
| 6 | 31253444 | 0 | 4.381418 | 3.845421 | 7.68E-03 | 1.18E-05 | 1.20E-04 |  |  |
| 6 | 31253866 | 0 | 4.340249 | 3.72991 | 9.60E-03 | 1.42E-05 | 1.92E-04 |  |  |
| 6 | 32409530 | 1 | -4.55265 | -5.6851 | 3.60E-03 | 5.30E-06 | 1.31E-08 | HLA-DRA |  |
