# Supplementary material for: Genetic Correlation and Causal Inference Between Female Fat Distribution and Preeclampsia: An Integrative Genomic Study
Source: FASEB J. 2026 Jun 23;40(12):e72074. doi: 10.1096/fj.202601888R (PMC13288445; doi:10.1096/fj.202601888R)
Supplement: Supplementary file 7 — Table S7: The SNP list of tissue‐level enrichment. Coefficient: regression coefficient; Other columns have been clarified in previous tables. [file FSB2-40-e72074-s008.docx]

| **Supplementary Table S7** | |  |  |  |  |
| --- | --- | --- | --- | --- | --- |
| ***The SNP list of tissue-level enrichment.*** *Coefficient: regression coefficient; Other columns have been clarified in previous tables.* | | | | | |
| **Traits** | **Name** | **Coefficient** | **SE** | ***P*** | ***Padj*** |
| WHR | Adipose_Subcutaneous | 2.20E-08 | 4.52E-09 | 5.36E-07 | 2.84E-05 |
| WHR | Breast_Mammary_Tissue | 2.18E-08 | 4.69E-09 | 1.73E-06 | 4.58E-05 |
| WHR | Adipose_Visceral_(Omentum) | 1.81E-08 | 5.06E-09 | 0.0001707 | 0.003016 |
| WHR | Cervix_Endocervix | 1.48E-08 | 4.71E-09 | 0.000825 | 0.010931 |
| WHR | Uterus | 9.75E-09 | 3.68E-09 | 0.0040641 | 0.04308 |
| WHR | Cervix_Ectocervix | 8.72E-09 | 3.74E-09 | 0.0098339 | 0.067907 |
| WHR | Fallopian_Tube | 1.03E-08 | 4.44E-09 | 0.0098825 | 0.067907 |
| WHR | Adrenal_Gland | 1.19E-08 | 5.13E-09 | 0.0102501 | 0.067907 |
| WHR | Skin_Sun_Exposed_(Lower_leg) | 1.05E-08 | 5.02E-09 | 0.0180864 | 0.106509 |
| WHR | Liver | 9.79E-09 | 4.81E-09 | 0.0208851 | 0.110691 |
| WHR | Nerve_Tibial | 7.99E-09 | 4.18E-09 | 0.02788 | 0.134331 |
| WHR | Skin_Not_Sun_Exposed_(Suprapubic) | 8.37E-09 | 4.94E-09 | 0.0450175 | 0.198827 |
| WHR | Bladder | 5.74E-09 | 3.92E-09 | 0.0713263 | 0.282753 |
| WHR | Minor_Salivary_Gland | 6.54E-09 | 4.55E-09 | 0.0751453 | 0.282753 |
| WHR | Vagina | 5.82E-09 | 4.14E-09 | 0.0800245 | 0.282753 |
| WHR | Thyroid | 5.88E-09 | 4.81E-09 | 0.1106526 | 0.366537 |
| WHR | Prostate | 4.70E-09 | 4.79E-09 | 0.1632091 | 0.508828 |
| WHR | Kidney_Cortex | 3.36E-09 | 4.70E-09 | 0.2372736 | 0.698639 |
| WHR | Artery_Coronary | 2.54E-09 | 4.01E-09 | 0.2630552 | 0.728014 |
| WHR | Testis | 2.00E-09 | 3.35E-09 | 0.2747223 | 0.728014 |
| WHR | Ovary | 1.44E-09 | 2.77E-09 | 0.3019148 | 0.734765 |
| WHR | Muscle_Skeletal | 2.29E-09 | 4.49E-09 | 0.3049969 | 0.734765 |
| WHR | Artery_Tibial | 1.16E-09 | 3.80E-09 | 0.380059 | 0.846369 |
| WHR | Stomach | 1.49E-09 | 5.28E-09 | 0.3891533 | 0.846369 |
| WHR | Heart_Left_Ventricle | 9.18E-10 | 3.60E-09 | 0.3992305 | 0.846369 |
| WHR | Cells_EBV-transformed_lymphocytes | 3.58E-10 | 4.16E-09 | 0.465753 | 0.94942 |
| WHR | Esophagus_Gastroesophageal_Junction | 7.01E-11 | 3.96E-09 | 0.4929449 | 0.967633 |
| WHR | Colon_Sigmoid | -7.61E-10 | 4.41E-09 | 0.5684781 | 0.999995 |
| WHR | Esophagus_Muscularis | -1.01E-09 | 4.00E-09 | 0.599804 | 0.999995 |
| WHR | Colon_Transverse | -1.25E-09 | 4.53E-09 | 0.6083812 | 0.999995 |
| WHR | Heart_Atrial_Appendage | -1.01E-09 | 3.47E-09 | 0.6138991 | 0.999995 |
| WHR | Pancreas | -1.41E-09 | 3.35E-09 | 0.6625079 | 0.999995 |
| WHR | Pituitary | -2.64E-09 | 3.41E-09 | 0.7810722 | 0.999995 |
| WHR | Whole_Blood | -3.11E-09 | 3.94E-09 | 0.785176 | 0.999995 |
| WHR | Cells_Transformed_fibroblasts | -3.26E-09 | 3.36E-09 | 0.8342567 | 0.999995 |
| WHR | Esophagus_Mucosa | -4.85E-09 | 4.68E-09 | 0.8502178 | 0.999995 |
| WHR | Artery_Aorta | -4.50E-09 | 3.65E-09 | 0.8911203 | 0.999995 |
| WHR | Small_Intestine_Terminal_Ileum | -5.46E-09 | 4.34E-09 | 0.8955365 | 0.999995 |
| WHR | Lung | -4.59E-09 | 3.49E-09 | 0.906196 | 0.999995 |
| WHR | Spleen | -8.41E-09 | 3.78E-09 | 0.9869882 | 0.999995 |
| WHR | Brain_Substantia_nigra | -8.04E-09 | 2.57E-09 | 0.9991056 | 0.999995 |
| WHR | Brain_Nucleus_accumbens_(basal_ganglia) | -8.28E-09 | 2.50E-09 | 0.9995494 | 0.999995 |
| WHR | Brain_Frontal_Cortex_(BA9) | -8.31E-09 | 2.49E-09 | 0.999577 | 0.999995 |
| WHR | Brain_Hypothalamus | -8.63E-09 | 2.42E-09 | 0.9998195 | 0.999995 |
| WHR | Brain_Putamen_(basal_ganglia) | -9.19E-09 | 2.58E-09 | 0.9998203 | 0.999995 |
| WHR | Brain_Caudate_(basal_ganglia) | -9.57E-09 | 2.58E-09 | 0.9998945 | 0.999995 |
| WHR | Brain_Anterior_cingulate_cortex_(BA24) | -9.31E-09 | 2.37E-09 | 0.999956 | 0.999995 |
| WHR | Brain_Spinal_cord_(cervical_c-1) | -9.97E-09 | 2.52E-09 | 0.9999623 | 0.999995 |
| WHR | Brain_Cerebellar_Hemisphere | -9.62E-09 | 2.29E-09 | 0.999987 | 0.999995 |
| WHR | Brain_Cortex | -9.40E-09 | 2.23E-09 | 0.999987 | 0.999995 |
| WHR | Brain_Amygdala | -1.02E-08 | 2.37E-09 | 0.999992 | 0.999995 |
| WHR | Brain_Cerebellum | -9.43E-09 | 2.16E-09 | 0.9999939 | 0.999995 |
| WHR | Brain_Hippocampus | -1.05E-08 | 2.36E-09 | 0.9999953 | 0.999995 |
| PE | Adrenal_Gland | 2.30E-09 | 1.19E-09 | 0.0004963 | 0.026301 |
| PE | Brain_Cerebellum | 1.93E-09 | 9.99E-10 | 0.0010084 | 0.026723 |
| PE | Cervix_Ectocervix | 2.06E-09 | 1.07E-09 | 0.0015534 | 0.027443 |
| PE | Brain_Cerebellar_Hemisphere | 1.84E-09 | 9.66E-10 | 0.0021617 | 0.028642 |
| PE | Adipose_Subcutaneous | 1.37E-09 | 8.54E-10 | 0.0050875 | 0.053927 |
| PE | Uterus | 1.73E-09 | 1.13E-09 | 0.006985 | 0.061701 |
| PE | Ovary | 1.47E-09 | 9.96E-10 | 0.0092678 | 0.070171 |
| PE | Fallopian_Tube | 1.35E-09 | 1.06E-09 | 0.0152216 | 0.100843 |
| PE | Adipose_Visceral_(Omentum) | 1.23E-09 | 9.67E-10 | 0.0171714 | 0.10112 |
| PE | Cells_EBV-transformed_lymphocytes | 1.40E-09 | 1.15E-09 | 0.0211885 | 0.112299 |
| PE | Colon_Transverse | 1.44E-09 | 1.25E-09 | 0.0257394 | 0.124017 |
| PE | Lung | 1.29E-09 | 1.14E-09 | 0.0290594 | 0.128346 |
| PE | Spleen | 1.58E-09 | 1.42E-09 | 0.0327806 | 0.133644 |
| PE | Breast_Mammary_Tissue | 8.94E-10 | 8.40E-10 | 0.0378906 | 0.143443 |
| PE | Vagina | 1.01E-09 | 1.05E-09 | 0.0474448 | 0.167638 |
| PE | Artery_Tibial | 7.47E-10 | 9.09E-10 | 0.062096 | 0.205693 |
| PE | Small_Intestine_Terminal_Ileum | 8.83E-10 | 1.21E-09 | 0.0747459 | 0.233031 |
| PE | Brain_Putamen_(basal_ganglia) | 6.48E-10 | 9.89E-10 | 0.08702 | 0.256226 |
| PE | Stomach | 7.16E-10 | 1.14E-09 | 0.0951162 | 0.265324 |
| PE | Cells_Transformed_fibroblasts | 5.97E-10 | 9.72E-10 | 0.1017054 | 0.269519 |
| PE | Thyroid | 5.51E-10 | 9.98E-10 | 0.1151123 | 0.290522 |
| PE | Brain_Caudate_(basal_ganglia) | 5.34E-10 | 1.02E-09 | 0.1243622 | 0.2996 |
| PE | Minor_Salivary_Gland | 5.92E-10 | 1.14E-09 | 0.1308772 | 0.301587 |
| PE | Brain_Nucleus_accumbens_(basal_ganglia) | 4.76E-10 | 1.05E-09 | 0.1474978 | 0.325724 |
| PE | Liver | 4.26E-10 | 1.08E-09 | 0.1632912 | 0.346177 |
| PE | Cervix_Endocervix | 4.02E-10 | 1.04E-09 | 0.1714774 | 0.34955 |
| PE | Testis | 2.87E-10 | 8.78E-10 | 0.1893951 | 0.371776 |
| PE | Skin_Sun_Exposed_(Lower_leg) | 3.46E-10 | 1.20E-09 | 0.2042792 | 0.386671 |
| PE | Brain_Spinal_cord_(cervical_c-1) | 2.13E-10 | 8.18E-10 | 0.2175097 | 0.397518 |
| PE | Pituitary | 2.54E-10 | 9.93E-10 | 0.2257894 | 0.398895 |
| PE | Skin_Not_Sun_Exposed_(Suprapubic) | 2.49E-10 | 1.10E-09 | 0.2401443 | 0.410569 |
| PE | Brain_Hypothalamus | 5.42E-11 | 9.33E-10 | 0.2879136 | 0.476857 |
| PE | Heart_Left_Ventricle | 4.75E-11 | 1.06E-09 | 0.3001801 | 0.482107 |
| PE | Bladder | -2.57E-11 | 8.73E-10 | 0.328304 | 0.511768 |
| PE | Artery_Coronary | -1.80E-10 | 1.02E-09 | 0.3765266 | 0.570169 |
| PE | Colon_Sigmoid | -1.99E-10 | 9.59E-10 | 0.3955408 | 0.582324 |
| PE | Brain_Substantia_nigra | -2.54E-10 | 8.57E-10 | 0.4304129 | 0.616537 |
| PE | Pancreas | -4.00E-10 | 1.11E-09 | 0.4594741 | 0.640845 |
| PE | Prostate | -4.41E-10 | 1.20E-09 | 0.4730196 | 0.642821 |
| PE | Nerve_Tibial | -4.49E-10 | 9.87E-10 | 0.5097735 | 0.67545 |
| PE | Esophagus_Mucosa | -5.51E-10 | 1.15E-09 | 0.5291705 | 0.68405 |
| PE | Esophagus_Gastroesophageal_Junction | -5.84E-10 | 9.73E-10 | 0.5752199 | 0.725873 |
| PE | Brain_Hippocampus | -5.41E-10 | 8.96E-10 | 0.5897565 | 0.726909 |
| PE | Brain_Amygdala | -5.31E-10 | 8.76E-10 | 0.6042644 | 0.727864 |
| PE | Heart_Atrial_Appendage | -5.99E-10 | 9.19E-10 | 0.6305405 | 0.742637 |
| PE | Muscle_Skeletal | -7.09E-10 | 1.02E-09 | 0.6570944 | 0.757087 |
| PE | Brain_Anterior_cingulate_cortex_(BA24) | -6.63E-10 | 9.35E-10 | 0.6747081 | 0.760841 |
| PE | Brain_Frontal_Cortex_(BA9) | -7.91E-10 | 9.55E-10 | 0.7211554 | 0.796276 |
| PE | Whole_Blood | -1.03E-09 | 1.19E-09 | 0.7460088 | 0.806907 |
| PE | Kidney_Cortex | -1.31E-09 | 1.13E-09 | 0.8282544 | 0.87795 |
| PE | Esophagus_Muscularis | -1.31E-09 | 1.00E-09 | 0.8702732 | 0.904402 |
| PE | Artery_Aorta | -1.67E-09 | 1.05E-09 | 0.9269996 | 0.944826 |
| PE | Brain_Cortex | -1.60E-09 | 9.20E-10 | 0.9592281 | 0.959228 |
