# Supplementary material for: Genetic Correlation and Causal Inference Between Female Fat Distribution and Preeclampsia: An Integrative Genomic Study
Source: FASEB J. 2026 Jun 23;40(12):e72074. doi: 10.1096/fj.202601888R (PMC13288445; doi:10.1096/fj.202601888R)
Supplement: Supplementary file 8 — Table S8: The significant results of SNP heritability enrichment analysis of WHR and PE at cell level. Prop_SN: Proportion of SNP heritability attributable to the given cell type or tissue; Prop_h2: Proportion of total heritability explained by the given cell type or tissue; Prop_h2_SE: Standard error of the heritability proportion estimate; Enrichment: Enrichment score indicating the relative SNP heritability contribution of the cell type or tissue; Enrichment_SE: Standard error of the enrichment score; Enrichment_P: P for the enrichment score; cellType: The specific cell type within the analyzed tissue. Other columns have been clarified in previous tables. [file FSB2-40-e72074-s003.docx]

| **Supplementary Table S8** | | | |  |  |  |  |  |  |  |  |
| --- | --- | --- | --- | --- | --- | --- | --- | --- | --- | --- | --- |
| ***The significant results of SNP heritability enrichment analysis of WHR and PE at cell level.*** *Prop_SN: Proportion of SNP heritability attributable to the given cell type or tissue; Prop_h2: Proportion of total heritability explained by the given cell type or tissue; Prop_h2_SE: Standard error of the heritability proportion estimate; Enrichment: Enrichment score indicating the relative SNP heritability contribution of the cell type or tissue; Enrichment_SE: Standard error of the enrichment score; Enrichment_P: P for the enrichment score; cellType: The specific cell type within the analyzed tissue.* *Other columns have been clarified in previous tables.* | | | | | | | | | | | |
| **Trait** | **Tissue** | **Prop._SNPs** | **Prop._h2** | **Prop._h2_SE** | **Enrichment** | **Enrichment_SE** | **Enrichment_*P*** | **Coefficient** | **Coefficient_SE** | **Coefficient_Z** | **cellType** |
| WHR | Blood | 0.16496 | 0.239856 | 0.011446387 | 1.454028055 | 0.069388897 | 7.53729E-10 | -9.49488E-09 | 7.0391E-09 | -1.348877146 | alpha_beta_T_cell |
| WHR | Blood | 0.272918 | 0.387933 | 0.018744696 | 1.421429285 | 0.068682574 | 1.0979E-08 | -1.60389E-09 | 5.9718E-09 | -0.268577986 | basophil |
| WHR | Blood | 0.142754 | 0.207258 | 0.012020133 | 1.451851799 | 0.084201632 | 1.93515E-07 | -1.60097E-09 | 7.45987E-09 | -0.214611006 | Cd141_positive_myeloid_dendritic_cell |
| WHR | Blood | 0.319029 | 0.402772 | 0.018600252 | 1.262491989 | 0.058302681 | 6.28155E-06 | -9.65877E-09 | 3.63695E-09 | -2.655731812 | cd4_positive_alpha_beta_memory_t_cell |
| WHR | Blood | 0.303706 | 0.3934 | 0.019297415 | 1.295333153 | 0.063539789 | 3.46358E-06 | -8.39176E-09 | 3.59287E-09 | -2.335673812 | CD4_t_cell |
| WHR | Blood | 0.327827 | 0.434874 | 0.019344458 | 1.326535396 | 0.059008087 | 1.07045E-07 | -7.36276E-09 | 3.62957E-09 | -2.028549044 | cd8_positive_alpha_beta_cytokine_secreting_effector_t_cell |
| WHR | Blood | 0.257115 | 0.34322 | 0.01782738 | 1.334891256 | 0.069336257 | 1.55875E-06 | -7.79309E-09 | 4.06682E-09 | -1.916263244 | cd8_positive_alpha_beta_t_cell |
| WHR | Blood | 0.223345 | 0.301125 | 0.018560638 | 1.348252046 | 0.083103138 | 3.07848E-05 | -8.2559E-09 | 4.28691E-09 | -1.925837871 | CD8_t_cell |
| WHR | Blood | 0.522855 | 0.677818 | 0.017766929 | 1.296377286 | 0.033980587 | 3.45907E-15 | -2.71437E-09 | 3.28549E-09 | -0.826167759 | classical_monocyte |
| WHR | Blood | 0.023577 | 0.038268 | 0.005714442 | 1.623091516 | 0.242371305 | 0.01061831 | -2.62353E-09 | 9.25922E-09 | -0.283342217 | erythrocyte |
| WHR | Blood | 0.351054 | 0.480906 | 0.018961219 | 1.369892872 | 0.054012279 | 1.41192E-10 | -3.14288E-09 | 4.17497E-09 | -0.752791346 | hematopoietic_stem_cell |
| WHR | Blood | 0.101764 | 0.163173 | 0.012459735 | 1.603438729 | 0.122437356 | 3.20447E-06 | -7.25731E-09 | 5.34222E-09 | -1.358482028 | macrophage |
| WHR | Blood | 0.284439 | 0.381692 | 0.019595493 | 1.341910404 | 0.068891747 | 2.05976E-06 | -3.85695E-09 | 4.63521E-09 | -0.832098875 | memory_b_cell |
| WHR | Blood | 0.446776 | 0.605785 | 0.019472337 | 1.355902184 | 0.043584108 | 2.23411E-13 | -3.24429E-09 | 3.27293E-09 | -0.99125042 | monocyte |
| WHR | Blood | 0.202735 | 0.26254 | 0.014094266 | 1.294990965 | 0.069520547 | 2.50498E-05 | -3.81338E-09 | 3.6708E-09 | -1.038842507 | naive_b_cell |
| WHR | Blood | 0.202149 | 0.273098 | 0.013648717 | 1.350972134 | 0.067518101 | 1.61727E-07 | -8.2805E-09 | 3.74435E-09 | -2.211464372 | naive_thymus_derived_cd4_positive_alpha_beta_t_cell |
| WHR | Blood | 0.004317 | 0.005959 | 0.000854454 | 1.380484958 | 0.197945236 | 0.051213143 | -2.1156E-08 | 2.02546E-08 | -1.044505981 | NAMPT_neutrophil |
| WHR | Blood | 0.171402 | 0.25964 | 0.014586495 | 1.514798426 | 0.085101002 | 2.59665E-09 | -3.51838E-11 | 3.6019E-09 | -0.009768112 | neutrophil |
| WHR | Blood | 0.316652 | 0.427406 | 0.019657069 | 1.349764646 | 0.062077776 | 7.54736E-08 | -5.94382E-09 | 3.74336E-09 | -1.587830362 | nk_cell |
| WHR | Blood | 0.204197 | 0.280912 | 0.01766197 | 1.375692357 | 0.086494928 | 1.48105E-05 | -8.89854E-09 | 4.22002E-09 | -2.108646994 | NK_cell |
| WHR | Blood | 0.427054 | 0.570248 | 0.019797096 | 1.335305149 | 0.046357332 | 5.11063E-12 | -2.24328E-09 | 4.08117E-09 | -0.549665017 | plasma_cell |
| WHR | Blood | 0.1277 | 0.192146 | 0.011999484 | 1.504671398 | 0.093966249 | 4.53662E-07 | 3.51007E-09 | 1.1776E-08 | 0.29807054 | plasmacytoid_dendritic_cell |
| WHR | Blood | 0.097799 | 0.15878 | 0.015738179 | 1.623535012 | 0.160924158 | 0.000196947 | 2.96585E-09 | 7.15608E-09 | 0.414451963 | platelet |
| WHR | Blood | 0.281817 | 0.388348 | 0.019701751 | 1.378015665 | 0.069909707 | 2.51112E-07 | -4.61671E-09 | 4.20892E-09 | -1.096886415 | type_I_NK_T_cell |
| WHR | Adipose tissue | 0.002783 | 0.001826 | 0.000295473 | 0.656187066 | 0.106160383 | 0.00115078 | -1.49085E-07 | 6.64247E-08 | -2.244417451 | B_Cells |
| WHR | Adipose tissue | 0.381677 | 0.499378 | 0.019645813 | 1.308378079 | 0.051472324 | 2.87347E-09 | -6.2422E-09 | 3.46418E-09 | -1.801928246 | Endothelial_cells |
| WHR | Adipose tissue | 0.361475 | 0.528708 | 0.01865749 | 1.462643682 | 0.051614949 | 1.17137E-15 | 4.17632E-09 | 3.11089E-09 | 1.342483306 | Fibroblasts |
| WHR | Adipose tissue | 0.008703 | 0.004783 | 0.001641254 | 0.549517555 | 0.188582673 | 0.018794662 | -7.32394E-08 | 2.85014E-08 | -2.569680024 | Leucocytes |
| WHR | Adipose tissue | 0.343853 | 0.479558 | 0.019363428 | 1.394660921 | 0.056313171 | 3.09033E-10 | -2.40136E-09 | 3.92808E-09 | -0.611332315 | Macrophages |
| WHR | Adipose tissue | 0.438053 | 0.616238 | 0.019365864 | 1.40676497 | 0.044208953 | 1.19021E-16 | 2.86416E-09 | 3.50003E-09 | 0.818323529 | Mesenchymal_Stem_Cells |
| WHR | Adipose tissue | 0.174061 | 0.271269 | 0.015261422 | 1.558471691 | 0.087678649 | 5.69877E-10 | -2.56696E-09 | 4.62718E-09 | -0.554758368 | Myofibroblasts |
| WHR | Adipose tissue | 0.108236 | 0.137493 | 0.009594744 | 1.270300468 | 0.088646342 | 0.001849868 | -1.37621E-08 | 3.76839E-09 | -3.651967592 | Natural_Killar_cells |
| WHR | Adipose tissue | 0.102835 | 0.162963 | 0.012209492 | 1.584698096 | 0.118728677 | 1.0064E-06 | -1.9296E-09 | 4.57949E-09 | -0.421355862 | Neutrophil |
| WHR | Adipose tissue | 0.049382 | 0.08574 | 0.009680738 | 1.736269063 | 0.196039661 | 0.000206261 | 9.83744E-09 | 1.47462E-08 | 0.667118686 | Plasma_cells |
| WHR | Adipose tissue | 0.058081 | 0.08313 | 0.004473568 | 1.431278545 | 0.077022659 | 2.48048E-08 | -2.21748E-08 | 6.20512E-09 | -3.57362168 | Smooth_muscle_cells |
| WHR | Adipose tissue | 0.111974 | 0.144362 | 0.01013945 | 1.28925203 | 0.090551963 | 0.001056901 | -1.13331E-08 | 3.90883E-09 | -2.899356697 | T_Cells |
| WHR | Ovary | 0.487543 | 0.624621 | 0.01823956 | 1.281159682 | 0.037411151 | 2.59677E-13 | -5.57092E-09 | 3.18829E-09 | -1.747309532 | endothelial_cell |
| WHR | Ovary | 0.299552 | 0.392681 | 0.018645384 | 1.310893971 | 0.062244206 | 2.18691E-06 | -4.92117E-09 | 4.61111E-09 | -1.067241211 | glandular_cell |
| WHR | Ovary | 0.59655 | 0.734947 | 0.016033774 | 1.231995299 | 0.02687749 | 1.23155E-15 | -2.71723E-09 | 2.76941E-09 | -0.981161522 | granulosa_cell |
| WHR | Ovary | 0.372439 | 0.51916 | 0.020217898 | 1.393944742 | 0.054285064 | 1.83079E-10 | -2.60641E-09 | 3.89897E-09 | -0.668486619 | immune_cell |
| WHR | Ovary | 0.552063 | 0.657497 | 0.01608035 | 1.190982563 | 0.029127745 | 3.92919E-10 | -2.83807E-09 | 3.72654E-09 | -0.76158424 | oocyte |
| WHR | Ovary | 0.308255 | 0.425635 | 0.018710056 | 1.380789154 | 0.060696763 | 5.60972E-09 | -6.19576E-11 | 4.72496E-09 | -0.013112835 | ovarian_surface_epithelial_cell |
| WHR | Ovary | 0.387378 | 0.509632 | 0.019964429 | 1.315593337 | 0.051537314 | 1.5707E-08 | -3.32885E-09 | 3.89542E-09 | -0.854554555 | smooth_muscle_cell |
| WHR | Ovary | 0.181469 | 0.270535 | 0.015152352 | 1.490804638 | 0.083498132 | 6.57363E-09 | -3.09305E-09 | 3.81262E-09 | -0.811265314 | smooth_muscle_pericyte |
| WHR | Ovary | 0.098007 | 0.161777 | 0.012936777 | 1.650673107 | 0.131998905 | 1.53366E-06 | 6.39156E-09 | 5.75441E-09 | 1.110722997 | stromal_cell_of_ovary |
| WHR | Ovary | 0.28473 | 0.377885 | 0.019012467 | 1.327169399 | 0.066773718 | 5.04744E-06 | -3.8572E-09 | 4.43488E-09 | -0.869740682 | stromal_cell |
| WHR | Ovary | 0.561776 | 0.747874 | 0.016138862 | 1.331268666 | 0.028728299 | 1.82698E-21 | 3.8964E-09 | 3.03235E-09 | 1.284943758 | theca_cell |
| WHR | Ovary | 0.522069 | 0.699409 | 0.019487698 | 1.339686734 | 0.037327819 | 4.13211E-17 | -1.73317E-09 | 3.45194E-09 | -0.502083956 | theca_stroma |
| WHR | Ovary | 0.247191 | 0.345774 | 0.015490161 | 1.398810442 | 0.062664653 | 8.57614E-10 | -2.44593E-09 | 4.65019E-09 | -0.525985285 | V2 |
| WHR | Uterus | 0.195045 | 0.273172 | 0.013353617 | 1.40055898 | 0.068464396 | 2.59367E-08 | -5.49383E-09 | 5.12081E-09 | -1.072844371 | Ciliated_epithelium |
| WHR | Uterus | 0.405316 | 0.534225 | 0.018106508 | 1.318045825 | 0.044672569 | 4.96807E-11 | -8.33696E-09 | 4.17229E-09 | -1.998171575 | Cycling_epithelial_cells |
| WHR | Uterus | 0.108497 | 0.158976 | 0.009197557 | 1.465251458 | 0.084772353 | 1.30204E-07 | -1.10786E-08 | 7.29996E-09 | -1.517623651 | Cycling_immune_cells |
| WHR | Uterus | 0.440308 | 0.577673 | 0.018832458 | 1.311973851 | 0.042771066 | 5.37373E-12 | -1.92664E-09 | 3.17549E-09 | -0.606721321 | Endometrial_stromal_fibbroblast |
| WHR | Uterus | 0.282475 | 0.409381 | 0.01808067 | 1.449265072 | 0.06400807 | 2.0691E-12 | -4.24973E-09 | 3.79226E-09 | -1.120633857 | Endothelium |
| WHR | Uterus | 0.343291 | 0.470166 | 0.018325495 | 1.36958601 | 0.053381846 | 3.42625E-11 | -1.03122E-08 | 4.25355E-09 | -2.424386583 | Lymphatic_endothelium |
| WHR | Uterus | 0.170248 | 0.263295 | 0.01770575 | 1.546541641 | 0.103999994 | 9.82114E-07 | -7.59167E-10 | 5.05354E-09 | -0.150224856 | Macrophages |
| WHR | Uterus | 0.070389 | 0.108426 | 0.007109863 | 1.540388144 | 0.101008765 | 6.94282E-08 | -1.15004E-08 | 5.33347E-09 | -2.156263292 | Myometrial_smooth_muscle |
| WHR | Uterus | 0.089935 | 0.151475 | 0.010394821 | 1.684269951 | 0.115581573 | 6.73223E-09 | 1.38454E-09 | 7.01253E-09 | 0.197438371 | Pericyte |
| WHR | Uterus | 0.072628 | 0.088823 | 0.00909849 | 1.222982326 | 0.125274775 | 0.070275361 | -1.48148E-08 | 5.32678E-09 | -2.781185632 | T_lymphocytes |
| WHR | Uterus | 0.234042 | 0.318066 | 0.01575733 | 1.35901445 | 0.067326977 | 2.47771E-07 | -6.89022E-09 | 4.22038E-09 | -1.632606282 | Unciliated_epithelium |
| WHR | Uterus | 0.102012 | 0.188892 | 0.014508302 | 1.851657505 | 0.142221113 | 2.90135E-08 | 1.22442E-08 | 7.34368E-09 | 1.66731239 | Uterine_fibroblast |
| WHR | Uterus | 0.060691 | 0.09383 | 0.009495948 | 1.546045051 | 0.15646507 | 0.000594808 | -5.83031E-09 | 9.26306E-09 | -0.629415315 | Uterine_NK_cells |
| WHR | Uterus | 0.099075 | 0.159493 | 0.013303576 | 1.609823327 | 0.134278268 | 5.35112E-06 | -4.343E-09 | 6.37465E-09 | -0.681291732 | Vascular_smooth_muscle |
| BMI | Blood | 0.16496 | 0.197763 | 0.006825783 | 1.198857238 | 0.041378432 | 1.26614E-06 | 1.0439E-09 | 4.83348E-09 | 0.215972822 | Alpha Beta T Cell |
| BMI | Blood | 0.272918 | 0.323395 | 0.011242079 | 1.184953712 | 0.041192182 | 6.62413E-06 | 1.98743E-09 | 2.69181E-09 | 0.738322358 | Basophil |
| BMI | Blood | 0.142754 | 0.168366 | 0.006715059 | 1.179411237 | 0.047039322 | 0.00011849 | -2.34604E-10 | 3.75813E-09 | -0.06242578 | Cd141 Positive Myeloid Dendritic Cell |
| BMI | Blood | 0.319029 | 0.358575 | 0.0114164 | 1.123956731 | 0.035784822 | 0.00051988 | -3.12384E-09 | 2.06594E-09 | -1.512068076 | Cd4 Positive Alpha Beta Memory T Cell |
| BMI | Blood | 0.303706 | 0.349411 | 0.013416651 | 1.15049037 | 0.044176443 | 0.000687822 | -5.17562E-10 | 2.60764E-09 | -0.198479056 | Cd4 T Cell |
| BMI | Blood | 0.327827 | 0.380769 | 0.012667319 | 1.161491596 | 0.038640228 | 2.7004E-05 | -1.40181E-10 | 2.08008E-09 | -0.067392028 | Cd8 Positive Alpha Beta Cytokine Secreting Effector T Cell |
| BMI | Blood | 0.257115 | 0.300199 | 0.011391237 | 1.167566334 | 0.044304085 | 0.000169083 | -5.40414E-10 | 2.42323E-09 | -0.223013937 | Cd8 Positive Alpha Beta T Cell |
| BMI | Blood | 0.223345 | 0.263472 | 0.011576837 | 1.179667582 | 0.051833963 | 0.000524074 | 4.41586E-10 | 2.45002E-09 | 0.180237642 | Cd8 T Cell |
| BMI | Blood | 0.522855 | 0.559928 | 0.014336513 | 1.070905347 | 0.027419659 | 0.008534131 | -4.58322E-09 | 2.35956E-09 | -1.942402565 | Classical Monocyte |
| BMI | Blood | 0.023577 | 0.035749 | 0.003402049 | 1.516271244 | 0.144293884 | 0.000343158 | 7.34745E-09 | 5.75615E-09 | 1.276453901 | Erythrocyte |
| BMI | Blood | 0.351054 | 0.410559 | 0.013075147 | 1.169502924 | 0.037245417 | 5.65931E-06 | 7.06019E-10 | 2.59809E-09 | 0.271745766 | Hematopoietic Stem Cell |
| BMI | Blood | 0.101764 | 0.126991 | 0.008795073 | 1.247892092 | 0.086426034 | 0.004110692 | 1.75661E-10 | 3.7017E-09 | 0.047454015 | Macrophage |
| BMI | Blood | 0.284439 | 0.324149 | 0.010030533 | 1.139608171 | 0.035264279 | 6.20225E-05 | -1.81779E-09 | 2.40019E-09 | -0.757354491 | Memory B Cell |
| BMI | Blood | 0.446776 | 0.495797 | 0.014303938 | 1.109722498 | 0.032015899 | 0.000561007 | -1.78116E-09 | 2.42032E-09 | -0.735919076 | Monocyte |
| BMI | Blood | 0.202735 | 0.228559 | 0.008784257 | 1.127376328 | 0.043328712 | 0.00289387 | -5.01324E-10 | 2.39169E-09 | -0.209610807 | Naive B Cell |
| BMI | Blood | 0.202149 | 0.240954 | 0.00882055 | 1.191960668 | 0.043633905 | 1.20539E-05 | -5.91575E-10 | 2.2072E-09 | -0.268020918 | Naive Thymus Derived Cd4 Positive Alpha Beta T Cell |
| BMI | Blood | 0.171402 | 0.192859 | 0.010075178 | 1.125183152 | 0.058780928 | 0.03193865 | -2.41742E-11 | 2.50176E-09 | -0.009662871 | Neutrophil |
| BMI | Blood | 0.316652 | 0.360971 | 0.012659407 | 1.139960339 | 0.039978889 | 0.000451083 | -2.09244E-09 | 2.2072E-09 | -0.948008985 | Nk Cell |
| BMI | Blood | 0.204197 | 0.249199 | 0.01176172 | 1.220385748 | 0.05759998 | 0.000158969 | 1.84008E-09 | 2.82833E-09 | 0.650590373 | Nk Cell |
| BMI | Blood | 0.427054 | 0.474933 | 0.013190107 | 1.112113999 | 0.030886255 | 0.000261439 | -2.60578E-09 | 2.11053E-09 | -1.234656928 | Plasma Cell |
| BMI | Blood | 0.1277 | 0.156728 | 0.006609979 | 1.227312068 | 0.0517618 | 1.34375E-05 | 4.94887E-09 | 5.64616E-09 | 0.876501308 | Plasmacytoid Dendritic Cell |
| BMI | Blood | 0.097799 | 0.126579 | 0.007542866 | 1.294285227 | 0.077126415 | 0.000122819 | 4.87358E-09 | 3.29985E-09 | 1.476911665 | Platelet |
| BMI | Blood | 0.281817 | 0.332556 | 0.011999229 | 1.180040633 | 0.042578072 | 2.46068E-05 | 8.6759E-10 | 2.18663E-09 | 0.396770075 | Type I Nk T Cell |
| BMI | Adipose | 0.381677 | 0.432792 | 0.012214555 | 1.133920967 | 0.032002317 | 3.21659E-05 | 1.12344E-10 | 2.22444E-09 | 0.050504476 | Endothelial Cells |
| BMI | Adipose | 0.361475 | 0.410164 | 0.01329181 | 1.134695684 | 0.036771083 | 0.000253587 | -3.83815E-09 | 2.22021E-09 | -1.728734148 | Fibroblasts |
| BMI | Adipose | 0.343853 | 0.391006 | 0.011987783 | 1.137131522 | 0.034863149 | 6.87329E-05 | 1.07003E-09 | 2.29329E-09 | 0.466589355 | Macrophages |
| BMI | Adipose | 0.438053 | 0.495748 | 0.014279384 | 1.131707138 | 0.032597389 | 4.58398E-05 | -3.92905E-09 | 2.43516E-09 | -1.613465622 | Mesenchymal Stem Cells |
| BMI | Adipose | 0.174061 | 0.224936 | 0.007734213 | 1.292284425 | 0.044433952 | 4.28355E-11 | 1.59738E-09 | 2.36247E-09 | 0.676147169 | Myofibroblasts |
| BMI | Adipose | 0.108236 | 0.126851 | 0.007356763 | 1.171979171 | 0.067969515 | 0.012434399 | 1.41964E-09 | 3.10033E-09 | 0.457899298 | Natural Killar Cells |
| BMI | Adipose | 0.049382 | 0.06182 | 0.003512216 | 1.251886764 | 0.071124089 | 0.000485923 | 6.16581E-10 | 5.59512E-09 | 0.110199639 | Plasma Cells |
| BMI | Adipose | 0.058081 | 0.074774 | 0.003488087 | 1.287409055 | 0.060055347 | 1.36883E-06 | 4.37667E-11 | 5.61487E-09 | 0.007794789 | Smooth Muscle Cells |
| BMI | Ovary | 0.487543 | 0.544186 | 0.013685728 | 1.116178872 | 0.028070788 | 3.68937E-05 | 5.20428E-10 | 2.46389E-09 | 0.211221774 | Endothelial Cell |
| BMI | Ovary | 0.299552 | 0.343528 | 0.008720767 | 1.146805614 | 0.029112687 | 3.60714E-07 | -3.82633E-09 | 2.22241E-09 | -1.721700519 | Glandular Cell |
| BMI | Ovary | 0.59655 | 0.653953 | 0.013262357 | 1.096225043 | 0.02223175 | 1.69515E-05 | -3.03903E-10 | 2.73523E-09 | -0.111107221 | Granulosa Cell |
| BMI | Ovary | 0.372439 | 0.408134 | 0.011535752 | 1.095838739 | 0.030973498 | 0.001642935 | -3.59025E-09 | 2.39413E-09 | -1.499603497 | Immune Cell |
| BMI | Ovary | 0.552063 | 0.603549 | 0.012785123 | 1.093260779 | 0.023158813 | 4.89992E-05 | -9.68213E-10 | 2.87098E-09 | -0.337241881 | Oocyte |
| BMI | Ovary | 0.308255 | 0.351363 | 0.009422769 | 1.139845737 | 0.030568138 | 5.36988E-06 | -2.40085E-09 | 2.32674E-09 | -1.031853447 | Ovarian Surface Epithelial Cell |
| BMI | Ovary | 0.387378 | 0.429182 | 0.013081926 | 1.107913751 | 0.033770427 | 0.001505206 | -3.88319E-10 | 2.13651E-09 | -0.181754299 | Smooth Muscle Cell |
| BMI | Ovary | 0.181469 | 0.222942 | 0.008489586 | 1.228537426 | 0.046782477 | 7.28023E-07 | 1.2958E-09 | 2.18197E-09 | 0.593868973 | Smooth Muscle Pericyte |
| BMI | Ovary | 0.098007 | 0.120224 | 0.006560508 | 1.226690366 | 0.066939383 | 0.000816262 | -8.51556E-10 | 3.05136E-09 | -0.279073869 | Stromal Cell Of Ovary |
| BMI | Ovary | 0.28473 | 0.308435 | 0.010923973 | 1.083254906 | 0.038366104 | 0.029187026 | -2.97441E-09 | 2.517E-09 | -1.181727884 | Stromal Cell |
| BMI | Ovary | 0.561776 | 0.617964 | 0.013579231 | 1.100018375 | 0.024171977 | 3.66094E-05 | -1.08425E-09 | 2.62831E-09 | -0.41252848 | Theca Cell |
| BMI | Ovary | 0.522069 | 0.57953 | 0.013504966 | 1.11006441 | 0.025868161 | 1.53171E-05 | -3.57568E-09 | 2.38689E-09 | -1.498048305 | Theca Stroma |
| BMI | Uterus | 0.195045 | 0.233627 | 0.008669119 | 1.197812984 | 0.044446833 | 1.02757E-05 | -5.75792E-10 | 3.04385E-09 | -0.189165811 | Ciliated Epithelium |
| BMI | Uterus | 0.405316 | 0.453626 | 0.012056816 | 1.119191299 | 0.029746704 | 5.89869E-05 | -2.93347E-09 | 2.51946E-09 | -1.164324379 | Cycling Epithelial Cells |
| BMI | Uterus | 0.108497 | 0.128255 | 0.007173423 | 1.182103885 | 0.066116253 | 0.005588136 | 7.41385E-10 | 5.39984E-09 | 0.137297633 | Cycling Immune Cells |
| BMI | Uterus | 0.440308 | 0.493227 | 0.012684807 | 1.120186403 | 0.028808917 | 2.76439E-05 | -4.28538E-09 | 2.16448E-09 | -1.979862836 | Endometrial Stromal Fibbroblast |
| BMI | Uterus | 0.282475 | 0.317644 | 0.01074718 | 1.124505102 | 0.038046503 | 0.000848168 | -2.23188E-09 | 1.95728E-09 | -1.140293733 | Endothelium |
| BMI | Uterus | 0.343291 | 0.391574 | 0.011032696 | 1.14064961 | 0.03213805 | 1.00609E-05 | -3.02124E-09 | 2.28804E-09 | -1.320447023 | Lymphatic Endothelium |
| BMI | Uterus | 0.070389 | 0.089399 | 0.004031629 | 1.27008509 | 0.057276752 | 2.17685E-06 | 2.67224E-10 | 2.92078E-09 | 0.091490561 | Myometrial Smooth Muscle |
| BMI | Uterus | 0.089935 | 0.112818 | 0.005448816 | 1.254438757 | 0.060586199 | 2.17171E-05 | 7.15506E-10 | 3.86587E-09 | 0.185082779 | Pericyte |
| BMI | Uterus | 0.234042 | 0.265709 | 0.009086751 | 1.135305477 | 0.038825328 | 0.000515402 | -2.74579E-09 | 2.59651E-09 | -1.05749389 | Unciliated Epithelium |
| BMI | Uterus | 0.102012 | 0.125475 | 0.006992166 | 1.230001918 | 0.068542389 | 0.000890314 | -2.02617E-09 | 3.34307E-09 | -0.606082881 | Uterine Fibroblast |
| BMI | Uterus | 0.099075 | 0.128204 | 0.005773313 | 1.294009644 | 0.058272336 | 5.81784E-07 | 1.17564E-09 | 2.72809E-09 | 0.43093754 | Vascular Smooth Muscle |
