# Supplementary material for: Genetic Correlation and Causal Inference Between Female Fat Distribution and Preeclampsia: An Integrative Genomic Study
Source: FASEB J. 2026 Jun 23;40(12):e72074. doi: 10.1096/fj.202601888R (PMC13288445; doi:10.1096/fj.202601888R)
Supplement: Supplementary file 9 — Table S9: The results for WHR and PE: gene‐sets implicated by GSA‐MIXeR analysis (AIC > 0). GO: name of the gene‐set from GO or SynGO database; NGENES: number of genes in the gene‐set, after filtering on genes used in GSA‐MiXeR analysis; enrich: MiXeR fold‐enrichment of heritability; se_enrich: standard error of enrich estimate; h2_frac: gene‐set's fraction of heritability over heritability of all protein‐coding genes; se_h2_frac: standard error of h2_frac; h2_base_frac: same as h2_frac; se_h2_base_frac: standard error of h2_base_frac; excl_GENE: it reports which gene within gene set leads to lower enrichment of the remaining genes after excluding excl_GENE; excl_enrich: enrichment of all remaining genes in the gene‐set after exclusion of the excl_GENE; se_excl_enrich: standard error of excl_GENE; MIXER_AIC: the value of Akaike information criterion, computed as 2*loglike_diff: 2*loglike_df; positive MIXER_AIC indicates that full MiXeR model (with gene‐specific effect size variance) has a better fit than the baseline model in the genomic region defined by the GO column; loglike_diff: improvement in the log‐likelihood in the genomic region defined by the GO column; loglike_df: number of parameters fitted for the region defined by the GO column; GENE_LIST: this gives the list of genes included in the gene set. [file FSB2-40-e72074-s014.docx]

| **Supplementary Table S9** | |  |  |  |  |  |  |  |  |  |  |  |  |  |
| --- | --- | --- | --- | --- | --- | --- | --- | --- | --- | --- | --- | --- | --- | --- |
| ***The results for WHR and PE: gene-sets implicated by GSA-MIXeR analysis (AIC > 0)****. GO: name of the gene-set from GO or SynGO database; NGENES: number of genes in the gene-set, after filtering on genes used in GSA-MiXeR analysis; enrich: MiXeR fold-enrichment of heritability; se_enrich: standard error of enrich estimate; h2_frac: gene-set's fraction of heritability over heritability of all protein-coding genes; se_h2_frac: standard error of h2_frac; h2_base_frac: same as h2_frac; se_h2_base_frac: standard error of h2_base_frac; excl_GENE: it reports which gene within gene set leads to lower enrichment of the remaining genes after excluding excl_GENE; excl_enrich: enrichment of all remaining genes in the gene-set after exclusion of the excl_GENE; se_excl_enrich: standard error of excl_GENE; MIXER_AIC: the value of Akaike information criterion, computed as 2*loglike_diff: 2*loglike_df; positive MIXER_AIC indicates that full MiXeR model (with gene-specific effect size variance) has a better fit than the baseline model in the genomic region defined by the GO column; loglike_diff: improvement in the log-likelihood in the genomic region defined by the GO column; loglike_df: number of parameters fitted for the region defined by the GO column; GENE_LIST: this gives the list of genes included in the gene set.* | | | | | | | | | | | | | | |
| **Trait** | **GO** | **NGENES** | **enrich** | **se_enrich** | **MIXER_AIC** | **loglike_diff** | **loglike_df** | **h2_frac** | **se_h2_frac** | **h2_base_frac** | **excl_GENE** | **excl_enrich** | **se_excl_enrich** | **GENE_LIST** |
| WHR | GOBP_POSITIVE_REGULATION_OF_MAST_CELL_CHEMOTAXIS | 5 | 23.25 | 8.81 | 34.03 | 31.02 | 14 | 0.00936 | 0.00355 | 0.00040 | VEGFA | 7.51 | 4.77 | PGF SWAP70 VEGFA VEGFB VEGFC |
| WHR | GOBP_REGULATION_OF_ADHERENS_JUNCTION_ORGANIZATION | 5 | 15.78 | 6.38 | 34.57 | 28.29 | 11 | 0.00854 | 0.00345 | 0.00054 | VEGFA | 2.42 | 1.27 | ADD1 BMP6 PTPN23 RDX VEGFA |
| WHR | GOBP_POSITIVE_REGULATION_OF_TROPHOBLAST_CELL_MIGRATION | 7 | 14.28 | 6.32 | 23.86 | 26.93 | 15 | 0.00771 | 0.00341 | 0.00054 | VEGFA | 0.16 | 0.17 | ACVR1B AGO2 APELA C1QBP SMURF2 SYDE1 VEGFA |
| WHR | GOBP_POSITIVE_REGULATION_OF_ENDOTHELIAL_CELL_CHEMOTAXIS_BY_VEGF_ACTIVATED_VASCULAR_ENDOTHELIAL_GROWTH_FACTOR_RECEPTOR_SIGNALING_PATHWAY | 5 | 13.59 | 5.92 | 38.12 | 27.06 | 8 | 0.00781 | 0.00340 | 0.00058 | VEGFA | 0.4 | 0.51 | HSPB1 KDR PRKD1 PRKD2 VEGFA |
| WHR | GOBP_VASCULAR_ENDOTHELIAL_GROWTH_FACTOR_RECEPTOR_2_SIGNALING_PATHWAY | 5 | 13.23 | 5.63 | 39.95 | 26.97 | 7 | 0.00805 | 0.00342 | 0.00061 | VEGFA | 0.92 | 0.84 | CLEC14A DAB2IP KDR PDCD6 VEGFA |
| WHR | GOBP_POSITIVE_REGULATION_OF_HISTONE_DEACETYLASE_ACTIVITY | 6 | 12.72 | 5.56 | 28.23 | 27.11 | 13 | 0.00781 | 0.00342 | 0.00061 | VEGFA | 0.36 | 0.35 | C6orf89 LRRK2 PINK1 PRKD1 PRKD2 VEGFA |
| WHR | GOBP_CARDIAC_VASCULAR_SMOOTH_MUSCLE_CELL_DIFFERENTIATION | 8 | 12.19 | 4.28 | 34.13 | 31.06 | 14 | 0.00975 | 0.00342 | 0.00080 | VEGFA | 3.35 | 1.93 | GATA6 GPER1 HEY2 MESP1 MYOCD NOTCH1 SRF VEGFA |
| WHR | GOBP_NEGATIVE_REGULATION_OF_ENDOTHELIAL_CELL_DIFFERENTIATION | 9 | 12.15 | 7.6 | 18.24 | 27.12 | 18 | 0.01023 | 0.00640 | 0.00084 | VEGFA | 3.86 | 7.65 | ACVRL1 FOXJ2 ID1 JAG1 NOTCH4 S1PR3 VEGFA XDH ZEB1 |
| WHR | GOBP_POSITIVE_REGULATION_OF_DEACETYLASE_ACTIVITY | 8 | 11.88 | 5.09 | 20.53 | 27.27 | 17 | 0.00793 | 0.00340 | 0.00067 | VEGFA | 0.57 | 0.45 | C6orf89 FNTA LRRK2 MAPK8 PINK1 PRKD1 PRKD2 VEGFA |
| WHR | GOBP_REGULATION_OF_MAST_CELL_CHEMOTAXIS | 7 | 11.68 | 4.32 | 24.42 | 31.21 | 19 | 0.00963 | 0.00356 | 0.00083 | VEGFA | 3.05 | 1.73 | PGF RAC2 RIN3 SWAP70 VEGFA VEGFB VEGFC |
| WHR | GOBP_VASCULAR_ASSOCIATED_SMOOTH_MUSCLE_CELL_DEVELOPMENT | 11 | 11.09 | 4.11 | 4.23 | 31.12 | 29 | 0.00953 | 0.00353 | 0.00086 | VEGFA | 2.74 | 1.63 | ADM COMP EDNRA EFEMP2 ENG HES1 HEY2 NOTCH1 RAMP2 SGCB VEGFA |
| WHR | GOBP_POSITIVE_REGULATION_OF_BLOOD_VESSEL_ENDOTHELIAL_CELL_PROLIFERATION_INVOLVED_IN_SPROUTING_ANGIOGENESIS | 9 | 11.05 | 4.72 | 30.38 | 27.19 | 12 | 0.00808 | 0.00345 | 0.00073 | VEGFA | 0.76 | 0.56 | AGTR1 APELA APLNR FGFBP1 GATA2 HMOX1 JCAD PPP1R16B VEGFA |
| WHR | GOBP_POSITIVE_REGULATION_OF_TRANSCRIPTION_FROM_RNA_POLYMERASE_II_PROMOTER_IN_RESPONSE_TO_HYPOXIA | 6 | 10.68 | 4.3 | 32.29 | 28.14 | 12 | 0.00841 | 0.00338 | 0.00079 | VEGFA | 1.23 | 0.81 | HIF1A NFE2L2 NOTCH1 RBPJ TP53 VEGFA |
| WHR | GOMF_VASCULAR_ENDOTHELIAL_GROWTH_FACTOR_RECEPTOR_BINDING | 13 | 9.56 | 3.25 | 8.71 | 32.35 | 28 | 0.01026 | 0.00349 | 0.00107 | VEGFA | 2.9 | 1.37 | CADM4 CCDC88A CD2AP CDH5 DAB2IP GREM1 ITGA5 ITGB3 PDCL3 PGF VEGFA VEGFB VEGFC |
| WHR | GOMF_VASCULAR_ENDOTHELIAL_GROWTH_FACTOR_RECEPTOR_2_BINDING | 9 | 9.53 | 3.73 | 26.43 | 28.22 | 15 | 0.00855 | 0.00335 | 0.00090 | VEGFA | 1.24 | 0.79 | CADM4 CCDC88A CDH5 DAB2IP GREM1 ITGA5 ITGB3 PDCL3 VEGFA |
| WHR | GOBP_POST_EMBRYONIC_CAMERA_TYPE_EYE_DEVELOPMENT | 7 | 9.44 | 3.87 | 33.5 | 27.75 | 11 | 0.00850 | 0.00349 | 0.00090 | VEGFA | 1.17 | 0.81 | BAK1 BAX BCL11B FZD5 KDR KLF4 VEGFA |
| WHR | GOBP_REGULATION_OF_HISTONE_DEACETYLASE_ACTIVITY | 9 | 9.31 | 3.78 | 7.89 | 26.94 | 23 | 0.00833 | 0.00339 | 0.00090 | VEGFA | 0.94 | 0.62 | C6orf89 CAMK2D KDM5A LRRK2 PINK1 PRKD1 PRKD2 SPHK2 VEGFA |
| WHR | GOBP_REGULATION_OF_DEACETYLASE_ACTIVITY | 11 | 8.91 | 3.55 | 0.19 | 27.1 | 27 | 0.00845 | 0.00337 | 0.00095 | VEGFA | 1.03 | 0.64 | C6orf89 CAMK2D FNTA KDM5A LRRK2 MAPK8 PINK1 PRKD1 PRKD2 SPHK2 VEGFA |
| WHR | GOBP_PRIMITIVE_HEMOPOIESIS | 7 | 8.89 | 3.82 | 25 | 27.5 | 15 | 0.00791 | 0.00340 | 0.00089 | VEGFA | 0.36 | 0.28 | GATA2 STK3 STK4 TAL1 THOC5 VEGFA ZFPM1 |
| WHR | GOBP_POSITIVE_REGULATION_OF_CELL_MIGRATION_BY_VASCULAR_ENDOTHELIAL_GROWTH_FACTOR_SIGNALING_PATHWAY | 8 | 8.55 | 3.64 | 26.86 | 27.43 | 14 | 0.00805 | 0.00342 | 0.00094 | VEGFA | 0.52 | 0.36 | GAB1 HSPB1 KDR MYO1C PIK3CD PRKD1 PRKD2 VEGFA |
| WHR | GOBP_MAST_CELL_MIGRATION | 11 | 8.49 | 3.09 | 4.74 | 31.37 | 29 | 0.00984 | 0.00359 | 0.00116 | VEGFA | 2.22 | 1.14 | CHGA KIT PGF PIK3CD RAC2 RIN3 STAT5B SWAP70 VEGFA VEGFB VEGFC |
| WHR | GOBP_NEGATIVE_REGULATION_OF_CELL_CELL_ADHESION_MEDIATED_BY_CADHERIN | 9 | 8.43 | 3.41 | 15.19 | 28.6 | 21 | 0.00852 | 0.00345 | 0.00101 | VEGFA | 1.03 | 0.56 | BMP6 EPCAM MAD2L2 NOTCH1 NOTCH4 PLG PPM1F RGCC VEGFA |
| WHR | GOBP_POSITIVE_REGULATION_OF_WNT_SIGNALING_PATHWAY_PLANAR_CELL_POLARITY_PATHWAY | 8 | 8.24 | 2.25 | 68.38 | 53.19 | 19 | 0.00729 | 0.00199 | 0.00088 | RSPO3 | 1.93 | 0.98 | ABL1 ANKRD6 DAB2 DKK1 MLLT3 NKD1 PLEKHA4 RSPO3 |
| WHR | GOBP_REGULATION_OF_BLOOD_BRAIN_BARRIER_PERMEABILITY | 8 | 7.47 | 3.07 | 25.32 | 28.66 | 16 | 0.00852 | 0.00351 | 0.00114 | VEGFA | 0.9 | 0.59 | ABCC8 ANGPT1 OCLN SH3GL2 TJP1 TJP2 TJP3 VEGFA |
| WHR | GOBP_REGULATION_OF_RETINAL_GANGLION_CELL_AXON_GUIDANCE | 5 | 7.08 | 3.01 | 45.98 | 27.99 | 5 | 0.00809 | 0.00344 | 0.00114 | VEGFA | 0.45 | 0.59 | NRP1 POU4F2 PTPRO SLIT2 VEGFA |
| WHR | GOBP_BRANCHING_INVOLVED_IN_LABYRINTHINE_LAYER_MORPHOGENESIS | 13 | 6.59 | 1.81 | 48.77 | 50.39 | 26 | 0.00637 | 0.00175 | 0.00097 | RSPO3 | 0.68 | 0.45 | ADM FGFR2 FZD5 GCM1 GRB2 GRHL2 IL10 RSPO3 SOCS3 SPINT1 SPINT2 ST14 TMED2 |
| WHR | GOBP_POSITIVE_REGULATION_OF_PROTEIN_KINASE_C_SIGNALING | 10 | 6.52 | 2.61 | 19.7 | 28.85 | 19 | 0.00849 | 0.00339 | 0.00130 | VEGFA | 0.74 | 0.57 | ADGRV1 ADRA1A CD40 FLT4 MC1R PLA2G6 SPHK2 VEGFA WNT11 WNT5A |
| WHR | GOBP_REGULATION_OF_WNT_SIGNALING_PATHWAY_PLANAR_CELL_POLARITY_PATHWAY | 15 | 6.42 | 1.58 | 41.71 | 54.85 | 34 | 0.00841 | 0.00207 | 0.00131 | RSPO3 | 2.18 | 0.82 | ABL1 ANKRD6 DAB2 DACT1 DKK1 MKS1 MLLT3 NKD1 NPHP3 PLEKHA4 RSPO3 SFRP1 SFRP2 SPEF1 ZNRF3 |
| WHR | GOBP_POSITIVE_REGULATION_OF_NON_CANONICAL_WNT_SIGNALING_PATHWAY | 14 | 6.22 | 1.52 | 43.28 | 54.64 | 33 | 0.00825 | 0.00202 | 0.00133 | RSPO3 | 2.02 | 0.81 | ABL1 ANKRD6 CSNK1D CSNK1E DAB2 DKK1 LBX2 MLLT3 NKD1 PLEKHA4 RSPO3 SFRP1 WNT5A WNT5B |
| WHR | GOBP_POSITIVE_REGULATION_OF_ENDOTHELIAL_CELL_CHEMOTAXIS | 13 | 5.88 | 2.2 | 18.9 | 30.45 | 21 | 0.01027 | 0.00384 | 0.00175 | VEGFA | 1.67 | 0.85 | FGF18 FGF2 FGFR1 HSPB1 KDR LGMN MET P2RX4 PRKD1 PRKD2 SEMA5A SMOC2 VEGFA |
| WHR | GOBP_REGULATION_OF_NITRIC_OXIDE_MEDIATED_SIGNAL_TRANSDUCTION | 10 | 5.84 | 2.31 | 26.13 | 29.06 | 16 | 0.00896 | 0.00355 | 0.00153 | VEGFA | 0.96 | 0.7 | ATP2B4 EGFR GUCY1A1 GUCY1A2 INS NOS1AP PDE5A SPINK1 THBS1 VEGFA |
| WHR | GOBP_REGULATION_OF_AXON_GUIDANCE | 11 | 5.8 | 2.39 | 27.55 | 28.77 | 15 | 0.00835 | 0.00344 | 0.00144 | VEGFA | 0.55 | 0.52 | FEZF2 MYCBP2 NOVA2 NRP1 POU4F2 PTPRO SLIT2 TBR1 TUBB2B VEGFA YTHDF1 |
| WHR | GOBP_POSITIVE_REGULATION_OF_AXON_EXTENSION_INVOLVED_IN_AXON_GUIDANCE | 7 | 5.63 | 2.31 | 35.81 | 27.9 | 10 | 0.00856 | 0.00352 | 0.00152 | VEGFA | 0.67 | 0.44 | BMPR2 CXCL12 DSCAM MEGF8 NRP1 SEMA5A VEGFA |
| WHR | GOBP_VENOUS_BLOOD_VESSEL_MORPHOGENESIS | 9 | 5.37 | 2.26 | 27.68 | 27.84 | 14 | 0.00832 | 0.00350 | 0.00155 | VEGFA | 0.48 | 0.44 | CCBE1 CCM2 EFNB2 ENG HEG1 NOTCH1 PROX1 TBX20 VEGFA |
| WHR | GOBP_REGULATION_OF_ENDOTHELIAL_CELL_CHEMOTAXIS | 19 | 5.05 | 1.74 | 6.41 | 33.21 | 30 | 0.01159 | 0.00399 | 0.00230 | VEGFA | 1.86 | 0.82 | CXCL13 FGF1 FGF18 FGF2 FGF4 FGFR1 HRG HSPB1 KDR LGMN MET NOTCH1 P2RX4 PRKD1 PRKD2 SEMA5A SMOC2 THBS1 VEGFA |
| WHR | GOMF_PLATELET_DERIVED_GROWTH_FACTOR_RECEPTOR_BINDING | 13 | 5.03 | 2.11 | 17.85 | 29.93 | 21 | 0.00829 | 0.00347 | 0.00165 | VEGFA | 0.43 | 0.29 | ERN1 IL1R1 ITGA5 ITGB3 LYN PDGFA PDGFB PDGFC PDGFD PDGFRA PDGFRB PTPRJ VEGFA |
| WHR | GOBP_CORONARY_ARTERY_MORPHOGENESIS | 9 | 4.98 | 2.12 | 28.02 | 28.01 | 14 | 0.00802 | 0.00341 | 0.00161 | VEGFA | 0.25 | 0.19 | ARID2 HAND2 LRP2 NOTCH1 NRP1 SEC24B TBX1 TGFBR1 VEGFA |
| WHR | GOBP_POSITIVE_REGULATION_OF_MELANOCYTE_DIFFERENTIATION | 5 | 4.97 | 1.95 | 7.12 | 9.56 | 6 | 0.00526 | 0.00207 | 0.00106 | ADAMTS9 | 0.91 | 0.56 | ADAMTS20 ADAMTS9 BCL2 KITLG ZEB2 |
| WHR | GOBP_REGULATION_OF_MELANOCYTE_DIFFERENTIATION | 6 | 4.49 | 1.73 | 3.14 | 9.57 | 8 | 0.00537 | 0.00207 | 0.00120 | ADAMTS9 | 0.89 | 0.5 | ADAMTS20 ADAMTS9 BCL2 GNA11 KITLG ZEB2 |
| WHR | GOBP_VENOUS_BLOOD_VESSEL_DEVELOPMENT | 16 | 4.3 | 1.69 | 11.43 | 29.71 | 24 | 0.00963 | 0.00378 | 0.00224 | VEGFA | 0.96 | 0.56 | ACVR2B ACVRL1 APLNR BMPR2 CCBE1 CCM2 EFNB2 ENG FOXF1 HEG1 NKX2-5 NOTCH1 PROX1 SEMA3C TBX20 VEGFA |
| WHR | GOBP_LABYRINTHINE_LAYER_MORPHOGENESIS | 22 | 3.9 | 1.05 | 23.95 | 50.98 | 39 | 0.00707 | 0.00190 | 0.00181 | RSPO3 | 0.75 | 0.37 | ADM BMP5 BMP7 CCN1 DNAJB6 FGFR2 FZD5 GCM1 GJB5 GRB2 GRHL2 IL10 LEF1 NCOA1 RSPO3 SOCS3 SPINT1 SPINT2 ST14 TMED2 WNT7B ZFP36L1 |
| WHR | GOBP_CORONARY_VASCULATURE_MORPHOGENESIS | 18 | 3.71 | 1.16 | 17.55 | 36.77 | 28 | 0.01133 | 0.00354 | 0.00306 | VEGFA | 1.28 | 0.47 | ACE ARID2 FGF2 HAND2 HEY2 LRP2 NOTCH1 NRP1 PDGFRB SEC24B SETD2 SGCD SPRED1 TBX1 TBX5 TGFBR1 TGFBR3 VEGFA |
| WHR | GOBP_MOTOR_NEURON_MIGRATION | 6 | 3.42 | 1.48 | 42.17 | 29.08 | 8 | 0.00786 | 0.00341 | 0.00230 | VEGFA | 0.1 | 0.12 | DAB1 LHX1 NRP1 OLIG3 RELN VEGFA |
| WHR | GOBP_COMMISSURAL_NEURON_AXON_GUIDANCE | 11 | 3.09 | 1.2 | 32.37 | 31.18 | 15 | 0.00881 | 0.00341 | 0.00285 | VEGFA | 0.43 | 0.21 | DAG1 EPHB2 FZD3 GDNF NCAM1 NFIB NRP1 PTCH1 RYK SMO VEGFA |
| WHR | GOBP_THORAX_AND_ANTERIOR_ABDOMEN_DETERMINATION | 5 | 3.02 | 1.81 | 6.01 | 8 | 5 | 0.00091 | 0.00055 | 0.00030 | TIFAB | 2.98 | 1.79 | BASP1 DCANP1 NEUROG1 TIFAB WT1 |
| WHR | GOBP_REGULATION_OF_SALIVA_SECRETION | 7 | 2.71 | 1.62 | 0.71 | 8.36 | 8 | 0.00093 | 0.00055 | 0.00034 | OPRK1 | 2.5 | 1.69 | AQP1 DCANP1 FGF10 NEUROG1 OPRK1 TACR1 TIFAB |
| WHR | GOBP_RETINAL_GANGLION_CELL_AXON_GUIDANCE | 21 | 2.14 | 0.7 | 8.05 | 32.03 | 28 | 0.01071 | 0.00349 | 0.00500 | VEGFA | 0.63 | 0.25 | ALCAM BMPR1B EFNA5 EPHA7 EPHB1 EPHB2 EPHB3 ISL1 ISL2 NRCAM NRP1 POU4F2 POU4F3 PTPRM PTPRO ROBO2 RPL24 SEMA4F SLIT1 SLIT2 VEGFA |
| WHR | GOBP_ZYGOTIC_DETERMINATION_OF_ANTERIOR_POSTERIOR_AXIS_EMBRYO | 6 | 1.54 | 0.84 | 2.03 | 8.02 | 7 | 0.00110 | 0.00060 | 0.00071 | TIFAB | 1.52 | 0.83 | BASP1 DCANP1 NEUROG1 PCSK6 TIFAB WT1 |
| WHR | GOBP_VESTIBULOCOCHLEAR_NERVE_FORMATION | 5 | 1.39 | 0.93 | 7.57 | 8.78 | 5 | 0.00081 | 0.00055 | 0.00059 | TIFAB | 1.36 | 0.91 | ATP8B1 DCANP1 NEUROG1 PAX2 TIFAB |
| WHR | GOBP_VESTIBULOCOCHLEAR_NERVE_MORPHOGENESIS | 7 | 0.61 | 0.39 | 5.63 | 9.82 | 7 | 0.00088 | 0.00057 | 0.00144 | TIFAB | 0.6 | 0.39 | ATP8B1 DCANP1 NEUROG1 NRP1 NRP2 PAX2 TIFAB |
| PE | GOBP_Ductus arteriosus closure | 5 | 8.5 | 3.91 | 1.54 | 7.77 | 7 | 0.00328 | 0.00151 | 0.00039 | FOXF1 | 1.5 | 1.39 | FOXF1 HPGD MYOCD STRA6 TFAP2B |
| PE | GOBP_Regulation of retinal ganglion cell axon guidance | 5 | 2.66 | 1.48 | 1.45 | 5.72 | 5 | 0.00279 | 0.00155 | 0.00105 | VEGFA | 0.6 | 0.58 | NRP1 POU4F2 PTPRO SLIT2 VEGFA |
