# Supplementary material for: Genetic Correlation and Causal Inference Between Female Fat Distribution and Preeclampsia: An Integrative Genomic Study
Source: FASEB J. 2026 Jun 23;40(12):e72074. doi: 10.1096/fj.202601888R (PMC13288445; doi:10.1096/fj.202601888R)
Supplement: Supplementary file 10 — Table S10: GSA‐MIXeR gene‐level heritability estimates for WHR. h2‐ an estimate of gene's heritability from the full GSA‐MiXeR model; se_h2: standard error of h2; Other columns have been clarified in previous tables. [file FSB2-40-e72074-s009.docx]

| **Supplementary Table S10** | | |  |  |  |  |  |  |  |  |
| --- | --- | --- | --- | --- | --- | --- | --- | --- | --- | --- |
| ***GSA-MIXeR gene-level heritability estimates for WHR.*** *h2- an estimate of gene's heritability from the full GSA-MiXeR model; se_h2: standard error of h2; Other columns have been clarified in previous tables.* | | | | | | | | | | |
| **GENE** | **enrich** | **se_enrich** | **MIXER_AIC** | **loglike_diff** | **loglike_df** | **h2** | **se_h2** | **h2_frac** | **se_h2_frac** | **h2_base_frac** |
| RSPO3 | 55.62 | 17.39 | 97.65 | 49.83 | 1 | 0.001657 | 0.000518 | 0.005781 | 0.001807 | 0.000104 |
| COBLL1 | 16.87 | 8.02 | 56.47 | 29.23 | 1 | 0.000977 | 0.000464 | 0.003410 | 0.001621 | 0.000202 |
| ZC3H11B | 83.99 | 24.18 | 55.80 | 28.90 | 1 | 0.000445 | 0.000128 | 0.001551 | 0.000447 | 0.000018 |
| VEGFA | 43.71 | 19.46 | 51.51 | 26.76 | 1 | 0.002194 | 0.000977 | 0.007654 | 0.003407 | 0.000175 |
| GORAB | 72.55 | 41.51 | 15.10 | 8.55 | 1 | 0.000413 | 0.000236 | 0.001442 | 0.000825 | 0.000020 |
| FOXP2 | 7.58 | 3.54 | 15.02 | 8.51 | 1 | 0.000528 | 0.000246 | 0.001841 | 0.000859 | 0.000243 |
| ADAMTS9 | 26.17 | 11.61 | 14.78 | 8.39 | 1 | 0.001276 | 0.000566 | 0.004453 | 0.001975 | 0.000170 |
| NKX2-6 | 16.50 | 10.89 | 14.24 | 9.12 | 2 | 0.000405 | 0.000267 | 0.001413 | 0.000932 | 0.000086 |
| CNTN5 | 7.19 | 2.85 | 12.86 | 7.43 | 1 | 0.000704 | 0.000279 | 0.002456 | 0.000973 | 0.000342 |
| FTO | 3.39 | 1.35 | 12.48 | 8.24 | 2 | 0.000611 | 0.000244 | 0.002131 | 0.000850 | 0.000628 |
| TBX15 | 15.44 | 12.01 | 11.43 | 6.72 | 1 | 0.000694 | 0.000540 | 0.002423 | 0.001885 | 0.000157 |
| FLRT1 | 9.15 | 4.62 | 10.62 | 7.31 | 2 | 0.000467 | 0.000236 | 0.001629 | 0.000823 | 0.000178 |
| EYA1 | 7.41 | 3.56 | 10.35 | 6.18 | 1 | 0.000517 | 0.000249 | 0.001805 | 0.000868 | 0.000244 |
| ITPR2 | 5.77 | 2.49 | 9.64 | 5.82 | 1 | 0.000616 | 0.000266 | 0.002150 | 0.000929 | 0.000372 |
| ETAA1 | 92.67 | 46.54 | 9.44 | 5.72 | 1 | 0.000352 | 0.000177 | 0.001228 | 0.000617 | 0.000013 |
| MEIS1 | 5.08 | 2.83 | 8.17 | 5.09 | 1 | 0.000662 | 0.000369 | 0.002311 | 0.001289 | 0.000455 |
| SMAP2 | 8.19 | 5.75 | 8.15 | 5.08 | 1 | 0.000247 | 0.000173 | 0.000862 | 0.000605 | 0.000105 |
| ARID5B | 2.87 | 1.29 | 7.98 | 4.99 | 1 | 0.000560 | 0.000252 | 0.001956 | 0.000879 | 0.000680 |
| EBF2 | 3.84 | 1.68 | 7.92 | 4.96 | 1 | 0.000508 | 0.000222 | 0.001772 | 0.000776 | 0.000462 |
| EYA2 | 5.20 | 2.80 | 7.24 | 4.62 | 1 | 0.000363 | 0.000195 | 0.001265 | 0.000682 | 0.000243 |
| SOBP | 7.03 | 4.18 | 6.54 | 4.27 | 1 | 0.000383 | 0.000228 | 0.001338 | 0.000796 | 0.000190 |
| AKAP6 | 2.68 | 1.26 | 6.54 | 4.27 | 1 | 0.000564 | 0.000265 | 0.001967 | 0.000924 | 0.000734 |
| MACROD1 | 6.02 | 3.00 | 6.51 | 7.26 | 4 | 0.000472 | 0.000235 | 0.001646 | 0.000820 | 0.000273 |
| MN1 | 8.09 | 4.96 | 6.41 | 4.20 | 1 | 0.000338 | 0.000207 | 0.001180 | 0.000723 | 0.000146 |
| TOMM40 | 26.34 | 18.40 | 6.38 | 7.19 | 4 | 0.000459 | 0.000321 | 0.001601 | 0.001119 | 0.000061 |
| SIRPG | 33.16 | 18.86 | 6.26 | 5.13 | 2 | 0.000332 | 0.000189 | 0.001160 | 0.000659 | 0.000035 |
| VEGFC | 29.78 | 19.22 | 6.01 | 4.00 | 1 | 0.000482 | 0.000311 | 0.001680 | 0.001084 | 0.000056 |
| CEBPA | 36.35 | 28.91 | 5.99 | 3.99 | 1 | 0.000449 | 0.000357 | 0.001565 | 0.001245 | 0.000043 |
| MC4R | 134.45 | 95.58 | 5.97 | 3.98 | 1 | 0.000068 | 0.000048 | 0.000237 | 0.000169 | 0.000002 |
| DNMT3A | 9.81 | 6.12 | 5.89 | 3.95 | 1 | 0.000427 | 0.000266 | 0.001488 | 0.000928 | 0.000152 |
| SGCZ | 5.32 | 2.65 | 5.71 | 3.86 | 1 | 0.000465 | 0.000232 | 0.001623 | 0.000809 | 0.000305 |
| LONRF1 | 49.39 | 36.81 | 5.65 | 3.82 | 1 | 0.000645 | 0.000481 | 0.002251 | 0.001677 | 0.000046 |
| PEMT | 19.32 | 14.45 | 5.64 | 5.82 | 3 | 0.000401 | 0.000300 | 0.001400 | 0.001047 | 0.000072 |
| PARD3 | 3.00 | 1.39 | 5.60 | 3.80 | 1 | 0.000362 | 0.000168 | 0.001263 | 0.000585 | 0.000421 |
| AHNAK | 15.49 | 9.99 | 5.25 | 5.63 | 3 | 0.000665 | 0.000429 | 0.002319 | 0.001495 | 0.000150 |
| NR5A2 | 3.14 | 1.57 | 5.04 | 3.52 | 1 | 0.000311 | 0.000156 | 0.001086 | 0.000544 | 0.000346 |
| BCKDHB | 10.93 | 6.28 | 4.98 | 3.49 | 1 | 0.000230 | 0.000132 | 0.000801 | 0.000460 | 0.000073 |
| DNER | 4.80 | 2.88 | 4.97 | 3.48 | 1 | 0.000367 | 0.000220 | 0.001280 | 0.000768 | 0.000267 |
| HTR1E | 40.08 | 26.02 | 4.86 | 3.43 | 1 | 0.000290 | 0.000188 | 0.001011 | 0.000656 | 0.000025 |
| LRIG1 | 8.06 | 8.21 | 4.84 | 4.42 | 2 | 0.000386 | 0.000393 | 0.001346 | 0.001372 | 0.000167 |
| C13orf42 | 16.04 | 8.75 | 4.83 | 3.42 | 1 | 0.000261 | 0.000142 | 0.000910 | 0.000496 | 0.000057 |
| DCP1B | 11.37 | 7.47 | 4.80 | 3.40 | 1 | 0.000322 | 0.000212 | 0.001125 | 0.000739 | 0.000099 |
| EYA4 | 5.38 | 3.05 | 4.79 | 3.40 | 1 | 0.000321 | 0.000182 | 0.001120 | 0.000635 | 0.000208 |
| RAF1 | 33.40 | 22.31 | 4.78 | 4.39 | 2 | 0.000716 | 0.000478 | 0.002498 | 0.001669 | 0.000075 |
| TMCC1 | 53.93 | 41.32 | 4.77 | 3.38 | 1 | 0.000644 | 0.000494 | 0.002247 | 0.001722 | 0.000042 |
| KCNJ12 | 10.22 | 9.04 | 4.59 | 3.29 | 1 | 0.000226 | 0.000200 | 0.000790 | 0.000698 | 0.000077 |
| RCN1 | 17.22 | 13.02 | 4.48 | 3.24 | 1 | 0.000265 | 0.000200 | 0.000924 | 0.000699 | 0.000054 |
| ZNF664-RFLNA | 14.94 | 8.94 | 4.45 | 7.22 | 5 | 0.000910 | 0.000545 | 0.003176 | 0.001901 | 0.000213 |
| TIFAB | 16.91 | 11.75 | 4.43 | 4.22 | 2 | 0.000222 | 0.000154 | 0.000775 | 0.000538 | 0.000046 |
| HOXC13 | 18.60 | 13.75 | 4.38 | 4.19 | 2 | 0.000332 | 0.000245 | 0.001159 | 0.000856 | 0.000062 |
| WNT2 | 9.30 | 7.79 | 4.37 | 3.19 | 1 | 0.000213 | 0.000178 | 0.000742 | 0.000621 | 0.000080 |
| PRMT6 | 54.96 | 52.80 | 4.37 | 3.18 | 1 | 0.000195 | 0.000187 | 0.000680 | 0.000654 | 0.000012 |
| SPHKAP | 16.10 | 10.88 | 4.36 | 3.18 | 1 | 0.000206 | 0.000139 | 0.000720 | 0.000487 | 0.000045 |
| NRXN3 | 2.27 | 1.09 | 4.36 | 3.18 | 1 | 0.000547 | 0.000263 | 0.001909 | 0.000919 | 0.000842 |
| SNX10 | 7.81 | 5.10 | 4.34 | 3.17 | 1 | 0.000397 | 0.000259 | 0.001384 | 0.000904 | 0.000177 |
| DIRAS2 | 67.76 | 53.87 | 4.31 | 3.15 | 1 | 0.000308 | 0.000245 | 0.001074 | 0.000854 | 0.000016 |
| EN1 | 31.48 | 16.71 | 4.28 | 3.14 | 1 | 0.000844 | 0.000448 | 0.002943 | 0.001562 | 0.000093 |
| KIRREL1 | 4.91 | 2.91 | 4.19 | 3.10 | 1 | 0.000354 | 0.000210 | 0.001235 | 0.000732 | 0.000252 |
| PCK1 | 10.94 | 6.95 | 4.15 | 3.07 | 1 | 0.000272 | 0.000173 | 0.000950 | 0.000603 | 0.000087 |
| AK4 | 13.63 | 12.49 | 3.94 | 2.97 | 1 | 0.000241 | 0.000221 | 0.000839 | 0.000769 | 0.000062 |
| SPOCK3 | 10.98 | 6.56 | 3.91 | 2.95 | 1 | 0.000285 | 0.000170 | 0.000995 | 0.000594 | 0.000091 |
| CRADD | 3.63 | 2.27 | 3.82 | 2.91 | 1 | 0.000262 | 0.000164 | 0.000913 | 0.000571 | 0.000251 |
| TAMM41 | 15.68 | 14.02 | 3.81 | 2.90 | 1 | 0.000246 | 0.000220 | 0.000857 | 0.000766 | 0.000055 |
| TRPS1 | 4.38 | 2.74 | 3.80 | 2.90 | 1 | 0.000344 | 0.000215 | 0.001200 | 0.000751 | 0.000274 |
| HEY2 | 30.29 | 20.85 | 3.76 | 3.88 | 2 | 0.000458 | 0.000315 | 0.001598 | 0.001100 | 0.000053 |
| DMXL2 | 7.88 | 6.52 | 3.75 | 2.87 | 1 | 0.000216 | 0.000179 | 0.000754 | 0.000624 | 0.000096 |
| RPS6KA5 | 8.03 | 9.66 | 3.71 | 3.86 | 2 | 0.000208 | 0.000250 | 0.000725 | 0.000872 | 0.000090 |
| SGCD | 3.98 | 2.48 | 3.45 | 2.73 | 1 | 0.000195 | 0.000121 | 0.000680 | 0.000424 | 0.000171 |
| TRPC4 | 7.26 | 4.62 | 3.45 | 2.72 | 1 | 0.000278 | 0.000177 | 0.000970 | 0.000618 | 0.000134 |
| MON1B | 36.67 | 21.57 | 3.37 | 3.69 | 2 | 0.000356 | 0.000209 | 0.001242 | 0.000730 | 0.000034 |
| DCANP1 | 17.16 | 11.97 | 3.34 | 3.67 | 2 | 0.000216 | 0.000151 | 0.000755 | 0.000527 | 0.000044 |
| TNFAIP8 | 5.27 | 3.52 | 3.33 | 3.67 | 2 | 0.000344 | 0.000229 | 0.001199 | 0.000800 | 0.000227 |
| ANO6 | 17.08 | 15.91 | 3.31 | 2.66 | 1 | 0.000307 | 0.000286 | 0.001072 | 0.000999 | 0.000063 |
| SSPN | 21.42 | 13.18 | 3.29 | 2.65 | 1 | 0.000479 | 0.000295 | 0.001670 | 0.001028 | 0.000078 |
| ENO4 | 22.59 | 15.17 | 3.28 | 4.64 | 3 | 0.000233 | 0.000157 | 0.000813 | 0.000546 | 0.000036 |
| CYP2S1 | 19.61 | 15.72 | 3.13 | 3.56 | 2 | 0.000229 | 0.000183 | 0.000799 | 0.000640 | 0.000041 |
| SLC2A2 | 15.54 | 12.54 | 3.13 | 2.56 | 1 | 0.000174 | 0.000141 | 0.000608 | 0.000491 | 0.000039 |
| LMX1B | 3.85 | 2.15 | 3.12 | 2.56 | 1 | 0.000318 | 0.000177 | 0.001110 | 0.000618 | 0.000288 |
| MYH14 | 10.58 | 6.53 | 3.11 | 3.55 | 2 | 0.000478 | 0.000295 | 0.001669 | 0.001030 | 0.000158 |
| KLF14 | 47.36 | 37.92 | 3.02 | 2.51 | 1 | 0.000353 | 0.000283 | 0.001232 | 0.000987 | 0.000026 |
| B3GNT2 | 26.43 | 23.54 | 2.96 | 2.48 | 1 | 0.000136 | 0.000121 | 0.000475 | 0.000423 | 0.000018 |
| ABCA1 | 4.33 | 2.10 | 2.95 | 3.48 | 2 | 0.000508 | 0.000246 | 0.001772 | 0.000859 | 0.000409 |
| TMEM92 | 12.96 | 9.85 | 2.84 | 2.42 | 1 | 0.000228 | 0.000173 | 0.000795 | 0.000604 | 0.000061 |
| WSCD2 | 6.27 | 4.51 | 2.83 | 2.42 | 1 | 0.000187 | 0.000135 | 0.000652 | 0.000470 | 0.000104 |
| PAEP | 113.64 | 77.11 | 2.82 | 3.41 | 2 | 0.000088 | 0.000059 | 0.000306 | 0.000207 | 0.000003 |
| NRG2 | 6.14 | 3.53 | 2.80 | 3.40 | 2 | 0.000254 | 0.000146 | 0.000886 | 0.000508 | 0.000144 |
| NBAS | 5.37 | 4.03 | 2.79 | 2.40 | 1 | 0.000183 | 0.000138 | 0.000640 | 0.000481 | 0.000119 |
| L3MBTL3 | 12.83 | 10.53 | 2.72 | 3.36 | 2 | 0.000289 | 0.000237 | 0.001009 | 0.000828 | 0.000079 |
| SH3RF3 | 3.29 | 2.02 | 2.70 | 2.35 | 1 | 0.000335 | 0.000206 | 0.001167 | 0.000718 | 0.000355 |
| PIK3CG | 14.89 | 10.01 | 2.69 | 2.34 | 1 | 0.000156 | 0.000105 | 0.000544 | 0.000366 | 0.000037 |
| RTCB | 29.16 | 21.78 | 2.63 | 3.31 | 2 | 0.000263 | 0.000197 | 0.000918 | 0.000686 | 0.000031 |
| SVEP1 | 5.76 | 4.00 | 2.60 | 2.30 | 1 | 0.000310 | 0.000215 | 0.001082 | 0.000752 | 0.000188 |
| PPARG | 9.68 | 7.16 | 2.59 | 2.30 | 1 | 0.000415 | 0.000307 | 0.001448 | 0.001071 | 0.000150 |
| TUSC1 | 7.22 | 5.70 | 2.58 | 2.29 | 1 | 0.000113 | 0.000089 | 0.000395 | 0.000311 | 0.000055 |
| BNC2 | 2.00 | 1.04 | 2.53 | 2.27 | 1 | 0.000391 | 0.000203 | 0.001363 | 0.000708 | 0.000680 |
| FBXL7 | 4.17 | 3.19 | 2.41 | 2.20 | 1 | 0.000191 | 0.000146 | 0.000667 | 0.000510 | 0.000160 |
| BACE2 | 6.45 | 4.29 | 2.40 | 2.20 | 1 | 0.000211 | 0.000140 | 0.000735 | 0.000489 | 0.000114 |
| ROBO4 | 6.06 | 5.15 | 2.34 | 3.17 | 2 | 0.000224 | 0.000191 | 0.000782 | 0.000666 | 0.000129 |
| MEX3C | 15.16 | 11.36 | 2.31 | 2.15 | 1 | 0.000118 | 0.000089 | 0.000413 | 0.000309 | 0.000027 |
| CDK20 | 41.18 | 34.54 | 2.29 | 2.14 | 1 | 0.000108 | 0.000090 | 0.000376 | 0.000315 | 0.000009 |
| HMGXB4 | 13.12 | 13.87 | 2.25 | 3.13 | 2 | 0.000145 | 0.000153 | 0.000506 | 0.000535 | 0.000039 |
| OSMR | 16.10 | 10.26 | 2.25 | 3.12 | 2 | 0.000212 | 0.000135 | 0.000740 | 0.000472 | 0.000046 |
| FAM13A | 4.31 | 2.68 | 2.20 | 3.10 | 2 | 0.000243 | 0.000151 | 0.000847 | 0.000526 | 0.000196 |
| ONECUT1 | 5.71 | 3.92 | 2.12 | 2.06 | 1 | 0.000179 | 0.000123 | 0.000626 | 0.000430 | 0.000110 |
| MED13L | 5.40 | 3.71 | 2.10 | 2.05 | 1 | 0.000249 | 0.000171 | 0.000867 | 0.000596 | 0.000161 |
| RBBP6 | 7.40 | 5.56 | 2.05 | 2.03 | 1 | 0.000144 | 0.000108 | 0.000502 | 0.000378 | 0.000068 |
| SUCNR1 | 41.14 | 36.00 | 2.04 | 2.02 | 1 | 0.000139 | 0.000122 | 0.000486 | 0.000425 | 0.000012 |
| GRIK2 | 3.00 | 1.44 | 1.94 | 1.97 | 1 | 0.000274 | 0.000131 | 0.000956 | 0.000457 | 0.000318 |
| DENND1A | 3.25 | 2.25 | 1.93 | 2.97 | 2 | 0.000363 | 0.000251 | 0.001268 | 0.000877 | 0.000390 |
| SYCE1L | 26.09 | 15.89 | 1.90 | 2.95 | 2 | 0.000361 | 0.000220 | 0.001258 | 0.000766 | 0.000048 |
| KCNH5 | 5.18 | 3.96 | 1.89 | 1.95 | 1 | 0.000164 | 0.000125 | 0.000572 | 0.000437 | 0.000110 |
| ANKRD12 | 9.83 | 7.57 | 1.79 | 2.89 | 2 | 0.000184 | 0.000142 | 0.000642 | 0.000495 | 0.000065 |
| ARRDC4 | 13.63 | 11.98 | 1.70 | 1.85 | 1 | 0.000256 | 0.000225 | 0.000893 | 0.000785 | 0.000065 |
| PDLIM3 | 12.28 | 8.81 | 1.70 | 1.85 | 1 | 0.000174 | 0.000124 | 0.000605 | 0.000434 | 0.000049 |
| CMIP | 2.05 | 1.13 | 1.68 | 1.84 | 1 | 0.000282 | 0.000155 | 0.000984 | 0.000541 | 0.000480 |
| EHHADH | 24.90 | 27.70 | 1.67 | 1.84 | 1 | 0.000101 | 0.000112 | 0.000353 | 0.000392 | 0.000014 |
| PRR16 | 7.26 | 4.49 | 1.63 | 1.81 | 1 | 0.000182 | 0.000112 | 0.000634 | 0.000392 | 0.000087 |
| ZNF503 | 6.30 | 4.43 | 1.63 | 1.81 | 1 | 0.000223 | 0.000157 | 0.000776 | 0.000546 | 0.000123 |
| DLEU7 | 17.42 | 15.49 | 1.62 | 1.81 | 1 | 0.000327 | 0.000291 | 0.001140 | 0.001014 | 0.000065 |
| APOH | 61.01 | 41.77 | 1.60 | 2.80 | 2 | 0.000140 | 0.000096 | 0.000490 | 0.000335 | 0.000008 |
| NEGR1 | 2.49 | 1.47 | 1.58 | 1.79 | 1 | 0.000203 | 0.000120 | 0.000709 | 0.000419 | 0.000284 |
| FAM220A | 40.63 | 36.52 | 1.57 | 1.79 | 1 | 0.000253 | 0.000227 | 0.000882 | 0.000792 | 0.000022 |
| ADAMTS1 | 3.99 | 3.28 | 1.56 | 1.78 | 1 | 0.000133 | 0.000109 | 0.000462 | 0.000380 | 0.000116 |
| RPIA | 22.25 | 17.88 | 1.53 | 1.77 | 1 | 0.000139 | 0.000112 | 0.000487 | 0.000391 | 0.000022 |
| TMEM200C | 11.01 | 8.56 | 1.51 | 1.76 | 1 | 0.000327 | 0.000255 | 0.001142 | 0.000888 | 0.000104 |
| ISM1 | 4.08 | 2.91 | 1.47 | 1.73 | 1 | 0.000261 | 0.000187 | 0.000912 | 0.000651 | 0.000224 |
| STAM | 10.71 | 9.77 | 1.45 | 1.72 | 1 | 0.000205 | 0.000187 | 0.000717 | 0.000654 | 0.000067 |
| ZNF91 | 61.48 | 61.98 | 1.43 | 1.71 | 1 | 0.000166 | 0.000167 | 0.000579 | 0.000583 | 0.000009 |
| CNTLN | 6.36 | 4.38 | 1.40 | 1.70 | 1 | 0.000189 | 0.000130 | 0.000659 | 0.000454 | 0.000104 |
| BARHL2 | 8.87 | 6.30 | 1.37 | 1.69 | 1 | 0.000196 | 0.000139 | 0.000683 | 0.000485 | 0.000077 |
| KCNJ15 | 5.27 | 4.01 | 1.37 | 1.68 | 1 | 0.000184 | 0.000140 | 0.000641 | 0.000488 | 0.000122 |
| BOD1 | 34.21 | 25.18 | 1.36 | 1.68 | 1 | 0.000225 | 0.000165 | 0.000784 | 0.000577 | 0.000023 |
| ASB13 | 8.95 | 5.74 | 1.36 | 2.68 | 2 | 0.000255 | 0.000163 | 0.000890 | 0.000570 | 0.000099 |
| BLID | 19.88 | 16.03 | 1.36 | 1.68 | 1 | 0.000126 | 0.000101 | 0.000439 | 0.000354 | 0.000022 |
| SH3BP4 | 5.72 | 4.46 | 1.35 | 1.68 | 1 | 0.000316 | 0.000246 | 0.001103 | 0.000859 | 0.000193 |
| ZMIZ1 | 1.66 | 0.87 | 1.30 | 1.65 | 1 | 0.000357 | 0.000186 | 0.001246 | 0.000650 | 0.000750 |
| ABCA13 | 4.92 | 3.14 | 1.29 | 1.64 | 1 | 0.000151 | 0.000096 | 0.000528 | 0.000337 | 0.000107 |
| NPR3 | 3.66 | 2.60 | 1.24 | 1.62 | 1 | 0.000121 | 0.000086 | 0.000424 | 0.000302 | 0.000116 |
| KDM4C | 2.32 | 1.41 | 1.23 | 1.61 | 1 | 0.000234 | 0.000142 | 0.000815 | 0.000496 | 0.000351 |
| SEMA3C | 7.71 | 5.67 | 1.20 | 1.60 | 1 | 0.000337 | 0.000248 | 0.001176 | 0.000865 | 0.000153 |
| ARHGAP12 | 11.00 | 11.13 | 1.18 | 1.59 | 1 | 0.000234 | 0.000237 | 0.000817 | 0.000827 | 0.000074 |
| RUNX1T1 | 3.81 | 2.97 | 1.18 | 1.59 | 1 | 0.000106 | 0.000083 | 0.000370 | 0.000289 | 0.000097 |
| LMBR1 | 9.70 | 6.73 | 1.16 | 2.58 | 2 | 0.000201 | 0.000139 | 0.000701 | 0.000487 | 0.000072 |
| DPYD | 2.79 | 1.67 | 1.15 | 1.58 | 1 | 0.000325 | 0.000195 | 0.001134 | 0.000679 | 0.000406 |
| MLLT3 | 4.39 | 2.90 | 1.10 | 1.55 | 1 | 0.000284 | 0.000188 | 0.000992 | 0.000655 | 0.000226 |
| GALNTL6 | 3.72 | 2.39 | 1.09 | 1.55 | 1 | 0.000221 | 0.000142 | 0.000772 | 0.000496 | 0.000207 |
| BHLHE40 | 4.23 | 3.78 | 1.09 | 1.55 | 1 | 0.000123 | 0.000110 | 0.000430 | 0.000384 | 0.000102 |
| MPC1 | 14.61 | 16.84 | 1.07 | 1.54 | 1 | 0.000126 | 0.000145 | 0.000440 | 0.000507 | 0.000030 |
| FCGR2C | 10.97 | 8.87 | 1.06 | 1.53 | 1 | 0.000159 | 0.000129 | 0.000555 | 0.000449 | 0.000051 |
| SIAH2 | 31.60 | 30.79 | 1.03 | 1.52 | 1 | 0.000235 | 0.000229 | 0.000821 | 0.000800 | 0.000026 |
| ENPP7 | 20.03 | 19.22 | 1.03 | 1.51 | 1 | 0.000485 | 0.000466 | 0.001694 | 0.001625 | 0.000085 |
| TRIB2 | 10.83 | 8.07 | 1.03 | 1.51 | 1 | 0.000158 | 0.000118 | 0.000552 | 0.000412 | 0.000051 |
| TYRP1 | 40.41 | 30.72 | 1.00 | 1.50 | 1 | 0.000129 | 0.000098 | 0.000450 | 0.000342 | 0.000011 |
| LGI2 | 6.51 | 4.38 | 0.96 | 2.48 | 2 | 0.000209 | 0.000141 | 0.000729 | 0.000490 | 0.000112 |
| NOG | 30.83 | 27.80 | 0.96 | 1.48 | 1 | 0.000165 | 0.000149 | 0.000576 | 0.000519 | 0.000019 |
| UBE2E2 | 4.29 | 3.35 | 0.95 | 1.47 | 1 | 0.000118 | 0.000092 | 0.000410 | 0.000320 | 0.000096 |
| UPP2 | 4.95 | 4.09 | 0.95 | 1.47 | 1 | 0.000197 | 0.000163 | 0.000687 | 0.000568 | 0.000139 |
| MLYCD | 12.44 | 10.97 | 0.94 | 1.47 | 1 | 0.000183 | 0.000161 | 0.000639 | 0.000563 | 0.000051 |
| IRX5 | 7.09 | 7.23 | 0.93 | 1.47 | 1 | 0.000156 | 0.000160 | 0.000546 | 0.000557 | 0.000077 |
| ANXA5 | 4.84 | 3.69 | 0.93 | 1.47 | 1 | 0.000122 | 0.000093 | 0.000426 | 0.000325 | 0.000088 |
| MSR1 | 17.61 | 15.34 | 0.93 | 1.46 | 1 | 0.000124 | 0.000108 | 0.000432 | 0.000376 | 0.000025 |
| PTCH1 | 3.29 | 2.49 | 0.92 | 1.46 | 1 | 0.000147 | 0.000111 | 0.000511 | 0.000387 | 0.000155 |
| KTN1 | 11.41 | 9.31 | 0.92 | 1.46 | 1 | 0.000189 | 0.000154 | 0.000660 | 0.000539 | 0.000058 |
| GRM8 | 2.96 | 1.87 | 0.92 | 1.46 | 1 | 0.000269 | 0.000170 | 0.000940 | 0.000592 | 0.000317 |
| SMARCE1 | 17.69 | 15.44 | 0.91 | 2.45 | 2 | 0.000137 | 0.000120 | 0.000479 | 0.000418 | 0.000027 |
| CENPW | 25.62 | 26.95 | 0.89 | 1.45 | 1 | 0.000212 | 0.000223 | 0.000739 | 0.000777 | 0.000029 |
| ITGA8 | 4.77 | 3.54 | 0.86 | 1.43 | 1 | 0.000140 | 0.000104 | 0.000489 | 0.000363 | 0.000103 |
| INSR | 3.34 | 2.37 | 0.85 | 1.42 | 1 | 0.000258 | 0.000183 | 0.000899 | 0.000637 | 0.000269 |
| FIG4 | 12.16 | 8.77 | 0.84 | 2.42 | 2 | 0.000183 | 0.000132 | 0.000637 | 0.000460 | 0.000052 |
| MYH7 | 7.74 | 5.89 | 0.83 | 2.42 | 2 | 0.000223 | 0.000170 | 0.000779 | 0.000592 | 0.000101 |
| SPSB1 | 4.75 | 3.34 | 0.83 | 1.42 | 1 | 0.000303 | 0.000213 | 0.001057 | 0.000744 | 0.000222 |
| GPSM2 | 20.82 | 14.14 | 0.83 | 3.41 | 3 | 0.000190 | 0.000129 | 0.000664 | 0.000451 | 0.000032 |
| SOX5 | 1.51 | 0.76 | 0.82 | 1.41 | 1 | 0.000476 | 0.000241 | 0.001661 | 0.000842 | 0.001103 |
| COL6A2 | 4.83 | 3.33 | 0.79 | 2.39 | 2 | 0.000240 | 0.000165 | 0.000837 | 0.000577 | 0.000173 |
| SREK1 | 46.51 | 47.57 | 0.77 | 1.39 | 1 | 0.000248 | 0.000253 | 0.000864 | 0.000883 | 0.000019 |
| STC2 | 10.15 | 9.02 | 0.77 | 1.39 | 1 | 0.000196 | 0.000174 | 0.000685 | 0.000608 | 0.000067 |
| CXCL13 | 25.88 | 23.55 | 0.74 | 1.37 | 1 | 0.000144 | 0.000131 | 0.000503 | 0.000458 | 0.000019 |
| MAP3K9 | 7.60 | 6.50 | 0.74 | 1.37 | 1 | 0.000185 | 0.000158 | 0.000644 | 0.000551 | 0.000085 |
| POFUT2 | 7.53 | 10.70 | 0.69 | 1.35 | 1 | 0.000111 | 0.000158 | 0.000388 | 0.000552 | 0.000052 |
| HSPH1 | 8.88 | 11.19 | 0.69 | 1.35 | 1 | 0.000111 | 0.000140 | 0.000388 | 0.000489 | 0.000044 |
| ETS1 | 0.07 | 0.18 | 0.69 | 1.35 | 1 | 0.000010 | 0.000025 | 0.000035 | 0.000087 | 0.000490 |
| TENM2 | 0.08 | 0.18 | 0.69 | 1.35 | 1 | 0.000030 | 0.000068 | 0.000103 | 0.000238 | 0.001331 |
| KCNE3 | 16.31 | 16.27 | 0.67 | 1.34 | 1 | 0.000144 | 0.000143 | 0.000501 | 0.000500 | 0.000031 |
| GABRB3 | 4.82 | 3.52 | 0.66 | 1.33 | 1 | 0.000145 | 0.000106 | 0.000507 | 0.000370 | 0.000105 |
| FAM107B | 2.28 | 1.33 | 0.64 | 1.32 | 1 | 0.000244 | 0.000142 | 0.000852 | 0.000497 | 0.000373 |
| KBTBD8 | 17.71 | 14.34 | 0.62 | 1.31 | 1 | 0.000127 | 0.000103 | 0.000444 | 0.000360 | 0.000025 |
| ADRB2 | 3.65 | 2.93 | 0.61 | 1.31 | 1 | 0.000128 | 0.000103 | 0.000448 | 0.000360 | 0.000123 |
| VWA5B1 | 4.02 | 2.95 | 0.60 | 1.30 | 1 | 0.000148 | 0.000108 | 0.000515 | 0.000377 | 0.000128 |
| CPO | 20.78 | 16.66 | 0.60 | 1.30 | 1 | 0.000180 | 0.000145 | 0.000629 | 0.000504 | 0.000030 |
| BICD2 | 8.88 | 8.09 | 0.57 | 1.28 | 1 | 0.000092 | 0.000083 | 0.000319 | 0.000291 | 0.000036 |
| KHDRBS3 | 8.71 | 7.99 | 0.56 | 1.28 | 1 | 0.000130 | 0.000120 | 0.000455 | 0.000417 | 0.000052 |
| CADPS | 2.06 | 1.21 | 0.56 | 1.28 | 1 | 0.000280 | 0.000164 | 0.000976 | 0.000572 | 0.000474 |
| CIDEA | 6.06 | 4.84 | 0.54 | 1.27 | 1 | 0.000141 | 0.000113 | 0.000492 | 0.000393 | 0.000081 |
| NEDD9 | 0.07 | 0.17 | 0.54 | 1.27 | 1 | 0.000011 | 0.000026 | 0.000039 | 0.000091 | 0.000542 |
| DUSP10 | 5.71 | 4.60 | 0.53 | 1.27 | 1 | 0.000200 | 0.000161 | 0.000696 | 0.000561 | 0.000122 |
| SANBR | 35.97 | 24.49 | 0.52 | 3.26 | 3 | 0.000251 | 0.000171 | 0.000875 | 0.000596 | 0.000024 |
| KCNT2 | 8.21 | 7.74 | 0.52 | 1.26 | 1 | 0.000138 | 0.000130 | 0.000481 | 0.000453 | 0.000059 |
| VASH2 | 10.13 | 7.40 | 0.52 | 2.26 | 2 | 0.000206 | 0.000151 | 0.000720 | 0.000526 | 0.000071 |
| RCOR1 | 6.06 | 4.89 | 0.51 | 1.26 | 1 | 0.000108 | 0.000087 | 0.000377 | 0.000304 | 0.000062 |
| KIFC3 | 14.17 | 14.43 | 0.50 | 2.25 | 2 | 0.000277 | 0.000282 | 0.000966 | 0.000984 | 0.000068 |
| CYP2A13 | 74.43 | 66.92 | 0.49 | 2.24 | 2 | 0.000179 | 0.000161 | 0.000626 | 0.000563 | 0.000008 |
| GPR83 | 28.65 | 22.66 | 0.48 | 2.24 | 2 | 0.000167 | 0.000132 | 0.000582 | 0.000460 | 0.000020 |
| ADIPOQ | 12.69 | 10.41 | 0.46 | 1.23 | 1 | 0.000130 | 0.000107 | 0.000454 | 0.000373 | 0.000036 |
| CADM2 | 1.98 | 1.14 | 0.45 | 1.23 | 1 | 0.000203 | 0.000116 | 0.000707 | 0.000406 | 0.000357 |
| KRT7 | 5.27 | 4.03 | 0.44 | 1.22 | 1 | 0.000192 | 0.000147 | 0.000670 | 0.000513 | 0.000127 |
| NPFFR2 | 42.56 | 40.97 | 0.44 | 1.22 | 1 | 0.000082 | 0.000079 | 0.000287 | 0.000276 | 0.000007 |
| MRLN | 28.29 | 30.45 | 0.42 | 1.21 | 1 | 0.000082 | 0.000089 | 0.000288 | 0.000310 | 0.000010 |
| MTMR3 | 4.88 | 3.47 | 0.41 | 1.20 | 1 | 0.000096 | 0.000068 | 0.000333 | 0.000237 | 0.000068 |
| AQP4 | 10.48 | 8.83 | 0.40 | 1.20 | 1 | 0.000157 | 0.000133 | 0.000549 | 0.000463 | 0.000052 |
| IRS2 | 6.75 | 5.11 | 0.39 | 1.19 | 1 | 0.000178 | 0.000135 | 0.000620 | 0.000470 | 0.000092 |
| LSM4 | 12.00 | 14.35 | 0.39 | 2.19 | 2 | 0.000164 | 0.000197 | 0.000573 | 0.000686 | 0.000048 |
| LRFN2 | 2.63 | 1.85 | 0.37 | 1.19 | 1 | 0.000141 | 0.000099 | 0.000494 | 0.000346 | 0.000188 |
| CCK | 9.55 | 8.91 | 0.37 | 1.19 | 1 | 0.000077 | 0.000072 | 0.000269 | 0.000251 | 0.000028 |
| ACE | 5.13 | 5.36 | 0.37 | 1.18 | 1 | 0.000094 | 0.000098 | 0.000327 | 0.000342 | 0.000064 |
| KCNMA1 | 0.08 | 0.17 | 0.35 | 1.18 | 1 | 0.000020 | 0.000046 | 0.000070 | 0.000160 | 0.000919 |
| GRIN3A | 5.51 | 4.88 | 0.35 | 3.18 | 3 | 0.000232 | 0.000205 | 0.000809 | 0.000717 | 0.000147 |
| CNR1 | 5.21 | 4.19 | 0.35 | 1.17 | 1 | 0.000131 | 0.000105 | 0.000456 | 0.000367 | 0.000087 |
| MEGF9 | 4.22 | 4.05 | 0.35 | 1.17 | 1 | 0.000078 | 0.000074 | 0.000271 | 0.000260 | 0.000064 |
| TSEN15 | 21.80 | 17.29 | 0.34 | 2.17 | 2 | 0.000113 | 0.000090 | 0.000394 | 0.000313 | 0.000018 |
| APOB | 7.62 | 5.45 | 0.34 | 1.17 | 1 | 0.000113 | 0.000081 | 0.000396 | 0.000283 | 0.000052 |
| VLDLR | 3.49 | 2.74 | 0.34 | 1.17 | 1 | 0.000221 | 0.000174 | 0.000772 | 0.000607 | 0.000221 |
| H6PD | 4.09 | 3.49 | 0.33 | 1.16 | 1 | 0.000140 | 0.000119 | 0.000488 | 0.000416 | 0.000119 |
| SOCS6 | 7.37 | 6.68 | 0.32 | 1.16 | 1 | 0.000148 | 0.000134 | 0.000517 | 0.000468 | 0.000070 |
| NPY | 10.56 | 7.62 | 0.30 | 1.15 | 1 | 0.000122 | 0.000088 | 0.000427 | 0.000308 | 0.000040 |
| CBLN2 | 15.68 | 17.61 | 0.28 | 1.14 | 1 | 0.000123 | 0.000138 | 0.000427 | 0.000480 | 0.000027 |
| CYP4F22 | 7.61 | 6.05 | 0.28 | 1.14 | 1 | 0.000112 | 0.000089 | 0.000392 | 0.000312 | 0.000052 |
| LIN9 | 14.58 | 13.27 | 0.28 | 2.14 | 2 | 0.000186 | 0.000170 | 0.000650 | 0.000592 | 0.000045 |
| ANXA10 | 10.81 | 11.20 | 0.27 | 1.14 | 1 | 0.000131 | 0.000135 | 0.000456 | 0.000472 | 0.000042 |
| RAB38 | 8.25 | 7.85 | 0.27 | 1.13 | 1 | 0.000113 | 0.000107 | 0.000394 | 0.000375 | 0.000048 |
| KPNA2 | 28.57 | 23.97 | 0.26 | 1.13 | 1 | 0.000136 | 0.000114 | 0.000475 | 0.000398 | 0.000017 |
| GLI2 | 2.63 | 1.90 | 0.26 | 1.13 | 1 | 0.000232 | 0.000168 | 0.000810 | 0.000585 | 0.000308 |
| ACVR1C | 15.22 | 14.39 | 0.25 | 1.13 | 1 | 0.000142 | 0.000134 | 0.000496 | 0.000468 | 0.000033 |
| WNT5A | 7.26 | 6.31 | 0.24 | 1.12 | 1 | 0.000209 | 0.000182 | 0.000729 | 0.000634 | 0.000100 |
| SH3TC1 | 4.98 | 4.23 | 0.24 | 1.12 | 1 | 0.000180 | 0.000153 | 0.000629 | 0.000534 | 0.000126 |
| HDAC4 | 1.95 | 1.30 | 0.23 | 1.12 | 1 | 0.000246 | 0.000164 | 0.000860 | 0.000572 | 0.000441 |
| ABO | 4.22 | 3.56 | 0.23 | 1.11 | 1 | 0.000103 | 0.000087 | 0.000358 | 0.000302 | 0.000085 |
| VSX2 | 4.87 | 5.54 | 0.23 | 1.11 | 1 | 0.000077 | 0.000087 | 0.000268 | 0.000305 | 0.000055 |
| RXFP2 | 6.08 | 5.51 | 0.22 | 1.11 | 1 | 0.000113 | 0.000102 | 0.000393 | 0.000357 | 0.000065 |
| SLITRK1 | 31.02 | 28.71 | 0.21 | 1.11 | 1 | 0.000157 | 0.000146 | 0.000549 | 0.000508 | 0.000018 |
| SDK2 | 2.16 | 1.39 | 0.21 | 1.10 | 1 | 0.000250 | 0.000160 | 0.000872 | 0.000560 | 0.000403 |
| RBPJ | 3.35 | 2.46 | 0.20 | 1.10 | 1 | 0.000193 | 0.000141 | 0.000673 | 0.000493 | 0.000201 |
| GARIN4 | 30.94 | 29.10 | 0.19 | 2.10 | 2 | 0.000195 | 0.000183 | 0.000680 | 0.000640 | 0.000022 |
| PCP4L1 | 12.46 | 9.27 | 0.19 | 2.10 | 2 | 0.000145 | 0.000108 | 0.000506 | 0.000377 | 0.000041 |
| FGF1 | 5.47 | 4.75 | 0.19 | 1.10 | 1 | 0.000225 | 0.000195 | 0.000784 | 0.000681 | 0.000143 |
| NOL11 | 7.97 | 7.72 | 0.19 | 1.09 | 1 | 0.000080 | 0.000077 | 0.000278 | 0.000269 | 0.000035 |
| GSTA3 | 70.57 | 87.89 | 0.18 | 1.09 | 1 | 0.000099 | 0.000123 | 0.000346 | 0.000430 | 0.000005 |
| LITAF | 4.71 | 3.78 | 0.17 | 1.09 | 1 | 0.000209 | 0.000167 | 0.000728 | 0.000584 | 0.000154 |
| TEAD1 | 2.89 | 2.07 | 0.17 | 1.09 | 1 | 0.000201 | 0.000144 | 0.000702 | 0.000502 | 0.000243 |
| PTGFR | 19.98 | 18.46 | 0.13 | 1.06 | 1 | 0.000129 | 0.000119 | 0.000450 | 0.000416 | 0.000023 |
| ARL14EPL | 22.09 | 19.66 | 0.12 | 2.06 | 2 | 0.000132 | 0.000117 | 0.000460 | 0.000409 | 0.000021 |
| ETV6 | 0.07 | 0.18 | 0.12 | 1.06 | 1 | 0.000009 | 0.000022 | 0.000031 | 0.000077 | 0.000427 |
| TSPAN9 | 1.66 | 1.08 | 0.11 | 1.06 | 1 | 0.000283 | 0.000183 | 0.000987 | 0.000640 | 0.000595 |
| RTL1 | 6.83 | 5.49 | 0.11 | 1.05 | 1 | 0.000163 | 0.000131 | 0.000569 | 0.000457 | 0.000083 |
| AKAIN1 | 8.69 | 7.35 | 0.11 | 1.05 | 1 | 0.000167 | 0.000142 | 0.000584 | 0.000494 | 0.000067 |
| TPD52L1 | 7.61 | 5.92 | 0.09 | 2.04 | 2 | 0.000190 | 0.000147 | 0.000661 | 0.000514 | 0.000087 |
| C9orf152 | 10.05 | 9.60 | 0.06 | 1.03 | 1 | 0.000106 | 0.000101 | 0.000368 | 0.000352 | 0.000037 |
| RNASE10 | 29.90 | 27.66 | 0.05 | 1.03 | 1 | 0.000170 | 0.000157 | 0.000594 | 0.000550 | 0.000020 |
| ENOX1 | 1.90 | 1.17 | 0.03 | 1.02 | 1 | 0.000235 | 0.000144 | 0.000820 | 0.000504 | 0.000431 |
| PRSS35 | 23.37 | 22.25 | 0.03 | 1.02 | 1 | 0.000104 | 0.000099 | 0.000362 | 0.000344 | 0.000015 |
| OR10G3 | 9.55 | 8.41 | 0.03 | 1.02 | 1 | 0.000120 | 0.000106 | 0.000418 | 0.000368 | 0.000044 |
| RCSD1 | 5.84 | 3.99 | 0.02 | 2.01 | 2 | 0.000243 | 0.000166 | 0.000847 | 0.000579 | 0.000145 |
| CALML3 | 12.21 | 12.03 | 0.01 | 1.00 | 1 | 0.000143 | 0.000141 | 0.000499 | 0.000492 | 0.000041 |
| SCN7A | 10.37 | 10.64 | 0.00 | 1.00 | 1 | 0.000102 | 0.000104 | 0.000355 | 0.000363 | 0.000034 |
