# Supplementary material for: Genetic Correlation and Causal Inference Between Female Fat Distribution and Preeclampsia: An Integrative Genomic Study
Source: FASEB J. 2026 Jun 23;40(12):e72074. doi: 10.1096/fj.202601888R (PMC13288445; doi:10.1096/fj.202601888R)
Supplement: Supplementary file 11 — Table S11: GSA‐MIXeR gene‐level heritability estimates for PE. The columns are as follows: GENE: gene name; h2: an estimate of gene's heritability from the full GSA‐MiXeR model; se_h2: standard error of h2; Other columns have been clarified in previous tables. [file FSB2-40-e72074-s013.docx]

| **Supplementary Table S11** | | |  |  |  |  |  |  |  |  |
| --- | --- | --- | --- | --- | --- | --- | --- | --- | --- | --- |
| ***GSA-MIXeR gene-level heritability estimates for PE.*** *The columns are as follows: GENE: gene name; h2: an estimate of gene's heritability from the full GSA-MiXeR model; se_h2: standard error of h2; Other columns have been clarified in previous tables.* | | | | | | | | | | |
| **GENE** | **enrich** | **se_enrich** | **MIXER_AIC** | **loglike_diff** | **loglike_df** | **h2** | **se_h2** | **h2_frac** | **se_h2_frac** | **h2_base_frac** |
| FGF5 | 41.49 | 20.86 | 23.32 | 12.66 | 1 | 1.14E-04 | 5.70E-05 | 1.43E-03 | 7.20E-04 | 3.50E-05 |
| ZNF831 | 16.12 | 8.53 | 19.89 | 10.95 | 1 | 1.77E-04 | 9.40E-05 | 2.22E-03 | 1.17E-03 | 1.37E-04 |
| NPR3 | 29.16 | 14.48 | 16.58 | 9.29 | 1 | 2.17E-04 | 1.08E-04 | 2.72E-03 | 1.35E-03 | 9.30E-05 |
| MECOM | 4.15 | 1.6 | 11.65 | 6.82 | 1 | 1.87E-04 | 7.20E-05 | 2.34E-03 | 9.02E-04 | 5.63E-04 |
| PLCE1 | 19.32 | 9.56 | 11.59 | 7.8 | 2 | 2.41E-04 | 1.19E-04 | 3.02E-03 | 1.49E-03 | 1.56E-04 |
| FOXF1 | 37.69 | 18.65 | 10.14 | 7.07 | 2 | 2.24E-04 | 1.11E-04 | 2.81E-03 | 1.39E-03 | 7.50E-05 |
| ODAD3 | 24.03 | 17.74 | 8.7 | 8.35 | 4 | 9.00E-05 | 6.70E-05 | 1.13E-03 | 8.37E-04 | 4.70E-05 |
| VEGFA | 13.67 | 8.81 | 8.51 | 5.26 | 1 | 1.81E-04 | 1.16E-04 | 2.26E-03 | 1.46E-03 | 1.66E-04 |
| ADRB1 | 40.78 | 27.82 | 8.17 | 5.08 | 1 | 9.30E-05 | 6.30E-05 | 1.16E-03 | 7.92E-04 | 2.80E-05 |
| CTDP1 | 11.75 | 7.57 | 7.98 | 4.99 | 1 | 9.80E-05 | 6.30E-05 | 1.23E-03 | 7.95E-04 | 1.05E-04 |
| TSHZ3 | 13.98 | 8.05 | 7.26 | 4.63 | 1 | 1.70E-04 | 9.80E-05 | 2.13E-03 | 1.23E-03 | 1.53E-04 |
| FRMD1 | 24.54 | 14.76 | 7.22 | 5.61 | 2 | 9.30E-05 | 5.60E-05 | 1.16E-03 | 6.98E-04 | 4.70E-05 |
| TBXAS1 | 18.03 | 8.67 | 7.01 | 6.5 | 3 | 2.70E-04 | 1.30E-04 | 3.39E-03 | 1.63E-03 | 1.88E-04 |
| RNF180 | 37.68 | 26.26 | 6.38 | 4.19 | 1 | 7.30E-05 | 5.10E-05 | 9.09E-04 | 6.34E-04 | 2.40E-05 |
| E2F7 | 17.04 | 9.74 | 6.33 | 4.17 | 1 | 1.10E-04 | 6.30E-05 | 1.37E-03 | 7.85E-04 | 8.10E-05 |
| MFGE8 | 35.42 | 20.21 | 5.78 | 4.89 | 2 | 1.12E-04 | 6.40E-05 | 1.40E-03 | 8.01E-04 | 4.00E-05 |
| FURIN | 7.73 | 5.32 | 5.65 | 5.82 | 3 | 1.18E-04 | 8.10E-05 | 1.48E-03 | 1.02E-03 | 1.92E-04 |
| GMPR | 32.1 | 24.71 | 5.57 | 4.79 | 2 | 1.21E-04 | 9.30E-05 | 1.51E-03 | 1.17E-03 | 4.70E-05 |
| RGL3 | 25.04 | 19.11 | 5.55 | 7.77 | 5 | 8.40E-05 | 6.40E-05 | 1.05E-03 | 8.01E-04 | 4.20E-05 |
| ADAMTS18 | 6.54 | 3.9 | 5.54 | 3.77 | 1 | 9.10E-05 | 5.40E-05 | 1.15E-03 | 6.82E-04 | 1.75E-04 |
| RYK | 36.53 | 27.93 | 5.51 | 3.75 | 1 | 9.80E-05 | 7.50E-05 | 1.23E-03 | 9.41E-04 | 3.40E-05 |
| TNFRSF1B | 17.36 | 11.81 | 5.48 | 3.74 | 1 | 2.03E-04 | 1.38E-04 | 2.55E-03 | 1.73E-03 | 1.47E-04 |
| WT1 | 9.54 | 5.51 | 5.3 | 3.65 | 1 | 6.70E-05 | 3.90E-05 | 8.40E-04 | 4.85E-04 | 8.80E-05 |
| PPFIA2 | 13.18 | 10.15 | 5.28 | 4.64 | 2 | 1.15E-04 | 8.90E-05 | 1.44E-03 | 1.11E-03 | 1.09E-04 |
| CRYZL2P-SEC16B | 5.76 | 3.3 | 5.22 | 4.61 | 2 | 1.13E-04 | 6.50E-05 | 1.42E-03 | 8.12E-04 | 2.46E-04 |
| THRB | 4.7 | 2.3 | 5.16 | 3.58 | 1 | 1.43E-04 | 7.00E-05 | 1.79E-03 | 8.78E-04 | 3.81E-04 |
| SSR3 | 28.55 | 20.97 | 5.14 | 4.57 | 2 | 1.21E-04 | 8.90E-05 | 1.52E-03 | 1.12E-03 | 5.30E-05 |
| VPS13D | 16.08 | 11.57 | 5.11 | 3.56 | 1 | 2.19E-04 | 1.57E-04 | 2.74E-03 | 1.97E-03 | 1.70E-04 |
| ODC1 | 19.59 | 13.3 | 4.97 | 4.48 | 2 | 1.27E-04 | 8.60E-05 | 1.59E-03 | 1.08E-03 | 8.10E-05 |
| SEC16B | 7.44 | 4.65 | 4.94 | 4.47 | 2 | 1.09E-04 | 6.80E-05 | 1.36E-03 | 8.51E-04 | 1.83E-04 |
| TNRC6C | 34.69 | 23.62 | 4.82 | 4.41 | 2 | 1.61E-04 | 1.10E-04 | 2.02E-03 | 1.38E-03 | 5.80E-05 |
| UNC5C | 5.28 | 2.71 | 4.8 | 4.4 | 2 | 1.73E-04 | 8.90E-05 | 2.17E-03 | 1.12E-03 | 4.11E-04 |
| SMOC2 | 13.44 | 7.43 | 4.68 | 3.34 | 1 | 1.17E-04 | 6.50E-05 | 1.47E-03 | 8.12E-04 | 1.09E-04 |
| NTSR1 | 18.83 | 11.74 | 4.55 | 3.27 | 1 | 1.05E-04 | 6.60E-05 | 1.32E-03 | 8.22E-04 | 7.00E-05 |
| MTHFR | 10.9 | 7.84 | 4.46 | 5.23 | 3 | 1.60E-04 | 1.15E-04 | 2.01E-03 | 1.45E-03 | 1.85E-04 |
| FMO2 | 11.82 | 9.75 | 4.41 | 3.21 | 1 | 5.50E-05 | 4.50E-05 | 6.83E-04 | 5.64E-04 | 5.80E-05 |
| CACNA1D | 8.16 | 4.65 | 4.41 | 4.2 | 2 | 1.63E-04 | 9.30E-05 | 2.05E-03 | 1.17E-03 | 2.51E-04 |
| CNTNAP4 | 5.19 | 3.31 | 4.17 | 3.08 | 1 | 7.30E-05 | 4.70E-05 | 9.20E-04 | 5.86E-04 | 1.77E-04 |
| SYT1 | 6.08 | 4.47 | 4.13 | 3.07 | 1 | 9.60E-05 | 7.10E-05 | 1.21E-03 | 8.89E-04 | 1.99E-04 |
| DAZL | 32.77 | 24.55 | 4.09 | 3.05 | 1 | 7.60E-05 | 5.70E-05 | 9.49E-04 | 7.11E-04 | 2.90E-05 |
| SLC44A3 | 10.28 | 5.79 | 4.01 | 4 | 2 | 1.11E-04 | 6.20E-05 | 1.39E-03 | 7.82E-04 | 1.35E-04 |
| CCDC71L | 18.56 | 13.19 | 3.95 | 2.98 | 1 | 7.20E-05 | 5.10E-05 | 9.05E-04 | 6.43E-04 | 4.90E-05 |
| ERC2 | 3.01 | 1.74 | 3.94 | 2.97 | 1 | 1.47E-04 | 8.50E-05 | 1.85E-03 | 1.07E-03 | 6.14E-04 |
| TPM4 | 8.09 | 4.96 | 3.93 | 3.96 | 2 | 1.18E-04 | 7.20E-05 | 1.48E-03 | 9.06E-04 | 1.83E-04 |
| SVEP1 | 8.41 | 4.95 | 3.85 | 2.92 | 1 | 1.11E-04 | 6.60E-05 | 1.40E-03 | 8.22E-04 | 1.66E-04 |
| ANO6 | 15.23 | 11.92 | 3.82 | 2.91 | 1 | 6.90E-05 | 5.40E-05 | 8.63E-04 | 6.76E-04 | 5.70E-05 |
| HMCN2 | 12.43 | 5.73 | 3.79 | 3.9 | 2 | 1.57E-04 | 7.20E-05 | 1.97E-03 | 9.08E-04 | 1.58E-04 |
| ATP11A | 5.78 | 3.8 | 3.76 | 2.88 | 1 | 7.90E-05 | 5.20E-05 | 9.88E-04 | 6.50E-04 | 1.71E-04 |
| TMEM171 | 34.33 | 25.44 | 3.75 | 2.87 | 1 | 7.80E-05 | 5.80E-05 | 9.83E-04 | 7.28E-04 | 2.90E-05 |
| GRB10 | 5.31 | 3.93 | 3.51 | 2.76 | 1 | 6.50E-05 | 4.80E-05 | 8.11E-04 | 6.01E-04 | 1.53E-04 |
| KLF6 | 3.38 | 2.05 | 3.51 | 2.75 | 1 | 7.60E-05 | 4.60E-05 | 9.48E-04 | 5.75E-04 | 2.80E-04 |
| THADA | 2.74 | 1.59 | 3.49 | 3.74 | 2 | 9.70E-05 | 5.60E-05 | 1.21E-03 | 7.06E-04 | 4.43E-04 |
| CNKSR3 | 4.39 | 2.72 | 3.49 | 2.74 | 1 | 8.70E-05 | 5.40E-05 | 1.09E-03 | 6.76E-04 | 2.48E-04 |
| SOX4 | 59.83 | 45.1 | 3.46 | 2.73 | 1 | 9.10E-05 | 6.80E-05 | 1.14E-03 | 8.56E-04 | 1.90E-05 |
| NAV3 | 6.68 | 4.09 | 3.4 | 2.7 | 1 | 1.08E-04 | 6.60E-05 | 1.35E-03 | 8.26E-04 | 2.02E-04 |
| TRPC6 | 19.16 | 12.53 | 3.38 | 2.69 | 1 | 9.90E-05 | 6.50E-05 | 1.24E-03 | 8.09E-04 | 6.50E-05 |
| TRIM36 | 36.48 | 27.01 | 3.34 | 2.67 | 1 | 8.00E-05 | 6.00E-05 | 1.01E-03 | 7.46E-04 | 2.80E-05 |
| F5 | 8.69 | 7.82 | 3.31 | 3.65 | 2 | 1.10E-04 | 9.90E-05 | 1.38E-03 | 1.24E-03 | 1.59E-04 |
| PEX2 | 33.63 | 27.12 | 3.3 | 2.65 | 1 | 6.40E-05 | 5.10E-05 | 7.99E-04 | 6.44E-04 | 2.40E-05 |
| RASGRP1 | 13.84 | 9.29 | 3.26 | 3.63 | 2 | 1.09E-04 | 7.30E-05 | 1.36E-03 | 9.16E-04 | 9.90E-05 |
| KIF5C | 17.41 | 12.74 | 3.13 | 3.56 | 2 | 6.30E-05 | 4.60E-05 | 7.85E-04 | 5.74E-04 | 4.50E-05 |
| COL4A1 | 3.05 | 1.58 | 3 | 3.5 | 2 | 1.39E-04 | 7.20E-05 | 1.74E-03 | 9.05E-04 | 5.71E-04 |
| GLCCI1 | 8.18 | 5.54 | 2.96 | 2.48 | 1 | 6.30E-05 | 4.30E-05 | 7.90E-04 | 5.36E-04 | 9.70E-05 |
| SERTAD2 | 14.59 | 9.85 | 2.86 | 2.43 | 1 | 8.70E-05 | 5.90E-05 | 1.09E-03 | 7.34E-04 | 7.50E-05 |
| INPP4B | 3.48 | 2.16 | 2.78 | 2.39 | 1 | 1.10E-04 | 6.80E-05 | 1.38E-03 | 8.57E-04 | 3.96E-04 |
| CREG1 | 9.2 | 7.34 | 2.6 | 2.3 | 1 | 5.30E-05 | 4.20E-05 | 6.65E-04 | 5.30E-04 | 7.20E-05 |
| PTPRK | 5.5 | 3.67 | 2.54 | 2.27 | 1 | 1.05E-04 | 7.00E-05 | 1.32E-03 | 8.82E-04 | 2.40E-04 |
| PLA2G5 | 39.01 | 27.59 | 2.46 | 2.23 | 1 | 6.80E-05 | 4.80E-05 | 8.58E-04 | 6.07E-04 | 2.20E-05 |
| TLL1 | 16.95 | 11.24 | 2.43 | 2.22 | 1 | 8.50E-05 | 5.60E-05 | 1.06E-03 | 7.04E-04 | 6.30E-05 |
| ZBTB10 | 15.61 | 12.8 | 2.42 | 2.21 | 1 | 5.00E-05 | 4.10E-05 | 6.24E-04 | 5.12E-04 | 4.00E-05 |
| VSTM2L | 13.55 | 11.68 | 2.36 | 2.18 | 1 | 5.10E-05 | 4.40E-05 | 6.34E-04 | 5.46E-04 | 4.70E-05 |
| B4GALT5 | 4.69 | 3.6 | 2.33 | 2.16 | 1 | 6.00E-05 | 4.60E-05 | 7.50E-04 | 5.76E-04 | 1.60E-04 |
| METTL22 | 34.5 | 26.01 | 2.32 | 2.16 | 1 | 6.80E-05 | 5.10E-05 | 8.50E-04 | 6.40E-04 | 2.50E-05 |
| HEATR3 | 10.86 | 9.62 | 2.3 | 2.15 | 1 | 3.30E-05 | 2.90E-05 | 4.14E-04 | 3.67E-04 | 3.80E-05 |
| ETS1 | 1.64 | 1.11 | 2.25 | 2.13 | 1 | 7.80E-05 | 5.20E-05 | 9.75E-04 | 6.57E-04 | 5.93E-04 |
| ADGRL2 | 1.59 | 0.75 | 2.22 | 2.11 | 1 | 1.13E-04 | 5.30E-05 | 1.42E-03 | 6.67E-04 | 8.92E-04 |
| WDR7 | 11.01 | 6.99 | 2.22 | 3.11 | 2 | 1.43E-04 | 9.10E-05 | 1.79E-03 | 1.14E-03 | 1.63E-04 |
| PLA2G4A | 7.25 | 5.19 | 2.22 | 2.11 | 1 | 3.90E-05 | 2.80E-05 | 4.89E-04 | 3.50E-04 | 6.70E-05 |
| ARHGAP28 | 7.66 | 5.29 | 2.2 | 2.1 | 1 | 5.40E-05 | 3.70E-05 | 6.80E-04 | 4.69E-04 | 8.90E-05 |
| NFIX | 14.1 | 9.45 | 2.14 | 5.07 | 4 | 1.57E-04 | 1.05E-04 | 1.96E-03 | 1.32E-03 | 1.39E-04 |
| CDCP1 | 11.13 | 7.64 | 2.13 | 2.07 | 1 | 9.50E-05 | 6.50E-05 | 1.19E-03 | 8.15E-04 | 1.07E-04 |
| TRIB2 | 13.52 | 10.15 | 2.08 | 2.04 | 1 | 7.60E-05 | 5.70E-05 | 9.52E-04 | 7.14E-04 | 7.00E-05 |
| EML2 | 8.98 | 7.75 | 2.07 | 3.03 | 2 | 6.90E-05 | 6.00E-05 | 8.69E-04 | 7.50E-04 | 9.70E-05 |
| PIK3C3 | 18.24 | 14.08 | 2.04 | 2.02 | 1 | 5.10E-05 | 3.90E-05 | 6.36E-04 | 4.91E-04 | 3.50E-05 |
| AGTR1 | 9.01 | 6.93 | 2.01 | 2.01 | 1 | 7.30E-05 | 5.60E-05 | 9.20E-04 | 7.08E-04 | 1.02E-04 |
| ICA1 | 5.04 | 3.3 | 1.97 | 1.99 | 1 | 8.00E-05 | 5.20E-05 | 1.00E-03 | 6.55E-04 | 1.98E-04 |
| UQCRFS1 | 32.19 | 26.04 | 1.96 | 1.98 | 1 | 4.20E-05 | 3.40E-05 | 5.24E-04 | 4.24E-04 | 1.60E-05 |
| ZBTB46 | 8.75 | 4.93 | 1.93 | 4.97 | 4 | 1.13E-04 | 6.40E-05 | 1.42E-03 | 8.01E-04 | 1.63E-04 |
| TMTC2 | 7.06 | 4.82 | 1.9 | 1.95 | 1 | 1.21E-04 | 8.30E-05 | 1.52E-03 | 1.04E-03 | 2.15E-04 |
| ADA | 14.39 | 10.02 | 1.87 | 2.93 | 2 | 6.30E-05 | 4.40E-05 | 7.91E-04 | 5.51E-04 | 5.50E-05 |
| NKAIN2 | 4.3 | 2.86 | 1.84 | 1.92 | 1 | 1.20E-04 | 8.00E-05 | 1.51E-03 | 1.00E-03 | 3.50E-04 |
| PLCH1 | 8.55 | 5.32 | 1.83 | 2.91 | 2 | 5.50E-05 | 3.40E-05 | 6.86E-04 | 4.26E-04 | 8.00E-05 |
| PXYLP1 | 9.9 | 7.5 | 1.81 | 1.91 | 1 | 5.60E-05 | 4.20E-05 | 7.00E-04 | 5.31E-04 | 7.10E-05 |
| AUH | 24.6 | 17.78 | 1.73 | 1.87 | 1 | 8.50E-05 | 6.10E-05 | 1.07E-03 | 7.71E-04 | 4.30E-05 |
| VEGFC | 22.47 | 21.05 | 1.72 | 1.86 | 1 | 8.00E-05 | 7.50E-05 | 1.01E-03 | 9.41E-04 | 4.50E-05 |
| TET2 | 9.9 | 8.12 | 1.71 | 1.86 | 1 | 7.00E-05 | 5.70E-05 | 8.75E-04 | 7.18E-04 | 8.80E-05 |
| ACTA1 | 34.56 | 24.03 | 1.68 | 2.84 | 2 | 7.20E-05 | 5.00E-05 | 9.01E-04 | 6.26E-04 | 2.60E-05 |
| AQP9 | 30.52 | 22.85 | 1.67 | 1.84 | 1 | 9.20E-05 | 6.90E-05 | 1.16E-03 | 8.66E-04 | 3.80E-05 |
| SCN5A | 8.41 | 5.73 | 1.64 | 1.82 | 1 | 6.90E-05 | 4.70E-05 | 8.61E-04 | 5.87E-04 | 1.02E-04 |
| CEP43 | 21.1 | 22.45 | 1.64 | 1.82 | 1 | 6.70E-05 | 7.10E-05 | 8.34E-04 | 8.88E-04 | 4.00E-05 |
| GUCY2C | 45.65 | 39.43 | 1.63 | 1.81 | 1 | 4.50E-05 | 3.90E-05 | 5.70E-04 | 4.92E-04 | 1.20E-05 |
| ZBTB34 | 25.83 | 32.74 | 1.62 | 1.81 | 1 | 6.30E-05 | 8.00E-05 | 7.96E-04 | 1.01E-03 | 3.10E-05 |
| ELFN1 | 18.32 | 15.33 | 1.59 | 1.79 | 1 | 7.40E-05 | 6.20E-05 | 9.31E-04 | 7.78E-04 | 5.10E-05 |
| RPL39L | 50.39 | 51.84 | 1.57 | 1.78 | 1 | 1.90E-05 | 1.90E-05 | 2.34E-04 | 2.41E-04 | 5.00E-06 |
| RIPK4 | 6.07 | 5.2 | 1.56 | 1.78 | 1 | 5.30E-05 | 4.50E-05 | 6.64E-04 | 5.70E-04 | 1.10E-04 |
| CMTM8 | 12.47 | 9.24 | 1.56 | 1.78 | 1 | 8.50E-05 | 6.30E-05 | 1.06E-03 | 7.85E-04 | 8.50E-05 |
| ARHGAP42 | 4.74 | 3.28 | 1.5 | 1.75 | 1 | 9.00E-05 | 6.20E-05 | 1.13E-03 | 7.81E-04 | 2.38E-04 |
| ST7 | 12.04 | 9.15 | 1.49 | 1.74 | 1 | 8.70E-05 | 6.60E-05 | 1.09E-03 | 8.31E-04 | 9.10E-05 |
| CDH10 | 8.61 | 6.34 | 1.46 | 1.73 | 1 | 5.30E-05 | 3.90E-05 | 6.63E-04 | 4.88E-04 | 7.70E-05 |
| RPH3A | 19.85 | 12.17 | 1.46 | 2.73 | 2 | 1.06E-04 | 6.50E-05 | 1.32E-03 | 8.11E-04 | 6.70E-05 |
| DAO | 35.17 | 26.84 | 1.44 | 2.72 | 2 | 6.80E-05 | 5.20E-05 | 8.59E-04 | 6.55E-04 | 2.40E-05 |
| SOX11 | 19.44 | 16.06 | 1.41 | 1.71 | 1 | 3.90E-05 | 3.30E-05 | 4.93E-04 | 4.08E-04 | 2.50E-05 |
| FAM124A | 12.42 | 10.13 | 1.39 | 1.7 | 1 | 7.20E-05 | 5.90E-05 | 9.00E-04 | 7.34E-04 | 7.20E-05 |
| ZSWIM2 | 18.49 | 15.87 | 1.39 | 1.69 | 1 | 3.50E-05 | 3.00E-05 | 4.38E-04 | 3.76E-04 | 2.40E-05 |
| RAB27A | 5.97 | 3.94 | 1.38 | 2.69 | 2 | 7.50E-05 | 5.00E-05 | 9.43E-04 | 6.23E-04 | 1.58E-04 |
| OR10G3 | 16.59 | 12.6 | 1.36 | 1.68 | 1 | 4.30E-05 | 3.30E-05 | 5.37E-04 | 4.07E-04 | 3.20E-05 |
| TMEM72 | 11.57 | 9.14 | 1.35 | 1.68 | 1 | 3.20E-05 | 2.50E-05 | 4.03E-04 | 3.19E-04 | 3.50E-05 |
| CEP20 | 14.3 | 10.38 | 1.33 | 2.66 | 2 | 6.00E-05 | 4.30E-05 | 7.47E-04 | 5.43E-04 | 5.20E-05 |
| SCARB1 | 6.71 | 4.18 | 1.31 | 1.65 | 1 | 5.80E-05 | 3.60E-05 | 7.33E-04 | 4.57E-04 | 1.09E-04 |
| PGBD5 | 10.03 | 7.81 | 1.3 | 1.65 | 1 | 6.40E-05 | 5.00E-05 | 8.03E-04 | 6.25E-04 | 8.00E-05 |
| ADRA1B | 7.67 | 5.28 | 1.3 | 1.65 | 1 | 4.40E-05 | 3.00E-05 | 5.47E-04 | 3.77E-04 | 7.10E-05 |
| PLPP4 | 13.53 | 10.64 | 1.27 | 1.64 | 1 | 6.60E-05 | 5.20E-05 | 8.27E-04 | 6.50E-04 | 6.10E-05 |
| GPD2 | 8.01 | 7.34 | 1.27 | 1.63 | 1 | 5.50E-05 | 5.00E-05 | 6.88E-04 | 6.30E-04 | 8.60E-05 |
| SPATA9 | 17.15 | 12.32 | 1.26 | 3.63 | 3 | 1.02E-04 | 7.30E-05 | 1.28E-03 | 9.21E-04 | 7.50E-05 |
| ZCCHC2 | 8.48 | 7.47 | 1.15 | 1.57 | 1 | 6.30E-05 | 5.60E-05 | 7.94E-04 | 6.99E-04 | 9.40E-05 |
| PPP1R12A | 10.5 | 10.5 | 1.11 | 1.56 | 1 | 4.20E-05 | 4.20E-05 | 5.22E-04 | 5.22E-04 | 5.00E-05 |
| PIEZO2 | 2.29 | 1.5 | 1.11 | 1.55 | 1 | 7.40E-05 | 4.90E-05 | 9.33E-04 | 6.11E-04 | 4.07E-04 |
| CDH20 | 7.41 | 4.47 | 1.1 | 1.55 | 1 | 9.40E-05 | 5.70E-05 | 1.18E-03 | 7.09E-04 | 1.59E-04 |
| NTRK2 | 5.27 | 3.49 | 1.1 | 1.55 | 1 | 7.30E-05 | 4.80E-05 | 9.19E-04 | 6.08E-04 | 1.74E-04 |
| SIK1 | 56.9 | 46.59 | 1.08 | 1.54 | 1 | 5.43E-04 | 4.45E-04 | 6.81E-03 | 5.57E-03 | 1.20E-04 |
| TACSTD2 | 17.42 | 24.43 | 1.05 | 1.53 | 1 | 6.50E-05 | 9.20E-05 | 8.18E-04 | 1.15E-03 | 4.70E-05 |
| ARID5B | 1.56 | 1.08 | 1.04 | 1.52 | 1 | 9.60E-05 | 6.60E-05 | 1.20E-03 | 8.29E-04 | 7.67E-04 |
| KLF13 | 3.3 | 2.58 | 1.02 | 1.51 | 1 | 5.40E-05 | 4.20E-05 | 6.76E-04 | 5.28E-04 | 2.05E-04 |
| SHQ1 | 10.26 | 8.71 | 1 | 1.5 | 1 | 3.70E-05 | 3.10E-05 | 4.59E-04 | 3.90E-04 | 4.50E-05 |
| TRPS1 | 2.89 | 2.16 | 0.98 | 1.49 | 1 | 6.60E-05 | 4.90E-05 | 8.31E-04 | 6.20E-04 | 2.87E-04 |
| MYL10 | 12.41 | 10.63 | 0.97 | 1.49 | 1 | 3.60E-05 | 3.10E-05 | 4.55E-04 | 3.90E-04 | 3.70E-05 |
| EIF2D | 15.04 | 9.91 | 0.97 | 2.48 | 2 | 7.10E-05 | 4.70E-05 | 8.96E-04 | 5.91E-04 | 6.00E-05 |
| TSHZ2 | 3.26 | 2.09 | 0.92 | 1.46 | 1 | 1.16E-04 | 7.40E-05 | 1.45E-03 | 9.30E-04 | 4.46E-04 |
| NWD2 | 10.53 | 6.11 | 0.91 | 2.45 | 2 | 8.90E-05 | 5.20E-05 | 1.12E-03 | 6.49E-04 | 1.06E-04 |
| OTOGL | 5.93 | 4.83 | 0.84 | 1.42 | 1 | 5.30E-05 | 4.30E-05 | 6.59E-04 | 5.37E-04 | 1.11E-04 |
| IGLL5 | 25.52 | 19.13 | 0.83 | 2.42 | 2 | 4.60E-05 | 3.40E-05 | 5.73E-04 | 4.30E-04 | 2.20E-05 |
| ENPEP | 6.44 | 5.29 | 0.81 | 1.41 | 1 | 3.20E-05 | 2.60E-05 | 4.02E-04 | 3.30E-04 | 6.20E-05 |
| SLC45A3 | 8.49 | 9.13 | 0.81 | 1.4 | 1 | 7.20E-05 | 7.70E-05 | 9.01E-04 | 9.70E-04 | 1.06E-04 |
| ZNF528 | 50.93 | 39.07 | 0.79 | 3.4 | 3 | 4.40E-05 | 3.40E-05 | 5.49E-04 | 4.22E-04 | 1.10E-05 |
| LGALSL | 12.83 | 11.13 | 0.77 | 1.39 | 1 | 4.40E-05 | 3.80E-05 | 5.51E-04 | 4.78E-04 | 4.30E-05 |
| RPL34 | 18.96 | 15.87 | 0.76 | 1.38 | 1 | 5.30E-05 | 4.50E-05 | 6.70E-04 | 5.61E-04 | 3.50E-05 |
| RASSF10 | 16.72 | 13.27 | 0.74 | 1.37 | 1 | 4.20E-05 | 3.30E-05 | 5.20E-04 | 4.13E-04 | 3.10E-05 |
| SCAF8 | 12.47 | 10.89 | 0.73 | 1.36 | 1 | 5.30E-05 | 4.60E-05 | 6.65E-04 | 5.81E-04 | 5.30E-05 |
| CYP2B6 | 17.25 | 15.97 | 0.72 | 1.36 | 1 | 6.20E-05 | 5.70E-05 | 7.76E-04 | 7.18E-04 | 4.50E-05 |
| CDH5 | 8.2 | 6.03 | 0.66 | 1.33 | 1 | 5.60E-05 | 4.10E-05 | 7.06E-04 | 5.19E-04 | 8.60E-05 |
| USP31 | 4.44 | 3.78 | 0.64 | 1.32 | 1 | 3.40E-05 | 2.90E-05 | 4.26E-04 | 3.63E-04 | 9.60E-05 |
| MMAA | 14.54 | 14.78 | 0.61 | 1.31 | 1 | 3.50E-05 | 3.50E-05 | 4.34E-04 | 4.41E-04 | 3.00E-05 |
| CLEC3A | 20.32 | 22.19 | 0.61 | 1.31 | 1 | 3.90E-05 | 4.30E-05 | 4.95E-04 | 5.40E-04 | 2.40E-05 |
| STOML1 | 14.32 | 11.05 | 0.6 | 2.3 | 2 | 7.60E-05 | 5.90E-05 | 9.51E-04 | 7.34E-04 | 6.60E-05 |
| SEMA6D | 1.95 | 1.58 | 0.6 | 1.3 | 1 | 7.00E-05 | 5.70E-05 | 8.75E-04 | 7.09E-04 | 4.50E-04 |
| RBPMS | 9.04 | 6.71 | 0.59 | 2.29 | 2 | 9.80E-05 | 7.20E-05 | 1.22E-03 | 9.07E-04 | 1.35E-04 |
| MAP10 | 19.77 | 18.34 | 0.57 | 1.29 | 1 | 3.70E-05 | 3.50E-05 | 4.68E-04 | 4.34E-04 | 2.40E-05 |
| PCDH9 | 1.63 | 1.05 | 0.57 | 1.29 | 1 | 7.50E-05 | 4.90E-05 | 9.44E-04 | 6.11E-04 | 5.79E-04 |
| SCML4 | 2.95 | 2.44 | 0.53 | 1.27 | 1 | 5.60E-05 | 4.60E-05 | 7.00E-04 | 5.79E-04 | 2.37E-04 |
| PRTFDC1 | 8.77 | 8.54 | 0.51 | 1.25 | 1 | 6.00E-05 | 5.80E-05 | 7.53E-04 | 7.33E-04 | 8.60E-05 |
| SATB1 | 3.61 | 2.78 | 0.51 | 1.25 | 1 | 6.60E-05 | 5.10E-05 | 8.27E-04 | 6.37E-04 | 2.29E-04 |
| FEZF2 | 15.95 | 15.05 | 0.5 | 1.25 | 1 | 3.30E-05 | 3.10E-05 | 4.11E-04 | 3.88E-04 | 2.60E-05 |
| CMIP | 1.94 | 1.24 | 0.47 | 1.24 | 1 | 7.00E-05 | 4.50E-05 | 8.77E-04 | 5.62E-04 | 4.52E-04 |
| PML | 11.15 | 8.93 | 0.45 | 2.22 | 2 | 7.70E-05 | 6.20E-05 | 9.70E-04 | 7.77E-04 | 8.70E-05 |
| LSAMP | 2.74 | 1.81 | 0.43 | 1.22 | 1 | 8.40E-05 | 5.60E-05 | 1.06E-03 | 6.97E-04 | 3.85E-04 |
| SMAD2 | 6.8 | 5.87 | 0.41 | 1.2 | 1 | 5.60E-05 | 4.80E-05 | 7.04E-04 | 6.07E-04 | 1.03E-04 |
| SYT13 | 12.14 | 22.92 | 0.41 | 2.2 | 2 | 3.90E-05 | 7.40E-05 | 4.94E-04 | 9.33E-04 | 4.10E-05 |
| SLC26A3 | 19.6 | 14.97 | 0.39 | 2.19 | 2 | 5.90E-05 | 4.50E-05 | 7.37E-04 | 5.63E-04 | 3.80E-05 |
| CCND2 | 5.83 | 3.73 | 0.31 | 2.16 | 2 | 6.20E-05 | 4.00E-05 | 7.82E-04 | 5.00E-04 | 1.34E-04 |
| MYO5A | 10.24 | 10.11 | 0.31 | 3.16 | 3 | 1.00E-04 | 9.90E-05 | 1.26E-03 | 1.24E-03 | 1.23E-04 |
| PDZRN3 | 4.34 | 3.55 | 0.3 | 1.15 | 1 | 6.20E-05 | 5.10E-05 | 7.76E-04 | 6.34E-04 | 1.79E-04 |
| APBB1IP | 3.23 | 2.84 | 0.3 | 1.15 | 1 | 5.60E-05 | 4.90E-05 | 7.01E-04 | 6.16E-04 | 2.17E-04 |
| VIT | 8.36 | 7.04 | 0.28 | 1.14 | 1 | 7.50E-05 | 6.40E-05 | 9.46E-04 | 7.98E-04 | 1.13E-04 |
| DEPDC1 | 23.99 | 20.9 | 0.27 | 1.14 | 1 | 3.00E-05 | 2.60E-05 | 3.72E-04 | 3.24E-04 | 1.60E-05 |
| PARVA | 4.52 | 3.17 | 0.27 | 1.13 | 1 | 6.30E-05 | 4.40E-05 | 7.89E-04 | 5.54E-04 | 1.75E-04 |
| ZNF883 | 21.4 | 17.19 | 0.25 | 1.13 | 1 | 2.50E-05 | 2.00E-05 | 3.13E-04 | 2.51E-04 | 1.50E-05 |
| CASD1 | 15.37 | 15.56 | 0.25 | 1.12 | 1 | 6.80E-05 | 6.90E-05 | 8.53E-04 | 8.64E-04 | 5.60E-05 |
| RAD23B | 5.38 | 4.63 | 0.23 | 1.12 | 1 | 3.60E-05 | 3.10E-05 | 4.47E-04 | 3.85E-04 | 8.30E-05 |
| UGCG | 2.96 | 2.88 | 0.23 | 1.12 | 1 | 4.70E-05 | 4.60E-05 | 5.91E-04 | 5.77E-04 | 2.00E-04 |
| GABRB3 | 9.49 | 7.4 | 0.23 | 1.11 | 1 | 5.20E-05 | 4.00E-05 | 6.46E-04 | 5.04E-04 | 6.80E-05 |
| CALCOCO1 | 11.67 | 9.13 | 0.21 | 1.11 | 1 | 5.50E-05 | 4.30E-05 | 6.86E-04 | 5.37E-04 | 5.90E-05 |
| HTR1E | 39.51 | 36.15 | 0.19 | 1.1 | 1 | 5.80E-05 | 5.30E-05 | 7.30E-04 | 6.68E-04 | 1.80E-05 |
| JUND | 8.12 | 6.1 | 0.19 | 2.09 | 2 | 5.10E-05 | 3.80E-05 | 6.35E-04 | 4.77E-04 | 7.80E-05 |
| WSCD2 | 6.51 | 5.88 | 0.19 | 1.09 | 1 | 3.60E-05 | 3.30E-05 | 4.56E-04 | 4.12E-04 | 7.00E-05 |
| ITPR2 | 1.89 | 1.35 | 0.18 | 1.09 | 1 | 5.90E-05 | 4.20E-05 | 7.34E-04 | 5.24E-04 | 3.88E-04 |
| MARCKS | 9.06 | 7.74 | 0.18 | 1.09 | 1 | 3.40E-05 | 2.90E-05 | 4.29E-04 | 3.67E-04 | 4.70E-05 |
| CERS4 | 6.17 | 4.94 | 0.17 | 1.08 | 1 | 3.80E-05 | 3.00E-05 | 4.76E-04 | 3.81E-04 | 7.70E-05 |
| STK39 | 2.58 | 2.23 | 0.17 | 1.08 | 1 | 4.20E-05 | 3.70E-05 | 5.33E-04 | 4.61E-04 | 2.07E-04 |
| CRACD | 10.2 | 7.63 | 0.17 | 2.08 | 2 | 1.01E-04 | 7.60E-05 | 1.27E-03 | 9.49E-04 | 1.24E-04 |
| FIGN | 3.46 | 2.81 | 0.15 | 1.07 | 1 | 4.40E-05 | 3.60E-05 | 5.53E-04 | 4.49E-04 | 1.60E-04 |
| PREP | 5.31 | 4.6 | 0.14 | 1.07 | 1 | 3.60E-05 | 3.10E-05 | 4.48E-04 | 3.88E-04 | 8.40E-05 |
| EPC2 | 8.25 | 8.61 | 0.14 | 1.07 | 1 | 2.20E-05 | 2.30E-05 | 2.71E-04 | 2.83E-04 | 3.30E-05 |
| HMHB1 | 54.02 | 48.93 | 0.14 | 1.07 | 1 | 3.50E-05 | 3.20E-05 | 4.43E-04 | 4.01E-04 | 8.00E-06 |
| NUDT7 | 11.06 | 10.63 | 0.12 | 1.06 | 1 | 3.60E-05 | 3.40E-05 | 4.46E-04 | 4.29E-04 | 4.00E-05 |
| BHLHE41 | 11.31 | 13.11 | 0.08 | 1.04 | 1 | 4.60E-05 | 5.30E-05 | 5.78E-04 | 6.71E-04 | 5.10E-05 |
| PTPN2 | 3.91 | 3.91 | 0.08 | 1.04 | 1 | 4.10E-05 | 4.10E-05 | 5.18E-04 | 5.17E-04 | 1.32E-04 |
| CCNYL1 | 10.97 | 9.05 | 0.07 | 2.03 | 2 | 5.00E-05 | 4.10E-05 | 6.28E-04 | 5.18E-04 | 5.70E-05 |
| LUZP2 | 7.01 | 5.27 | 0.03 | 1.02 | 1 | 6.70E-05 | 5.00E-05 | 8.37E-04 | 6.29E-04 | 1.19E-04 |
| NABP1 | 46.55 | 49.22 | 0.03 | 1.02 | 1 | 3.50E-05 | 3.70E-05 | 4.37E-04 | 4.62E-04 | 9.00E-06 |
| VRK1 | 11.63 | 11.42 | 0.03 | 1.02 | 1 | 5.20E-05 | 5.10E-05 | 6.46E-04 | 6.35E-04 | 5.60E-05 |
| SLC38A11 | 6.39 | 5.92 | 0.03 | 1.02 | 1 | 3.00E-05 | 2.70E-05 | 3.71E-04 | 3.43E-04 | 5.80E-05 |
| FTO | 2.68 | 1.71 | 0.02 | 2.01 | 2 | 7.00E-05 | 4.50E-05 | 8.75E-04 | 5.61E-04 | 3.27E-04 |
| EDEM2 | 29.12 | 21.87 | 0.01 | 2 | 2 | 8.90E-05 | 6.70E-05 | 1.12E-03 | 8.43E-04 | 3.90E-05 |
| SPHKAP | 8.7 | 7.95 | 0 | 1 | 1 | 3.60E-05 | 3.30E-05 | 4.51E-04 | 4.13E-04 | 5.20E-05 |
| SOSTDC1 | 10.54 | 10.93 | 0 | 1 | 1 | 2.10E-05 | 2.20E-05 | 2.67E-04 | 2.77E-04 | 2.50E-05 |
