# Supplementary material for: Genetic Correlation and Causal Inference Between Female Fat Distribution and Preeclampsia: An Integrative Genomic Study
Source: FASEB J. 2026 Jun 23;40(12):e72074. doi: 10.1096/fj.202601888R (PMC13288445; doi:10.1096/fj.202601888R)
Supplement: Supplementary file 12 — Table S12: Significant TWAS analysis results. [file FSB2-40-e72074-s005.docx]

| **Supplementary Table S12** | | |  |  |  |  |
| --- | --- | --- | --- | --- | --- | --- |
| ***Significant TWAS analysis results.*** | | | | | | |
| Tissue | GENE | TWAS.Z.PP | TWAS.P.PP | MODEL | TWAS.Z.WHR | TWAS.P.WHR |
| Blood | TCF19 | 4.79502 | 0.00000163 | susie | 5.888 | 3.91E-09 |
| Ovary | CYP21A2 | 4.4263 | 0.00000959 | enet | 5.2715 | 0.000000135 |
