# Supplementary material for: Genetic Correlation and Causal Inference Between Female Fat Distribution and Preeclampsia: An Integrative Genomic Study
Source: FASEB J. 2026 Jun 23;40(12):e72074. doi: 10.1096/fj.202601888R (PMC13288445; doi:10.1096/fj.202601888R)
Supplement: Supplementary file 13 — Table S13: Results of MR analyses between WHR and PE. No. SNP: The number of genetic variants used as instrumental variables in the MR analysis; WM: Weighted median; IVW: Inverse variance weighted; Mean F stasistic: The average F statistic of all SNPs involved in MR analysis. Other columns have been clarified in previous tables. [file FSB2-40-e72074-s001.docx]

| **Supplementary Table S13** | | |  |  |  |  |  |
| --- | --- | --- | --- | --- | --- | --- | --- |
| ***Results of MR analyses between WHR and PE.*** *No.SNP: The number of genetic variants used as instrumental variables in the MR analysis; WM: Weighted median; IVW: Inverse variance weighted; Mean F stasistic: The average F statistic of all SNPs involved in MR analysis. Other columns have been clarified in previous tables.* | | | | | | | |
| **Exposure** | **Outcome** | **Method** | **No.SNP** | **BETA** | **SE** | ***P*** | ***Mean F statistic*** |
| **WHR** | **PE** | MR Egger | 240 | 0.082463 | 0.116699 | 0.480489 | 87.16 |
|  |  | WM | 240 | 0.21343 | 0.037468 | 0.376741 |  |
|  |  | IVW | 240 | 0.173065 | 0.047495 | 0.000269 |  |
|  |  | BWMR | 240 | 0.089506 | 0.07525 | 0.313786 |  |
|  |  | RAPS | 240 | 0.00305 | 0.139001 | 0.997895 |  |
| **PE** | **WHR** | Wald ratio | 1 | -0.00482 | 0.028144 | 0.863887 | 35.85 |
