# Supplementary material for: Genetic Correlation and Causal Inference Between Female Fat Distribution and Preeclampsia: An Integrative Genomic Study
Source: FASEB J. 2026 Jun 23;40(12):e72074. doi: 10.1096/fj.202601888R (PMC13288445; doi:10.1096/fj.202601888R)
Supplement: Supplementary file 14 — Table S14: Sensitivity analysis of MR analyses. Exposure: The exposure variable; Outcome: The outcome variable; Method: Mendelian Randomization method used for analysis; Q: Cochran's Q statistic, assessing heterogeneity among SNPs; Q_df: Degrees of freedom for the Q statistic; Q_P: P associated with the Q statistic, indicating the presence of heterogeneity. [file FSB2-40-e72074-s010.docx]

| **Supplementary Table S14** | |  |  |  |  |  |
| --- | --- | --- | --- | --- | --- | --- |
| ***Sensitivity analysis of MR analyses.*** *Exposure: The exposure variable; Outcome: The outcome variable; Method: Mendelian Randomization method used for analysis; Q: Cochran’s Q statistic, assessing heterogeneity among SNPs; Q_df: Degrees of freedom for the Q statistic; Q_P: P associated with the Q statistic, indicating the presence of heterogeneity.* | | | | | | |
| **Exposure** | **Outcome** | **Method** | **Q** | **Q_df** | **Q_*P*** | **MR PRESSO P** |
| WHR | PE | MR Egger | 235.77 | 238 | 5.29E-01 | 1.41E-01 |
|  |  | Inverse variance weighted | 236.49 | 239 | 5.34E-01 |  |
| PE | WHR | Inverse variance weighted | NA | NA | NA | NA |
